# Crystal structures of five compounds in the aluminium–ruthenium–silicon system

**DOI:** 10.1107/S2056989023008393

**Published:** 2023-09-29

**Authors:** Koichi Kitahara, Hiroyuki Takakura, Yutaka Iwasaki, Kaoru Kimura

**Affiliations:** a Department of Materials Science and Engineering, School of Electrical and Computer Engineering, National Defense Academy, Yokosuka 239-8686, Japan; b Department of Advanced Materials Science, Graduate School of Frontier Sciences, The University of Tokyo, Kashiwa 277-8561, Japan; c Division of Applied Physics, Faculty of Engineering, Hokkaido University, Sapporo 060-8628, Japan; d National Institute for Materials Science (NIMS), Tsukuba 305-0047, Japan; Vienna University of Technology, Austria

**Keywords:** crystal structure, aluminium–ruthenium–silicon system, single-crystal X-ray diffraction, isotypism, structural similarity, crystallographic shear, unit-cell twinning, quasicrystal approximant

## Abstract

Single crystals of five compounds with approximate compositions ∼Ru_16_(Al_0.78_Si_0.22_)_47_, (I), ∼Ru_9_(Al_0.70_Si_0.30_)_32_, (II), ∼Ru_10_(Al_0.67_Si_0.33_)_41_, (III), ∼Ru(Al_0.57_Si_0.43_)_5_, (IV), and ∼Ru_2_(Al_0.46_Si_0.54_)_9_, (V), were obtained from polycrystalline lumps mainly composed of the target compounds, and their crystal structures were determined by means of single-crystal X-ray diffraction. The crystal structure of (I) can be related to that of a cubic rational crystalline approximant to an icosa­hedral quasicrystal through crystallographic shear and then unit-cell twinning. The crystal structure of (II) is isotypic with that of a phase with composition ∼Fe_9_(Al,Si)_32_. The crystal structure of (III) is comprised of edge-sharing Ru(Al,Si)_9–11_ polyhedra with disordered chains along edges of polyhedra. The crystal structure of (IV) is of the LiIrSn_4_ type. The crystal structure of (V) can be viewed as a crystallographic shear structure derived from that of (IV).

## Abstract 2

The crystal structures of five compounds in the Al–Ru–Si system with approximate compositions ∼Ru_16_(Al_0.78_Si_0.22_)_47_ (I), ∼Ru_9_(Al_0.70_Si_0.30_)_32_ (II), ∼Ru_10_(Al_0.67_Si_0.33_)_41_ (III), ∼Ru(Al_0.57_Si_0.43_)_5_ (IV) and ∼Ru_2_(Al_0.46_Si_0.54_)_9_ (V) are presented. Notably, the crystal structure of (I) can be related to that of a cubic rational crystalline approximant to an icosa­hedral quasicrystal through crystallographic shear and then unit-cell twinning.

## 1. Chemical context

Semiconductors with complex crystal structures have attracted attention as thermoelectric materials (Snyder & Toberer, 2008 ▸; Zhang & Zhao, 2015 ▸; Liu *et al.*, 2017 ▸). To facilitate a search for such materials, knowledge on crystal structures is essential because it serves as a basis for understanding how semiconductivity and structural complexity meet in a material. Recently, a semiconducting compound with a complex crystal structure, which is considered a cubic rational crystalline approximant to an icosa­hedral quasicrystal, was discovered near the composition Al_67.6_Ru_23.5_Si_8.9_ (Iwasaki *et al.*, 2019 ▸). Following this discovery, phase equilibria in the Al–Ru–Si system at 1200 K were thoroughly investigated, and eleven new ternary phases were identified (Kitahara *et al.*, 2023 ▸). In the course of these investigations, single crystals of five new compounds with approximate compositions ∼Ru_16_(Al_0.78_Si_0.22_)_47_ (I), ∼Ru_9_(Al_0.70_Si_0.30_)_32_ (II), ∼Ru_10_(Al_0.67_Si_0.33_)_41_ (III), ∼Ru(Al_0.57_Si_0.43_)_5_ (IV) and ∼Ru_2_(Al_0.46_Si_0.54_)_9_ (V) could be obtained from polycrystalline lumps mainly composed of the target compounds. Here we report the crystal structures of these compounds.

## 2. Structural commentary

**Figure 1:**
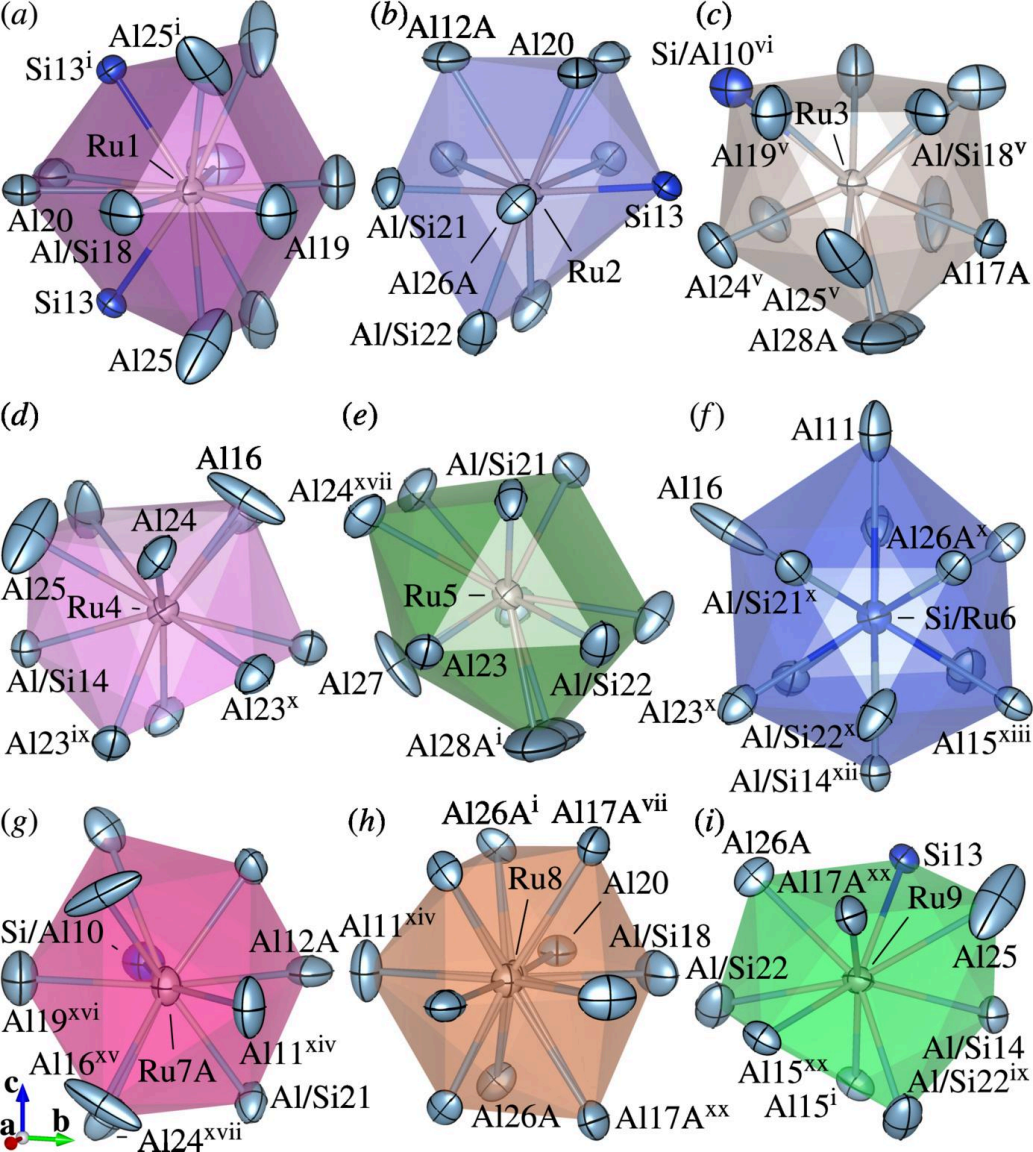
Coordination environments of (*a*) Ru1, (*b*) Ru2, (*c*) Ru3, (*d*) Ru4, (*e*) Ru5, (*f*) Si/Ru6, (*g*) Ru7, (*h*) Ru8 and (*i*) Ru9 in the crystal structure of (I) with displacement ellipsoids plotted at the 99% probability level. Unlabelled atoms in (*a*–*f*), (*g*) and (*h*) are related to the corresponding labelled atoms by (iii), (i) and (xix), respectively. Only the major disordered components are shown except for Ru8, Al20 and Al28*A*, for which two split components with equal occupancy are shown. Colour codes: Al and (Al/Si) (light blue); Ru (light brown); Si, (Si/Al) and (Si/Ru) (blue). [Symmetry codes: (i) *x*, *y*, −*z* + 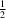 ; (iii) −*x*, *y*, *z*; (v) −*x* + 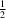 , −*y* + 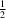 , *z* + 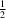 ; (vi) −*x*, −*y*, −*z* + 1; (vii) −*x* + 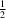 , −*y* + 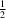 , −*z* + 1; (ix) −*x* + 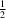 , −*y* + 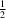 , −*z*; (*x*) −*x* + 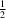 , *y* + 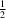 , *z*; (xii) −*x*, −*y* + 1, −*z*; (xiii) −*x*, −*y* + 1, *z* − 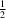 ; (xiv) *x* + 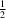 , *y* − 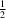 , −*z* + 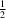 ; (xv) *x* + 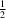 , *y* − 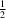 , *z*; (xvi) −*x* + 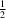 , *y* − 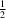 , −*z* + 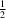 ; (xvii) −*x* + 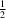 , *y* − 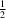 , *z*; (xix) −*x* + 1, *y*, *z*; (xx) −*x* + 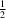 , −*y* + 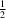 , *z* − 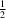 .]

**Figure 2:**
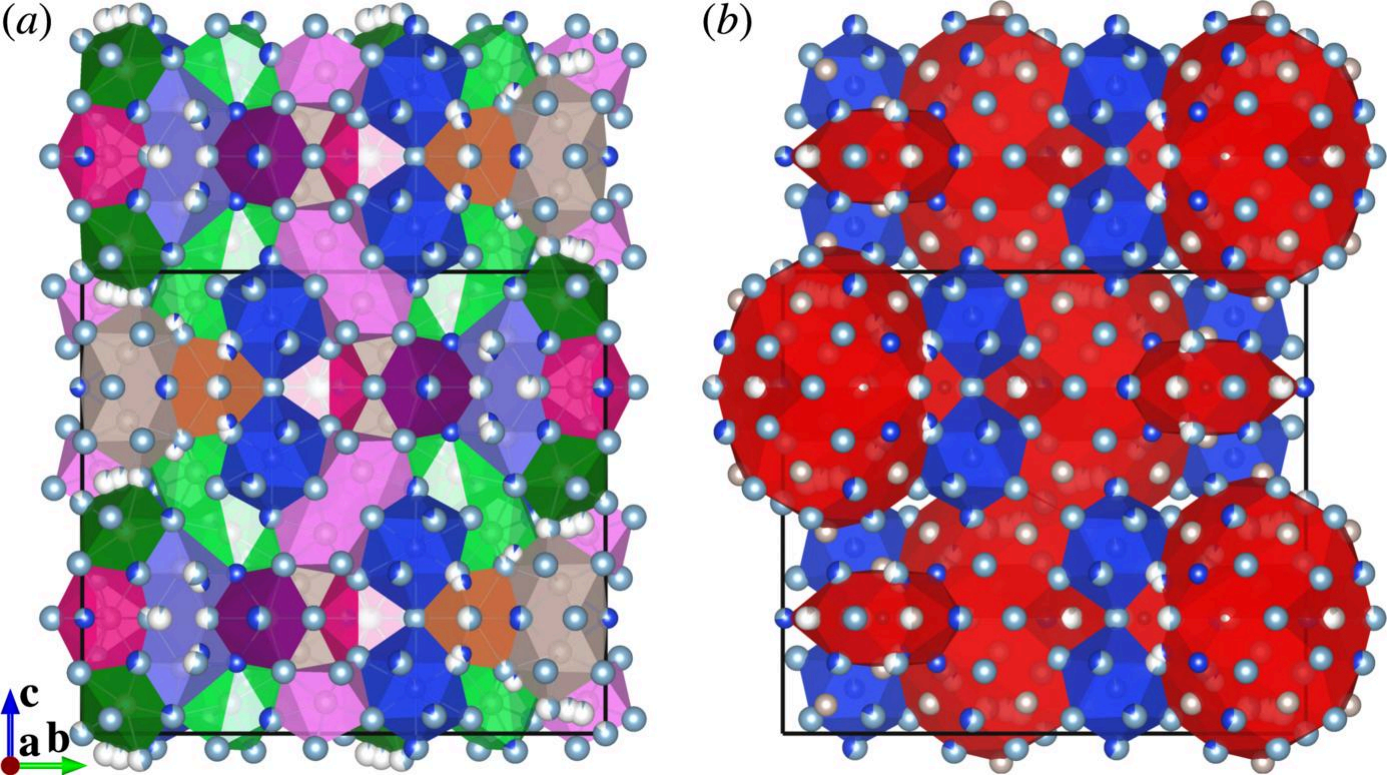
Packing diagrams for (I) based on (*a*) the coordination polyhedra given in Fig. 1 ▸ and (*b*) larger structural units (clusters).

The crystal structure of (I) consists of Ru-centred polyhedra with coordination numbers ranging from nine to eleven [Fig. 1 ▸(*a*–*e*, *g*–*i*)] and (Si,Ru)-centred icosa­hedra [Fig. 1 ▸(*f*)]. Each polyhedron is connected to three, four, five, six or fourteen others through face-sharing. A sensible represent­ation of the crystal packing in (I) is obtained by using large structural units (clusters) [Fig. 2 ▸(*b*)] instead of using coordination polyhedra [Fig. 2 ▸(*a*)].

**Figure 3:**
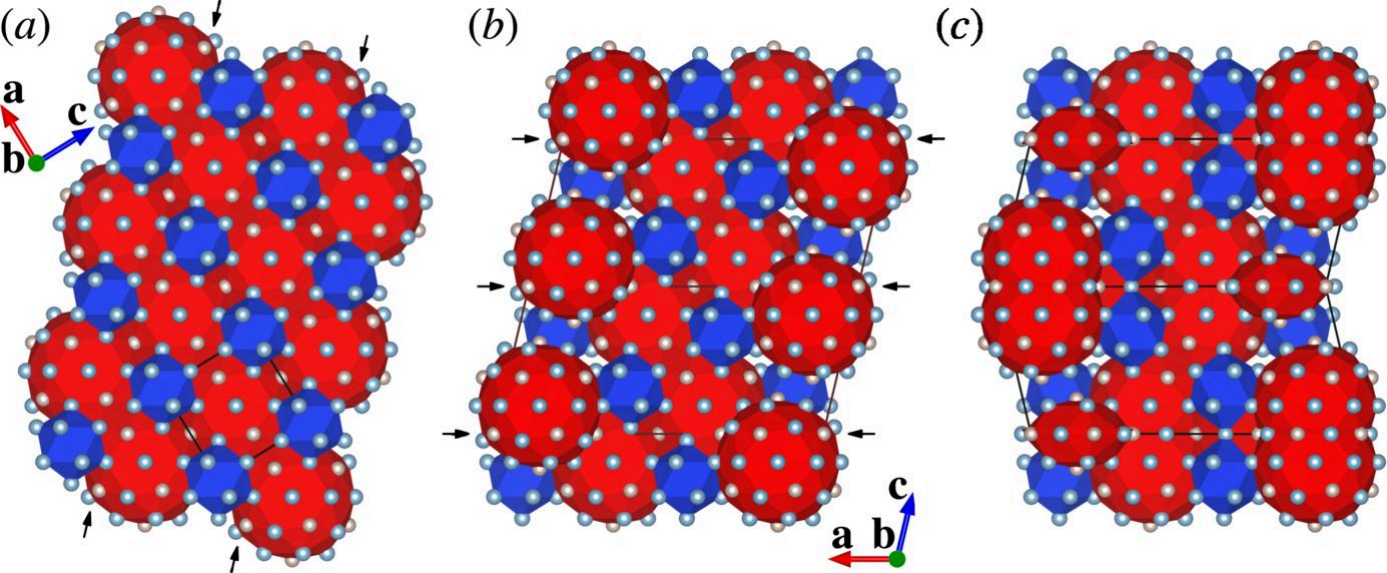
The structural units given in Fig. 2 ▸(*b*) as derived from (*a*) the parent cubic approximant structure by applying (*b*) crystallographic shear and then (*c*) unit-cell twinning. Shear planes [(10 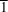 ) planes of the parent structure] are indicated by arrows in (*a*). Twin planes [(001) planes of the sheared structure] are indicated by arrows in (*b*). Colour codes: icosa­hedral clusters (blue); pseudo-Mackay clusters (red).

The crystal structure of (I) can be related to that of a rational crystalline approximant to an icosa­hedral quasicrystal through crystallographic shear and then unit-cell twinning, as detailed in the following. The crystallographic shear and unit-cell twinning are two of the most important structure building operations by which a recombination structure is derived from a simpler parent structure (Lima-de-Faria *et al.*, 1990 ▸). In this case, the parent structure is a 1/0 cubic approximant to a Mackay-type icosa­hedral quasicrystal. Crystal structures of this type are known for the phases with approximate compositions ∼RhAl_2.63_ (ICSD 406525; Grin *et al.*, 1997 ▸), ∼IrAl_2.75_ (ICSD 406526; Grin *et al.*, 1997 ▸), ∼Al_20.5_Ru_5.1_Ni_2.9_ (ICSD 230569; Simura *et al.*, 2017 ▸) and ∼Al_5.64_Fe_0.96_Pd_1.04_ (ICSD 45248; Li & Fan, 2019 ▸). The crystal structure of the semiconducting compound ∼Al_67.6_Ru_23.5_Si_8.9_ is also considered to fall into this type (Iwasaki *et al.*, 2019 ▸). Fig. 3 ▸(*a*) shows an idealized crystal structure of this cubic approximant, which can be viewed as a CsCl-type packing of two types of clusters, icosa­hedral clusters and pseudo-Mackay clusters (Simura *et al.*, 2017 ▸). Fig. 3 ▸(*b*) shows a crystallographic shear structure derived from the parent structure. The shear planes are (10 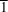 ) planes of the parent structure [Fig. 3 ▸(*a*)], and the shear operation was applied at cluster level. Fig. 3 ▸(*c*) shows the crystal structure obtained by applying unit-cell twinning to the sheared structure. The twin planes are (001) planes of the sheared structure [Fig. 3 ▸(*b*)], and the twin operation was applied at the atomic level. Two constituent parts of the twinned structure are related by reflection across the twin planes as the mirror planes. Atoms close to each twin plane were shifted onto the plane prior to the twinning. The twinned structure is comparable to the cluster-based representation of (I) shown in Fig. 2 ▸(*b*). Note that the shear structure shown in Fig. 3 ▸(*b*) can also be obtained through so-called linear phason strain (Yamamoto, 1996 ▸) instead of crystallographic shear and hence directly relates to an icosa­hedral quasicrystal as a monoclinic approximant.

**Figure 4:**
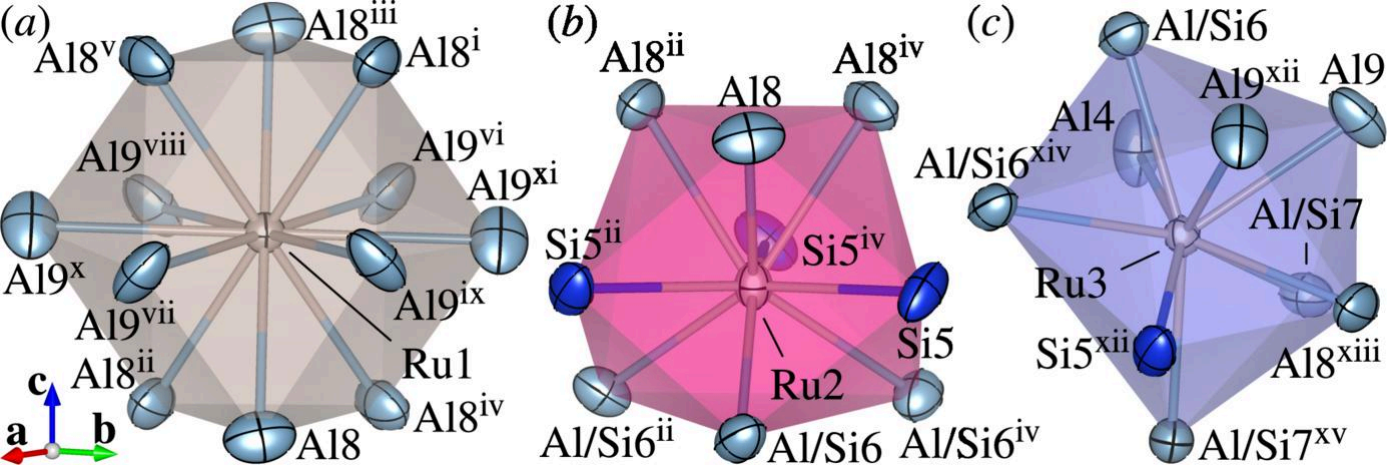
Coordination environments of (*a*) Ru1, (*b*) Ru2 and (*c*) Ru3 in the crystal structure of (II) with displacement ellipsoids plotted at the 99% probability level. Colour codes: Al and Al/Si (light blue); Ru (light brown); Si (blue). [Symmetry codes: (i) *x* − *y*, *x*, −*z* + 1; (ii) −*x* + *y*, −*x*, *z*; (iii) −*x*, −*y*, −*z* + 1; (iv) −*y*, *x* − *y*, *z*; (v) *y*, −*x* + *y*, −*z* + 1; (vi) *y* − 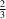 , −*x* + *y* − 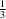 , −*z* + 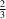 ; (vii) −*y* + 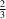 , *x* − *y* + 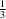 , *z* + 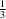 ; (viii) *x* − 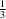 , *y* − 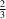 , *z* + 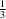 ; (ix) −*x* + 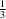 , −*y* + 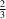 , −*z* + 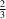 ; (*x*) *x* − *y* + 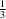 , *x* − 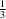 , −*z* + 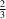 ; (xi) −*x* + *y* − 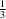 , −*x* + 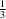 , *z* + 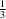 ; (xii) *x* − *y* + 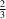 , *x* + 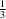 , −*z* + 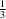 ; (xiii) −*x* + *y* + 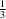 , −*x* + 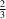 , *z* − 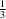 ; (xiv) −*x* + 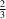 , −*y* + 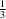 , −*z* + 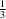 ; (xv) *y*, −*x* + *y*, −*z*.]

The crystal structure of (II) consists of two types of Ru-centred polyhedra, RuAl_12_ cubocta­hedra [Fig. 4 ▸(*a*)] and Ru(Al,Si)_9_ square-face tricapped trigonal prisms [Fig. 4 ▸(*b*, *c*)]. Each cubocta­hedron is surrounded by eight square-face tricapped trigonal prisms sharing its eight triangular faces. Each square-face tricapped trigonal prism is in turn connected to others through edge-sharing.

**Figure 5:**
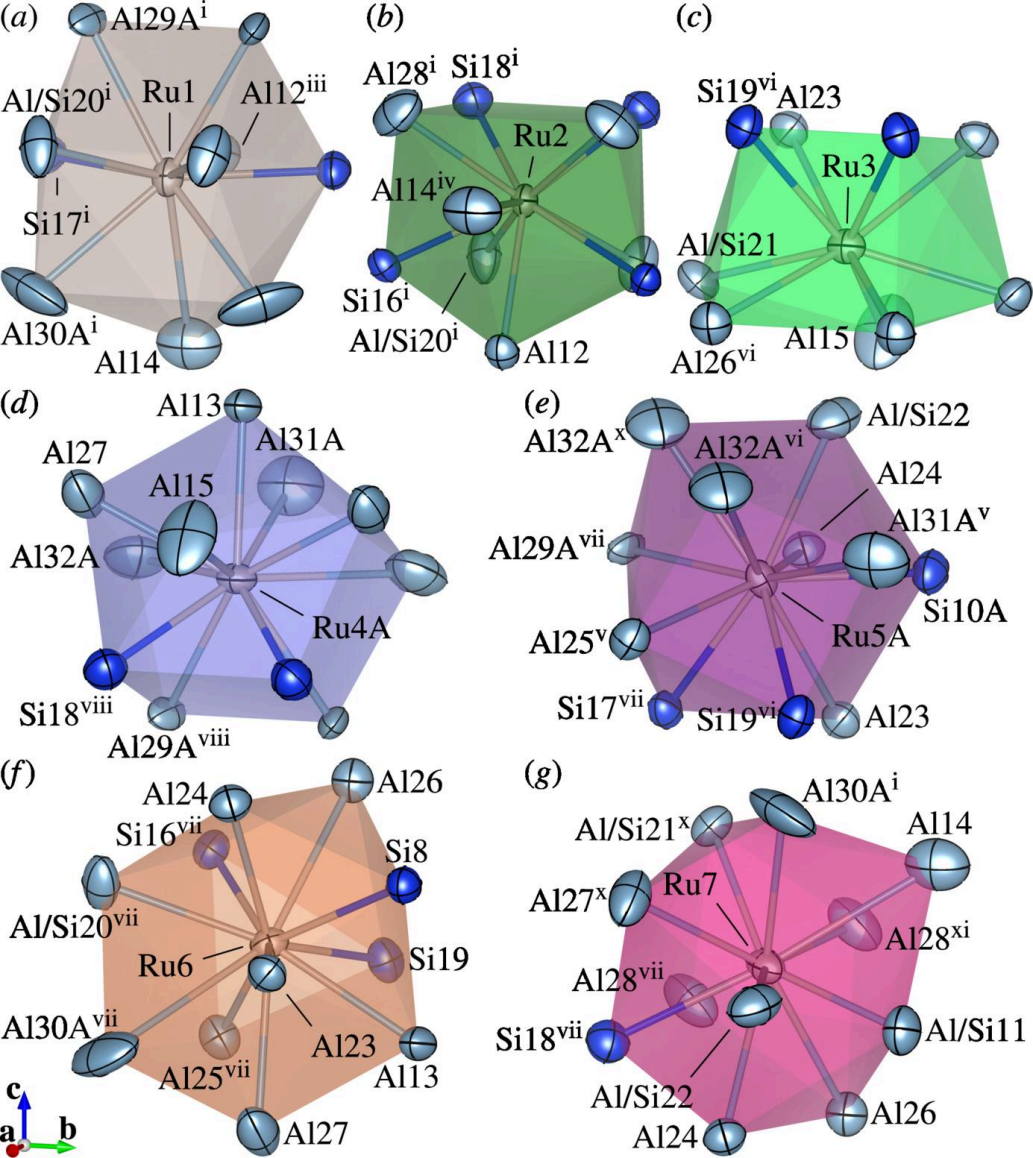
Coordination environments of (*a*) Ru1, (*b*) Ru2, (*c*) Ru3, (*d*) Ru4, (*e*) Ru5, (*f*) Ru6 and (*g*) Ru7 in the crystal structure of (III) with displacement ellipsoids plotted at the 99% probability level. Unlabelled atoms in (*a*–*d*) are related to the corresponding labelled atoms by (vii). Only the major disordered components are shown. Colour codes: Al and Al/Si (light blue); Ru (light brown); Si (blue). [Symmetry codes: (i) −*x* + 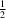 , *y* − 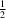 , *z* + 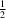 ; (iii) *x* − 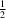 , *y*, −*z* + 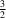 ; (iv) *x* + 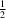 , *y*, −*z* + 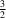 ; (v) *x* + 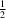 , −*y* + 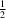 , −*z* + 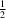 ; (vi) *x* + 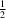 , *y*, −*z* + 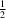 ; (vii) *x*, −*y* + 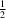 , *z*; (viii) −*x* + 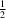 , *y* − 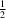 , *z* − 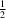 ; (*x*) −*x* + 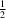 , −*y*, *z* + 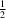 ; (xi) −*x*, *y* − 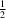 , −*z* + 1.]

**Figure 6:**
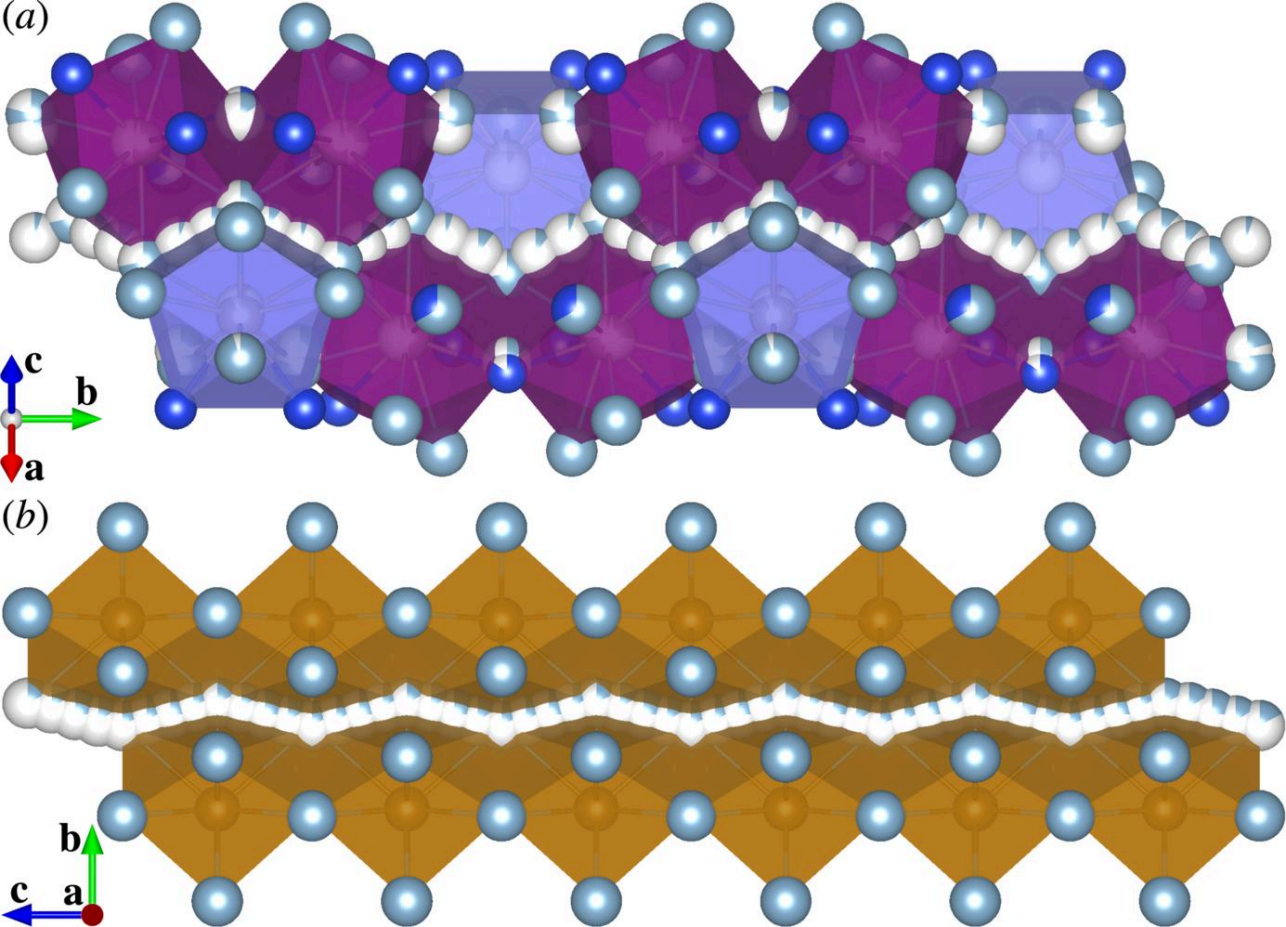
Disordered chains along polyhedral edges (*a*) in the crystal structure of (III) and (*b*) in ∼Fe_2_Al_5_ (CCDC 1880447; Vinokur *et al.*, 2019 ▸). Colour codes: Al (light blue); Fe (brown); Ru (light brown); Si (blue).

The crystal structure of (III) is comprised of Ru(Al,Si)_10_ [Fig. 5 ▸(*a*, *b*)], Ru(Al,Si)_9_ [Fig. 5 ▸(*c*)] and Ru(Al,Si)_11_ [Fig. 5 ▸(*d*–*g*)] polyhedra. Unlike the polyhedra in (I) and (II), no faces are shared between these polyhedra. These polyhedra are connected through edge-sharing. As an unusual feature, extensive positional disorder within a chain motif along edges of Ru4- and Ru5-centred polyhedra [Fig. 6 ▸(*a*)] is found in (III). A similar disordered chain structure [Fig. 6 ▸(*b*)] was also reported for the phase with approximate composition ∼Fe_2_Al_5_ (Burkhardt *et al.*, 1994 ▸; Vinokur *et al.*, 2019 ▸).

**Figure 7:**
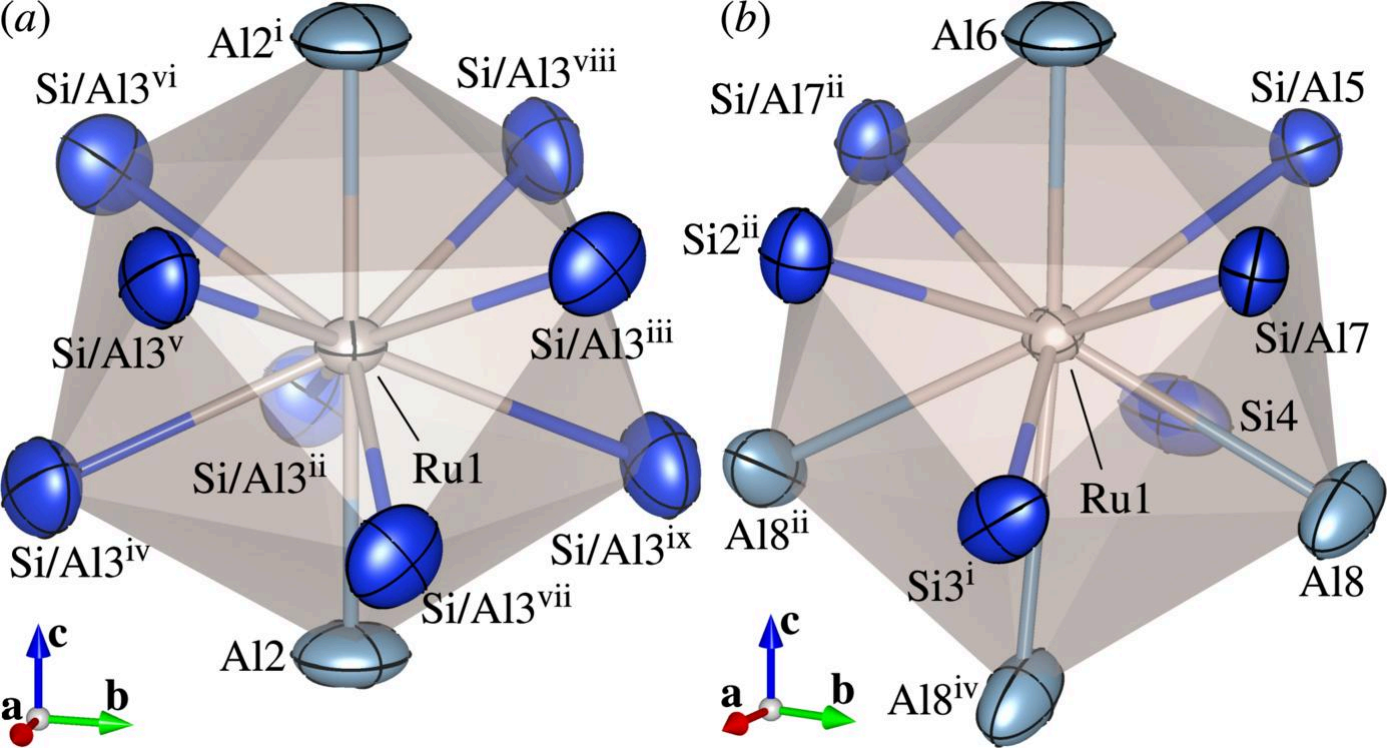
Coordination environments of (*a*) Ru1 in the crystal structure of (IV) [symmetry codes: (i) −*x*, *y*, −*z* + 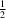 ; (ii) *y* − 1, −*x*, *z*; (iii) *y* − 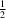 , −*x* + 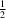 , −*z* + 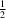 ; (iv) *x*, *y* − 1, *z*; (v) −*x* + 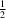 , −*y* + 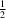 , −*z* + 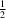 ; (vi) −*y* + 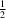 , *x* − 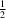 , −*z* + 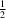 ; (vii) −*y* + 1, *x*, *z*; (viii) *x* − 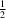 , *y* − 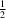 , −*z* + 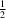 ; (ix) −*x*, −*y* + 1, *z*] and (*b*) Ru1 in the crystal structure of (V) [symmetry codes: (i) −*x* + 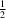 , −*y* + 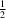 , *z* − 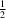 ; (ii) −*x* + 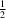 , *y* − 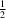 , *z*; (iv) −*x* + 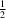 , −*y* + 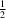 , −*z*] with displacement ellipsoids plotted at the 99% probability level. Colour codes: Al (light blue); Ru (light brown); Si and Si/Al (blue).

**Figure 8:**
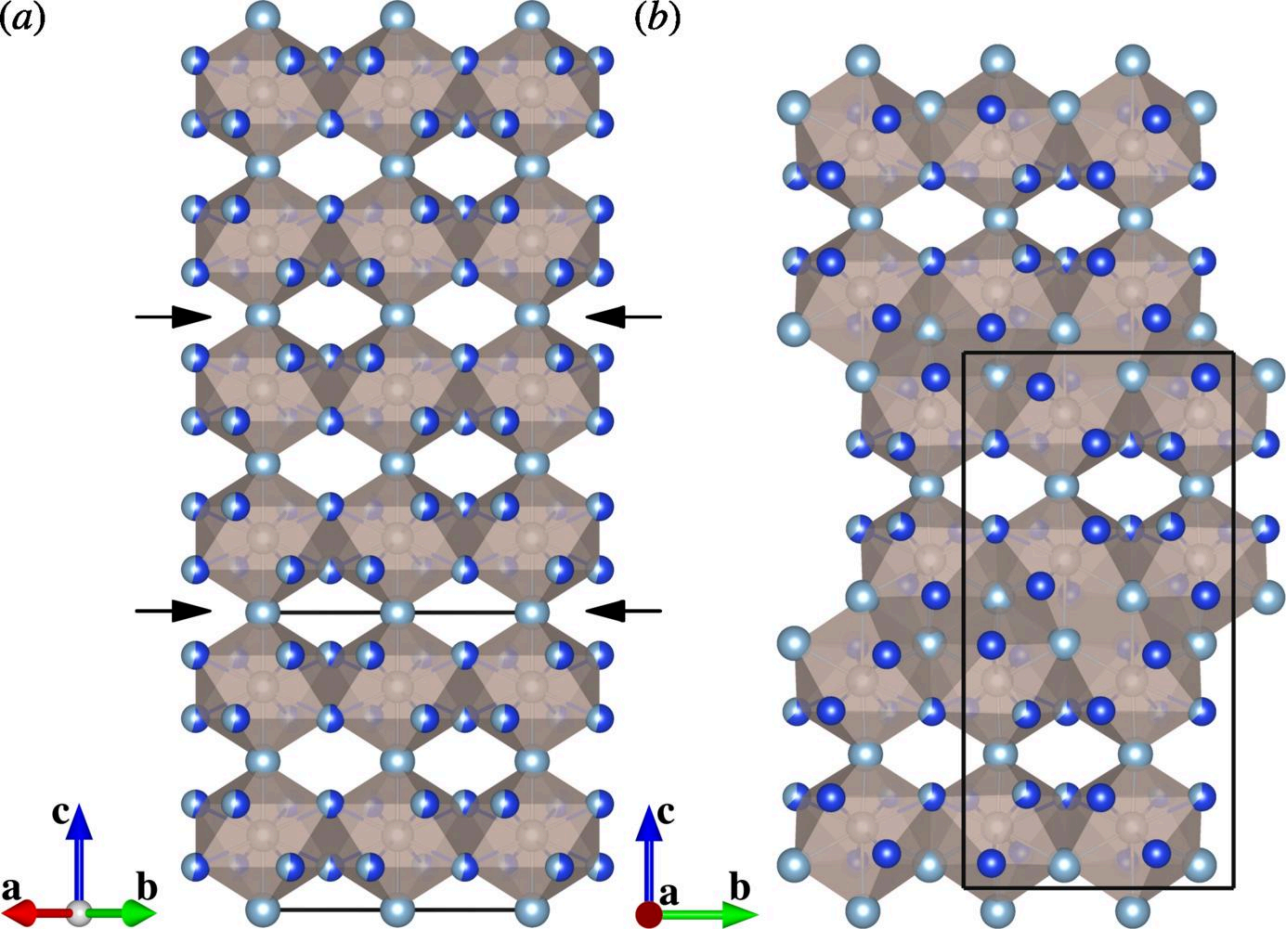
Packing diagrams for the crystal structures (*a*) of (IV) and (*b*) of (V). Crystallographic shear planes [(001) planes of (IV)] are indicated by arrows in (*a*). Colour codes: Al (light blue); Ru (light brown); Si (blue).

The crystal structures of (IV) and (V) consist of Ru(Al,Si)_10_ bicapped square anti­prisms [Fig. 7 ▸(*a*, *b*)]. In both crystal structures, the two cap positions of each polyhedron [Al2^i^ and Al2 in Fig. 7 ▸(*a*); Al6 and Al8^iv^ in Fig. 7 ▸(*b*)] are occupied solely by Al, and each polyhedron is connected to four others in the (001) plane through edge-sharing. Along the [001] direction, each polyhedron is connected to two others sharing its vertices at the two cap positions in (IV) [Fig. 8 ▸(*a*)], while each polyhedron is connected to one another through vertex-sharing and two others through edge-sharing in (V) [Fig. 8 ▸(*b*)]. The structure of (V) can be viewed as a crystallographic shear structure derived from that of (IV). The corresponding shear planes are (001) planes of (IV) [Fig. 8 ▸(*a*)].

## 3. Database survey

Searches for isotypic crystal structures to those of the five compounds were carried out using the Inorganic Crystal Structure Database (ICSD, version 4.9.0; Zagorac *et al.*, 2019 ▸). For (I), (III) and (V), queries for structures with the Pearson symbols *oS*250–254, *oP*200–204 and *oS*88, respectively, were made, but no entries for isotypic crystals were found. For (II), a query for *hR*41 was made, and three nearly identical structures (ICSD 99169; Sugiyama *et al.*, 2004 ▸; ICSD 423182, 423183; Roger *et al.*, 2011 ▸) of the crystal ∼Fe_9_(Al,Si)_32_ were found. Although isotypism is indicated between (II) and ∼Fe_9_(Al,Si)_32_, unlike in (II), the centres of the cubocta­hedra are not occupied solely by transition-metal atoms in these structures. For (IV), a query for *tI*24 was made, and some isotypic crystals of the LiIrSn_4_ type (ICSD 412252; Wu *et al.*, 2002 ▸) were found. Limiting the results to Al-containing crystal structures, isotypic crystals in the Al–Fe–Si (ICSD 79709; Gueneau *et al.*, 1995 ▸; ICSD 4837, 4839; Zhou *et al.*, 2018 ▸), Al–Fe–Ge (ICSD 235910; Reisinger *et al.*, 2018 ▸) and Al–Ge–Mn (ICSD 8608; Sasaki *et al.*, 2019 ▸) systems were found. In these structures and that of (IV), the Li sites of the LiIrSn_4_-type structure are occupied solely by Al, whereas the Sn sites are occupied by (Al,Si) or (Al,Ge).

## 4. Synthesis and crystallization

Polycrystalline samples with the nominal compositions Al_58.0_Ru_25.3_Si_16.7_ for (I) and Al_37.5_Ru_17.8_Si_44.7_ for (V) were prepared from powders of aluminium (Kojundo Chemical Lab., Japan, 99.9%), ruthenium (Tanaka Kikinzoku Kogyo K. K., Japan, 99.90% or purer) and silicon (Kojundo Chemical Lab., Japan, 99.99% or purer) using arc melting in an argon atmosphere (NEV-ACD-05, Nissin Giken Corporation, Japan). Each sample was then wrapped in tantalum foil, sealed in a silica tube filled with argon and annealed in a furnace at 1200 K for approximately 264 h, followed by water quenching. Powders for polycrystalline samples with the nominal compositions Al_55.5_Ru_21.7_Si_22.8_ for (II), Al_55.0_Ru_19.2_Si_25.8_ for (III) and Al_48.0_Ru_16.2_Si_35.8_ for (IV) were mechanically compacted without melting. Each sample was then placed in an aluminium nitride crucible, sealed in a silica tube filled with argon and reacted in a furnace at 1200 K for approximately 160 h or longer, followed by water quenching. Single crystals were selected from crushed fragments of the polycrystalline samples (lumps), which are mainly composed of the target compounds. The compositions of the target compounds in the samples were analysed as described elsewhere (Kitahara *et al.*, 2023 ▸) using an energy-dispersive spectrometer (X-Max, Oxford Instruments, UK) equipped in a scanning electron microscope (SU6600, Hitachi High-Technologies Corporation, Japan). Determined compositions for equilibria at 1200 K are Al_58.2 (5)_Ru_25.1 (4)_Si_16.7 (3)_ for (I), Al_55.2 (6)_Ru_21.6 (4)_Si_23.1 (7)_ for (II), Al_54.1 (5)_Ru_19.6 (3)_Si_26.3 (4)_ for (III), Al_47.3 (6)_Ru_16.3 (3)_Si_36.4 (6)_ for (IV) and Al_38.0 (5)_Ru_17.8 (3)_Si_44.2 (6)_ for (V). These compositions are similar to the nominal compositions and consistent with the approximate single-phase regions [from Al_62_Ru_25_Si_13_ to Al_56_Ru_26_Si_18_ for (I), from Al_57_Ru_22_Si_21_ to Al_54_Ru_22_Si_24_ for (II), from Al_55_Ru_20_Si_25_ to Al_54_Ru_20_Si_26_ for (III), from Al_49_Ru_17_Si_34_ to Al_47_Ru_17_Si_36_ for (IV) and from Al_41_Ru_18_Si_41_ to Al_37_Ru_18_Si_45_ for (V)] (Kitahara *et al.*, 2023 ▸).

## 5. Refinement

Crystal data, data collection and structure refinement details are summarized in Table 1 ▸.

For (I) and (III), significant residual electron density peaks were found around some sites after routine structure refinement procedures. To account for these peaks, some split sites were introduced for (I) [Ru7*B* from Ru7*A*; Ru8 (Wyckoff position changed into 8*g* from 4*c*); Al12*B* from Al12*A*; Si17*B* from Al17*A*; Si26*B* from Al26*A*; Al28*A* (16*h* from 8*f*); Al28*B* and Al28*C* from Al28*A*] and (III) [Ru4*B* from Ru4*A*; Ru5*B* from Ru5*A*; Si9*B* from Si9*A*; Al10*B* from Si10*A*; Al29*B* from Al29*A*; Al30*B* from Al30*A*; Al31*B* and Al31*C* from Al31*A*; Al32*B* from Al32*A*]. Anisotropic displacement parameters were not introduced for minor split components [Ru7*B*, Al12*B*, Si17*B*, Si26*B*, Al28*B* and Al28*C* in (I); Ru4*B*, Ru5*B*, Al10*B*, Al29*B*, Al30*B*, Al31*B*, Al31*C* and Al32*B* in (III)] except for Si9*B* in (III).

Choices of Al, Si or (Al,Si) mixing were basically based on the site-occupancy factors (SOFs). After routine structure-refinement procedures and adding split sites, the SOF of each site was refined independently one by one. (Al,Si) mixing was introduced for some sites for (I) (Si/Al10, Al/Si14, Al/Si18, Al/Si21 and Al/Si22), (II) (Al/Si6 and Al/Si7), (III) (Al/Si11, Al/Si20, Al/Si21 and Al/Si22), (IV) (Si/Al3) and (V) (Si/Al5 and Si/Al7) because the SOF becomes significantly higher than 1 with Al occupation only and lower than 1 with Si occupation only for these sites. The positions and displacement parameters of mixing components were constrained using EXYZ and EADP instructions of *SHELXL* (Sheldrick, 2015*b* ▸). For split sites, (Al,Si) mixing was not introduced, and Al or Si was chosen for each site so that the sum of the SOFs of the split components are close to 1. Choice of Si for Si/Ru6 in (I) is rather arbitrary, and it may be Al or even a vacancy.

After assigning chemical species to each site, the sum of the SOFs for each group of mixed or split sites was constrained to 1 unless it was significantly lower than 1. Sites with SOFs lower than 1 were found for (I) (Al27) and (III) (Al15), and the SOFs for these sites were freely refined. For sites along disordered chains in (III) [Al31*A*, Al31*B*, Al31*C*, Al32*A* and Al32*B*; see also *Structural commentary* and Fig. 6 ▸(*a*)], there is no *a priori* expected value for the sum of the SOFs, and therefore the SOFs of these sites were refined without any constraints. For these partially occupied sites, the assignment as Al is by no means justified, and some of them may be occupied by Si.

Since it is generally difficult to distinguish between Al and Si solely from X-ray diffraction data, restraints on the chemical compositions based on the chemical analysis data including their uncertainties (see *Synthesis and crystallization*) were introduced using SUMP instructions of *SHELXL* (Sheldrick, 2015*b* ▸) for the final refinement cycles. The compositions deduced from the final refinement are Al_57.8 (3)_Ru_25.6_Si_16.6 (3)_ for (I), Al_54.8 (5)_Ru_22.0_Si_23.3 (5)_ for (II), Al_53.6 (3)_Ru_19.7_Si_26.6 (3)_ for (III), Al_47.2 (5)_Ru_16.7_Si_36.1 (5)_ for (IV) and Al_37.2 (4)_Ru_18.2_Si_44.6 (4)_ for (V). These compositions are consistent with the chemical analysis data.

## Supplementary Material

Crystal structure: contains datablock(s) I, II, III, IV, V, global. DOI: 10.1107/S2056989023008393/wm5690sup1.cif

Structure factors: contains datablock(s) I. DOI: 10.1107/S2056989023008393/wm5690Isup2.hkl

Structure factors: contains datablock(s) II. DOI: 10.1107/S2056989023008393/wm5690IIsup3.hkl

Structure factors: contains datablock(s) III. DOI: 10.1107/S2056989023008393/wm5690IIIsup4.hkl

Structure factors: contains datablock(s) IV. DOI: 10.1107/S2056989023008393/wm5690IVsup5.hkl

Structure factors: contains datablock(s) V. DOI: 10.1107/S2056989023008393/wm5690Vsup6.hkl

CCDC references: 2297119, 2297118, 2297117, 2297116, 2297115

Additional supporting information:  crystallographic information; 3D view; checkCIF report

## Figures and Tables

**Table 1:** Experimental details

	(I)	(II)	(III)	(IV)	(V)
Crystal data					
Chemical formula	∼Ru_16_(Al_0.78_Si_0.22_)_47_	∼Ru_9_(Al_0.70_Si_0.30_)_32_	∼Ru_10_(Al_0.67_Si_0.33_)_41_	∼Ru(Al_0.57_Si_0.43_)_5_	∼Ru_2_(Al_0.46_Si_0.54_)_9_
*M*_r_	2900.59	1783.65	2122.53	238.38	450.41
Crystal system, space group	Orthorhombic, *C* *m* *c* *m*	Trigonal, *R* 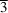	Orthorhombic, *P* *n* *m* *a*	Tetragonal, *I*4/*m* *c* *m*	Orthorhombic, *C* *m* *c* *m*
Temperature (K)	301	296	298	297	301
*a*, *b*, *c* (Å)	7.6217 (2), 23.4434 (6), 20.6877 (6)	10.4479 (2), 10.4479 (2), 19.6774 (4)	15.0794 (2), 11.8713 (2), 16.9291 (3)	6.20079 (8), 6.20079 (8), 9.6937 (2)	8.63058 (14), 8.79888 (15), 17.4620 (3)
α, β, γ (°)	90, 90, 90	90, 90, 120	90, 90, 90	90, 90, 90	90, 90, 90
*V* (Å^3^)	3696.46 (19)	1860.17 (7)	3030.50 (9)	372.72 (1)	1326.05 (4)
*Z*	4	3	4	4	8
Radiation type	Mo *K*α	Mo *K*α	Mo *K*α	Mo *K*α	Mo *K*α
μ (mm^−1^)	7.59	6.59	6.18	5.32	5.88
Crystal size (mm)	0.04 × 0.02 × 0.02	0.03 × 0.03 × 0.03	0.04 × 0.02 × 0.02	0.05 × 0.03 × 0.02	0.05 × 0.04 × 0.04

Data collection					
Diffractometer	XtaLAB Synergy R, HyPix	XtaLAB Synergy R, HyPix	XtaLAB Synergy R, HyPix	XtaLAB Synergy R, HyPix	XtaLAB Synergy R, HyPix
Absorption correction	Gaussian (*CrysAlis PRO*; Matsumoto *et al.*, 2021 ▸)	Gaussian (*CrysAlis PRO*; Matsumoto *et al.*, 2021 ▸)	Gaussian (*CrysAlis PRO*; Matsumoto *et al.*, 2021 ▸)	Gaussian (*CrysAlis PRO*; Matsumoto *et al.*, 2021 ▸)	Gaussian (*CrysAlis PRO*; Matsumoto *et al.*, 2021 ▸)
*T*_min_, *T*_max_	0.824, 0.924	0.868, 0.879	0.867, 0.929	0.833, 0.906	0.826, 0.868
No. of measured, independent and observed [*I* > 2σ(*I*)] reflections	11822, 2616, 2280	14255, 1193, 1127	33654, 5672, 4940	8063, 295, 279	17672, 1029, 1001
*R*_int_	0.020	0.027	0.022	0.029	0.016
(sin θ/λ)_max_ (Å^−1^)	0.718	0.720	0.781	0.864	0.719

Refinement					
*R*[*F* ^2^ > 2σ(*F* ^2^)], *wR*(*F* ^2^), *S*	0.015, 0.032, 1.04	0.013, 0.024, 1.08	0.015, 0.026, 1.01	0.009, 0.021, 1.21	0.011, 0.027, 1.31
No. of reflections	2616	1193	5672	295	1029
No. of parameters	218	66	303	13	61
No. of restraints	5	2	2	2	2
Δρ_max_, Δρ_min_ (e Å^−3^)	0.64, −0.69	0.45, −0.53	0.78, −0.74	0.44, −0.42	0.37, −0.42

Computer programs: *CrysAlis PRO* (Matsumoto *et al.*, 2021 ▸), *SHELXT* (Sheldrick, 2015*a* ▸), *SHELXL* (Sheldrick, 2015*b* ▸), *VESTA* (Momma & Izumi, 2011 ▸) and *publCIF* (Westrip, 2010 ▸).

## supplementary crystallographic information

### Hexatriacontaaluminium hexadecaruthenium decasilicon (I) Crystal data

**Table:**

~Ru_16_(Al_0.78_Si_0.22_)_47_	*D*_x_ = 5.212 Mg m^−^^3^
*M**_r_* = 2900.59	Mo *K*α radiation, λ = 0.71073 Å
Orthorhombic, *C**m**c**m*	Cell parameters from 5906 reflections
*a* = 7.6217 (2) Å	θ = 2.6–30.8°
*b* = 23.4434 (6) Å	µ = 7.59 mm^−^^1^
*c* = 20.6877 (6) Å	*T* = 301 K
*V* = 3696.46 (19) Å^3^	Prism, metallic light colourless
*Z* = 4	0.04 × 0.02 × 0.02 mm
*F*(000) = 5237.80	

### Hexatriacontaaluminium hexadecaruthenium decasilicon (I) Data collection

**Table:**

XtaLAB Synergy R, HyPix diffractometer	2616 independent reflections
Radiation source: micro-focus sealed X-ray tube, Mova (Mo) X-ray Source	2280 reflections with *I* > 2σ(*I*)
Mirror monochromator	*R*_int_ = 0.020
Detector resolution: 10.0000 pixels mm^-1^	θ_max_ = 30.7°, θ_min_ = 1.7°
ω scans	*h* = −9→7
Absorption correction: gaussian (CrysAlisPro; Matsumoto *et al.*, 2021)	*k* = −32→33
*T*_min_ = 0.824, *T*_max_ = 0.924	*l* = −22→27
11822 measured reflections	

### Hexatriacontaaluminium hexadecaruthenium decasilicon (I) Refinement

**Table:**

Refinement on *F*^2^	Primary atom site location: dual
Least-squares matrix: full	Secondary atom site location: difference Fourier map
*R*[*F*^2^ > 2σ(*F*^2^)] = 0.015	*w* = 1/[σ^2^(*F*_o_^2^) + (0.015*P*)^2^ + 1.7856*P*] where *P* = (*F*_o_^2^ + 2*F*_c_^2^)/3
*wR*(*F*^2^) = 0.032	(Δ/σ)_max_ = 0.002
*S* = 1.04	Δρ_max_ = 0.64 e Å^−^^3^
2616 reflections	Δρ_min_ = −0.69 e Å^−^^3^
218 parameters	Extinction correction: SHELXL-2019/3 (Sheldrick, 2015b), Fc^*^=kFc[1+0.001xFc^2^λ^3^/sin(2θ)]^-1/4^
5 restraints	Extinction coefficient: 0.000036 (4)

### Hexatriacontaaluminium hexadecaruthenium decasilicon (I) Special details

**Table:**

Geometry. All esds (except the esd in the dihedral angle between two l.s. planes) are estimated using the full covariance matrix. The cell esds are taken into account individually in the estimation of esds in distances, angles and torsion angles; correlations between esds in cell parameters are only used when they are defined by crystal symmetry. An approximate (isotropic) treatment of cell esds is used for estimating esds involving l.s. planes.

### Hexatriacontaaluminium hexadecaruthenium decasilicon (I) Fractional atomic coordinates and isotropic or equivalent isotropic displacement parameters (Å^2^)

**Table:**

	*x*	*y*	*z*	*U*_iso_*/*U*_eq_	Occ. (<1)
Ru1	0.000000	0.35437 (2)	0.250000	0.00702 (8)	
Ru2	0.000000	0.19071 (2)	0.14245 (2)	0.00523 (6)	
Ru3	0.000000	0.08703 (2)	0.67676 (2)	0.00497 (6)	
Ru4	0.000000	0.46501 (2)	0.06755 (2)	0.00580 (6)	
Ru5	0.000000	0.08260 (2)	0.06182 (2)	0.00605 (6)	
Si6	0.000000	0.63703 (3)	0.09750 (4)	0.0067 (3)	0.9481 (19)
Ru6	0.000000	0.63703 (3)	0.09750 (4)	0.0067 (3)	0.0519 (19)
Ru7A	0.2872 (5)	0.05250 (15)	0.250000	0.0071 (3)	0.87 (3)
Ru7B	0.308 (2)	0.0461 (8)	0.250000	0.0123 (19)*	0.13 (3)
Ru8	0.4748 (2)	0.24633 (2)	0.250000	0.0057 (4)	0.5
Ru9	0.20167 (2)	0.28208 (2)	0.06561 (2)	0.00560 (5)	
Si10	0.000000	0.00419 (5)	0.250000	0.0088 (6)	0.73 (12)
Al10	0.000000	0.00419 (5)	0.250000	0.0088 (6)	0.27 (12)
Al11	0.000000	0.63795 (6)	0.250000	0.0132 (3)	
Al12A	0.000000	0.12915 (10)	0.250000	0.0126 (9)	0.856 (12)
Al12B	0.079 (3)	0.1477 (10)	0.250000	0.020 (5)*	0.072 (6)
Si13	0.000000	0.29501 (3)	0.15414 (4)	0.00569 (17)	
Al14	0.000000	0.36199 (4)	0.03013 (4)	0.0068 (4)	0.59 (8)
Si14	0.000000	0.36199 (4)	0.03013 (4)	0.0068 (4)	0.41 (8)
Al15	0.000000	0.25809 (4)	0.53285 (5)	0.00662 (19)	
Al16	0.000000	0.52766 (5)	0.16337 (5)	0.0189 (2)	
Al17A	0.000000	0.19089 (7)	0.63750 (11)	0.0091 (5)	0.908 (9)
Si17B	0.000000	0.1757 (7)	0.6124 (11)	0.012 (4)*	0.092 (9)
Al18	0.32754 (14)	0.33805 (4)	0.250000	0.0126 (5)	0.52 (9)
Si18	0.32754 (14)	0.33805 (4)	0.250000	0.0126 (5)	0.48 (9)
Al19	0.20942 (15)	0.44221 (4)	0.250000	0.0133 (2)	
Al20	0.1113 (3)	0.23957 (8)	0.250000	0.0096 (4)	0.5
Al21	0.20919 (10)	0.10789 (3)	0.15319 (3)	0.0089 (3)	0.63 (5)
Si21	0.20919 (10)	0.10789 (3)	0.15319 (3)	0.0089 (3)	0.37 (5)
Al22	0.18810 (10)	0.17255 (3)	0.03759 (4)	0.0119 (3)	0.81 (6)
Si22	0.18810 (10)	0.17255 (3)	0.03759 (4)	0.0119 (3)	0.19 (6)
Al23	0.31517 (10)	0.05413 (3)	0.03152 (3)	0.00897 (14)	
Al24	0.31724 (10)	0.49470 (3)	0.13170 (4)	0.01044 (15)	
Al25	0.18885 (12)	0.38526 (3)	0.13490 (4)	0.0223 (2)	
Al26A	0.3202 (4)	0.21649 (10)	0.1531 (2)	0.0070 (7)	0.593 (13)
Si26B	0.2898 (5)	0.22406 (13)	0.1730 (3)	0.0125 (7)*	0.407 (13)
Al27	0.000000	0.000000	0.000000	0.0161 (6)	0.853 (7)
Al28A	0.0495 (3)	0.10981 (8)	0.55507 (9)	0.0328 (10)	0.445 (3)
Al28B	0.000000	0.0716 (12)	0.5512 (8)	0.008 (6)*	0.064 (7)
Al28C	0.000000	0.0454 (15)	0.5436 (12)	0.011 (9)*	0.046 (7)

### Hexatriacontaaluminium hexadecaruthenium decasilicon (I) Atomic displacement parameters (Å^2^)

**Table:**

	*U*^11^	*U*^22^	*U*^33^	*U*^12^	*U*^13^	*U*^23^
Ru1	0.0119 (2)	0.00458 (16)	0.00454 (16)	0.000	0.000	0.000
Ru2	0.00655 (14)	0.00520 (11)	0.00395 (12)	0.000	0.000	−0.00041 (8)
Ru3	0.00556 (14)	0.00514 (11)	0.00421 (12)	0.000	0.000	0.00000 (9)
Ru4	0.00580 (13)	0.00635 (11)	0.00525 (12)	0.000	0.000	0.00106 (9)
Ru5	0.00665 (14)	0.00574 (11)	0.00577 (12)	0.000	0.000	−0.00051 (9)
Si6	0.0058 (5)	0.0074 (4)	0.0068 (4)	0.000	0.000	0.0002 (3)
Ru6	0.0058 (5)	0.0074 (4)	0.0068 (4)	0.000	0.000	0.0002 (3)
Ru7A	0.0063 (5)	0.0058 (5)	0.0090 (3)	−0.0001 (3)	0.000	0.000
Ru8	0.0052 (11)	0.00544 (18)	0.00647 (19)	0.0004 (2)	0.000	0.000
Ru9	0.00554 (10)	0.00702 (8)	0.00425 (9)	0.00082 (6)	0.00052 (7)	0.00001 (6)
Si10	0.0060 (9)	0.0114 (8)	0.0092 (8)	0.000	0.000	0.000
Al10	0.0060 (9)	0.0114 (8)	0.0092 (8)	0.000	0.000	0.000
Al11	0.0105 (8)	0.0060 (6)	0.0230 (8)	0.000	0.000	0.000
Al12A	0.0192 (17)	0.0135 (11)	0.0053 (9)	0.000	0.000	0.000
Si13	0.0069 (5)	0.0054 (4)	0.0047 (4)	0.000	0.000	−0.0008 (3)
Al14	0.0073 (6)	0.0053 (6)	0.0080 (6)	0.000	0.000	−0.0001 (3)
Si14	0.0073 (6)	0.0053 (6)	0.0080 (6)	0.000	0.000	−0.0001 (3)
Al15	0.0045 (5)	0.0090 (4)	0.0064 (4)	0.000	0.000	0.0019 (3)
Al16	0.0037 (6)	0.0390 (7)	0.0140 (5)	0.000	0.000	−0.0183 (5)
Al17A	0.0124 (7)	0.0060 (7)	0.0090 (10)	0.000	0.000	0.0011 (6)
Al18	0.0091 (7)	0.0167 (6)	0.0120 (6)	0.0067 (4)	0.000	0.000
Si18	0.0091 (7)	0.0167 (6)	0.0120 (6)	0.0067 (4)	0.000	0.000
Al19	0.0138 (6)	0.0084 (5)	0.0178 (5)	−0.0024 (4)	0.000	0.000
Al20	0.0142 (12)	0.0092 (9)	0.0055 (9)	0.0019 (7)	0.000	0.000
Al21	0.0103 (5)	0.0068 (4)	0.0095 (4)	0.0021 (2)	−0.0039 (3)	−0.0005 (2)
Si21	0.0103 (5)	0.0068 (4)	0.0095 (4)	0.0021 (2)	−0.0039 (3)	−0.0005 (2)
Al22	0.0112 (5)	0.0097 (4)	0.0148 (5)	0.0015 (3)	0.0059 (3)	0.0041 (3)
Si22	0.0112 (5)	0.0097 (4)	0.0148 (5)	0.0015 (3)	0.0059 (3)	0.0041 (3)
Al23	0.0091 (4)	0.0088 (3)	0.0090 (3)	0.0011 (2)	0.0030 (3)	0.0015 (2)
Al24	0.0091 (4)	0.0099 (3)	0.0123 (3)	0.0018 (3)	−0.0013 (3)	0.0042 (3)
Al25	0.0262 (5)	0.0121 (4)	0.0285 (5)	−0.0080 (3)	−0.0213 (4)	0.0051 (3)
Al26A	0.0040 (9)	0.0086 (7)	0.0084 (13)	−0.0013 (5)	−0.0024 (8)	0.0017 (7)
Al27	0.0105 (11)	0.0136 (10)	0.0243 (11)	0.000	0.000	−0.0147 (7)
Al28A	0.069 (3)	0.0227 (11)	0.0063 (8)	0.0029 (9)	0.0017 (9)	−0.0007 (6)

### Hexatriacontaaluminium hexadecaruthenium decasilicon (I) Geometric parameters (Å, º)

**Table:**

Ru1—Si13	2.4228 (9)	Ru9—Si26B	2.690 (3)
Ru1—Si13^i^	2.4228 (9)	Ru9—Al17A^xx^	2.7899 (10)
Ru1—Si18	2.5255 (10)	Si10—Al19^xxi^	2.6487 (13)
Ru1—Al18^ii^	2.5255 (10)	Si10—Al19^xvi^	2.6487 (13)
Ru1—Al19	2.6055 (11)	Si10—Al24^xxi^	2.8248 (7)
Ru1—Al19^ii^	2.6056 (10)	Si10—Al24^xvi^	2.8248 (7)
Ru1—Al20^ii^	2.8218 (19)	Si10—Al24^xvii^	2.8248 (7)
Ru1—Al20	2.8218 (19)	Si10—Al24^xxii^	2.8248 (7)
Ru1—Al25	2.8750 (10)	Al12A—Al12B^ii^	0.74 (3)
Ru1—Al25^i^	2.8750 (10)	Al12A—Al12B	0.74 (3)
Ru1—Al25^iii^	2.8750 (10)	Al12A—Al21^iii^	2.6080 (9)
Ru2—Si26B	2.427 (3)	Al12A—Al21^i^	2.6080 (9)
Ru2—Si26B^iii^	2.427 (3)	Al12A—Al21^ii^	2.6080 (9)
Ru2—Si13	2.4571 (8)	Al12A—Al21	2.6080 (9)
Ru2—Al12B	2.515 (9)	Al12A—Al20	2.724 (3)
Ru2—Al12B^ii^	2.515 (9)	Al12A—Al20^ii^	2.724 (3)
Ru2—Al21^iii^	2.5221 (7)	Al12B—Al12B^ii^	1.20 (5)
Ru2—Al21	2.5222 (7)	Al12B—Al20	2.17 (2)
Ru2—Al26A^iii^	2.524 (2)	Al12B—Al21^i^	2.423 (11)
Ru2—Al26A	2.524 (2)	Al12B—Al21	2.423 (11)
Ru2—Al22	2.6349 (7)	Al12B—Al20^ii^	2.595 (19)
Ru3—Si17B	2.469 (11)	Al12B—Si26B^i^	2.89 (2)
Ru3—Al24^iv^	2.5456 (7)	Al12B—Si26B	2.89 (2)
Ru3—Al24^v^	2.5456 (7)	Si13—Al20^ii^	2.5183 (14)
Ru3—Al17A	2.5668 (13)	Si13—Al20	2.5183 (14)
Ru3—Al28A^iii^	2.6011 (18)	Si13—Al25	2.5897 (10)
Ru3—Al28A	2.6011 (18)	Si13—Al25^iii^	2.5898 (10)
Ru3—Al25^iv^	2.6069 (8)	Si13—Si26B	2.793 (4)
Ru3—Al25^v^	2.6069 (8)	Si13—Si26B^iii^	2.793 (4)
Ru3—Si10^vi^	2.6207 (11)	Al14—Al25	2.6585 (11)
Ru3—Al28B	2.623 (18)	Al14—Al25^iii^	2.6585 (11)
Ru3—Al18^vii^	2.6662 (9)	Al14—Al23^ix^	2.7345 (10)
Ru3—Al18^iv^	2.6662 (9)	Al14—Al23^viii^	2.7345 (10)
Ru4—Al16	2.4671 (11)	Al14—Al15^i^	2.7624 (13)
Ru4—Al23^viii^	2.5271 (7)	Al14—Al22^viii^	2.8756 (9)
Ru4—Al23^ix^	2.5271 (7)	Al14—Al22^ix^	2.8756 (9)
Ru4—Al14	2.5361 (9)	Al15—Si17B	2.538 (12)
Ru4—Al23^x^	2.6278 (7)	Al15—Al17A	2.6774 (16)
Ru4—Al23^xi^	2.6278 (7)	Al15—Al22^ii^	2.8634 (11)
Ru4—Al25^iii^	2.7401 (8)	Al15—Al22^i^	2.8634 (11)
Ru4—Al25	2.7402 (8)	Al15—Al22^v^	2.8818 (9)
Ru4—Al24	2.8446 (8)	Al15—Al22^iv^	2.8818 (9)
Ru4—Al24^iii^	2.8446 (8)	Al16—Al24	2.6216 (8)
Ru5—Al27	2.3207 (2)	Al16—Al24^iii^	2.6216 (8)
Ru5—Al28C^i^	2.35 (2)	Al16—Al21^x^	2.9146 (11)
Ru5—Al28B^i^	2.352 (16)	Al16—Al21^xi^	2.9146 (11)
Ru5—Al28A^ii^	2.5293 (18)	Al17A—Si17B	0.63 (2)
Ru5—Al28A^i^	2.5293 (18)	Al17A—Al28A^iii^	2.581 (3)
Ru5—Al21	2.5429 (7)	Al17A—Al28A	2.581 (3)
Ru5—Al21^iii^	2.5429 (7)	Al17A—Al26A^v^	2.588 (2)
Ru5—Al23^iii^	2.5708 (7)	Al17A—Al26A^iv^	2.588 (2)
Ru5—Al23	2.5708 (7)	Al17A—Si26B^v^	2.661 (3)
Ru5—Al22	2.5988 (7)	Al17A—Si26B^iv^	2.661 (3)
Si6—Al26A^x^	2.583 (5)	Al17A—Al18^vii^	2.7577 (18)
Si6—Al26A^xi^	2.583 (5)	Al17A—Al18^iv^	2.7577 (18)
Si6—Al21^x^	2.5898 (8)	Si17B—Al28A^iii^	1.98 (2)
Si6—Al21^xi^	2.5898 (8)	Si17B—Al28A	1.98 (2)
Si6—Al14^xii^	2.6404 (12)	Si17B—Al28B	2.75 (4)
Si6—Al23^xi^	2.7613 (9)	Si17B—Al25^iv^	2.808 (7)
Si6—Al23^x^	2.7613 (9)	Si17B—Al25^v^	2.808 (7)
Si6—Al15^xiii^	2.7990 (12)	Si18—Al19	2.6026 (14)
Si6—Al22^xi^	2.8073 (9)	Si18—Al25	2.8305 (10)
Si6—Al22^x^	2.8073 (9)	Si18—Al25^i^	2.8305 (9)
Si6—Al16	2.9037 (15)	Si18—Al20	2.837 (2)
Ru7A—Al21	2.460 (3)	Al19—Al25^i^	2.7344 (10)
Ru7A—Al21^i^	2.460 (3)	Al19—Al25	2.7344 (10)
Ru7A—Si10	2.465 (2)	Al19—Al24	2.8599 (9)
Ru7A—Al16^xiv^	2.486 (3)	Al19—Al24^i^	2.8599 (9)
Ru7A—Al16^xv^	2.486 (3)	Al20—Al20^ii^	1.696 (4)
Ru7A—Al11^xv^	2.5774 (16)	Al20—Si26B^i^	2.127 (7)
Ru7A—Al19^xvi^	2.586 (4)	Al20—Si26B	2.127 (7)
Ru7A—Al12B	2.74 (2)	Al20—Al26A	2.616 (6)
Ru7A—Al12A	2.832 (5)	Al20—Al26A^i^	2.616 (6)
Ru7A—Al24^xvii^	2.9086 (11)	Al21—Al26A	2.6828 (18)
Ru7B—Al16^xiv^	2.355 (14)	Al21—Al24^xvii^	2.6980 (10)
Ru7B—Al16^xv^	2.355 (14)	Al21—Si26B	2.822 (3)
Ru7B—Al19^xvi^	2.440 (18)	Al21—Al22	2.8362 (10)
Ru7B—Si10	2.543 (13)	Al22—Al28A^i^	2.637 (2)
Ru7B—Al21	2.583 (15)	Al22—Al26A	2.791 (4)
Ru7B—Al21^i^	2.583 (15)	Al22—Al22^iii^	2.8673 (15)
Ru7B—Al11^xv^	2.604 (11)	Al23—Al24^xvii^	2.6935 (10)
Ru7B—Al24^xvii^	2.890 (6)	Al23—Al27	2.7939 (7)
Ru7B—Al24^xvi^	2.890 (6)	Al23—Al23^xix^	2.8174 (15)
Ru7B—Ru7B^xviii^	2.93 (4)	Al23—Al23^xxiii^	2.8534 (13)
Ru8—Ru8^xviii^	0.384 (4)	Al23—Al28C^i^	2.868 (13)
Ru8—Si26B^i^	2.191 (4)	Al24—Al28C^xx^	2.48 (2)
Ru8—Si26B	2.191 (4)	Al24—Al28B^xx^	2.670 (16)
Ru8—Si18	2.4257 (13)	Al24—Al25	2.7467 (10)
Ru8—Al26A	2.428 (3)	Al24—Al24^xix^	2.7858 (15)
Ru8—Al26A^i^	2.428 (3)	Al25—Al28A^xx^	2.592 (2)
Ru8—Si26B^xviii^	2.455 (3)	Al26A—Si26B	0.503 (2)
Ru8—Si26B^xix^	2.455 (3)	Al26A—Al26A^xix^	2.741 (6)
Ru8—Al11^xv^	2.5478 (14)	Al27—Al28C^i^	1.40 (3)
Ru8—Al18^xviii^	2.6254 (15)	Al27—Al28C^xxiv^	1.40 (3)
Ru8—Al26A^xviii^	2.636 (3)	Al27—Al28B^i^	1.98 (3)
Ru9—Si13	2.4102 (7)	Al27—Al28B^xxiv^	1.98 (3)
Ru9—Al22^ix^	2.5287 (7)	Al27—Al28A^i^	2.8403 (19)
Ru9—Al14	2.5320 (7)	Al27—Al28A^xxiv^	2.8403 (19)
Ru9—Al26A	2.5415 (17)	Al28A—Al28A^iii^	0.754 (5)
Ru9—Al15^xx^	2.5526 (5)	Al28A—Al28B	0.98 (2)
Ru9—Al15^i^	2.6130 (8)	Al28A—Al28C	1.57 (4)
Ru9—Al22	2.6343 (7)	Al28B—Al28C	0.63 (2)
Ru9—Si17B^xx^	2.662 (6)	Al28C—Al28C^vi^	2.79 (7)
			
Si13—Ru1—Si13^i^	109.88 (4)	Ru3—Al17A—Al28A^iii^	60.70 (6)
Si13—Ru1—Si18	85.009 (14)	Si17B—Al17A—Al28A	16.0 (9)
Si13^i^—Ru1—Si18	85.008 (14)	Ru3—Al17A—Al28A	60.69 (6)
Si13—Ru1—Al18^ii^	85.008 (14)	Al28A^iii^—Al17A—Al28A	16.81 (11)
Si13^i^—Ru1—Al18^ii^	85.008 (14)	Si17B—Al17A—Al26A^v^	125.3 (8)
Si18—Ru1—Al18^ii^	162.57 (5)	Ru3—Al17A—Al26A^v^	139.14 (13)
Si13—Ru1—Al19	117.000 (18)	Al28A^iii^—Al17A—Al26A^v^	141.06 (11)
Si13^i^—Ru1—Al19	117.001 (18)	Al28A—Al17A—Al26A^v^	128.54 (8)
Si18—Ru1—Al19	60.93 (3)	Si17B—Al17A—Al26A^iv^	125.3 (8)
Al18^ii^—Ru1—Al19	136.49 (4)	Ru3—Al17A—Al26A^iv^	139.14 (13)
Si13—Ru1—Al19^ii^	117.000 (18)	Al28A^iii^—Al17A—Al26A^iv^	128.53 (8)
Si13^i^—Ru1—Al19^ii^	117.001 (18)	Al28A—Al17A—Al26A^iv^	141.06 (11)
Si18—Ru1—Al19^ii^	136.49 (4)	Al26A^v^—Al17A—Al26A^iv^	63.95 (14)
Al18^ii^—Ru1—Al19^ii^	60.93 (3)	Si17B—Al17A—Si26B^v^	130.6 (7)
Al19—Ru1—Al19^ii^	75.56 (5)	Ru3—Al17A—Si26B^v^	128.58 (15)
Si13—Ru1—Al20^ii^	56.78 (2)	Al28A^iii^—Al17A—Si26B^v^	145.29 (10)
Si13^i^—Ru1—Al20^ii^	56.78 (2)	Al28A—Al17A—Si26B^v^	130.25 (9)
Si18—Ru1—Al20^ii^	98.78 (5)	Al26A^v^—Al17A—Si26B^v^	10.89 (5)
Al18^ii^—Ru1—Al20^ii^	63.79 (5)	Al26A^iv^—Al17A—Si26B^v^	69.85 (14)
Al19—Ru1—Al20^ii^	159.71 (5)	Si17B—Al17A—Si26B^iv^	130.6 (7)
Al19^ii^—Ru1—Al20^ii^	124.73 (5)	Ru3—Al17A—Si26B^iv^	128.58 (15)
Si13—Ru1—Al20	56.78 (2)	Al28A^iii^—Al17A—Si26B^iv^	130.25 (9)
Si13^i^—Ru1—Al20	56.78 (2)	Al28A—Al17A—Si26B^iv^	145.29 (10)
Si18—Ru1—Al20	63.80 (5)	Al26A^v^—Al17A—Si26B^iv^	69.85 (14)
Al18^ii^—Ru1—Al20	98.78 (5)	Al26A^iv^—Al17A—Si26B^iv^	10.89 (5)
Al19—Ru1—Al20	124.73 (5)	Si26B^v^—Al17A—Si26B^iv^	74.02 (16)
Al19^ii^—Ru1—Al20	159.71 (5)	Si17B—Al17A—Al15	70.5 (11)
Al20^ii^—Ru1—Al20	34.98 (9)	Ru3—Al17A—Al15	144.49 (11)
Si13—Ru1—Al25	57.77 (2)	Al28A^iii^—Al17A—Al15	84.21 (8)
Si13^i^—Ru1—Al25	145.35 (2)	Al28A—Al17A—Al15	84.21 (8)
Si18—Ru1—Al25	62.825 (19)	Al26A^v^—Al17A—Al15	66.88 (12)
Al18^ii^—Ru1—Al25	122.209 (17)	Al26A^iv^—Al17A—Al15	66.88 (12)
Al19—Ru1—Al25	59.618 (17)	Si26B^v^—Al17A—Al15	77.42 (14)
Al19^ii^—Ru1—Al25	96.18 (2)	Si26B^iv^—Al17A—Al15	77.42 (14)
Al20^ii^—Ru1—Al25	113.00 (3)	Si17B—Al17A—Al18^vii^	123.8 (9)
Al20—Ru1—Al25	95.15 (3)	Ru3—Al17A—Al18^vii^	59.97 (3)
Si13—Ru1—Al25^i^	145.35 (2)	Al28A^iii^—Al17A—Al18^vii^	116.49 (7)
Si13^i^—Ru1—Al25^i^	57.78 (2)	Al28A—Al17A—Al18^vii^	107.86 (6)
Si18—Ru1—Al25^i^	62.825 (19)	Al26A^v^—Al17A—Al18^vii^	81.29 (14)
Al18^ii^—Ru1—Al25^i^	122.210 (17)	Al26A^iv^—Al17A—Al18^vii^	110.69 (12)
Al19—Ru1—Al25^i^	59.616 (17)	Si26B^v^—Al17A—Al18^vii^	70.40 (15)
Al19^ii^—Ru1—Al25^i^	96.18 (2)	Si26B^iv^—Al17A—Al18^vii^	103.80 (13)
Al20^ii^—Ru1—Al25^i^	113.00 (3)	Al15—Al17A—Al18^vii^	145.81 (5)
Al20—Ru1—Al25^i^	95.15 (3)	Si17B—Al17A—Al18^iv^	123.8 (9)
Al25—Ru1—Al25^i^	111.83 (3)	Ru3—Al17A—Al18^iv^	59.97 (3)
Si13—Ru1—Al25^iii^	57.77 (2)	Al28A^iii^—Al17A—Al18^iv^	107.86 (6)
Si13^i^—Ru1—Al25^iii^	145.35 (2)	Al28A—Al17A—Al18^iv^	116.49 (7)
Si18—Ru1—Al25^iii^	122.210 (17)	Al26A^v^—Al17A—Al18^iv^	110.69 (12)
Al18^ii^—Ru1—Al25^iii^	62.825 (19)	Al26A^iv^—Al17A—Al18^iv^	81.29 (14)
Al19—Ru1—Al25^iii^	96.18 (2)	Si26B^v^—Al17A—Al18^iv^	103.80 (13)
Al19^ii^—Ru1—Al25^iii^	59.616 (17)	Si26B^iv^—Al17A—Al18^iv^	70.40 (15)
Al20^ii^—Ru1—Al25^iii^	95.15 (3)	Al15—Al17A—Al18^iv^	145.81 (5)
Al20—Ru1—Al25^iii^	113.00 (3)	Al18^vii^—Al17A—Al18^iv^	56.93 (5)
Al25—Ru1—Al25^iii^	60.08 (3)	Si17B—Al17A—Ru8^vii^	175.5 (5)
Al25^i^—Ru1—Al25^iii^	150.82 (3)	Ru3—Al17A—Ru8^vii^	103.83 (6)
Si26B—Ru2—Si26B^iii^	131.1 (3)	Al28A^iii^—Al17A—Ru8^vii^	163.69 (7)
Si26B—Ru2—Si13	69.75 (9)	Al28A—Al17A—Ru8^vii^	159.96 (7)
Si26B^iii^—Ru2—Si13	69.75 (9)	Al26A^v^—Al17A—Ru8^vii^	53.88 (9)
Si26B—Ru2—Al12B	71.4 (7)	Al26A^iv^—Al17A—Ru8^vii^	58.94 (9)
Si26B^iii^—Ru2—Al12B	96.7 (5)	Si26B^v^—Al17A—Ru8^vii^	47.63 (10)
Si13—Ru2—Al12B	108.2 (5)	Si26B^iv^—Al17A—Ru8^vii^	53.82 (9)
Si26B—Ru2—Al12B^ii^	96.7 (5)	Al15—Al17A—Ru8^vii^	111.59 (6)
Si26B^iii^—Ru2—Al12B^ii^	71.4 (7)	Al18^vii^—Al17A—Ru8^vii^	52.15 (5)
Si13—Ru2—Al12B^ii^	108.2 (5)	Al18^iv^—Al17A—Ru8^vii^	56.82 (6)
Al12B—Ru2—Al12B^ii^	27.6 (12)	Al17A—Si17B—Al28A^iii^	159.0 (12)
Si26B—Ru2—Al21^iii^	143.17 (6)	Al17A—Si17B—Al28A	159.0 (12)
Si26B^iii^—Ru2—Al21^iii^	69.49 (9)	Al28A^iii^—Si17B—Al28A	21.9 (3)
Si13—Ru2—Al21^iii^	139.236 (17)	Al17A—Si17B—Ru3	91.8 (12)
Al12B—Ru2—Al21^iii^	76.4 (6)	Al28A^iii^—Si17B—Ru3	70.5 (5)
Al12B^ii^—Ru2—Al21^iii^	57.5 (4)	Al28A—Si17B—Ru3	70.5 (5)
Si26B—Ru2—Al21	69.49 (9)	Al17A—Si17B—Al15	96.0 (12)
Si26B^iii^—Ru2—Al21	143.17 (6)	Al28A^iii^—Si17B—Al15	101.8 (8)
Si13—Ru2—Al21	139.237 (17)	Al28A—Si17B—Al15	101.8 (8)
Al12B—Ru2—Al21	57.5 (4)	Ru3—Si17B—Al15	172.2 (12)
Al12B^ii^—Ru2—Al21	76.4 (6)	Al17A—Si17B—Ru9^iv^	95.2 (8)
Al21^iii^—Ru2—Al21	78.42 (3)	Al28A^iii^—Si17B—Ru9^iv^	84.8 (5)
Si26B—Ru2—Al26A^iii^	141.3 (2)	Al28A—Si17B—Ru9^iv^	103.5 (8)
Si26B^iii^—Ru2—Al26A^iii^	11.46 (6)	Ru3—Si17B—Ru9^iv^	120.6 (2)
Si13—Ru2—Al26A^iii^	75.71 (7)	Al15—Si17B—Ru9^iv^	58.7 (2)
Al12B—Ru2—Al26A^iii^	104.4 (5)	Al17A—Si17B—Ru9^v^	95.2 (8)
Al12B^ii^—Ru2—Al26A^iii^	77.8 (6)	Al28A^iii^—Si17B—Ru9^v^	103.5 (8)
Al21^iii^—Ru2—Al26A^iii^	64.24 (6)	Al28A—Si17B—Ru9^v^	84.8 (5)
Al21—Ru2—Al26A^iii^	141.99 (5)	Ru3—Si17B—Ru9^v^	120.6 (2)
Si26B—Ru2—Al26A	11.46 (6)	Al15—Si17B—Ru9^v^	58.7 (2)
Si26B^iii^—Ru2—Al26A	141.3 (2)	Ru9^iv^—Si17B—Ru9^v^	117.3 (4)
Si13—Ru2—Al26A	75.70 (7)	Al17A—Si17B—Al28B	151.9 (13)
Al12B—Ru2—Al26A	77.8 (6)	Al28A^iii^—Si17B—Al28B	14.9 (3)
Al12B^ii^—Ru2—Al26A	104.4 (5)	Al28A—Si17B—Al28B	14.9 (3)
Al21^iii^—Ru2—Al26A	141.99 (4)	Ru3—Si17B—Al28B	60.1 (5)
Al21—Ru2—Al26A	64.24 (6)	Al15—Si17B—Al28B	112.1 (9)
Al26A^iii^—Ru2—Al26A	150.45 (19)	Ru9^iv^—Si17B—Al28B	99.3 (7)
Si26B—Ru2—Al22	76.76 (16)	Ru9^v^—Si17B—Al28B	99.3 (7)
Si26B^iii^—Ru2—Al22	139.60 (14)	Al17A—Si17B—Al25^iv^	98.7 (8)
Si13—Ru2—Al22	103.99 (2)	Al28A^iii^—Si17B—Al25^iv^	62.7 (3)
Al12B—Ru2—Al22	122.3 (4)	Al28A—Si17B—Al25^iv^	82.1 (6)
Al12B^ii^—Ru2—Al22	142.5 (6)	Ru3—Si17B—Al25^iv^	58.8 (2)
Al21^iii^—Ru2—Al22	106.99 (2)	Al15—Si17B—Al25^iv^	119.7 (3)
Al21—Ru2—Al22	66.69 (2)	Ru9^iv^—Si17B—Al25^iv^	61.84 (8)
Al26A^iii^—Ru2—Al22	129.56 (11)	Ru9^v^—Si17B—Al25^iv^	166.1 (11)
Al26A—Ru2—Al22	65.46 (12)	Al28B—Si17B—Al25^iv^	67.9 (4)
Si17B—Ru3—Al24^iv^	115.9 (5)	Al17A—Si17B—Al25^v^	98.7 (8)
Si17B—Ru3—Al24^v^	115.9 (5)	Al28A^iii^—Si17B—Al25^v^	82.1 (6)
Al24^iv^—Ru3—Al24^v^	66.35 (3)	Al28A—Si17B—Al25^v^	62.7 (3)
Si17B—Ru3—Al17A	14.2 (6)	Ru3—Si17B—Al25^v^	58.8 (2)
Al24^iv^—Ru3—Al17A	126.73 (5)	Al15—Si17B—Al25^v^	119.7 (3)
Al24^v^—Ru3—Al17A	126.73 (5)	Ru9^iv^—Si17B—Al25^v^	166.1 (11)
Si17B—Ru3—Al28A^iii^	46.0 (6)	Ru9^v^—Si17B—Al25^v^	61.84 (8)
Al24^iv^—Ru3—Al28A^iii^	73.78 (5)	Al28B—Si17B—Al25^v^	67.9 (4)
Al24^v^—Ru3—Al28A^iii^	83.07 (5)	Al25^iv^—Si17B—Al25^v^	115.3 (5)
Al17A—Ru3—Al28A^iii^	59.93 (7)	Ru8—Si18—Ru1	126.28 (6)
Si17B—Ru3—Al28A	46.0 (6)	Ru8—Si18—Al19	172.67 (7)
Al24^iv^—Ru3—Al28A	83.07 (5)	Ru1—Si18—Al19	61.05 (3)
Al24^v^—Ru3—Al28A	73.78 (5)	Ru8—Si18—Ru8^xviii^	7.45 (7)
Al17A—Ru3—Al28A	59.93 (7)	Ru1—Si18—Ru8^xviii^	133.73 (6)
Al28A^iii^—Ru3—Al28A	16.68 (11)	Al19—Si18—Ru8^xviii^	165.22 (6)
Si17B—Ru3—Al25^iv^	67.11 (10)	Ru8—Si18—Al18^xviii^	62.43 (5)
Al24^iv^—Ru3—Al25^iv^	64.41 (2)	Ru1—Si18—Al18^xviii^	171.29 (2)
Al24^v^—Ru3—Al25^iv^	124.31 (3)	Al19—Si18—Al18^xviii^	110.24 (3)
Al17A—Ru3—Al25^iv^	70.03 (2)	Ru8^xviii^—Si18—Al18^xviii^	54.99 (4)
Al28A^iii^—Ru3—Al25^iv^	59.69 (6)	Ru8—Si18—Ru3^vii^	110.84 (5)
Al28A—Ru3—Al25^iv^	76.07 (6)	Ru1—Si18—Ru3^vii^	112.80 (3)
Si17B—Ru3—Al25^v^	67.11 (10)	Al19—Si18—Ru3^vii^	63.41 (3)
Al24^iv^—Ru3—Al25^v^	124.31 (3)	Ru8^xviii^—Si18—Ru3^vii^	104.87 (4)
Al24^v^—Ru3—Al25^v^	64.41 (2)	Al18^xviii^—Si18—Ru3^vii^	60.46 (2)
Al17A—Ru3—Al25^v^	70.03 (2)	Ru8—Si18—Ru3^xx^	110.84 (5)
Al28A^iii^—Ru3—Al25^v^	76.07 (6)	Ru1—Si18—Ru3^xx^	112.80 (3)
Al28A—Ru3—Al25^v^	59.69 (6)	Al19—Si18—Ru3^xx^	63.41 (3)
Al25^iv^—Ru3—Al25^v^	130.93 (4)	Ru8^xviii^—Si18—Ru3^xx^	104.87 (4)
Si17B—Ru3—Si10^vi^	177.3 (6)	Al18^xviii^—Si18—Ru3^xx^	60.46 (2)
Al24^iv^—Ru3—Si10^vi^	66.27 (2)	Ru3^vii^—Si18—Ru3^xx^	69.26 (3)
Al24^v^—Ru3—Si10^vi^	66.27 (2)	Ru8—Si18—Al17A^vii^	63.98 (5)
Al17A—Ru3—Si10^vi^	163.13 (6)	Ru1—Si18—Al17A^vii^	120.56 (3)
Al28A^iii^—Ru3—Si10^vi^	136.65 (5)	Al19—Si18—Al17A^vii^	113.31 (5)
Al28A—Ru3—Si10^vi^	136.64 (5)	Ru8^xviii^—Si18—Al17A^vii^	61.64 (5)
Al25^iv^—Ru3—Si10^vi^	113.29 (2)	Al18^xviii^—Si18—Al17A^vii^	61.53 (3)
Al25^v^—Ru3—Si10^vi^	113.29 (2)	Ru3^vii^—Si18—Al17A^vii^	56.46 (4)
Si17B—Ru3—Al28B	65.3 (8)	Ru3^xx^—Si18—Al17A^vii^	114.00 (4)
Al24^iv^—Ru3—Al28B	62.2 (5)	Ru8—Si18—Al17A^xx^	63.98 (5)
Al24^v^—Ru3—Al28B	62.2 (5)	Ru1—Si18—Al17A^xx^	120.56 (3)
Al17A—Ru3—Al28B	79.5 (6)	Al19—Si18—Al17A^xx^	113.31 (5)
Al28A^iii^—Ru3—Al28B	21.5 (5)	Ru8^xviii^—Si18—Al17A^xx^	61.64 (5)
Al28A—Ru3—Al28B	21.5 (5)	Al18^xviii^—Si18—Al17A^xx^	61.53 (3)
Al25^iv^—Ru3—Al28B	72.86 (18)	Ru3^vii^—Si18—Al17A^xx^	114.00 (4)
Al25^v^—Ru3—Al28B	72.86 (18)	Ru3^xx^—Si18—Al17A^xx^	56.46 (4)
Si10^vi^—Ru3—Al28B	117.4 (6)	Al17A^vii^—Si18—Al17A^xx^	115.12 (8)
Si17B—Ru3—Al18^vii^	75.6 (5)	Ru8—Si18—Al25	121.29 (3)
Al24^iv^—Ru3—Al18^vii^	166.84 (2)	Ru1—Si18—Al25	64.64 (3)
Al24^v^—Ru3—Al18^vii^	115.73 (3)	Al19—Si18—Al25	60.26 (3)
Al17A—Ru3—Al18^vii^	63.57 (5)	Ru8^xviii^—Si18—Al25	122.31 (3)
Al28A^iii^—Ru3—Al18^vii^	119.09 (5)	Al18^xviii^—Si18—Al25	111.93 (3)
Al28A—Ru3—Al18^vii^	110.08 (5)	Ru3^vii^—Si18—Al25	113.87 (4)
Al25^iv^—Ru3—Al18^vii^	118.23 (3)	Ru3^xx^—Si18—Al25	56.53 (2)
Al25^v^—Ru3—Al18^vii^	64.92 (3)	Al17A^vii^—Si18—Al25	169.79 (5)
Si10^vi^—Ru3—Al18^vii^	102.06 (2)	Al17A^xx^—Si18—Al25	64.16 (5)
Al28B—Ru3—Al18^vii^	130.8 (4)	Ru8—Si18—Al25^i^	121.29 (3)
Si17B—Ru3—Al18^iv^	75.6 (5)	Ru1—Si18—Al25^i^	64.64 (3)
Al24^iv^—Ru3—Al18^iv^	115.73 (3)	Al19—Si18—Al25^i^	60.26 (3)
Al24^v^—Ru3—Al18^iv^	166.84 (2)	Ru8^xviii^—Si18—Al25^i^	122.31 (3)
Al17A—Ru3—Al18^iv^	63.57 (5)	Al18^xviii^—Si18—Al25^i^	111.93 (3)
Al28A^iii^—Ru3—Al18^iv^	110.07 (5)	Ru3^vii^—Si18—Al25^i^	56.53 (2)
Al28A—Ru3—Al18^iv^	119.09 (5)	Ru3^xx^—Si18—Al25^i^	113.86 (4)
Al25^iv^—Ru3—Al18^iv^	64.92 (3)	Al17A^vii^—Si18—Al25^i^	64.17 (5)
Al25^v^—Ru3—Al18^iv^	118.23 (3)	Al17A^xx^—Si18—Al25^i^	169.79 (5)
Si10^vi^—Ru3—Al18^iv^	102.06 (2)	Al25—Si18—Al25^i^	114.54 (5)
Al28B—Ru3—Al18^iv^	130.8 (4)	Ru8—Si18—Al20	63.09 (6)
Al18^vii^—Ru3—Al18^iv^	59.08 (4)	Ru1—Si18—Al20	63.19 (5)
Al16—Ru4—Al23^viii^	139.23 (2)	Al19—Si18—Al20	124.24 (6)
Al16—Ru4—Al23^ix^	139.23 (2)	Ru8^xviii^—Si18—Al20	70.54 (6)
Al23^viii^—Ru4—Al23^ix^	67.76 (3)	Al18^xviii^—Si18—Al20	125.52 (5)
Al16—Ru4—Al14	144.31 (4)	Ru3^vii^—Si18—Al20	145.349 (14)
Al23^viii^—Ru4—Al14	65.38 (2)	Ru3^xx^—Si18—Al20	145.349 (14)
Al23^ix^—Ru4—Al14	65.38 (2)	Al17A^vii^—Si18—Al20	94.40 (4)
Al16—Ru4—Al23^x^	75.80 (3)	Al17A^xx^—Si18—Al20	94.40 (4)
Al23^viii^—Ru4—Al23^x^	102.12 (2)	Al25—Si18—Al20	95.81 (4)
Al23^ix^—Ru4—Al23^x^	67.19 (3)	Al25^i^—Si18—Al20	95.81 (4)
Al14—Ru4—Al23^x^	132.11 (2)	Ru7B^xxv^—Al19—Ru7A^xxv^	3.6 (4)
Al16—Ru4—Al23^xi^	75.80 (3)	Ru7B^xxv^—Al19—Si18	162.8 (5)
Al23^viii^—Ru4—Al23^xi^	67.19 (3)	Ru7A^xxv^—Al19—Si18	159.20 (10)
Al23^ix^—Ru4—Al23^xi^	102.12 (2)	Ru7B^xxv^—Al19—Ru1	139.2 (5)
Al14—Ru4—Al23^xi^	132.11 (2)	Ru7A^xxv^—Al19—Ru1	142.79 (9)
Al23^x^—Ru4—Al23^xi^	64.83 (3)	Si18—Al19—Ru1	58.02 (3)
Al16—Ru4—Al25^iii^	89.86 (3)	Ru7B^xxv^—Al19—Si10^xxvii^	59.8 (5)
Al23^viii^—Ru4—Al25^iii^	89.91 (3)	Ru7A^xxv^—Al19—Si10^xxvii^	56.17 (9)
Al23^ix^—Ru4—Al25^iii^	125.74 (2)	Si18—Al19—Si10^xxvii^	103.03 (5)
Al14—Ru4—Al25^iii^	60.36 (2)	Ru1—Al19—Si10^xxvii^	161.04 (5)
Al23^x^—Ru4—Al25^iii^	165.54 (3)	Ru7B^xxv^—Al19—Al25^i^	118.98 (5)
Al23^xi^—Ru4—Al25^iii^	113.90 (2)	Ru7A^xxv^—Al19—Al25^i^	119.26 (2)
Al16—Ru4—Al25	89.86 (3)	Si18—Al19—Al25^i^	64.00 (3)
Al23^viii^—Ru4—Al25	125.74 (2)	Ru1—Al19—Al25^i^	65.10 (3)
Al23^ix^—Ru4—Al25	89.91 (3)	Si10^xxvii^—Al19—Al25^i^	108.41 (3)
Al14—Ru4—Al25	60.36 (2)	Ru7B^xxv^—Al19—Al25	118.98 (5)
Al23^x^—Ru4—Al25	113.90 (2)	Ru7A^xxv^—Al19—Al25	119.26 (2)
Al23^xi^—Ru4—Al25	165.54 (3)	Si18—Al19—Al25	64.00 (3)
Al25^iii^—Ru4—Al25	63.37 (4)	Ru1—Al19—Al25	65.10 (3)
Al16—Ru4—Al24	58.633 (16)	Si10^xxvii^—Al19—Al25	108.41 (3)
Al23^viii^—Ru4—Al24	153.59 (2)	Al25^i^—Al19—Al25	121.10 (5)
Al23^ix^—Ru4—Al24	87.02 (2)	Ru7B^xxv^—Al19—Ru3^vii^	106.9 (4)
Al14—Ru4—Al24	112.050 (17)	Ru7A^xxv^—Al19—Ru3^vii^	103.87 (7)
Al23^x^—Ru4—Al24	58.81 (2)	Si18—Al19—Ru3^vii^	59.41 (3)
Al23^xi^—Ru4—Al24	113.17 (2)	Ru1—Al19—Ru3^vii^	107.11 (3)
Al25^iii^—Ru4—Al24	112.10 (3)	Si10^xxvii^—Al19—Ru3^vii^	57.80 (3)
Al25—Ru4—Al24	58.89 (2)	Al25^i^—Al19—Ru3^vii^	56.54 (2)
Al16—Ru4—Al24^iii^	58.634 (16)	Al25—Al19—Ru3^vii^	113.66 (4)
Al23^viii^—Ru4—Al24^iii^	87.02 (2)	Ru7B^xxv^—Al19—Ru3^xx^	106.9 (4)
Al23^ix^—Ru4—Al24^iii^	153.59 (2)	Ru7A^xxv^—Al19—Ru3^xx^	103.87 (7)
Al14—Ru4—Al24^iii^	112.049 (17)	Si18—Al19—Ru3^xx^	59.41 (3)
Al23^x^—Ru4—Al24^iii^	113.17 (2)	Ru1—Al19—Ru3^xx^	107.11 (3)
Al23^xi^—Ru4—Al24^iii^	58.81 (2)	Si10^xxvii^—Al19—Ru3^xx^	57.80 (3)
Al25^iii^—Ru4—Al24^iii^	58.88 (2)	Al25^i^—Al19—Ru3^xx^	113.66 (4)
Al25—Ru4—Al24^iii^	112.10 (3)	Al25—Al19—Ru3^xx^	56.54 (2)
Al24—Ru4—Al24^iii^	116.42 (3)	Ru3^vii^—Al19—Ru3^xx^	66.33 (3)
Al27—Ru5—Al28C^i^	34.8 (9)	Ru7B^xxv^—Al19—Al24	65.53 (15)
Al27—Ru5—Al28B^i^	50.3 (7)	Ru7A^xxv^—Al19—Al24	64.34 (3)
Al28C^i^—Ru5—Al28B^i^	15.5 (6)	Si18—Al19—Al24	107.72 (3)
Al27—Ru5—Al28A^ii^	71.55 (4)	Ru1—Al19—Al24	121.07 (2)
Al28C^i^—Ru5—Al28A^ii^	37.4 (9)	Si10^xxvii^—Al19—Al24	61.56 (2)
Al28B^i^—Ru5—Al28A^ii^	22.7 (6)	Al25^i^—Al19—Al24	166.35 (5)
Al27—Ru5—Al28A^i^	71.55 (4)	Al25—Al19—Al24	58.76 (2)
Al28C^i^—Ru5—Al28A^i^	37.4 (9)	Ru3^vii^—Al19—Al24	110.17 (4)
Al28B^i^—Ru5—Al28A^i^	22.7 (6)	Ru3^xx^—Al19—Al24	53.74 (2)
Al28A^ii^—Ru5—Al28A^i^	17.15 (11)	Ru7B^xxv^—Al19—Al24^i^	65.53 (15)
Al27—Ru5—Al21	127.171 (16)	Ru7A^xxv^—Al19—Al24^i^	64.34 (3)
Al28C^i^—Ru5—Al21	140.96 (9)	Si18—Al19—Al24^i^	107.71 (3)
Al28B^i^—Ru5—Al21	139.85 (16)	Ru1—Al19—Al24^i^	121.07 (2)
Al28A^ii^—Ru5—Al21	138.19 (5)	Si10^xxvii^—Al19—Al24^i^	61.56 (2)
Al28A^i^—Ru5—Al21	123.94 (6)	Al25^i^—Al19—Al24^i^	58.76 (2)
Al27—Ru5—Al21^iii^	127.172 (16)	Al25—Al19—Al24^i^	166.35 (5)
Al28C^i^—Ru5—Al21^iii^	140.96 (9)	Ru3^vii^—Al19—Al24^i^	53.74 (2)
Al28B^i^—Ru5—Al21^iii^	139.85 (16)	Ru3^xx^—Al19—Al24^i^	110.17 (4)
Al28A^ii^—Ru5—Al21^iii^	123.94 (6)	Al24—Al19—Al24^i^	117.69 (4)
Al28A^i^—Ru5—Al21^iii^	138.19 (5)	Al20^ii^—Al20—Si26B^i^	129.78 (9)
Al21—Ru5—Al21^iii^	77.66 (3)	Al20^ii^—Al20—Si26B	129.78 (9)
Al27—Ru5—Al23^iii^	69.449 (16)	Si26B^i^—Al20—Si26B	97.04 (16)
Al28C^i^—Ru5—Al23^iii^	71.17 (14)	Al20^ii^—Al20—Al12B	83.4 (7)
Al28B^i^—Ru5—Al23^iii^	74.28 (16)	Si26B^i^—Al20—Al12B	84.5 (4)
Al28A^ii^—Ru5—Al23^iii^	72.12 (6)	Si26B—Al20—Al12B	84.5 (4)
Al28A^i^—Ru5—Al23^iii^	88.37 (6)	Al20^ii^—Al20—Si13^i^	70.32 (5)
Al21—Ru5—Al23^iii^	145.86 (2)	Si26B^i^—Al20—Si13^i^	73.37 (7)
Al21^iii^—Ru5—Al23^iii^	69.88 (2)	Si26B—Al20—Si13^i^	153.36 (12)
Al27—Ru5—Al23	69.449 (16)	Al12B—Al20—Si13^i^	118.3 (3)
Al28C^i^—Ru5—Al23	71.17 (14)	Al20^ii^—Al20—Si13	70.32 (5)
Al28B^i^—Ru5—Al23	74.28 (16)	Si26B^i^—Al20—Si13	153.36 (12)
Al28A^ii^—Ru5—Al23	88.38 (6)	Si26B—Al20—Si13	73.37 (7)
Al28A^i^—Ru5—Al23	72.12 (6)	Al12B—Al20—Si13	118.3 (3)
Al21—Ru5—Al23	69.88 (2)	Si13^i^—Al20—Si13	103.91 (8)
Al21^iii^—Ru5—Al23	145.86 (2)	Al20^ii^—Al20—Al12B^ii^	56.1 (6)
Al23^iii^—Ru5—Al23	138.27 (3)	Si26B^i^—Al20—Al12B^ii^	102.4 (4)
Al27—Ru5—Al22	124.802 (18)	Si26B—Al20—Al12B^ii^	102.4 (4)
Al28C^i^—Ru5—Al22	97.0 (7)	Al12B—Al20—Al12B^ii^	27.3 (13)
Al28B^i^—Ru5—Al22	84.1 (6)	Si13^i^—Al20—Al12B^ii^	103.9 (4)
Al28A^ii^—Ru5—Al22	72.13 (5)	Si13—Al20—Al12B^ii^	103.9 (4)
Al28A^i^—Ru5—Al22	61.87 (5)	Al20^ii^—Al20—Al26A	127.49 (5)
Al21—Ru5—Al22	66.94 (2)	Si26B^i^—Al20—Al26A	98.57 (10)
Al21^iii^—Ru5—Al22	107.46 (2)	Si26B—Al20—Al26A	2.89 (9)
Al23^iii^—Ru5—Al22	132.77 (2)	Al12B—Al20—Al26A	82.2 (4)
Al23—Ru5—Al22	69.40 (2)	Si13^i^—Al20—Al26A	156.21 (10)
Al26A^x^—Si6—Al26A^xi^	64.09 (6)	Si13—Al20—Al26A	73.05 (3)
Al26A^x^—Si6—Al21^x^	62.48 (5)	Al12B^ii^—Al20—Al26A	99.7 (4)
Al26A^xi^—Si6—Al21^x^	116.49 (5)	Al20^ii^—Al20—Al26A^i^	127.49 (5)
Al26A^x^—Si6—Al21^xi^	116.49 (5)	Si26B^i^—Al20—Al26A^i^	2.89 (9)
Al26A^xi^—Si6—Al21^xi^	62.48 (5)	Si26B—Al20—Al26A^i^	98.58 (10)
Al21^x^—Si6—Al21^xi^	117.71 (4)	Al12B—Al20—Al26A^i^	82.2 (4)
Al26A^x^—Si6—Al14^xii^	116.06 (7)	Si13^i^—Al20—Al26A^i^	73.05 (3)
Al26A^xi^—Si6—Al14^xii^	116.06 (7)	Si13—Al20—Al26A^i^	156.21 (10)
Al21^x^—Si6—Al14^xii^	116.56 (2)	Al12B^ii^—Al20—Al26A^i^	99.7 (4)
Al21^xi^—Si6—Al14^xii^	116.56 (2)	Al26A—Al20—Al26A^i^	100.00 (10)
Al26A^x^—Si6—Al23^xi^	176.81 (7)	Al20^ii^—Al20—Ru2	71.28 (5)
Al26A^xi^—Si6—Al23^xi^	117.20 (3)	Si26B^i^—Al20—Ru2	139.65 (11)
Al21^x^—Si6—Al23^xi^	118.09 (3)	Si26B—Al20—Ru2	60.02 (8)
Al21^xi^—Si6—Al23^xi^	66.27 (2)	Al12B—Al20—Ru2	62.1 (2)
Al14^xii^—Si6—Al23^xi^	60.78 (3)	Si13^i^—Al20—Ru2	141.15 (9)
Al26A^x^—Si6—Al23^x^	117.20 (3)	Si13—Al20—Ru2	56.80 (3)
Al26A^xi^—Si6—Al23^x^	176.81 (7)	Al12B^ii^—Al20—Ru2	57.39 (4)
Al21^x^—Si6—Al23^x^	66.27 (2)	Al26A—Al20—Ru2	57.36 (4)
Al21^xi^—Si6—Al23^x^	118.09 (3)	Al26A^i^—Al20—Ru2	138.65 (8)
Al14^xii^—Si6—Al23^x^	60.78 (3)	Al20^ii^—Al20—Ru2^i^	71.28 (5)
Al23^xi^—Si6—Al23^x^	61.35 (3)	Si26B^i^—Al20—Ru2^i^	60.03 (8)
Al26A^x^—Si6—Al15^xiii^	65.13 (7)	Si26B—Al20—Ru2^i^	139.65 (11)
Al26A^xi^—Si6—Al15^xiii^	65.13 (7)	Al12B—Al20—Ru2^i^	62.1 (2)
Al21^x^—Si6—Al15^xiii^	116.38 (2)	Si13^i^—Al20—Ru2^i^	56.80 (3)
Al21^xi^—Si6—Al15^xiii^	116.38 (2)	Si13—Al20—Ru2^i^	141.15 (9)
Al14^xii^—Si6—Al15^xiii^	60.96 (3)	Al12B^ii^—Al20—Ru2^i^	57.39 (4)
Al23^xi^—Si6—Al15^xiii^	112.46 (3)	Al26A—Al20—Ru2^i^	138.65 (8)
Al23^x^—Si6—Al15^xiii^	112.46 (3)	Al26A^i^—Al20—Ru2^i^	57.36 (4)
Al26A^x^—Si6—Al22^xi^	115.60 (5)	Ru2—Al20—Ru2^i^	114.71 (7)
Al26A^xi^—Si6—Al22^xi^	62.20 (5)	Al20^ii^—Al20—Al12A	71.86 (5)
Al21^x^—Si6—Al22^xi^	178.04 (4)	Si26B^i^—Al20—Al12A	92.10 (9)
Al21^xi^—Si6—Al22^xi^	63.25 (2)	Si26B—Al20—Al12A	92.10 (9)
Al14^xii^—Si6—Al22^xi^	63.63 (2)	Al12B—Al20—Al12A	11.6 (7)
Al23^xi^—Si6—Al22^xi^	63.80 (2)	Si13^i^—Al20—Al12A	112.68 (6)
Al23^x^—Si6—Al22^xi^	114.99 (3)	Si13—Al20—Al12A	112.68 (6)
Al15^xiii^—Si6—Al22^xi^	61.86 (2)	Al12B^ii^—Al20—Al12A	15.8 (6)
Al26A^x^—Si6—Al22^x^	62.20 (5)	Al26A—Al20—Al12A	89.59 (6)
Al26A^xi^—Si6—Al22^x^	115.60 (5)	Al26A^i^—Al20—Al12A	89.59 (6)
Al21^x^—Si6—Al22^x^	63.25 (2)	Ru2—Al20—Al12A	59.21 (4)
Al21^xi^—Si6—Al22^x^	178.04 (4)	Ru2^i^—Al20—Al12A	59.21 (4)
Al14^xii^—Si6—Al22^x^	63.63 (2)	Al12B—Al21—Ru7A	68.3 (4)
Al23^xi^—Si6—Al22^x^	114.99 (3)	Al12B—Al21—Ru2	61.1 (4)
Al23^x^—Si6—Al22^x^	63.80 (2)	Ru7A—Al21—Ru2	129.12 (10)
Al15^xiii^—Si6—Al22^x^	61.86 (2)	Al12B—Al21—Ru5	116.5 (6)
Al22^xi^—Si6—Al22^x^	115.73 (4)	Ru7A—Al21—Ru5	129.33 (4)
Al26A^x^—Si6—Al16	115.32 (7)	Ru2—Al21—Ru5	73.60 (2)
Al26A^xi^—Si6—Al16	115.32 (7)	Al12B—Al21—Ru7B	72.2 (6)
Al21^x^—Si6—Al16	63.79 (2)	Ru7A—Al21—Ru7B	4.0 (4)
Al21^xi^—Si6—Al16	63.79 (2)	Ru2—Al21—Ru7B	133.1 (4)
Al14^xii^—Si6—Al16	118.49 (4)	Ru5—Al21—Ru7B	128.9 (2)
Al23^xi^—Si6—Al16	67.08 (3)	Al12B—Al21—Si6^xv^	128.1 (7)
Al23^x^—Si6—Al16	67.08 (3)	Ru7A—Al21—Si6^xv^	107.13 (8)
Al15^xiii^—Si6—Al16	179.45 (4)	Ru2—Al21—Si6^xv^	107.38 (3)
Al22^xi^—Si6—Al16	117.98 (2)	Ru5—Al21—Si6^xv^	105.51 (3)
Al22^x^—Si6—Al16	117.98 (2)	Ru7B—Al21—Si6^xv^	104.1 (4)
Al21—Ru7A—Al21^i^	109.01 (17)	Al12B—Al21—Al12A	16.4 (7)
Al21—Ru7A—Si10	91.59 (11)	Ru7A—Al21—Al12A	67.88 (10)
Al21^i^—Ru7A—Si10	91.59 (11)	Ru2—Al21—Al12A	62.23 (4)
Al21—Ru7A—Al16^xiv^	150.26 (7)	Ru5—Al21—Al12A	103.42 (3)
Al21^i^—Ru7A—Al16^xiv^	72.21 (3)	Ru7B—Al21—Al12A	71.9 (4)
Si10—Ru7A—Al16^xiv^	118.15 (7)	Si6^xv^—Al21—Al12A	144.53 (5)
Al21—Ru7A—Al16^xv^	72.21 (3)	Al12B—Al21—Al26A	76.4 (6)
Al21^i^—Ru7A—Al16^xv^	150.26 (7)	Ru7A—Al21—Al26A	115.15 (8)
Si10—Ru7A—Al16^xv^	118.15 (7)	Ru2—Al21—Al26A	57.91 (6)
Al16^xiv^—Ru7A—Al16^xv^	92.25 (16)	Ru5—Al21—Al26A	114.75 (5)
Al21—Ru7A—Al11^xv^	75.04 (6)	Ru7B—Al21—Al26A	116.1 (2)
Al21^i^—Ru7A—Al11^xv^	75.04 (6)	Si6^xv^—Al21—Al26A	58.64 (11)
Si10—Ru7A—Al11^xv^	156.3 (2)	Al12A—Al21—Al26A	90.67 (13)
Al16^xiv^—Ru7A—Al11^xv^	76.80 (6)	Al12B—Al21—Al24^xvii^	119.0 (6)
Al16^xv^—Ru7A—Al11^xv^	76.80 (6)	Ru7A—Al21—Al24^xvii^	68.48 (6)
Al21—Ru7A—Al19^xvi^	122.02 (4)	Ru2—Al21—Al24^xvii^	134.06 (3)
Al21^i^—Ru7A—Al19^xvi^	122.02 (4)	Ru5—Al21—Al24^xvii^	66.51 (2)
Si10—Ru7A—Al19^xvi^	63.21 (5)	Ru7B—Al21—Al24^xvii^	66.3 (3)
Al16^xiv^—Ru7A—Al19^xvi^	76.07 (12)	Si6^xv^—Al21—Al24^xvii^	104.48 (3)
Al16^xv^—Ru7A—Al19^xvi^	76.07 (12)	Al12A—Al21—Al24^xvii^	105.60 (6)
Al11^xv^—Ru7A—Al19^xvi^	140.45 (19)	Al26A—Al21—Al24^xvii^	163.10 (12)
Al21—Ru7A—Al12B	55.23 (12)	Al12B—Al21—Si26B	66.3 (6)
Al21^i^—Ru7A—Al12B	55.23 (12)	Ru7A—Al21—Si26B	109.80 (10)
Si10—Ru7A—Al12B	81.9 (6)	Ru2—Al21—Si26B	53.66 (7)
Al16^xiv^—Ru7A—Al12B	124.7 (3)	Ru5—Al21—Si26B	118.00 (6)
Al16^xv^—Ru7A—Al12B	124.7 (3)	Ru7B—Al21—Si26B	111.4 (3)
Al11^xv^—Ru7A—Al12B	74.4 (6)	Si6^xv^—Al21—Si26B	67.88 (12)
Al19^xvi^—Ru7A—Al12B	145.1 (6)	Al12A—Al21—Si26B	80.63 (14)
Al21—Ru7A—Al12A	58.55 (10)	Al26A—Al21—Si26B	10.09 (5)
Al21^i^—Ru7A—Al12A	58.55 (10)	Al24^xvii^—Al21—Si26B	171.65 (10)
Si10—Ru7A—Al12A	66.73 (11)	Al12B—Al21—Al22	117.9 (4)
Al16^xiv^—Ru7A—Al12A	130.76 (9)	Ru7A—Al21—Al22	169.24 (9)
Al16^xv^—Ru7A—Al12A	130.76 (9)	Ru2—Al21—Al22	58.56 (2)
Al11^xv^—Ru7A—Al12A	89.61 (12)	Ru5—Al21—Al22	57.47 (2)
Al19^xvi^—Ru7A—Al12A	129.94 (10)	Ru7B—Al21—Al22	165.9 (4)
Al12B—Ru7A—Al12A	15.2 (6)	Si6^xv^—Al21—Al22	62.12 (3)
Al21—Ru7A—Al24^xvii^	59.64 (3)	Al12A—Al21—Al22	120.71 (5)
Al21^i^—Ru7A—Al24^xvii^	149.86 (17)	Al26A—Al21—Al22	60.68 (8)
Si10—Ru7A—Al24^xvii^	62.79 (3)	Al24^xvii^—Al21—Al22	112.49 (3)
Al16^xiv^—Ru7A—Al24^xvii^	132.53 (18)	Si26B—Al21—Al22	67.59 (10)
Al16^xv^—Ru7A—Al24^xvii^	57.51 (4)	Ru9^ix^—Al22—Ru5	133.43 (3)
Al11^xv^—Ru7A—Al24^xvii^	122.30 (3)	Ru9^ix^—Al22—Ru9	76.273 (19)
Al19^xvi^—Ru7A—Al24^xvii^	62.41 (5)	Ru5—Al22—Ru9	140.36 (3)
Al12B—Ru7A—Al24^xvii^	102.7 (3)	Ru9^ix^—Al22—Ru2	143.89 (3)
Al12A—Ru7A—Al24^xvii^	94.82 (10)	Ru5—Al22—Ru2	70.857 (19)
Al16^xiv^—Ru7B—Al16^xv^	99.1 (8)	Ru9—Al22—Ru2	71.503 (19)
Al16^xiv^—Ru7B—Al19^xvi^	81.4 (6)	Ru9^ix^—Al22—Al28A^i^	75.76 (5)
Al16^xv^—Ru7B—Al19^xvi^	81.4 (6)	Ru5—Al22—Al28A^i^	57.77 (5)
Al16^xiv^—Ru7B—Si10	120.2 (3)	Ru9—Al22—Al28A^i^	136.00 (5)
Al16^xv^—Ru7B—Si10	120.2 (3)	Ru2—Al22—Al28A^i^	118.07 (6)
Al19^xvi^—Ru7B—Si10	64.2 (3)	Ru9^ix^—Al22—Al26A	116.62 (6)
Al16^xiv^—Ru7B—Al21	150.9 (5)	Ru5—Al22—Al26A	109.46 (5)
Al16^xv^—Ru7B—Al21	72.18 (11)	Ru9—Al22—Al26A	55.78 (4)
Al19^xvi^—Ru7B—Al21	123.0 (2)	Ru2—Al22—Al26A	55.35 (9)
Si10—Ru7B—Al21	87.1 (5)	Al28A^i^—Al22—Al26A	166.65 (6)
Al16^xiv^—Ru7B—Al21^i^	72.18 (11)	Ru9^ix^—Al22—Si6^xv^	102.48 (3)
Al16^xv^—Ru7B—Al21^i^	150.9 (5)	Ru5—Al22—Si6^xv^	98.12 (3)
Al19^xvi^—Ru7B—Al21^i^	123.0 (2)	Ru9—Al22—Si6^xv^	99.13 (3)
Si10—Ru7B—Al21^i^	87.1 (5)	Ru2—Al22—Si6^xv^	98.35 (3)
Al21—Ru7B—Al21^i^	101.7 (8)	Al28A^i^—Al22—Si6^xv^	119.65 (6)
Al16^xiv^—Ru7B—Al11^xv^	78.6 (3)	Al26A—Al22—Si6^xv^	54.96 (8)
Al16^xv^—Ru7B—Al11^xv^	78.6 (3)	Ru9^ix^—Al22—Al21	156.62 (3)
Al19^xvi^—Ru7B—Al11^xv^	148.8 (10)	Ru5—Al22—Al21	55.59 (2)
Si10—Ru7B—Al11^xv^	147.0 (10)	Ru9—Al22—Al21	109.46 (3)
Al21—Ru7B—Al11^xv^	72.6 (4)	Ru2—Al22—Al21	54.75 (2)
Al21^i^—Ru7B—Al11^xv^	72.6 (4)	Al28A^i^—Al22—Al21	109.73 (5)
Al16^xiv^—Ru7B—Al24^xvii^	140.6 (9)	Al26A—Al22—Al21	56.94 (4)
Al16^xv^—Ru7B—Al24^xvii^	58.93 (16)	Si6^xv^—Al22—Al21	54.63 (2)
Al19^xvi^—Ru7B—Al24^xvii^	64.3 (3)	Ru9^ix^—Al22—Al15^i^	56.09 (2)
Si10—Ru7B—Al24^xvii^	62.3 (2)	Ru5—Al22—Al15^i^	112.97 (3)
Al21—Ru7B—Al24^xvii^	58.76 (13)	Ru9—Al22—Al15^i^	56.57 (2)
Al21^i^—Ru7B—Al24^xvii^	142.6 (8)	Ru2—Al22—Al15^i^	91.92 (3)
Al11^xv^—Ru7B—Al24^xvii^	122.0 (2)	Al28A^i^—Al22—Al15^i^	79.64 (5)
Al16^xiv^—Ru7B—Al24^xvi^	58.93 (16)	Al26A—Al22—Al15^i^	110.98 (5)
Al16^xv^—Ru7B—Al24^xvi^	140.6 (9)	Si6^xv^—Al22—Al15^i^	148.91 (3)
Al19^xvi^—Ru7B—Al24^xvi^	64.3 (3)	Al21—Al22—Al15^i^	146.28 (3)
Si10—Ru7B—Al24^xvi^	62.3 (2)	Ru9^ix^—Al22—Al22^iii^	109.401 (18)
Al21—Ru7B—Al24^xvi^	142.6 (8)	Ru5—Al22—Al22^iii^	56.521 (16)
Al21^i^—Ru7B—Al24^xvi^	58.76 (13)	Ru9—Al22—Al22^iii^	92.250 (17)
Al11^xv^—Ru7B—Al24^xvi^	122.0 (2)	Ru2—Al22—Al22^iii^	57.038 (17)
Al24^xvii^—Ru7B—Al24^xvi^	115.8 (4)	Al28A^i^—Al22—Al22^iii^	66.38 (5)
Al16^xiv^—Ru7B—Ru7B^xviii^	51.5 (3)	Al26A—Al22—Al22^iii^	111.14 (9)
Al16^xv^—Ru7B—Ru7B^xviii^	51.5 (3)	Si6^xv^—Al22—Al22^iii^	147.868 (19)
Al19^xvi^—Ru7B—Ru7B^xviii^	93.1 (4)	Al21—Al22—Al22^iii^	93.25 (2)
Si10—Ru7B—Ru7B^xviii^	157.3 (5)	Al15^i^—Al22—Al22^iii^	59.955 (17)
Al21—Ru7B—Ru7B^xviii^	106.9 (3)	Ru9^ix^—Al22—Al14^ix^	55.43 (2)
Al21^i^—Ru7B—Ru7B^xviii^	106.9 (3)	Ru5—Al22—Al14^ix^	108.75 (3)
Al11^xv^—Ru7B—Ru7B^xviii^	55.8 (5)	Ru9—Al22—Al14^ix^	110.44 (3)
Al24^xvii^—Ru7B—Ru7B^xviii^	109.2 (4)	Ru2—Al22—Al14^ix^	153.68 (4)
Al24^xvi^—Ru7B—Ru7B^xviii^	109.2 (4)	Al28A^i^—Al22—Al14^ix^	79.63 (6)
Ru8^xviii^—Ru8—Si26B^i^	130.06 (17)	Al26A—Al22—Al14^ix^	102.89 (9)
Ru8^xviii^—Ru8—Si26B	130.06 (17)	Si6^xv^—Al22—Al14^ix^	55.36 (3)
Si26B^i^—Ru8—Si26B	93.3 (3)	Al21—Al22—Al14^ix^	102.32 (3)
Ru8^xviii^—Ru8—Si18	117.55 (5)	Al15^i^—Al22—Al14^ix^	111.30 (3)
Si26B^i^—Ru8—Si18	85.04 (12)	Al22^iii^—Al22—Al14^ix^	145.76 (2)
Si26B—Ru8—Si18	85.03 (12)	Ru4^ix^—Al23—Ru5	139.89 (3)
Ru8^xviii^—Ru8—Al26A	119.05 (12)	Ru4^ix^—Al23—Ru4^xv^	77.88 (2)
Si26B^i^—Ru8—Al26A	102.7 (2)	Ru5—Al23—Ru4^xv^	129.65 (3)
Si26B—Ru8—Al26A	11.05 (8)	Ru4^ix^—Al23—Al24^xvii^	137.82 (3)
Si18—Ru8—Al26A	91.76 (9)	Ru5—Al23—Al24^xvii^	66.20 (2)
Ru8^xviii^—Ru8—Al26A^i^	119.05 (12)	Ru4^xv^—Al23—Al24^xvii^	64.62 (2)
Si26B^i^—Ru8—Al26A^i^	11.05 (8)	Ru4^ix^—Al23—Al14^ix^	57.47 (2)
Si26B—Ru8—Al26A^i^	102.7 (2)	Ru5—Al23—Al14^ix^	114.11 (3)
Si18—Ru8—Al26A^i^	91.76 (9)	Ru4^xv^—Al23—Al14^ix^	115.33 (3)
Al26A—Ru8—Al26A^i^	111.25 (18)	Al24^xvii^—Al23—Al14^ix^	157.49 (4)
Ru8^xviii^—Ru8—Si26B^xviii^	43.07 (15)	Ru4^ix^—Al23—Si6^xv^	103.97 (3)
Si26B^i^—Ru8—Si26B^xviii^	87.0 (3)	Ru5—Al23—Si6^xv^	99.99 (2)
Si26B—Ru8—Si26B^xviii^	153.06 (14)	Ru4^xv^—Al23—Si6^xv^	98.39 (3)
Si18—Ru8—Si26B^xviii^	121.78 (6)	Al24^xvii^—Al23—Si6^xv^	100.07 (3)
Al26A—Ru8—Si26B^xviii^	146.00 (8)	Al14^ix^—Al23—Si6^xv^	57.43 (3)
Al26A^i^—Ru8—Si26B^xviii^	76.0 (3)	Ru4^ix^—Al23—Al27	102.08 (2)
Ru8^xviii^—Ru8—Si26B^xix^	43.07 (15)	Ru5—Al23—Al27	51.056 (15)
Si26B^i^—Ru8—Si26B^xix^	153.05 (14)	Ru4^xv^—Al23—Al27	99.56 (2)
Si26B—Ru8—Si26B^xix^	87.0 (3)	Al24^xvii^—Al23—Al27	67.82 (2)
Si18—Ru8—Si26B^xix^	121.78 (6)	Al14^ix^—Al23—Al27	131.35 (3)
Al26A—Ru8—Si26B^xix^	76.0 (3)	Si6^xv^—Al23—Al27	150.89 (3)
Al26A^i^—Ru8—Si26B^xix^	146.00 (8)	Ru4^ix^—Al23—Al23^xix^	56.121 (17)
Si26B^xviii^—Ru8—Si26B^xix^	80.9 (3)	Ru5—Al23—Al23^xix^	159.133 (17)
Ru8^xviii^—Ru8—Al11^xv^	85.69 (4)	Ru4^xv^—Al23—Al23^xix^	57.583 (16)
Si26B^i^—Ru8—Al11^xv^	79.09 (8)	Al24^xvii^—Al23—Al23^xix^	112.01 (2)
Si26B—Ru8—Al11^xv^	79.10 (8)	Al14^ix^—Al23—Al23^xix^	58.992 (18)
Si18—Ru8—Al11^xv^	156.75 (8)	Si6^xv^—Al23—Al23^xix^	59.325 (17)
Al26A—Ru8—Al11^xv^	75.48 (5)	Al27—Al23—Al23^xix^	149.293 (14)
Al26A^i^—Ru8—Al11^xv^	75.48 (5)	Ru4^ix^—Al23—Al23^xxiii^	58.09 (2)
Si26B^xviii^—Ru8—Al11^xv^	74.51 (7)	Ru5—Al23—Al23^xxiii^	110.021 (16)
Si26B^xix^—Ru8—Al11^xv^	74.51 (7)	Ru4^xv^—Al23—Al23^xxiii^	54.723 (19)
Ru8^xviii^—Ru8—Al18^xviii^	54.98 (4)	Al24^xvii^—Al23—Al23^xxiii^	83.77 (3)
Si26B^i^—Ru8—Al18^xviii^	124.37 (8)	Al14^ix^—Al23—Al23^xxiii^	115.24 (4)
Si26B—Ru8—Al18^xviii^	124.36 (8)	Si6^xv^—Al23—Al23^xxiii^	148.424 (19)
Si18—Ru8—Al18^xviii^	62.58 (5)	Al27—Al23—Al23^xxiii^	59.293 (14)
Al26A—Ru8—Al18^xviii^	120.97 (5)	Al23^xix^—Al23—Al23^xxiii^	90.0
Al26A^i^—Ru8—Al18^xviii^	120.97 (5)	Ru4^ix^—Al23—Al28C^i^	90.8 (4)
Si26B^xviii^—Ru8—Al18^xviii^	75.82 (12)	Ru5—Al23—Al28C^i^	50.8 (4)
Si26B^xix^—Ru8—Al18^xviii^	75.82 (12)	Ru4^xv^—Al23—Al28C^i^	123.1 (7)
Al11^xv^—Ru8—Al18^xviii^	140.67 (8)	Al24^xvii^—Al23—Al28C^i^	93.8 (6)
Ru8^xviii^—Ru8—Al26A^xviii^	53.64 (11)	Al14^ix^—Al23—Al28C^i^	103.3 (7)
Si26B^i^—Ru8—Al26A^xviii^	76.4 (3)	Si6^xv^—Al23—Al28C^i^	138.2 (7)
Si26B—Ru8—Al26A^xviii^	150.61 (9)	Al27—Al23—Al28C^i^	28.5 (7)
Si18—Ru8—Al26A^xviii^	120.64 (4)	Al23^xix^—Al23—Al28C^i^	146.9 (4)
Al26A—Ru8—Al26A^xviii^	147.02 (8)	Al23^xxiii^—Al23—Al28C^i^	71.9 (6)
Al26A^i^—Ru8—Al26A^xviii^	65.4 (2)	Ru4^ix^—Al23—Al21	158.01 (3)
Si26B^xviii^—Ru8—Al26A^xviii^	10.60 (7)	Ru5—Al23—Al21	54.62 (2)
Si26B^xix^—Ru8—Al26A^xviii^	90.2 (2)	Ru4^xv^—Al23—Al21	104.25 (3)
Al11^xv^—Ru8—Al26A^xviii^	71.98 (4)	Al24^xvii^—Al23—Al21	57.17 (2)
Al18^xviii^—Ru8—Al26A^xviii^	82.95 (9)	Al14^ix^—Al23—Al21	103.50 (3)
Si13—Ru9—Al22^ix^	143.18 (3)	Si6^xv^—Al23—Al21	54.05 (2)
Si13—Ru9—Al14	74.93 (3)	Al27—Al23—Al21	99.14 (3)
Al22^ix^—Ru9—Al14	69.25 (2)	Al23^xix^—Al23—Al21	106.01 (2)
Si13—Ru9—Al26A	76.19 (12)	Al23^xxiii^—Al23—Al21	140.86 (4)
Al22^ix^—Ru9—Al26A	137.56 (11)	Al28C^i^—Al23—Al21	105.4 (4)
Al14—Ru9—Al26A	150.45 (13)	Ru4^ix^—Al23—Al22	112.68 (3)
Si13—Ru9—Al15^xx^	144.71 (3)	Ru5—Al23—Al22	55.75 (2)
Al22^ix^—Ru9—Al15^xx^	68.60 (3)	Ru4^xv^—Al23—Al22	156.11 (3)
Al14—Ru9—Al15^xx^	137.45 (3)	Al24^xvii^—Al23—Al22	109.38 (3)
Al26A—Ru9—Al15^xx^	69.48 (11)	Al14^ix^—Al23—Al22	60.73 (2)
Si13—Ru9—Al15^i^	104.138 (19)	Si6^xv^—Al23—Al22	58.86 (2)
Al22^ix^—Ru9—Al15^i^	68.15 (2)	Al27—Al23—Al22	98.94 (3)
Al14—Ru9—Al15^i^	64.92 (3)	Al23^xix^—Al23—Al22	109.21 (2)
Al26A—Ru9—Al15^i^	129.36 (6)	Al23^xxiii^—Al23—Al22	149.15 (4)
Al15^xx^—Ru9—Al15^i^	103.749 (11)	Al28C^i^—Al23—Al22	79.3 (7)
Si13—Ru9—Al22	105.35 (3)	Al21—Al23—Al22	57.77 (2)
Al22^ix^—Ru9—Al22	103.725 (19)	Al28C^xx^—Al24—Ru3^xx^	71.1 (7)
Al14—Ru9—Al22	129.31 (2)	Al28C^xx^—Al24—Al16	144.5 (3)
Al26A—Ru9—Al22	65.23 (9)	Ru3^xx^—Al24—Al16	129.42 (4)
Al15^xx^—Ru9—Al22	67.48 (2)	Al28C^xx^—Al24—Al28B^xx^	13.5 (6)
Al15^i^—Ru9—Al22	66.15 (3)	Ru3^xx^—Al24—Al28B^xx^	60.3 (5)
Si13—Ru9—Si17B^xx^	102.8 (4)	Al16—Al24—Al28B^xx^	144.0 (2)
Al22^ix^—Ru9—Si17B^xx^	82.4 (5)	Al28C^xx^—Al24—Al23^x^	80.9 (7)
Al14—Ru9—Si17B^xx^	110.4 (4)	Ru3^xx^—Al24—Al23^x^	151.18 (4)
Al26A—Ru9—Si17B^xx^	70.2 (5)	Al16—Al24—Al23^x^	72.20 (4)
Al15^xx^—Ru9—Si17B^xx^	58.2 (2)	Al28B^xx^—Al24—Al23^x^	90.9 (5)
Al15^i^—Ru9—Si17B^xx^	150.0 (5)	Al28C^xx^—Al24—Al21^x^	122.4 (8)
Al22—Ru9—Si17B^xx^	118.4 (3)	Ru3^xx^—Al24—Al21^x^	136.11 (3)
Si13—Ru9—Si26B	66.13 (13)	Al16—Al24—Al21^x^	66.43 (3)
Al22^ix^—Ru9—Si26B	145.78 (11)	Al28B^xx^—Al24—Al21^x^	135.6 (5)
Al14—Ru9—Si26B	139.92 (13)	Al23^x^—Al24—Al21^x^	65.80 (3)
Al26A—Ru9—Si26B	10.56 (5)	Al28C^xx^—Al24—Al25	82.2 (8)
Al15^xx^—Ru9—Si26B	79.06 (13)	Ru3^xx^—Al24—Al25	58.88 (2)
Al15^i^—Ru9—Si26B	132.98 (6)	Al16—Al24—Al25	86.60 (4)
Al22—Ru9—Si26B	72.46 (10)	Al28B^xx^—Al24—Al25	70.0 (5)
Si17B^xx^—Ru9—Si26B	71.0 (6)	Al23^x^—Al24—Al25	111.61 (3)
Si13—Ru9—Al17A^xx^	94.94 (4)	Al21^x^—Al24—Al25	152.63 (4)
Al22^ix^—Ru9—Al17A^xx^	94.80 (5)	Al28C^xx^—Al24—Al24^xix^	55.8 (3)
Al14—Ru9—Al17A^xx^	118.76 (4)	Ru3^xx^—Al24—Al24^xix^	56.826 (17)
Al26A—Ru9—Al17A^xx^	57.86 (6)	Al16—Al24—Al24^xix^	157.27 (3)
Al15^xx^—Ru9—Al17A^xx^	59.96 (3)	Al28B^xx^—Al24—Al24^xix^	58.6 (2)
Al15^i^—Ru9—Al17A^xx^	160.71 (4)	Al23^x^—Al24—Al24^xix^	112.00 (2)
Al22—Ru9—Al17A^xx^	111.76 (4)	Al21^x^—Al24—Al24^xix^	94.28 (2)
Si17B^xx^—Ru9—Al17A^xx^	13.0 (5)	Al25—Al24—Al24^xix^	110.87 (3)
Si26B—Ru9—Al17A^xx^	58.07 (8)	Al28C^xx^—Al24—Si10^xxvii^	112.9 (4)
Ru7A^ii^—Si10—Ru7A	125.3 (2)	Ru3^xx^—Al24—Si10^xxvii^	58.14 (3)
Ru7A^ii^—Si10—Ru7B^ii^	4.6 (4)	Al16—Al24—Si10^xxvii^	102.42 (3)
Ru7A—Si10—Ru7B^ii^	129.9 (6)	Al28B^xx^—Al24—Si10^xxvii^	109.2 (3)
Ru7A^ii^—Si10—Ru7B	129.9 (6)	Al23^x^—Al24—Si10^xxvii^	144.16 (4)
Ru7A—Si10—Ru7B	4.6 (4)	Al21^x^—Al24—Si10^xxvii^	79.43 (4)
Ru7B^ii^—Si10—Ru7B	134.5 (10)	Al25—Al24—Si10^xxvii^	103.20 (4)
Ru7A^ii^—Si10—Ru3^xxiv^	112.02 (9)	Al24^xix^—Al24—Si10^xxvii^	60.455 (15)
Ru7A—Si10—Ru3^xxiv^	112.02 (9)	Al28C^xx^—Al24—Ru4	92.4 (3)
Ru7B^ii^—Si10—Ru3^xxiv^	108.4 (4)	Ru3^xx^—Al24—Ru4	116.86 (3)
Ru7B—Si10—Ru3^xxiv^	108.4 (4)	Al16—Al24—Ru4	53.47 (3)
Ru7A^ii^—Si10—Ru3^vi^	112.02 (9)	Al28B^xx^—Al24—Ru4	90.57 (19)
Ru7A—Si10—Ru3^vi^	112.02 (9)	Al23^x^—Al24—Ru4	56.57 (2)
Ru7B^ii^—Si10—Ru3^vi^	108.4 (4)	Al21^x^—Al24—Ru4	104.72 (3)
Ru7B—Si10—Ru3^vi^	108.4 (4)	Al25—Al24—Ru4	58.66 (2)
Ru3^xxiv^—Si10—Ru3^vi^	70.63 (3)	Al24^xix^—Al24—Ru4	148.210 (16)
Ru7A^ii^—Si10—Al19^xxi^	60.62 (11)	Si10^xxvii^—Al24—Ru4	147.42 (3)
Ru7A—Si10—Al19^xxi^	174.09 (12)	Al28C^xx^—Al24—Al19	128.9 (8)
Ru7B^ii^—Si10—Al19^xxi^	56.0 (5)	Ru3^xx^—Al24—Al19	61.31 (3)
Ru7B—Si10—Al19^xxi^	169.5 (5)	Al16—Al24—Al19	69.38 (4)
Ru3^xxiv^—Si10—Al19^xxi^	63.41 (3)	Al28B^xx^—Al24—Al19	115.7 (5)
Ru3^vi^—Si10—Al19^xxi^	63.41 (3)	Al23^x^—Al24—Al19	140.66 (4)
Ru7A^ii^—Si10—Al19^xvi^	174.09 (12)	Al21^x^—Al24—Al19	105.11 (3)
Ru7A—Si10—Al19^xvi^	60.62 (11)	Al25—Al24—Al19	58.34 (3)
Ru7B^ii^—Si10—Al19^xvi^	169.5 (5)	Al24^xix^—Al24—Al19	106.70 (3)
Ru7B—Si10—Al19^xvi^	56.0 (5)	Si10^xxvii^—Al24—Al19	55.54 (3)
Ru3^xxiv^—Si10—Al19^xvi^	63.41 (3)	Ru4—Al24—Al19	92.85 (3)
Ru3^vi^—Si10—Al19^xvi^	63.41 (3)	Al28C^xx^—Al24—Ru5^xxvii^	68.3 (8)
Al19^xxi^—Si10—Al19^xvi^	113.47 (6)	Ru3^xx^—Al24—Ru5^xxvii^	117.27 (3)
Ru7A^ii^—Si10—Al24^xxi^	66.31 (4)	Al16—Al24—Ru5^xxvii^	111.16 (4)
Ru7A—Si10—Al24^xxi^	118.30 (3)	Al28B^xx^—Al24—Ru5^xxvii^	81.4 (5)
Ru7B^ii^—Si10—Al24^xxi^	64.89 (15)	Al23^x^—Al24—Ru5^xxvii^	54.85 (2)
Ru7B—Si10—Al24^xxi^	119.03 (7)	Al21^x^—Al24—Ru5^xxvii^	54.16 (2)
Ru3^xxiv^—Si10—Al24^xxi^	55.588 (19)	Al25—Al24—Ru5^xxvii^	148.62 (4)
Ru3^vi^—Si10—Al24^xxi^	115.89 (4)	Al24^xix^—Al24—Ru5^xxvii^	61.040 (15)
Al19^xxi^—Si10—Al24^xxi^	62.902 (19)	Si10^xxvii^—Al24—Ru5^xxvii^	98.06 (3)
Al19^xvi^—Si10—Al24^xxi^	111.66 (3)	Ru4—Al24—Ru5^xxvii^	110.63 (2)
Ru7A^ii^—Si10—Al24^xvi^	118.31 (3)	Al19—Al24—Ru5^xxvii^	151.32 (3)
Ru7A—Si10—Al24^xvi^	66.31 (4)	Si13—Al25—Al28A^xx^	124.19 (6)
Ru7B^ii^—Si10—Al24^xvi^	119.03 (7)	Si13—Al25—Ru3^xx^	131.16 (3)
Ru7B—Si10—Al24^xvi^	64.90 (15)	Al28A^xx^—Al25—Ru3^xx^	60.04 (5)
Ru3^xxiv^—Si10—Al24^xvi^	115.89 (4)	Si13—Al25—Al14	69.92 (3)
Ru3^vi^—Si10—Al24^xvi^	55.588 (19)	Al28A^xx^—Al25—Al14	84.62 (5)
Al19^xxi^—Si10—Al24^xvi^	111.66 (3)	Ru3^xx^—Al25—Al14	144.54 (4)
Al19^xvi^—Si10—Al24^xvi^	62.902 (18)	Si13—Al25—Al19	107.28 (4)
Al24^xxi^—Si10—Al24^xvi^	170.96 (6)	Al28A^xx^—Al25—Al19	119.27 (6)
Ru7A^ii^—Si10—Al24^xvii^	118.31 (3)	Ru3^xx^—Al25—Al19	62.41 (3)
Ru7A—Si10—Al24^xvii^	66.31 (4)	Al14—Al25—Al19	147.27 (5)
Ru7B^ii^—Si10—Al24^xvii^	119.03 (7)	Si13—Al25—Ru4	110.10 (3)
Ru7B—Si10—Al24^xvii^	64.90 (15)	Al28A^xx^—Al25—Ru4	92.85 (5)
Ru3^xxiv^—Si10—Al24^xvii^	55.588 (19)	Ru3^xx^—Al25—Ru4	118.47 (3)
Ru3^vi^—Si10—Al24^xvii^	115.89 (4)	Al14—Al25—Ru4	56.01 (2)
Al19^xxi^—Si10—Al24^xvii^	111.66 (3)	Al19—Al25—Ru4	98.03 (3)
Al19^xvi^—Si10—Al24^xvii^	62.902 (19)	Si13—Al25—Al24	164.76 (5)
Al24^xxi^—Si10—Al24^xvii^	59.09 (3)	Al28A^xx^—Al25—Al24	70.66 (5)
Al24^xvi^—Si10—Al24^xvii^	120.09 (3)	Ru3^xx^—Al25—Al24	56.71 (2)
Ru7A^ii^—Si10—Al24^xxii^	66.31 (4)	Al14—Al25—Al24	111.40 (4)
Ru7A—Si10—Al24^xxii^	118.30 (3)	Al19—Al25—Al24	62.90 (3)
Ru7B^ii^—Si10—Al24^xxii^	64.89 (15)	Ru4—Al25—Al24	62.46 (2)
Ru7B—Si10—Al24^xxii^	119.03 (7)	Si13—Al25—Si17B^xx^	94.5 (4)
Ru3^xxiv^—Si10—Al24^xxii^	115.89 (4)	Al28A^xx^—Al25—Si17B^xx^	42.9 (5)
Ru3^vi^—Si10—Al24^xxii^	55.588 (19)	Ru3^xx^—Al25—Si17B^xx^	54.1 (2)
Al19^xxi^—Si10—Al24^xxii^	62.902 (19)	Al14—Al25—Si17B^xx^	102.6 (3)
Al19^xvi^—Si10—Al24^xxii^	111.66 (3)	Al19—Al25—Si17B^xx^	110.1 (3)
Al24^xxi^—Si10—Al24^xxii^	120.09 (3)	Ru4—Al25—Si17B^xx^	135.0 (5)
Al24^xvi^—Si10—Al24^xxii^	59.09 (3)	Al24—Al25—Si17B^xx^	99.8 (3)
Al24^xvii^—Si10—Al24^xxii^	170.96 (6)	Si13—Al25—Ru9	52.79 (2)
Ru8^xxv^—Al11—Ru8^xi^	8.64 (8)	Al28A^xx^—Al25—Ru9	71.76 (5)
Ru8^xxv^—Al11—Ru7A^xi^	145.33 (12)	Ru3^xx^—Al25—Ru9	110.61 (3)
Ru8^xi^—Al11—Ru7A^xi^	136.69 (12)	Al14—Al25—Ru9	55.04 (2)
Ru8^xxv^—Al11—Ru7A^xxv^	136.69 (12)	Al19—Al25—Ru9	149.49 (4)
Ru8^xi^—Al11—Ru7A^xxv^	145.33 (12)	Ru4—Al25—Ru9	110.23 (3)
Ru7A^xi^—Al11—Ru7A^xxv^	78.0 (2)	Al24—Al25—Ru9	141.11 (4)
Ru8^xxv^—Al11—Ru7B^xi^	150.1 (5)	Si17B^xx^—Al25—Ru9	56.5 (2)
Ru8^xi^—Al11—Ru7B^xi^	141.4 (5)	Si13—Al25—Si18	76.05 (3)
Ru7A^xi^—Al11—Ru7B^xi^	4.7 (4)	Al28A^xx^—Al25—Si18	105.43 (6)
Ru7A^xxv^—Al11—Ru7B^xi^	73.2 (6)	Ru3^xx^—Al25—Si18	58.55 (3)
Ru8^xxv^—Al11—Ru7B^xxv^	141.4 (5)	Al14—Al25—Si18	143.89 (4)
Ru8^xi^—Al11—Ru7B^xxv^	150.1 (5)	Al19—Al25—Si18	55.73 (3)
Ru7A^xi^—Al11—Ru7B^xxv^	73.2 (6)	Ru4—Al25—Si18	152.96 (4)
Ru7A^xxv^—Al11—Ru7B^xxv^	4.7 (4)	Al24—Al25—Si18	104.62 (4)
Ru7B^xi^—Al11—Ru7B^xxv^	68.5 (10)	Si17B^xx^—Al25—Si18	68.0 (5)
Al12B^ii^—Al12A—Al12B	108 (3)	Ru9—Al25—Si18	94.56 (3)
Al12B^ii^—Al12A—Al21^iii^	67.5 (8)	Si26B—Al26A—Ru8	56.5 (4)
Al12B—Al12A—Al21^iii^	127.4 (4)	Si26B—Al26A—Ru2	73.2 (4)
Al12B^ii^—Al12A—Al21^i^	127.4 (4)	Ru8—Al26A—Ru2	127.6 (2)
Al12B—Al12A—Al21^i^	67.5 (8)	Si26B—Al26A—Ru9	101.8 (4)
Al21^iii^—Al12A—Al21^i^	157.96 (11)	Ru8—Al26A—Ru9	125.89 (10)
Al12B^ii^—Al12A—Al21^ii^	67.5 (8)	Ru2—Al26A—Ru9	74.86 (4)
Al12B—Al12A—Al21^ii^	127.4 (4)	Si26B—Al26A—Si6^xv^	149.4 (4)
Al21^iii^—Al12A—Al21^ii^	100.34 (4)	Ru8—Al26A—Si6^xv^	108.54 (8)
Al21^i^—Al12A—Al21^ii^	75.37 (3)	Ru2—Al26A—Si6^xv^	107.54 (11)
Al12B^ii^—Al12A—Al21	127.4 (4)	Ru9—Al26A—Si6^xv^	107.90 (18)
Al12B—Al12A—Al21	67.5 (8)	Si26B—Al26A—Al17A^xx^	92.8 (4)
Al21^iii^—Al12A—Al21	75.37 (3)	Ru8—Al26A—Al17A^xx^	66.69 (7)
Al21^i^—Al12A—Al21	100.34 (4)	Ru2—Al26A—Al17A^xx^	134.59 (9)
Al21^ii^—Al12A—Al21	157.96 (11)	Ru9—Al26A—Al17A^xx^	65.89 (6)
Al12B^ii^—Al12A—Ru2^i^	71.4 (7)	Si6^xv^—Al26A—Al17A^xx^	105.56 (15)
Al12B—Al12A—Ru2^i^	71.4 (7)	Si26B—Al26A—Al20	12.3 (4)
Al21^iii^—Al12A—Ru2^i^	138.44 (5)	Ru8—Al26A—Al20	66.65 (14)
Al21^i^—Al12A—Ru2^i^	57.296 (18)	Ru2—Al26A—Al20	61.85 (11)
Al21^ii^—Al12A—Ru2^i^	57.296 (18)	Ru9—Al26A—Al20	101.79 (13)
Al21—Al12A—Ru2^i^	138.44 (5)	Si6^xv^—Al26A—Al20	144.43 (8)
Al12B^ii^—Al12A—Ru2	71.4 (7)	Al17A^xx^—Al26A—Al20	104.15 (16)
Al12B—Al12A—Ru2	71.4 (7)	Si26B—Al26A—Ru8^xviii^	63.8 (4)
Al21^iii^—Al12A—Ru2	57.297 (18)	Ru8—Al26A—Ru8^xviii^	7.31 (7)
Al21^i^—Al12A—Ru2	138.44 (5)	Ru2—Al26A—Ru8^xviii^	134.7 (2)
Al21^ii^—Al12A—Ru2	138.44 (5)	Ru9—Al26A—Ru8^xviii^	126.28 (8)
Al21—Al12A—Ru2	57.298 (18)	Si6^xv^—Al26A—Ru8^xviii^	102.45 (8)
Ru2^i^—Al12A—Ru2	114.07 (9)	Al17A^xx^—Al26A—Ru8^xviii^	63.79 (7)
Al12B^ii^—Al12A—Al20	72.2 (15)	Al20—Al26A—Ru8^xviii^	73.95 (14)
Al12B—Al12A—Al20	35.9 (15)	Si26B—Al26A—Al21	100.9 (4)
Al21^iii^—Al12A—Al20	111.84 (7)	Ru8—Al26A—Al21	115.23 (8)
Al21^i^—Al12A—Al20	89.50 (5)	Ru2—Al26A—Al21	57.85 (3)
Al21^ii^—Al12A—Al20	111.84 (7)	Ru9—Al26A—Al21	117.54 (9)
Al21—Al12A—Al20	89.50 (5)	Si6^xv^—Al26A—Al21	58.88 (8)
Ru2^i^—Al12A—Al20	58.86 (4)	Al17A^xx^—Al26A—Al21	164.4 (2)
Ru2—Al12A—Al20	58.86 (4)	Al20—Al26A—Al21	90.24 (11)
Al12B^ii^—Al12A—Al20^ii^	35.9 (15)	Ru8^xviii^—Al26A—Al21	116.01 (7)
Al12B—Al12A—Al20^ii^	72.2 (15)	Si26B—Al26A—Al26A^xix^	117.3 (4)
Al21^iii^—Al12A—Al20^ii^	89.50 (5)	Ru8—Al26A—Al26A^xix^	60.96 (12)
Al21^i^—Al12A—Al20^ii^	111.84 (7)	Ru2—Al26A—Al26A^xix^	165.22 (9)
Al21^ii^—Al12A—Al20^ii^	89.50 (5)	Ru9—Al26A—Al26A^xix^	110.82 (8)
Al21—Al12A—Al20^ii^	111.84 (7)	Si6^xv^—Al26A—Al26A^xix^	57.95 (3)
Ru2^i^—Al12A—Al20^ii^	58.86 (4)	Al17A^xx^—Al26A—Al26A^xix^	58.02 (7)
Ru2—Al12A—Al20^ii^	58.86 (4)	Al20—Al26A—Al26A^xix^	127.49 (5)
Al20—Al12A—Al20^ii^	36.28 (10)	Ru8^xviii^—Al26A—Al26A^xix^	53.64 (11)
Al12B^ii^—Al12A—Ru7A	176.6 (15)	Al21—Al26A—Al26A^xix^	108.38 (7)
Al12B—Al12A—Ru7A	75.3 (15)	Al26A—Si26B—Al20	164.8 (5)
Al21^iii^—Al12A—Ru7A	110.57 (6)	Al26A—Si26B—Ru8	112.4 (4)
Al21^i^—Al12A—Ru7A	53.57 (3)	Al20—Si26B—Ru8	80.0 (2)
Al21^ii^—Al12A—Ru7A	110.57 (6)	Al26A—Si26B—Ru2	95.3 (4)
Al21—Al12A—Ru7A	53.57 (3)	Al20—Si26B—Ru2	70.59 (14)
Ru2^i^—Al12A—Ru7A	110.196 (14)	Ru8—Si26B—Ru2	148.4 (3)
Ru2—Al12A—Ru7A	110.197 (14)	Al26A—Si26B—Ru8^xviii^	105.6 (4)
Al20—Al12A—Ru7A	111.24 (6)	Al20—Si26B—Ru8^xviii^	86.8 (2)
Al20^ii^—Al12A—Ru7A	147.52 (8)	Ru8—Si26B—Ru8^xviii^	6.87 (7)
Al12A—Al12B—Al12B^ii^	36.0 (15)	Ru2—Si26B—Ru8^xviii^	154.5 (3)
Al12A—Al12B—Al20	133 (2)	Al26A—Si26B—Al17A^xx^	76.3 (4)
Al12B^ii^—Al12B—Al20	96.6 (7)	Al20—Si26B—Al17A^xx^	117.6 (2)
Al12A—Al12B—Al21^i^	96.1 (10)	Ru8—Si26B—Al17A^xx^	68.57 (8)
Al12B^ii^—Al12B—Al21^i^	114.2 (6)	Ru2—Si26B—Al17A^xx^	135.85 (12)
Al20—Al12B—Al21^i^	109.6 (8)	Ru8^xviii^—Si26B—Al17A^xx^	65.16 (8)
Al12A—Al12B—Al21	96.1 (10)	Al26A—Si26B—Ru9	67.6 (4)
Al12B^ii^—Al12B—Al21	114.2 (6)	Al20—Si26B—Ru9	111.86 (15)
Al20—Al12B—Al21	109.6 (8)	Ru8—Si26B—Ru9	129.86 (10)
Al21^i^—Al12B—Al21	111.5 (8)	Ru2—Si26B—Ru9	73.78 (8)
Al12A—Al12B—Ru2^i^	92.4 (10)	Ru8^xviii^—Si26B—Ru9	127.65 (10)
Al12B^ii^—Al12B—Ru2^i^	76.2 (6)	Al17A^xx^—Si26B—Ru9	62.85 (9)
Al20—Al12B—Ru2^i^	68.2 (5)	Al26A—Si26B—Si13	117.4 (4)
Al21^i^—Al12B—Ru2^i^	61.39 (6)	Al20—Si26B—Si13	59.77 (13)
Al21—Al12B—Ru2^i^	169.5 (12)	Ru8—Si26B—Si13	117.9 (2)
Al12A—Al12B—Ru2	92.4 (10)	Ru2—Si26B—Si13	55.64 (7)
Al12B^ii^—Al12B—Ru2	76.2 (6)	Ru8^xviii^—Si26B—Si13	122.8 (2)
Al20—Al12B—Ru2	68.2 (5)	Al17A^xx^—Si26B—Si13	89.50 (8)
Al21^i^—Al12B—Ru2	169.5 (12)	Ru9—Si26B—Si13	52.12 (5)
Al21—Al12B—Ru2	61.39 (6)	Al26A—Si26B—Al21	69.0 (4)
Ru2^i^—Al12B—Ru2	124.4 (8)	Al20—Si26B—Al21	97.72 (13)
Al12A—Al12B—Al20^ii^	92.1 (17)	Ru8—Si26B—Al21	118.41 (9)
Al12B^ii^—Al12B—Al20^ii^	56.1 (6)	Ru2—Si26B—Al21	56.84 (6)
Al20—Al12B—Al20^ii^	40.5 (3)	Ru8^xviii^—Si26B—Al21	117.29 (9)
Al21^i^—Al12B—Al20^ii^	123.3 (4)	Al17A^xx^—Si26B—Al21	144.5 (3)
Al21—Al12B—Al20^ii^	123.3 (4)	Ru9—Si26B—Al21	108.29 (15)
Ru2^i^—Al12B—Al20^ii^	62.3 (4)	Si13—Si26B—Al21	112.48 (9)
Ru2—Al12B—Al20^ii^	62.3 (4)	Al26A—Si26B—Al12B	119.2 (5)
Al12A—Al12B—Ru7A	89.5 (16)	Al20—Si26B—Al12B	48.4 (3)
Al12B^ii^—Al12B—Ru7A	125.5 (6)	Ru8—Si26B—Al12B	96.0 (4)
Al20—Al12B—Ru7A	138.0 (12)	Ru2—Si26B—Al12B	55.7 (4)
Al21^i^—Al12B—Ru7A	56.5 (4)	Ru8^xviii^—Si26B—Al12B	100.4 (4)
Al21—Al12B—Ru7A	56.5 (4)	Al17A^xx^—Si26B—Al12B	162.3 (4)
Ru2^i^—Al12B—Ru7A	117.7 (4)	Ru9—Si26B—Al12B	129.1 (4)
Ru2—Al12B—Ru7A	117.7 (4)	Si13—Si26B—Al12B	90.3 (4)
Al20^ii^—Al12B—Ru7A	178.5 (12)	Al21—Si26B—Al12B	50.2 (3)
Al12A—Al12B—Si26B^i^	144.6 (7)	Al28C^i^—Al27—Al28C^xxiv^	180 (3)
Al12B^ii^—Al12B—Si26B^i^	123.9 (4)	Al28C^i^—Al27—Al28B^i^	8.0 (11)
Al20—Al12B—Si26B^i^	47.2 (5)	Al28C^xxiv^—Al27—Al28B^i^	172.0 (11)
Al21^i^—Al12B—Si26B^i^	63.5 (4)	Al28C^i^—Al27—Al28B^xxiv^	172.0 (11)
Al21—Al12B—Si26B^i^	117.8 (10)	Al28C^xxiv^—Al27—Al28B^xxiv^	8.0 (11)
Ru2^i^—Al12B—Si26B^i^	52.9 (3)	Al28B^i^—Al27—Al28B^xxiv^	180.0 (13)
Ru2—Al12B—Si26B^i^	111.9 (9)	Al28C^i^—Al27—Ru5^xxviii^	106.3 (11)
Al20^ii^—Al12B—Si26B^i^	78.3 (6)	Al28C^xxiv^—Al27—Ru5^xxviii^	73.7 (11)
Ru7A—Al12B—Si26B^i^	100.5 (7)	Al28B^i^—Al27—Ru5^xxviii^	114.3 (5)
Al12A—Al12B—Si26B	144.6 (7)	Al28B^xxiv^—Al27—Ru5^xxviii^	65.7 (5)
Al12B^ii^—Al12B—Si26B	123.9 (4)	Al28C^i^—Al27—Ru5	73.7 (11)
Al20—Al12B—Si26B	47.2 (5)	Al28C^xxiv^—Al27—Ru5	106.3 (11)
Al21^i^—Al12B—Si26B	117.8 (10)	Al28B^i^—Al27—Ru5	65.7 (5)
Al21—Al12B—Si26B	63.5 (4)	Al28B^xxiv^—Al27—Ru5	114.3 (5)
Ru2^i^—Al12B—Si26B	111.9 (9)	Ru5^xxviii^—Al27—Ru5	180.0
Ru2—Al12B—Si26B	52.9 (3)	Al28C^i^—Al27—Al23^xxviii^	101.3 (5)
Al20^ii^—Al12B—Si26B	78.3 (6)	Al28C^xxiv^—Al27—Al23^xxviii^	78.7 (5)
Ru7A—Al12B—Si26B	100.5 (7)	Al28B^i^—Al27—Al23^xxviii^	105.0 (2)
Si26B^i^—Al12B—Si26B	67.0 (6)	Al28B^xxiv^—Al27—Al23^xxviii^	75.0 (2)
Ru9—Si13—Ru9^iii^	79.25 (3)	Ru5^xxviii^—Al27—Al23^xxviii^	59.495 (14)
Ru9—Si13—Ru1	133.97 (2)	Ru5—Al27—Al23^xxviii^	120.505 (14)
Ru9^iii^—Si13—Ru1	133.97 (2)	Al28C^i^—Al27—Al23^iii^	78.7 (5)
Ru9—Si13—Ru2	78.47 (2)	Al28C^xxiv^—Al27—Al23^iii^	101.3 (5)
Ru9^iii^—Si13—Ru2	78.46 (2)	Al28B^i^—Al27—Al23^iii^	75.0 (2)
Ru1—Si13—Ru2	130.71 (4)	Al28B^xxiv^—Al27—Al23^iii^	105.0 (2)
Ru9—Si13—Al20^ii^	138.45 (5)	Ru5^xxviii^—Al27—Al23^iii^	120.505 (14)
Ru9^iii^—Si13—Al20^ii^	108.59 (4)	Ru5—Al27—Al23^iii^	59.495 (14)
Ru1—Si13—Al20^ii^	69.62 (4)	Al23^xxviii^—Al27—Al23^iii^	61.41 (3)
Ru2—Si13—Al20^ii^	64.15 (4)	Al28C^i^—Al27—Al23^xxiii^	101.3 (5)
Ru9—Si13—Al20	108.59 (4)	Al28C^xxiv^—Al27—Al23^xxiii^	78.7 (5)
Ru9^iii^—Si13—Al20	138.44 (5)	Al28B^i^—Al27—Al23^xxiii^	105.0 (2)
Ru1—Si13—Al20	69.62 (4)	Al28B^xxiv^—Al27—Al23^xxiii^	75.0 (2)
Ru2—Si13—Al20	64.15 (4)	Ru5^xxviii^—Al27—Al23^xxiii^	59.495 (14)
Al20^ii^—Si13—Al20	39.36 (10)	Ru5—Al27—Al23^xxiii^	120.505 (14)
Ru9—Si13—Al25	68.38 (3)	Al23^xxviii^—Al27—Al23^xxiii^	118.59 (3)
Ru9^iii^—Si13—Al25	109.91 (4)	Al23^iii^—Al27—Al23^xxiii^	180.00 (4)
Ru1—Si13—Al25	69.91 (3)	Al28C^i^—Al27—Al23	78.7 (5)
Ru2—Si13—Al25	142.93 (3)	Al28C^xxiv^—Al27—Al23	101.3 (5)
Al20^ii^—Si13—Al25	136.87 (5)	Al28B^i^—Al27—Al23	75.0 (2)
Al20—Si13—Al25	110.82 (5)	Al28B^xxiv^—Al27—Al23	105.0 (2)
Ru9—Si13—Al25^iii^	109.91 (4)	Ru5^xxviii^—Al27—Al23	120.505 (14)
Ru9^iii^—Si13—Al25^iii^	68.38 (3)	Ru5—Al27—Al23	59.495 (14)
Ru1—Si13—Al25^iii^	69.91 (3)	Al23^xxviii^—Al27—Al23	180.0
Ru2—Si13—Al25^iii^	142.93 (3)	Al23^iii^—Al27—Al23	118.59 (3)
Al20^ii^—Si13—Al25^iii^	110.82 (5)	Al23^xxiii^—Al27—Al23	61.41 (3)
Al20—Si13—Al25^iii^	136.87 (5)	Al28C^i^—Al27—Al28A^i^	18.1 (10)
Al25—Si13—Al25^iii^	67.53 (4)	Al28C^xxiv^—Al27—Al28A^i^	161.9 (10)
Ru9—Si13—Si26B	61.75 (12)	Al28B^i^—Al27—Al28A^i^	11.3 (4)
Ru9^iii^—Si13—Si26B	122.39 (11)	Al28B^xxiv^—Al27—Al28A^i^	168.7 (4)
Ru1—Si13—Si26B	103.17 (11)	Ru5^xxviii^—Al27—Al28A^i^	122.36 (4)
Ru2—Si13—Si26B	54.62 (6)	Ru5—Al27—Al28A^i^	57.64 (4)
Al20^ii^—Si13—Si26B	81.32 (11)	Al23^xxviii^—Al27—Al28A^i^	115.61 (5)
Al20—Si13—Si26B	46.86 (13)	Al23^iii^—Al27—Al28A^i^	78.24 (5)
Al25—Si13—Si26B	93.92 (6)	Al23^xxiii^—Al27—Al28A^i^	101.76 (5)
Al25^iii^—Si13—Si26B	161.39 (6)	Al23—Al27—Al28A^i^	64.39 (5)
Ru9—Si13—Si26B^iii^	122.39 (11)	Al28C^i^—Al27—Al28A^xxiv^	161.9 (10)
Ru9^iii^—Si13—Si26B^iii^	61.75 (12)	Al28C^xxiv^—Al27—Al28A^xxiv^	18.1 (10)
Ru1—Si13—Si26B^iii^	103.17 (11)	Al28B^i^—Al27—Al28A^xxiv^	168.7 (4)
Ru2—Si13—Si26B^iii^	54.62 (6)	Al28B^xxiv^—Al27—Al28A^xxiv^	11.3 (4)
Al20^ii^—Si13—Si26B^iii^	46.86 (13)	Ru5^xxviii^—Al27—Al28A^xxiv^	57.64 (4)
Al20—Si13—Si26B^iii^	81.32 (11)	Ru5—Al27—Al28A^xxiv^	122.36 (4)
Al25—Si13—Si26B^iii^	161.40 (6)	Al23^xxviii^—Al27—Al28A^xxiv^	64.39 (5)
Al25^iii^—Si13—Si26B^iii^	93.93 (6)	Al23^iii^—Al27—Al28A^xxiv^	101.76 (5)
Si26B—Si13—Si26B^iii^	104.56 (11)	Al23^xxiii^—Al27—Al28A^xxiv^	78.24 (5)
Ru9—Al14—Ru9^iii^	74.75 (2)	Al23—Al27—Al28A^xxiv^	115.61 (5)
Ru9—Al14—Ru4	128.03 (3)	Al28A^i^—Al27—Al28A^xxiv^	180.00 (7)
Ru9^iii^—Al14—Ru4	128.03 (3)	Al28A^iii^—Al28A—Al28B	67.3 (6)
Ru9—Al14—Si6^xii^	107.23 (3)	Al28A^iii^—Al28A—Al28C	76.1 (3)
Ru9^iii^—Al14—Si6^xii^	107.23 (3)	Al28B—Al28A—Al28C	9.6 (7)
Ru4—Al14—Si6^xii^	107.28 (4)	Al28A^iii^—Al28A—Si17B	79.04 (15)
Ru9—Al14—Al25	65.59 (2)	Al28B—Al28A—Si17B	133.7 (9)
Ru9^iii^—Al14—Al25	104.13 (4)	Al28C—Al28A—Si17B	142.3 (8)
Ru4—Al14—Al25	63.62 (3)	Al28A^iii^—Al28A—Ru5^i^	81.43 (6)
Si6^xii^—Al14—Al25	144.44 (3)	Al28B—Al28A—Ru5^i^	68.4 (9)
Ru9—Al14—Al25^iii^	104.13 (4)	Al28C—Al28A—Ru5^i^	65.1 (8)
Ru9^iii^—Al14—Al25^iii^	65.60 (2)	Si17B—Al28A—Ru5^i^	137.7 (4)
Ru4—Al14—Al25^iii^	63.62 (3)	Al28A^iii^—Al28A—Al17A	81.59 (5)
Si6^xii^—Al14—Al25^iii^	144.44 (3)	Al28B—Al28A—Al17A	132.4 (9)
Al25—Al14—Al25^iii^	65.56 (5)	Al28C—Al28A—Al17A	140.4 (8)
Ru9—Al14—Al23^ix^	110.764 (16)	Si17B—Al28A—Al17A	5.0 (4)
Ru9^iii^—Al14—Al23^ix^	168.52 (4)	Ru5^i^—Al28A—Al17A	142.71 (9)
Ru4—Al14—Al23^ix^	57.15 (2)	Al28A^iii^—Al28A—Al25^v^	140.30 (5)
Si6^xii^—Al14—Al23^ix^	61.80 (3)	Al28B—Al28A—Al25^v^	113.0 (7)
Al25—Al14—Al23^ix^	87.35 (3)	Al28C—Al28A—Al25^v^	108.9 (6)
Al25^iii^—Al14—Al23^ix^	120.77 (4)	Si17B—Al28A—Al25^v^	74.4 (3)
Ru9—Al14—Al23^viii^	168.52 (4)	Ru5^i^—Al28A—Al25^v^	137.33 (10)
Ru9^iii^—Al14—Al23^viii^	110.764 (16)	Al17A—Al28A—Al25^v^	70.04 (6)
Ru4—Al14—Al23^viii^	57.15 (2)	Al28A^iii^—Al28A—Ru3	81.66 (5)
Si6^xii^—Al14—Al23^viii^	61.80 (3)	Al28B—Al28A—Ru3	80.5 (9)
Al25—Al14—Al23^viii^	120.77 (4)	Al28C—Al28A—Ru3	85.1 (9)
Al25^iii^—Al14—Al23^viii^	87.35 (3)	Si17B—Al28A—Ru3	63.5 (4)
Al23^ix^—Al14—Al23^viii^	62.02 (4)	Ru5^i^—Al28A—Ru3	148.43 (9)
Ru9—Al14—Al15^i^	58.96 (2)	Al17A—Al28A—Ru3	59.38 (5)
Ru9^iii^—Al14—Al15^i^	58.95 (2)	Al25^v^—Al28A—Ru3	60.26 (5)
Ru4—Al14—Al15^i^	169.63 (4)	Al28A^iii^—Al28A—Al22^i^	113.62 (5)
Si6^xii^—Al14—Al15^i^	62.36 (3)	Al28B—Al28A—Al22^i^	127.4 (10)
Al25—Al14—Al15^i^	124.44 (3)	Al28C—Al28A—Al22^i^	121.4 (8)
Al25^iii^—Al14—Al15^i^	124.44 (3)	Si17B—Al28A—Al22^i^	94.4 (3)
Al23^ix^—Al14—Al15^i^	114.47 (4)	Ru5^i^—Al28A—Al22^i^	60.36 (4)
Al23^viii^—Al14—Al15^i^	114.46 (4)	Al17A—Al28A—Al22^i^	97.35 (7)
Ru9—Al14—Al22^viii^	115.76 (3)	Al25^v^—Al28A—Al22^i^	97.47 (8)
Ru9^iii^—Al14—Al22^viii^	55.318 (17)	Ru3—Al28A—Al22^i^	151.18 (9)
Ru4—Al14—Al22^viii^	114.64 (2)	Al28A^iii^—Al28A—Al27^xxix^	82.37 (5)
Si6^xii^—Al14—Al22^viii^	61.01 (2)	Al28B—Al28A—Al27^xxix^	23.5 (9)
Al25—Al14—Al22^viii^	154.49 (4)	Al28C—Al28A—Al27^xxix^	15.9 (8)
Al25^iii^—Al14—Al22^viii^	90.42 (3)	Si17B—Al28A—Al27^xxix^	157.0 (2)
Al23^ix^—Al14—Al22^viii^	113.65 (4)	Ru5^i^—Al28A—Al27^xxix^	50.81 (4)
Al23^viii^—Al14—Al22^viii^	63.22 (2)	Al17A—Al28A—Al27^xxix^	155.92 (9)
Al15^i^—Al14—Al22^viii^	61.44 (2)	Al25^v^—Al28A—Al27^xxix^	113.47 (8)
Ru9—Al14—Al22^ix^	55.319 (17)	Ru3—Al28A—Al27^xxix^	100.54 (6)
Ru9^iii^—Al14—Al22^ix^	115.76 (3)	Al22^i^—Al28A—Al27^xxix^	105.50 (6)
Ru4—Al14—Al22^ix^	114.64 (2)	Al28C—Al28B—Al28A	155.5 (14)
Si6^xii^—Al14—Al22^ix^	61.01 (2)	Al28C—Al28B—Al28A^iii^	155.5 (14)
Al25—Al14—Al22^ix^	90.42 (3)	Al28A—Al28B—Al28A^iii^	45.5 (13)
Al25^iii^—Al14—Al22^ix^	154.49 (4)	Al28C—Al28B—Al27^xxix^	18 (2)
Al23^ix^—Al14—Al22^ix^	63.22 (2)	Al28A—Al28B—Al27^xxix^	145.2 (11)
Al23^viii^—Al14—Al22^ix^	113.65 (4)	Al28A^iii^—Al28B—Al27^xxix^	145.2 (11)
Al15^i^—Al14—Al22^ix^	61.44 (2)	Al28C—Al28B—Ru5^i^	82 (3)
Al22^viii^—Al14—Al22^ix^	111.52 (4)	Al28A—Al28B—Ru5^i^	88.9 (10)
Si17B—Al15—Ru9^iv^	63.07 (3)	Al28A^iii^—Al28B—Ru5^i^	88.9 (10)
Si17B—Al15—Ru9^v^	63.07 (3)	Al27^xxix^—Al28B—Ru5^i^	64.0 (6)
Ru9^iv^—Al15—Ru9^v^	125.94 (4)	Al28C—Al28B—Ru3	112 (3)
Si17B—Al15—Ru9^ii^	132.0 (4)	Al28A—Al28B—Ru3	78.0 (11)
Ru9^iv^—Al15—Ru9^ii^	76.250 (11)	Al28A^iii^—Al28B—Ru3	78.0 (11)
Ru9^v^—Al15—Ru9^ii^	144.13 (4)	Al27^xxix^—Al28B—Ru3	130.2 (10)
Si17B—Al15—Ru9^i^	132.0 (4)	Ru5^i^—Al28B—Ru3	165.8 (12)
Ru9^iv^—Al15—Ru9^i^	144.13 (4)	Al28C—Al28B—Al24^v^	66 (2)
Ru9^v^—Al15—Ru9^i^	76.250 (11)	Al28A—Al28B—Al24^v^	106.4 (7)
Ru9^ii^—Al15—Ru9^i^	72.07 (3)	Al28A^iii^—Al28B—Al24^v^	133.2 (14)
Si17B—Al15—Al17A	13.5 (5)	Al27^xxix^—Al28B—Al24^v^	80.8 (8)
Ru9^iv^—Al15—Al17A	64.43 (2)	Ru5^i^—Al28B—Al24^v^	133.1 (8)
Ru9^v^—Al15—Al17A	64.43 (2)	Ru3—Al28B—Al24^v^	57.5 (3)
Ru9^ii^—Al15—Al17A	139.19 (3)	Al28C—Al28B—Al24^iv^	66 (2)
Ru9^i^—Al15—Al17A	139.19 (3)	Al28A—Al28B—Al24^iv^	133.2 (14)
Si17B—Al15—Al14^i^	167.7 (6)	Al28A^iii^—Al28B—Al24^iv^	106.4 (7)
Ru9^iv^—Al15—Al14^i^	116.772 (19)	Al27^xxix^—Al28B—Al24^iv^	80.8 (8)
Ru9^v^—Al15—Al14^i^	116.772 (19)	Ru5^i^—Al28B—Al24^iv^	133.1 (8)
Ru9^ii^—Al15—Al14^i^	56.12 (2)	Ru3—Al28B—Al24^iv^	57.5 (3)
Ru9^i^—Al15—Al14^i^	56.12 (2)	Al24^v^—Al28B—Al24^iv^	62.9 (4)
Al17A—Al15—Al14^i^	154.18 (7)	Al28C—Al28B—Si17B	167 (3)
Si17B—Al15—Si6^xxvi^	111.0 (6)	Al28A—Al28B—Si17B	31.4 (8)
Ru9^iv^—Al15—Si6^xxvi^	101.37 (2)	Al28A^iii^—Al28B—Si17B	31.4 (8)
Ru9^v^—Al15—Si6^xxvi^	101.37 (2)	Al27^xxix^—Al28B—Si17B	175.2 (9)
Ru9^ii^—Al15—Si6^xxvi^	100.57 (3)	Ru5^i^—Al28B—Si17B	111.1 (9)
Ru9^i^—Al15—Si6^xxvi^	100.57 (3)	Ru3—Al28B—Si17B	54.6 (6)
Al17A—Al15—Si6^xxvi^	97.50 (6)	Al24^v^—Al28B—Si17B	103.3 (6)
Al14^i^—Al15—Si6^xxvi^	56.68 (3)	Al24^iv^—Al28B—Si17B	103.3 (6)
Si17B—Al15—Al22^ii^	78.3 (5)	Al28C—Al28B—Al23^ii^	74.0 (15)
Ru9^iv^—Al15—Al22^ii^	55.306 (19)	Al28A—Al28B—Al23^ii^	119.0 (11)
Ru9^v^—Al15—Al22^ii^	108.83 (3)	Al28A^iii^—Al28B—Al23^ii^	82.0 (5)
Ru9^ii^—Al15—Al22^ii^	57.28 (2)	Al27^xxix^—Al28B—Al23^ii^	65.0 (4)
Ru9^i^—Al15—Al22^ii^	92.78 (3)	Ru5^i^—Al28B—Al23^ii^	56.2 (3)
Al17A—Al15—Al22^ii^	89.97 (5)	Ru3—Al28B—Al23^ii^	126.0 (3)
Al14^i^—Al15—Al22^ii^	112.18 (4)	Al24^v^—Al28B—Al23^ii^	134.4 (9)
Si6^xxvi^—Al15—Al22^ii^	149.13 (2)	Al24^iv^—Al28B—Al23^ii^	81.8 (3)
Si17B—Al15—Al22^i^	78.3 (5)	Si17B—Al28B—Al23^ii^	112.8 (6)
Ru9^iv^—Al15—Al22^i^	108.83 (3)	Al28C—Al28B—Al23^i^	74.0 (15)
Ru9^v^—Al15—Al22^i^	55.306 (19)	Al28A—Al28B—Al23^i^	82.0 (5)
Ru9^ii^—Al15—Al22^i^	92.78 (3)	Al28A^iii^—Al28B—Al23^i^	119.0 (11)
Ru9^i^—Al15—Al22^i^	57.28 (2)	Al27^xxix^—Al28B—Al23^i^	65.0 (4)
Al17A—Al15—Al22^i^	89.97 (5)	Ru5^i^—Al28B—Al23^i^	56.2 (3)
Al14^i^—Al15—Al22^i^	112.18 (4)	Ru3—Al28B—Al23^i^	126.0 (3)
Si6^xxvi^—Al15—Al22^i^	149.13 (2)	Al24^v^—Al28B—Al23^i^	81.8 (3)
Al22^ii^—Al15—Al22^i^	60.09 (3)	Al24^iv^—Al28B—Al23^i^	134.4 (9)
Si17B—Al15—Al22^v^	114.0 (3)	Si17B—Al28B—Al23^i^	112.8 (6)
Ru9^iv^—Al15—Al22^v^	159.05 (4)	Al23^ii^—Al28B—Al23^i^	107.6 (6)
Ru9^v^—Al15—Al22^v^	57.609 (15)	Al28B—Al28C—Al27^xxix^	154 (4)
Ru9^ii^—Al15—Al22^v^	112.97 (3)	Al28B—Al28C—Al28A	14.9 (8)
Ru9^i^—Al15—Al22^v^	54.534 (18)	Al27^xxix^—Al28C—Al28A	146.0 (16)
Al17A—Al15—Al22^v^	107.73 (4)	Al28B—Al28C—Al28A^iii^	14.9 (8)
Al14^i^—Al15—Al22^v^	61.21 (2)	Al27^xxix^—Al28C—Al28A^iii^	146.0 (16)
Si6^xxvi^—Al15—Al22^v^	59.21 (2)	Al28A—Al28C—Al28A^iii^	27.7 (7)
Al22^ii^—Al15—Al22^v^	145.63 (3)	Al28B—Al28C—Ru5^i^	83 (3)
Al22^i^—Al15—Al22^v^	89.97 (2)	Al27^xxix^—Al28C—Ru5^i^	71.5 (10)
Si17B—Al15—Al22^iv^	114.0 (3)	Al28A—Al28C—Ru5^i^	77.5 (11)
Ru9^iv^—Al15—Al22^iv^	57.609 (15)	Al28A^iii^—Al28C—Ru5^i^	77.5 (11)
Ru9^v^—Al15—Al22^iv^	159.05 (4)	Al28B—Al28C—Al24^v^	101 (2)
Ru9^ii^—Al15—Al22^iv^	54.534 (18)	Al27^xxix^—Al28C—Al24^v^	100.7 (14)
Ru9^i^—Al15—Al22^iv^	112.97 (3)	Al28A—Al28C—Al24^v^	96.8 (10)
Al17A—Al15—Al22^iv^	107.73 (4)	Al28A^iii^—Al28C—Al24^v^	112.8 (14)
Al14^i^—Al15—Al22^iv^	61.21 (2)	Ru5^i^—Al28C—Al24^v^	145.4 (4)
Si6^xxvi^—Al15—Al22^iv^	59.21 (2)	Al28B—Al28C—Al24^iv^	101 (2)
Al22^ii^—Al15—Al22^iv^	89.97 (2)	Al27^xxix^—Al28C—Al24^iv^	100.7 (14)
Al22^i^—Al15—Al22^iv^	145.63 (3)	Al28A—Al28C—Al24^iv^	112.8 (14)
Al22^v^—Al15—Al22^iv^	111.16 (4)	Al28A^iii^—Al28C—Al24^iv^	96.8 (10)
Ru7B^xxv^—Al16—Ru7B^xi^	77.0 (6)	Ru5^i^—Al28C—Al24^iv^	145.4 (4)
Ru7B^xxv^—Al16—Ru4	136.1 (2)	Al24^v^—Al28C—Al24^iv^	68.4 (6)
Ru7B^xi^—Al16—Ru4	136.1 (2)	Al28B—Al28C—Al28C^vi^	154 (4)
Ru7B^xxv^—Al16—Ru7A^xxv^	4.1 (4)	Al27^xxix^—Al28C—Al28C^vi^	0.0 (8)
Ru7B^xi^—Al16—Ru7A^xxv^	79.3 (3)	Al28A—Al28C—Al28C^vi^	146.0 (16)
Ru4—Al16—Ru7A^xxv^	135.94 (4)	Al28A^iii^—Al28C—Al28C^vi^	146.0 (16)
Ru7B^xxv^—Al16—Ru7A^xi^	79.3 (3)	Ru5^i^—Al28C—Al28C^vi^	71.5 (10)
Ru7B^xi^—Al16—Ru7A^xi^	4.1 (4)	Al24^v^—Al28C—Al28C^vi^	100.7 (14)
Ru4—Al16—Ru7A^xi^	135.94 (4)	Al24^iv^—Al28C—Al28C^vi^	100.7 (14)
Ru7A^xxv^—Al16—Ru7A^xi^	81.43 (11)	Al28B—Al28C—Al23^ii^	93.7 (16)
Ru7B^xxv^—Al16—Al24	70.8 (3)	Al27^xxix^—Al28C—Al23^ii^	72.8 (7)
Ru7B^xi^—Al16—Al24	144.9 (4)	Al28A—Al28C—Al23^ii^	102.4 (11)
Ru4—Al16—Al24	67.90 (3)	Al28A^iii^—Al28C—Al23^ii^	79.2 (6)
Ru7A^xxv^—Al16—Al24	69.37 (4)	Ru5^i^—Al28C—Al23^ii^	58.0 (4)
Ru7A^xi^—Al16—Al24	148.30 (9)	Al24^v^—Al28C—Al23^ii^	153.6 (10)
Ru7B^xxv^—Al16—Al24^iii^	144.9 (4)	Al24^iv^—Al28C—Al23^ii^	87.4 (2)
Ru7B^xi^—Al16—Al24^iii^	70.8 (3)	Al28C^vi^—Al28C—Al23^ii^	72.8 (7)
Ru4—Al16—Al24^iii^	67.90 (3)	Al28B—Al28C—Al23^i^	93.7 (16)
Ru7A^xxv^—Al16—Al24^iii^	148.30 (9)	Al27^xxix^—Al28C—Al23^i^	72.8 (7)
Ru7A^xi^—Al16—Al24^iii^	69.37 (4)	Al28A—Al28C—Al23^i^	79.2 (6)
Al24—Al16—Al24^iii^	134.53 (5)	Al28A^iii^—Al28C—Al23^i^	102.4 (11)
Ru7B^xxv^—Al16—Si6	101.2 (5)	Ru5^i^—Al28C—Al23^i^	58.0 (4)
Ru7B^xi^—Al16—Si6	101.2 (5)	Al24^v^—Al28C—Al23^i^	87.4 (2)
Ru4—Al16—Si6	98.54 (4)	Al24^iv^—Al28C—Al23^i^	153.6 (10)
Ru7A^xxv^—Al16—Si6	97.56 (9)	Al28C^vi^—Al28C—Al23^i^	72.8 (7)
Ru7A^xi^—Al16—Si6	97.56 (9)	Al23^ii^—Al28C—Al23^i^	113.7 (8)
Al24—Al16—Si6	98.22 (3)	Al28B—Al28C—Ru3	56 (3)
Al24^iii^—Al16—Si6	98.22 (3)	Al27^xxix^—Al28C—Ru3	149.8 (17)
Ru7B^xxv^—Al16—Al21^x^	57.5 (5)	Al28A—Al28C—Ru3	62.5 (10)
Ru7B^xi^—Al16—Al21^x^	114.2 (3)	Al28A^iii^—Al28C—Ru3	62.5 (10)
Ru4—Al16—Al21^x^	109.03 (3)	Ru5^i^—Al28C—Ru3	138.7 (15)
Ru7A^xxv^—Al16—Al21^x^	53.48 (9)	Al24^v^—Al28C—Ru3	55.5 (5)
Ru7A^xi^—Al16—Al21^x^	113.39 (5)	Al24^iv^—Al28C—Ru3	55.5 (5)
Al24—Al16—Al21^x^	58.04 (2)	Al28C^vi^—Al28C—Ru3	149.8 (17)
Al24^iii^—Al16—Al21^x^	150.87 (5)	Al23^ii^—Al28C—Ru3	119.1 (7)
Si6—Al16—Al21^x^	52.86 (3)	Al23^i^—Al28C—Ru3	119.1 (7)
Ru7B^xxv^—Al16—Al21^xi^	114.2 (3)	Al28B—Al28C—Ru5^xxix^	159 (3)
Ru7B^xi^—Al16—Al21^xi^	57.5 (5)	Al27^xxix^—Al28C—Ru5^xxix^	47.4 (10)
Ru4—Al16—Al21^xi^	109.03 (3)	Al28A—Al28C—Ru5^xxix^	158.9 (10)
Ru7A^xxv^—Al16—Al21^xi^	113.39 (5)	Al28A^iii^—Al28C—Ru5^xxix^	158.9 (10)
Ru7A^xi^—Al16—Al21^xi^	53.48 (9)	Ru5^i^—Al28C—Ru5^xxix^	118.9 (13)
Al24—Al16—Al21^xi^	150.87 (5)	Al24^v^—Al28C—Ru5^xxix^	62.1 (7)
Al24^iii^—Al16—Al21^xi^	58.04 (2)	Al24^iv^—Al28C—Ru5^xxix^	62.1 (7)
Si6—Al16—Al21^xi^	52.86 (3)	Al28C^vi^—Al28C—Ru5^xxix^	47.4 (10)
Al21^x^—Al16—Al21^xi^	99.01 (5)	Al23^ii^—Al28C—Ru5^xxix^	97.9 (8)
Si17B—Al17A—Ru3	74.0 (11)	Al23^i^—Al28C—Ru5^xxix^	97.9 (8)
Si17B—Al17A—Al28A^iii^	16.0 (9)	Ru3—Al28C—Ru5^xxix^	102.3 (7)

Symmetry codes: (i) *x*, *y*, −*z*+1/2; (ii) −*x*, *y*, −*z*+1/2; (iii) −*x*, *y*, *z*; (iv) *x*−1/2, −*y*+1/2, *z*+1/2; (v) −*x*+1/2, −*y*+1/2, *z*+1/2; (vi) −*x*, −*y*, −*z*+1; (vii) −*x*+1/2, −*y*+1/2, −*z*+1; (viii) *x*−1/2, −*y*+1/2, −*z*; (ix) −*x*+1/2, −*y*+1/2, −*z*; (x) −*x*+1/2, *y*+1/2, *z*; (xi) *x*−1/2, *y*+1/2, *z*; (xii) −*x*, −*y*+1, −*z*; (xiii) −*x*, −*y*+1, *z*−1/2; (xiv) *x*+1/2, *y*−1/2, −*z*+1/2; (xv) *x*+1/2, *y*−1/2, *z*; (xvi) −*x*+1/2, *y*−1/2, −*z*+1/2; (xvii) −*x*+1/2, *y*−1/2, *z*; (xviii) −*x*+1, *y*, −*z*+1/2; (xix) −*x*+1, *y*, *z*; (xx) −*x*+1/2, −*y*+1/2, *z*−1/2; (xxi) *x*−1/2, *y*−1/2, *z*; (xxii) *x*−1/2, *y*−1/2, −*z*+1/2; (xxiii) *x*, −*y*, −*z*; (xxiv) −*x*, −*y*, *z*−1/2; (xxv) −*x*+1/2, *y*+1/2, −*z*+1/2; (xxvi) −*x*, −*y*+1, *z*+1/2; (xxvii) *x*+1/2, *y*+1/2, *z*; (xxviii) −*x*, −*y*, −*z*; (xxix) −*x*, −*y*, *z*+1/2.

### Docosaaluminium nonaruthenium decasilicon (II) Crystal data

**Table:**

~Ru_9_(Al_0.70_Si_0.30_)_32_	*D*_x_ = 4.777 Mg m^−^^3^
*M**_r_* = 1783.65	Mo *K*α radiation, λ = 0.71073 Å
Trigonal, *R*3	Cell parameters from 10605 reflections
*a* = 10.4479 (2) Å	θ = 3.9–30.7°
*c* = 19.6774 (4) Å	µ = 6.59 mm^−^^1^
*V* = 1860.17 (7) Å^3^	*T* = 296 K
*Z* = 3	Irregular, metallic light silver
*F*(000) = 2436.84	0.03 × 0.03 × 0.03 mm

### Docosaaluminium nonaruthenium decasilicon (II) Data collection

**Table:**

XtaLAB Synergy R, HyPix diffractometer	1193 independent reflections
Radiation source: micro-focus sealed X-ray tube, Mova (Mo) X-ray Source	1127 reflections with *I* > 2σ(*I*)
Mirror monochromator	*R*_int_ = 0.027
Detector resolution: 10.0000 pixels mm^-1^	θ_max_ = 30.8°, θ_min_ = 3.9°
ω scans	*h* = −14→14
Absorption correction: gaussian (CrysAlisPro; Matsumoto *et al.*, 2021)	*k* = −14→14
*T*_min_ = 0.868, *T*_max_ = 0.879	*l* = −27→28
14255 measured reflections	

### Docosaaluminium nonaruthenium decasilicon (II) Refinement

**Table:**

Refinement on *F*^2^	Primary atom site location: dual
Least-squares matrix: full	*w* = 1/[σ^2^(*F*_o_^2^) + (0.0111*P*)^2^ + 0.5812*P*] where *P* = (*F*_o_^2^ + 2*F*_c_^2^)/3
*R*[*F*^2^ > 2σ(*F*^2^)] = 0.013	(Δ/σ)_max_ = 0.002
*wR*(*F*^2^) = 0.024	Δρ_max_ = 0.45 e Å^−^^3^
*S* = 1.08	Δρ_min_ = −0.53 e Å^−^^3^
1193 reflections	Extinction correction: SHELXL-2019/3 (Sheldrick, 2015b), Fc^*^=kFc[1+0.001xFc^2^λ^3^/sin(2θ)]^-1/4^
66 parameters	Extinction coefficient: 0.000262 (16)
2 restraints	

### Docosaaluminium nonaruthenium decasilicon (II) Special details

**Table:**

Geometry. All esds (except the esd in the dihedral angle between two l.s. planes) are estimated using the full covariance matrix. The cell esds are taken into account individually in the estimation of esds in distances, angles and torsion angles; correlations between esds in cell parameters are only used when they are defined by crystal symmetry. An approximate (isotropic) treatment of cell esds is used for estimating esds involving l.s. planes.

### Docosaaluminium nonaruthenium decasilicon (II) Fractional atomic coordinates and isotropic or equivalent isotropic displacement parameters (Å^2^)

**Table:**

	*x*	*y*	*z*	*U*_iso_*/*U*_eq_	Occ. (<1)
Ru1	0.000000	0.000000	0.500000	0.00463 (7)	
Ru2	0.000000	0.000000	0.28833 (2)	0.00519 (6)	
Ru3	0.24368 (2)	0.24916 (2)	0.09171 (2)	0.00467 (5)	
Al4	0.000000	0.000000	0.12554 (5)	0.01068 (17)	
Si5	0.06845 (5)	0.25394 (5)	0.29541 (2)	0.00880 (9)	
Al6	0.22915 (5)	0.16512 (5)	0.21432 (2)	0.0079 (2)	0.58 (4)
Si6	0.22915 (5)	0.16512 (5)	0.21432 (2)	0.0079 (2)	0.42 (4)
Al7	0.00143 (5)	0.21088 (5)	0.03958 (2)	0.0076 (2)	0.82 (4)
Si7	0.00143 (5)	0.21088 (5)	0.03958 (2)	0.0076 (2)	0.18 (4)
Al8	0.16504 (5)	0.12448 (5)	0.39021 (2)	0.00948 (10)	
Al9	0.12627 (6)	0.37148 (6)	0.16275 (2)	0.01084 (11)	

### Docosaaluminium nonaruthenium decasilicon (II) Atomic displacement parameters (Å^2^)

**Table:**

	*U*^11^	*U*^22^	*U*^33^	*U*^12^	*U*^13^	*U*^23^
Ru1	0.00464 (9)	0.00464 (9)	0.00459 (13)	0.00232 (5)	0.000	0.000
Ru2	0.00444 (7)	0.00444 (7)	0.00667 (10)	0.00222 (4)	0.000	0.000
Ru3	0.00486 (7)	0.00394 (7)	0.00529 (7)	0.00224 (5)	0.00054 (4)	0.00006 (4)
Al4	0.0066 (2)	0.0066 (2)	0.0189 (4)	0.00329 (12)	0.000	0.000
Si5	0.0109 (2)	0.0054 (2)	0.0101 (2)	0.00405 (18)	−0.00401 (16)	−0.00003 (16)
Al6	0.0076 (3)	0.0096 (3)	0.0073 (3)	0.0048 (2)	0.00127 (16)	0.00249 (17)
Si6	0.0076 (3)	0.0096 (3)	0.0073 (3)	0.0048 (2)	0.00127 (16)	0.00249 (17)
Al7	0.0059 (3)	0.0102 (3)	0.0061 (3)	0.0036 (2)	0.00001 (16)	−0.00019 (17)
Si7	0.0059 (3)	0.0102 (3)	0.0061 (3)	0.0036 (2)	0.00001 (16)	−0.00019 (17)
Al8	0.0074 (2)	0.0088 (2)	0.0076 (2)	0.00063 (19)	−0.00180 (18)	−0.00055 (17)
Al9	0.0098 (2)	0.0097 (2)	0.0121 (2)	0.0042 (2)	0.00506 (18)	−0.00117 (19)

### Docosaaluminium nonaruthenium decasilicon (II) Geometric parameters (Å, º)

**Table:**

Ru1—Al8^i^	2.6625 (5)	Ru3—Al7^xv^	2.6119 (5)
Ru1—Al8^ii^	2.6625 (5)	Ru3—Al4	2.6597 (3)
Ru1—Al8^iii^	2.6625 (5)	Ru3—Al7^ii^	2.6643 (5)
Ru1—Al8^iv^	2.6626 (5)	Al4—Al6^iv^	2.7622 (7)
Ru1—Al8^v^	2.6626 (5)	Al4—Al6^ii^	2.7622 (7)
Ru1—Al8	2.6626 (5)	Al4—Al6	2.7622 (7)
Ru1—Al9^vi^	2.7432 (5)	Al4—Al7	2.7718 (7)
Ru1—Al9^vii^	2.7432 (5)	Al4—Al7^iv^	2.7718 (7)
Ru1—Al9^viii^	2.7432 (5)	Al4—Al7^ii^	2.7718 (7)
Ru1—Al9^ix^	2.7432 (5)	Si5—Si5^ix^	2.4056 (9)
Ru1—Al9^x^	2.7433 (5)	Si5—Al8^ix^	2.6749 (6)
Ru1—Al9^xi^	2.7433 (5)	Si5—Al6^iv^	2.7560 (6)
Ru2—Si5^ii^	2.3817 (4)	Si5—Al8	2.7736 (6)
Ru2—Si5	2.3817 (4)	Si5—Al6	2.7899 (6)
Ru2—Si5^iv^	2.3817 (4)	Si5—Al9	2.8188 (7)
Ru2—Al8	2.5380 (5)	Si5—Al8^iv^	2.8319 (7)
Ru2—Al8^ii^	2.5380 (5)	Al6—Al7^xii^	2.6531 (6)
Ru2—Al8^iv^	2.5381 (5)	Al6—Al9^xii^	2.8394 (7)
Ru2—Al6^ii^	2.5880 (5)	Al6—Al6^xiv^	2.8614 (9)
Ru2—Al6^iv^	2.5880 (5)	Al7—Al7^xvi^	2.6922 (6)
Ru2—Al6	2.5881 (5)	Al7—Al7^xv^	2.6922 (6)
Ru3—Si5^xii^	2.4214 (4)	Al7—Al9	2.8639 (7)
Ru3—Al8^xiii^	2.5175 (5)	Al8—Al9^vii^	2.6172 (7)
Ru3—Al6	2.5459 (5)	Al8—Al8^ii^	2.6956 (8)
Ru3—Al7	2.5702 (5)	Al8—Al8^iv^	2.6957 (8)
Ru3—Al9^xii^	2.5708 (5)	Al8—Al9^ix^	2.7562 (7)
Ru3—Al9	2.5806 (5)	Al9—Al9^xii^	2.7465 (5)
Ru3—Al6^xiv^	2.6110 (5)	Al9—Al9^xvii^	2.7465 (5)
			
Al8^i^—Ru1—Al8^ii^	180.0	Si5^ix^—Si5—Al8^ix^	65.93 (2)
Al8^i^—Ru1—Al8^iii^	60.824 (17)	Ru3^xvii^—Si5—Al8^ix^	58.956 (14)
Al8^ii^—Ru1—Al8^iii^	119.176 (17)	Ru2—Si5—Al6^iv^	59.969 (14)
Al8^i^—Ru1—Al8^iv^	119.176 (17)	Si5^ix^—Si5—Al6^iv^	177.02 (3)
Al8^ii^—Ru1—Al8^iv^	60.824 (17)	Ru3^xvii^—Si5—Al6^iv^	60.159 (14)
Al8^iii^—Ru1—Al8^iv^	119.176 (17)	Al8^ix^—Si5—Al6^iv^	115.87 (2)
Al8^i^—Ru1—Al8^v^	60.824 (17)	Ru2—Si5—Al8	58.394 (14)
Al8^ii^—Ru1—Al8^v^	119.176 (17)	Si5^ix^—Si5—Al8	61.71 (2)
Al8^iii^—Ru1—Al8^v^	60.824 (17)	Ru3^xvii^—Si5—Al8	159.94 (2)
Al8^iv^—Ru1—Al8^v^	180.000 (12)	Al8^ix^—Si5—Al8	127.637 (19)
Al8^i^—Ru1—Al8	119.177 (17)	Al6^iv^—Si5—Al8	116.45 (2)
Al8^ii^—Ru1—Al8	60.823 (17)	Ru2—Si5—Al6	59.434 (14)
Al8^iii^—Ru1—Al8	179.999 (12)	Si5^ix^—Si5—Al6	97.84 (3)
Al8^iv^—Ru1—Al8	60.824 (17)	Ru3^xvii^—Si5—Al6	119.201 (19)
Al8^v^—Ru1—Al8	119.176 (17)	Al8^ix^—Si5—Al6	109.51 (2)
Al8^i^—Ru1—Al9^vi^	89.014 (15)	Al6^iv^—Si5—Al6	83.85 (2)
Al8^ii^—Ru1—Al9^vi^	90.986 (15)	Al8—Si5—Al6	78.242 (18)
Al8^iii^—Ru1—Al9^vi^	57.891 (14)	Ru2—Si5—Al9	108.102 (19)
Al8^iv^—Ru1—Al9^vi^	61.286 (14)	Si5^ix^—Si5—Al9	110.55 (3)
Al8^v^—Ru1—Al9^vi^	118.714 (14)	Ru3^xvii^—Si5—Al9	58.171 (14)
Al8—Ru1—Al9^vi^	122.110 (14)	Al8^ix^—Si5—Al9	60.157 (17)
Al8^i^—Ru1—Al9^vii^	90.986 (15)	Al6^iv^—Si5—Al9	72.378 (18)
Al8^ii^—Ru1—Al9^vii^	89.014 (15)	Al8—Si5—Al9	141.66 (2)
Al8^iii^—Ru1—Al9^vii^	122.109 (14)	Al6—Si5—Al9	65.278 (17)
Al8^iv^—Ru1—Al9^vii^	118.714 (14)	Ru2—Si5—Al8^iv^	57.488 (14)
Al8^v^—Ru1—Al9^vii^	61.286 (14)	Si5^ix^—Si5—Al8^iv^	99.22 (3)
Al8—Ru1—Al9^vii^	57.890 (14)	Ru3^xvii^—Si5—Al8^iv^	103.669 (19)
Al9^vi^—Ru1—Al9^vii^	180.0	Al8^ix^—Si5—Al8^iv^	134.55 (2)
Al8^i^—Ru1—Al9^viii^	122.109 (14)	Al6^iv^—Si5—Al8^iv^	77.825 (18)
Al8^ii^—Ru1—Al9^viii^	57.891 (14)	Al8—Si5—Al8^iv^	57.48 (2)
Al8^iii^—Ru1—Al9^viii^	61.285 (14)	Al6—Si5—Al8^iv^	115.13 (2)
Al8^iv^—Ru1—Al9^viii^	89.013 (15)	Al9—Si5—Al8^iv^	150.02 (2)
Al8^v^—Ru1—Al9^viii^	90.987 (15)	Ru3—Al6—Ru2	129.707 (19)
Al8—Ru1—Al9^viii^	118.714 (14)	Ru3—Al6—Ru3^xiv^	112.612 (17)
Al9^vi^—Ru1—Al9^viii^	60.077 (1)	Ru2—Al6—Ru3^xiv^	106.091 (16)
Al9^vii^—Ru1—Al9^viii^	119.923 (1)	Ru3—Al6—Al7^xii^	116.69 (2)
Al8^i^—Ru1—Al9^ix^	57.891 (14)	Ru2—Al6—Al7^xii^	109.655 (19)
Al8^ii^—Ru1—Al9^ix^	122.109 (14)	Ru3^xiv^—Al6—Al7^xii^	60.806 (14)
Al8^iii^—Ru1—Al9^ix^	118.715 (14)	Ru3—Al6—Si5^ii^	143.99 (2)
Al8^iv^—Ru1—Al9^ix^	90.987 (15)	Ru2—Al6—Si5^ii^	52.818 (13)
Al8^v^—Ru1—Al9^ix^	89.013 (15)	Ru3^xiv^—Al6—Si5^ii^	53.554 (13)
Al8—Ru1—Al9^ix^	61.286 (14)	Al7^xii^—Al6—Si5^ii^	86.919 (19)
Al9^vi^—Ru1—Al9^ix^	119.923 (1)	Ru3—Al6—Al4	59.976 (17)
Al9^vii^—Ru1—Al9^ix^	60.077 (1)	Ru2—Al6—Al4	73.471 (19)
Al9^viii^—Ru1—Al9^ix^	180.0	Ru3^xiv^—Al6—Al4	119.885 (18)
Al8^i^—Ru1—Al9^x^	118.713 (14)	Al7^xii^—Al6—Al4	176.66 (3)
Al8^ii^—Ru1—Al9^x^	61.287 (14)	Si5^ii^—Al6—Al4	96.03 (2)
Al8^iii^—Ru1—Al9^x^	89.012 (15)	Ru3—Al6—Si5	111.671 (19)
Al8^iv^—Ru1—Al9^x^	122.111 (14)	Ru2—Al6—Si5	52.410 (13)
Al8^v^—Ru1—Al9^x^	57.889 (14)	Ru3^xiv^—Al6—Si5	133.10 (2)
Al8—Ru1—Al9^x^	90.987 (15)	Al7^xii^—Al6—Si5	85.969 (18)
Al9^vi^—Ru1—Al9^x^	119.921 (1)	Si5^ii^—Al6—Si5	95.90 (2)
Al9^vii^—Ru1—Al9^x^	60.079 (1)	Al4—Al6—Si5	95.257 (19)
Al9^viii^—Ru1—Al9^x^	60.079 (1)	Ru3—Al6—Al9^xii^	56.713 (13)
Al9^ix^—Ru1—Al9^x^	119.921 (1)	Ru2—Al6—Al9^xii^	144.13 (2)
Al8^i^—Ru1—Al9^xi^	61.287 (14)	Ru3^xiv^—Al6—Al9^xii^	99.795 (18)
Al8^ii^—Ru1—Al9^xi^	118.713 (14)	Al7^xii^—Al6—Al9^xii^	62.748 (17)
Al8^iii^—Ru1—Al9^xi^	90.988 (15)	Si5^ii^—Al6—Al9^xii^	148.12 (2)
Al8^iv^—Ru1—Al9^xi^	57.889 (14)	Al4—Al6—Al9^xii^	114.07 (2)
Al8^v^—Ru1—Al9^xi^	122.111 (14)	Si5—Al6—Al9^xii^	91.741 (19)
Al8—Ru1—Al9^xi^	89.013 (15)	Ru3—Al6—Al6^xiv^	57.391 (15)
Al9^vi^—Ru1—Al9^xi^	60.079 (1)	Ru2—Al6—Al6^xiv^	145.22 (3)
Al9^vii^—Ru1—Al9^xi^	119.921 (1)	Ru3^xiv^—Al6—Al6^xiv^	55.221 (15)
Al9^viii^—Ru1—Al9^xi^	119.921 (1)	Al7^xii^—Al6—Al6^xiv^	87.39 (2)
Al9^ix^—Ru1—Al9^xi^	60.079 (1)	Si5^ii^—Al6—Al6^xiv^	100.23 (2)
Al9^x^—Ru1—Al9^xi^	180.0	Al4—Al6—Al6^xiv^	90.54 (2)
Si5^ii^—Ru2—Si5	119.661 (2)	Si5—Al6—Al6^xiv^	162.19 (3)
Si5^ii^—Ru2—Si5^iv^	119.662 (2)	Al9^xii^—Al6—Al6^xiv^	70.54 (2)
Si5—Ru2—Si5^iv^	119.659 (2)	Ru3—Al7—Ru3^xvi^	117.649 (18)
Si5^ii^—Ru2—Al8	70.202 (16)	Ru3—Al7—Al6^xvii^	116.63 (2)
Si5—Ru2—Al8	68.549 (15)	Ru3^xvi^—Al7—Al6^xvii^	119.15 (2)
Si5^iv^—Ru2—Al8	124.453 (17)	Ru3—Al7—Ru3^iv^	116.862 (18)
Si5^ii^—Ru2—Al8^ii^	68.549 (15)	Ru3^xvi^—Al7—Ru3^iv^	114.346 (17)
Si5—Ru2—Al8^ii^	124.452 (17)	Al6^xvii^—Al7—Ru3^iv^	58.818 (14)
Si5^iv^—Ru2—Al8^ii^	70.202 (16)	Ru3—Al7—Al7^xvi^	134.381 (13)
Al8—Ru2—Al8^ii^	64.153 (18)	Ru3^xvi^—Al7—Al7^xvi^	57.944 (19)
Si5^ii^—Ru2—Al8^iv^	124.453 (17)	Al6^xvii^—Al7—Al7^xvi^	99.12 (2)
Si5—Ru2—Al8^iv^	70.202 (16)	Ru3^iv^—Al7—Al7^xvi^	58.365 (12)
Si5^iv^—Ru2—Al8^iv^	68.548 (15)	Ru3—Al7—Al7^xv^	59.461 (12)
Al8—Ru2—Al8^iv^	64.153 (18)	Ru3^xvi^—Al7—Al7^xv^	60.283 (19)
Al8^ii^—Ru2—Al8^iv^	64.152 (18)	Al6^xvii^—Al7—Al7^xv^	168.67 (2)
Si5^ii^—Ru2—Al6^ii^	68.158 (15)	Ru3^iv^—Al7—Al7^xv^	132.432 (13)
Si5—Ru2—Al6^ii^	149.111 (17)	Al7^xvi^—Al7—Al7^xv^	89.88 (2)
Si5^iv^—Ru2—Al6^ii^	67.213 (15)	Ru3—Al7—Al4	59.578 (10)
Al8—Ru2—Al6^ii^	135.530 (15)	Ru3^xvi^—Al7—Al4	135.92 (2)
Al8^ii^—Ru2—Al6^ii^	86.437 (15)	Al6^xvii^—Al7—Al4	94.86 (2)
Al8^iv^—Ru2—Al6^ii^	132.991 (15)	Ru3^iv^—Al7—Al4	58.545 (10)
Si5^ii^—Ru2—Al6^iv^	149.112 (17)	Al7^xvi^—Al7—Al4	91.721 (15)
Si5—Ru2—Al6^iv^	67.211 (15)	Al7^xv^—Al7—Al4	91.721 (15)
Si5^iv^—Ru2—Al6^iv^	68.157 (15)	Ru3—Al7—Al9	56.394 (14)
Al8—Ru2—Al6^iv^	132.990 (15)	Ru3^xvi^—Al7—Al9	142.61 (2)
Al8^ii^—Ru2—Al6^iv^	135.529 (15)	Al6^xvii^—Al7—Al9	61.810 (17)
Al8^iv^—Ru2—Al6^iv^	86.436 (15)	Ru3^iv^—Al7—Al9	97.917 (17)
Al6^ii^—Ru2—Al6^iv^	91.441 (15)	Al7^xvi^—Al7—Al9	156.03 (3)
Si5^ii^—Ru2—Al6	67.213 (15)	Al7^xv^—Al7—Al9	111.07 (2)
Si5—Ru2—Al6	68.157 (15)	Al4—Al7—Al9	76.65 (2)
Si5^iv^—Ru2—Al6	149.112 (17)	Ru3^vii^—Al8—Ru2	143.54 (2)
Al8—Ru2—Al6	86.435 (15)	Ru3^vii^—Al8—Al9^vii^	60.306 (15)
Al8^ii^—Ru2—Al6	132.991 (15)	Ru2—Al8—Al9^vii^	145.45 (2)
Al8^iv^—Ru2—Al6	135.529 (15)	Ru3^vii^—Al8—Ru1	109.918 (17)
Al6^ii^—Ru2—Al6	91.442 (15)	Ru2—Al8—Ru1	106.409 (17)
Al6^iv^—Ru2—Al6	91.441 (15)	Al9^vii^—Al8—Ru1	62.599 (15)
Si5^xii^—Ru3—Al8^xiii^	65.551 (15)	Ru3^vii^—Al8—Si5^ix^	55.492 (14)
Si5^xii^—Ru3—Al6	120.406 (15)	Ru2—Al8—Si5^ix^	104.439 (19)
Al8^xiii^—Ru3—Al6	124.414 (16)	Al9^vii^—Al8—Si5^ix^	109.63 (2)
Si5^xii^—Ru3—Al7	122.217 (15)	Ru1—Al8—Si5^ix^	117.12 (2)
Al8^xiii^—Ru3—Al7	73.932 (16)	Ru3^vii^—Al8—Al8^ii^	149.08 (3)
Al6—Ru3—Al7	116.122 (15)	Ru2—Al8—Al8^ii^	57.924 (9)
Si5^xii^—Ru3—Al9^xii^	68.678 (16)	Al9^vii^—Al8—Al8^ii^	91.00 (2)
Al8^xiii^—Ru3—Al9^xii^	65.586 (16)	Ru1—Al8—Al8^ii^	59.587 (8)
Al6—Ru3—Al9^xii^	67.410 (16)	Si5^ix^—Al8—Al8^ii^	155.06 (3)
Al7—Ru3—Al9^xii^	127.455 (16)	Ru3^vii^—Al8—Al8^iv^	143.73 (3)
Si5^xii^—Ru3—Al9	119.665 (16)	Ru2—Al8—Al8^iv^	57.925 (9)
Al8^xiii^—Ru3—Al9	61.760 (16)	Al9^vii^—Al8—Al8^iv^	122.187 (15)
Al6—Ru3—Al9	72.324 (16)	Ru1—Al8—Al8^iv^	59.588 (8)
Al7—Ru3—Al9	67.560 (16)	Si5^ix^—Al8—Al8^iv^	96.24 (3)
Al9^xii^—Ru3—Al9	64.436 (17)	Al8^ii^—Al8—Al8^iv^	60.0
Si5^xii^—Ru3—Al6^xiv^	66.289 (14)	Ru3^vii^—Al8—Al9^ix^	58.136 (15)
Al8^xiii^—Ru3—Al6^xiv^	127.640 (16)	Ru2—Al8—Al9^ix^	145.13 (2)
Al6—Ru3—Al6^xiv^	67.389 (17)	Al9^vii^—Al8—Al9^ix^	61.411 (12)
Al7—Ru3—Al6^xiv^	153.459 (16)	Ru1—Al8—Al9^ix^	60.799 (14)
Al9^xii^—Ru3—Al6^xiv^	78.881 (16)	Si5^ix^—Al8—Al9^ix^	62.510 (18)
Al9—Ru3—Al6^xiv^	133.370 (16)	Al8^ii^—Al8—Al9^ix^	120.386 (14)
Si5^xii^—Ru3—Al7^xv^	71.465 (15)	Al8^iv^—Al8—Al9^ix^	90.01 (2)
Al8^xiii^—Ru3—Al7^xv^	80.729 (15)	Ru3^vii^—Al8—Si5	104.754 (19)
Al6—Ru3—Al7^xv^	154.440 (16)	Ru2—Al8—Si5	53.057 (13)
Al7—Ru3—Al7^xv^	62.594 (14)	Al9^vii^—Al8—Si5	161.49 (2)
Al9^xii^—Ru3—Al7^xv^	135.585 (16)	Ru1—Al8—Si5	118.64 (2)
Al9—Ru3—Al7^xv^	123.870 (16)	Si5^ix^—Al8—Si5	52.363 (19)
Al6^xiv^—Ru3—Al7^xv^	102.325 (15)	Al8^ii^—Al8—Si5	105.493 (16)
Si5^xii^—Ru3—Al4	156.665 (16)	Al8^iv^—Al8—Si5	62.348 (19)
Al8^xiii^—Ru3—Al4	133.431 (13)	Al9^ix^—Al8—Si5	102.09 (2)
Al6—Ru3—Al4	64.05 (2)	Ru3^vii^—Al8—Si5^ii^	111.11 (2)
Al7—Ru3—Al4	63.982 (15)	Ru2—Al8—Si5^ii^	52.311 (13)
Al9^xii^—Ru3—Al4	127.93 (2)	Al9^vii^—Al8—Si5^ii^	101.04 (2)
Al9—Ru3—Al4	83.669 (15)	Ru1—Al8—Si5^ii^	116.598 (19)
Al6^xiv^—Ru3—Al4	98.593 (12)	Si5^ix^—Al8—Si5^ii^	125.72 (2)
Al7^xv^—Ru3—Al4	96.13 (2)	Al8^ii^—Al8—Si5^ii^	60.177 (19)
Si5^xii^—Ru3—Al7^ii^	93.941 (15)	Al8^iv^—Al8—Si5^ii^	103.902 (16)
Al8^xiii^—Ru3—Al7^ii^	141.416 (15)	Al9^ix^—Al8—Si5^ii^	162.00 (2)
Al6—Ru3—Al7^ii^	94.038 (15)	Si5—Al8—Si5^ii^	94.55 (2)
Al7—Ru3—Al7^ii^	93.18 (2)	Ru3^xvii^—Al9—Ru3	147.99 (2)
Al9^xii^—Ru3—Al7^ii^	139.245 (16)	Ru3^xvii^—Al9—Al8^xiii^	148.40 (2)
Al9—Ru3—Al7^ii^	146.275 (16)	Ru3—Al9—Al8^xiii^	57.934 (15)
Al6^xiv^—Ru3—Al7^ii^	60.377 (15)	Ru3^xvii^—Al9—Ru1^xviii^	105.884 (17)
Al7^xv^—Ru3—Al7^ii^	61.353 (14)	Ru3—Al9—Ru1^xviii^	105.607 (17)
Al4—Ru3—Al7^ii^	62.748 (15)	Al8^xiii^—Al9—Ru1^xviii^	59.509 (15)
Ru3—Al4—Ru3^iv^	113.957 (15)	Ru3^xvii^—Al9—Al9^xii^	138.86 (3)
Ru3—Al4—Ru3^ii^	113.956 (15)	Ru3—Al9—Al9^xii^	57.609 (18)
Ru3^iv^—Al4—Ru3^ii^	113.956 (15)	Al8^xiii^—Al9—Al9^xii^	61.789 (18)
Ru3—Al4—Al6^iv^	109.230 (14)	Ru1^xviii^—Al9—Al9^xii^	59.959 (1)
Ru3^iv^—Al4—Al6^iv^	55.974 (11)	Ru3^xvii^—Al9—Al9^xvii^	57.955 (19)
Ru3^ii^—Al4—Al6^iv^	134.84 (2)	Ru3—Al9—Al9^xvii^	148.22 (3)
Ru3—Al4—Al6^ii^	134.84 (2)	Al8^xiii^—Al9—Al9^xvii^	91.89 (2)
Ru3^iv^—Al4—Al6^ii^	109.230 (14)	Ru1^xviii^—Al9—Al9^xvii^	60.0
Ru3^ii^—Al4—Al6^ii^	55.974 (11)	Al9^xii^—Al9—Al9^xvii^	119.687 (4)
Al6^iv^—Al4—Al6^ii^	84.26 (3)	Ru3^xvii^—Al9—Al8^ix^	56.276 (14)
Ru3—Al4—Al6	55.973 (11)	Ru3—Al9—Al8^ix^	143.01 (2)
Ru3^iv^—Al4—Al6	134.84 (2)	Al8^xiii^—Al9—Al8^ix^	117.42 (3)
Ru3^ii^—Al4—Al6	109.231 (14)	Ru1^xviii^—Al9—Al8^ix^	57.913 (14)
Al6^iv^—Al4—Al6	84.26 (3)	Al9^xii^—Al9—Al8^ix^	87.05 (2)
Al6^ii^—Al4—Al6	84.26 (3)	Al9^xvii^—Al9—Al8^ix^	56.799 (18)
Ru3—Al4—Al7	56.440 (13)	Ru3^xvii^—Al9—Si5	53.152 (13)
Ru3^iv^—Al4—Al7	58.708 (14)	Ru3—Al9—Si5	109.71 (2)
Ru3^ii^—Al4—Al7	127.88 (3)	Al8^xiii^—Al9—Si5	154.89 (3)
Al6^iv^—Al4—Al7	87.091 (13)	Ru1^xviii^—Al9—Si5	109.909 (19)
Al6^ii^—Al4—Al7	167.862 (14)	Al9^xii^—Al9—Si5	93.10 (3)
Al6—Al4—Al7	103.356 (14)	Al9^xvii^—Al9—Si5	102.00 (3)
Ru3—Al4—Al7^iv^	127.88 (3)	Al8^ix^—Al9—Si5	57.332 (16)
Ru3^iv^—Al4—Al7^iv^	56.439 (13)	Ru3^xvii^—Al9—Al6^xvii^	55.877 (14)
Ru3^ii^—Al4—Al7^iv^	58.708 (14)	Ru3—Al9—Al6^xvii^	110.10 (2)
Al6^iv^—Al4—Al7^iv^	103.357 (14)	Al8^xiii^—Al9—Al6^xvii^	105.54 (2)
Al6^ii^—Al4—Al7^iv^	87.091 (13)	Ru1^xviii^—Al9—Al6^xvii^	121.90 (2)
Al6—Al4—Al7^iv^	167.862 (14)	Al9^xii^—Al9—Al6^xvii^	165.26 (3)
Al7—Al4—Al7^iv^	86.64 (2)	Al9^xvii^—Al9—Al6^xvii^	65.56 (2)
Ru3—Al4—Al7^ii^	58.708 (14)	Al8^ix^—Al9—Al6^xvii^	106.35 (2)
Ru3^iv^—Al4—Al7^ii^	127.88 (3)	Si5—Al9—Al6^xvii^	99.26 (2)
Ru3^ii^—Al4—Al7^ii^	56.439 (13)	Ru3^xvii^—Al9—Al7	108.93 (2)
Al6^iv^—Al4—Al7^ii^	167.862 (14)	Ru3—Al9—Al7	56.047 (14)
Al6^ii^—Al4—Al7^ii^	103.357 (14)	Al8^xiii^—Al9—Al7	67.695 (18)
Al6—Al4—Al7^ii^	87.091 (13)	Ru1^xviii^—Al9—Al7	123.68 (2)
Al7—Al4—Al7^ii^	86.64 (2)	Al9^xii^—Al9—Al7	110.49 (3)
Al7^iv^—Al4—Al7^ii^	86.64 (2)	Al9^xvii^—Al9—Al7	105.97 (3)
Ru2—Si5—Si5^ix^	118.81 (3)	Al8^ix^—Al9—Al7	160.85 (2)
Ru2—Si5—Ru3^xvii^	119.762 (18)	Si5—Al9—Al7	126.36 (2)
Si5^ix^—Si5—Ru3^xvii^	120.64 (3)	Al6^xvii^—Al9—Al7	55.443 (16)
Ru2—Si5—Al8^ix^	167.65 (2)		

Symmetry codes: (i) *x*−*y*, *x*, −*z*+1; (ii) −*x*+*y*, −*x*, *z*; (iii) −*x*, −*y*, −*z*+1; (iv) −*y*, *x*−*y*, *z*; (v) *y*, −*x*+*y*, −*z*+1; (vi) *y*−2/3, −*x*+*y*−1/3, −*z*+2/3; (vii) −*y*+2/3, *x*−*y*+1/3, *z*+1/3; (viii) *x*−1/3, *y*−2/3, *z*+1/3; (ix) −*x*+1/3, −*y*+2/3, −*z*+2/3; (x) *x*−*y*+1/3, *x*−1/3, −*z*+2/3; (xi) −*x*+*y*−1/3, −*x*+1/3, *z*+1/3; (xii) *x*−*y*+2/3, *x*+1/3, −*z*+1/3; (xiii) −*x*+*y*+1/3, −*x*+2/3, *z*−1/3; (xiv) −*x*+2/3, −*y*+1/3, −*z*+1/3; (xv) *y*, −*x*+*y*, −*z*; (xvi) *x*−*y*, *x*, −*z*; (xvii) *y*−1/3, −*x*+*y*+1/3, −*z*+1/3; (xviii) *x*+1/3, *y*+2/3, *z*−1/3.

### Heptacosaaluminium decaruthenium tridecasilicon (III) Crystal data

**Table:**

~Ru_10_(Al_0.67_Si_0.33_)_41_	*D*_x_ = 4.652 Mg m^−^^3^
*M**_r_* = 2122.53	Mo *K*α radiation, λ = 0.71073 Å
Orthorhombic, *P**n**m**a*	Cell parameters from 16783 reflections
*a* = 15.0794 (2) Å	θ = 3.4–33.8°
*b* = 11.8713 (2) Å	µ = 6.18 mm^−^^1^
*c* = 16.9291 (3) Å	*T* = 298 K
*V* = 3030.50 (9) Å^3^	Irregular, metallic light silver
*Z* = 4	0.04 × 0.02 × 0.02 mm
*F*(000) = 3888.20	

### Heptacosaaluminium decaruthenium tridecasilicon (III) Data collection

**Table:**

XtaLAB Synergy R, HyPix diffractometer	5672 independent reflections
Radiation source: micro-focus sealed X-ray tube, Mova (Mo) X-ray Source	4940 reflections with *I* > 2σ(*I*)
Mirror monochromator	*R*_int_ = 0.022
Detector resolution: 10.0000 pixels mm^-1^	θ_max_ = 33.7°, θ_min_ = 3.2°
ω scans	*h* = −23→21
Absorption correction: gaussian (CrysAlisPro; Matsumoto *et al.*, 2021)	*k* = −17→18
*T*_min_ = 0.867, *T*_max_ = 0.929	*l* = −25→24
33654 measured reflections	

### Heptacosaaluminium decaruthenium tridecasilicon (III) Refinement

**Table:**

Refinement on *F*^2^	Primary atom site location: dual
Least-squares matrix: full	Secondary atom site location: difference Fourier map
*R*[*F*^2^ > 2σ(*F*^2^)] = 0.015	*w* = 1/[σ^2^(*F*_o_^2^) + (0.0093*P*)^2^ + 1.1293*P*] where *P* = (*F*_o_^2^ + 2*F*_c_^2^)/3
*wR*(*F*^2^) = 0.026	(Δ/σ)_max_ = 0.003
*S* = 1.01	Δρ_max_ = 0.78 e Å^−^^3^
5672 reflections	Δρ_min_ = −0.74 e Å^−^^3^
303 parameters	Extinction correction: SHELXL-2019/3 (Sheldrick, 2015b), Fc^*^=kFc[1+0.001xFc^2^λ^3^/sin(2θ)]^-1/4^
2 restraints	Extinction coefficient: 0.000039 (4)

### Heptacosaaluminium decaruthenium tridecasilicon (III) Special details

**Table:**

Geometry. All esds (except the esd in the dihedral angle between two l.s. planes) are estimated using the full covariance matrix. The cell esds are taken into account individually in the estimation of esds in distances, angles and torsion angles; correlations between esds in cell parameters are only used when they are defined by crystal symmetry. An approximate (isotropic) treatment of cell esds is used for estimating esds involving l.s. planes.

### Heptacosaaluminium decaruthenium tridecasilicon (III) Fractional atomic coordinates and isotropic or equivalent isotropic displacement parameters (Å^2^)

**Table:**

	*x*	*y*	*z*	*U*_iso_*/*U*_eq_	Occ. (<1)
Ru1	0.15195 (2)	0.250000	0.76146 (2)	0.00513 (3)	
Ru2	0.41069 (2)	0.250000	0.87752 (2)	0.00474 (3)	
Ru3	0.45013 (2)	0.250000	0.16946 (2)	0.00542 (3)	
Ru4A	0.1834 (2)	0.250000	0.02174 (3)	0.00665 (15)	0.933 (16)
Ru4B	0.1655 (15)	0.250000	0.0209 (4)	0.0036 (13)*	0.067 (16)
Ru5A	0.42722 (8)	0.0630 (2)	0.38435 (7)	0.00501 (13)	0.74 (2)
Ru5B	0.4253 (3)	0.0509 (5)	0.3844 (2)	0.0052 (4)*	0.26 (2)
Ru6	0.17223 (2)	0.06068 (2)	0.25159 (2)	0.00500 (3)	
Ru7	0.15518 (2)	0.05637 (2)	0.52078 (2)	0.00672 (3)	
Si8	0.20752 (4)	0.250000	0.31440 (3)	0.00625 (11)	
Si9A	0.37913 (7)	0.250000	0.62569 (5)	0.0103 (3)	0.727 (3)
Si9B	0.43672 (17)	0.250000	0.59893 (13)	0.0095 (7)	0.273 (3)
Si10A	0.3512 (4)	0.250000	0.38677 (9)	0.0081 (6)	0.85 (2)
Al10B	0.3758 (16)	0.250000	0.3830 (5)	0.0082 (18)*	0.15 (2)
Al11	0.19544 (4)	0.250000	0.47722 (4)	0.0089 (3)	0.65 (6)
Si11	0.19544 (4)	0.250000	0.47722 (4)	0.0089 (3)	0.35 (6)
Al12	0.48579 (4)	0.250000	0.74245 (4)	0.00666 (12)	
Al13	0.16461 (4)	0.250000	0.16972 (4)	0.00748 (12)	
Al14	0.08651 (5)	0.250000	0.60711 (4)	0.01395 (15)	
Al15	0.33501 (5)	0.250000	0.07842 (5)	0.0183 (3)	0.950 (4)
Si16	0.05763 (3)	0.56298 (4)	0.31273 (2)	0.00608 (8)	
Si17	0.41912 (3)	0.56490 (4)	0.26431 (2)	0.00638 (8)	
Si18	0.19988 (3)	0.62931 (4)	0.45426 (2)	0.00859 (8)	
Si19	0.02383 (3)	0.14871 (4)	0.21915 (2)	0.00739 (8)	
Al20	0.21386 (3)	0.63959 (4)	0.30297 (3)	0.0109 (2)	0.55 (5)
Si20	0.21386 (3)	0.63959 (4)	0.30297 (3)	0.0109 (2)	0.45 (5)
Al21	0.42109 (3)	0.04580 (4)	0.12868 (3)	0.0072 (2)	0.64 (5)
Si21	0.42109 (3)	0.04580 (4)	0.12868 (3)	0.0072 (2)	0.36 (5)
Al22	0.33859 (3)	0.11923 (4)	0.51409 (3)	0.0115 (2)	0.67 (5)
Si22	0.33859 (3)	0.11923 (4)	0.51409 (3)	0.0115 (2)	0.33 (5)
Al23	0.33880 (3)	0.12822 (4)	0.25123 (3)	0.00721 (9)	
Al24	0.25334 (3)	0.04455 (4)	0.38472 (3)	0.00724 (9)	
Al25	0.07288 (3)	0.54572 (4)	0.15156 (3)	0.00777 (9)	
Al26	0.08203 (3)	0.13043 (4)	0.38371 (3)	0.00734 (9)	
Al27	0.21669 (3)	0.06506 (4)	0.09349 (3)	0.01224 (10)	
Al28	0.02308 (3)	0.59649 (5)	0.46632 (3)	0.01334 (10)	
Al29A	0.3572 (3)	0.64190 (10)	0.3938 (2)	0.0077 (5)	0.79 (2)
Al29B	0.3742 (7)	0.6377 (5)	0.4074 (7)	0.0109 (11)*	0.21 (2)
Al30A	0.28629 (14)	0.6034 (3)	0.16459 (9)	0.0188 (6)	0.754 (11)
Al30B	0.2701 (3)	0.6380 (5)	0.1547 (2)	0.0136 (8)*	0.246 (11)
Al31A	0.01771 (7)	0.250000	0.07942 (7)	0.0153 (5)	0.728 (5)
Al31B	0.0308 (4)	0.1965 (6)	0.0529 (4)	0.015 (2)*	0.104 (3)
Al31C	0.0457 (8)	0.1351 (13)	0.0372 (7)	0.016 (4)*	0.047 (3)
Al32A	0.06370 (5)	0.06801 (7)	0.01653 (4)	0.0151 (3)	0.716 (3)
Al32B	0.0186 (3)	0.0224 (4)	0.0048 (3)	0.0129 (15)*	0.121 (3)

### Heptacosaaluminium decaruthenium tridecasilicon (III) Atomic displacement parameters (Å^2^)

**Table:**

	*U*^11^	*U*^22^	*U*^33^	*U*^12^	*U*^13^	*U*^23^
Ru1	0.00431 (7)	0.00457 (8)	0.00651 (7)	0.000	−0.00060 (6)	0.000
Ru2	0.00433 (7)	0.00403 (7)	0.00587 (7)	0.000	−0.00033 (5)	0.000
Ru3	0.00414 (7)	0.00694 (8)	0.00517 (7)	0.000	−0.00026 (6)	0.000
Ru4A	0.0081 (5)	0.00790 (13)	0.00392 (11)	0.000	0.00035 (13)	0.000
Ru5A	0.00401 (16)	0.0042 (3)	0.00676 (16)	−0.00089 (18)	0.00098 (7)	−0.00108 (17)
Ru6	0.00449 (5)	0.00601 (6)	0.00452 (5)	−0.00090 (4)	−0.00053 (4)	0.00048 (4)
Ru7	0.00645 (5)	0.00859 (6)	0.00513 (5)	0.00172 (4)	−0.00042 (4)	−0.00090 (4)
Si8	0.0067 (3)	0.0055 (3)	0.0065 (3)	0.000	−0.0001 (2)	0.000
Si9A	0.0154 (5)	0.0066 (4)	0.0088 (4)	0.000	−0.0050 (3)	0.000
Si9B	0.0144 (15)	0.0052 (11)	0.0091 (11)	0.000	−0.0036 (9)	0.000
Si10A	0.0098 (14)	0.0055 (4)	0.0090 (4)	0.000	−0.0013 (4)	0.000
Al11	0.0091 (4)	0.0069 (4)	0.0108 (4)	0.000	−0.0005 (2)	0.000
Si11	0.0091 (4)	0.0069 (4)	0.0108 (4)	0.000	−0.0005 (2)	0.000
Al12	0.0060 (3)	0.0063 (3)	0.0076 (3)	0.000	0.0008 (2)	0.000
Al13	0.0108 (3)	0.0065 (3)	0.0051 (3)	0.000	0.0012 (2)	0.000
Al14	0.0090 (3)	0.0204 (4)	0.0124 (3)	0.000	0.0002 (3)	0.000
Al15	0.0172 (5)	0.0158 (5)	0.0219 (5)	0.000	−0.0147 (3)	0.000
Si16	0.00579 (18)	0.00568 (19)	0.00676 (18)	0.00030 (15)	−0.00049 (14)	−0.00084 (14)
Si17	0.00719 (18)	0.00518 (19)	0.00678 (18)	0.00049 (15)	0.00114 (14)	0.00033 (14)
Si18	0.0088 (2)	0.0083 (2)	0.00861 (19)	0.00025 (16)	−0.00109 (15)	0.00063 (15)
Si19	0.00649 (18)	0.0067 (2)	0.00891 (19)	0.00029 (16)	0.00037 (14)	−0.00100 (15)
Al20	0.0094 (3)	0.0074 (3)	0.0159 (3)	−0.00202 (17)	0.00544 (17)	−0.00150 (17)
Si20	0.0094 (3)	0.0074 (3)	0.0159 (3)	−0.00202 (17)	0.00544 (17)	−0.00150 (17)
Al21	0.0072 (3)	0.0072 (3)	0.0072 (3)	−0.00030 (17)	0.00040 (15)	−0.00223 (16)
Si21	0.0072 (3)	0.0072 (3)	0.0072 (3)	−0.00030 (17)	0.00040 (15)	−0.00223 (16)
Al22	0.0143 (3)	0.0126 (3)	0.0075 (3)	−0.00185 (19)	−0.00166 (17)	0.00134 (17)
Si22	0.0143 (3)	0.0126 (3)	0.0075 (3)	−0.00185 (19)	−0.00166 (17)	0.00134 (17)
Al23	0.0067 (2)	0.0077 (2)	0.0072 (2)	0.00081 (17)	0.00118 (16)	0.00033 (16)
Al24	0.0070 (2)	0.0080 (2)	0.0068 (2)	−0.00033 (17)	−0.00041 (16)	0.00027 (16)
Al25	0.0073 (2)	0.0074 (2)	0.0086 (2)	−0.00030 (17)	0.00002 (16)	0.00109 (17)
Al26	0.0060 (2)	0.0076 (2)	0.0085 (2)	−0.00025 (17)	−0.00033 (16)	0.00000 (16)
Al27	0.0154 (2)	0.0077 (2)	0.0135 (2)	−0.0003 (2)	0.00641 (19)	−0.00069 (18)
Al28	0.0115 (2)	0.0150 (3)	0.0135 (2)	−0.0029 (2)	0.00047 (18)	0.00552 (19)
Al29A	0.0139 (8)	0.0046 (4)	0.0047 (6)	0.0026 (3)	0.0010 (6)	−0.0011 (3)
Al30A	0.0182 (6)	0.0278 (11)	0.0103 (4)	−0.0093 (7)	0.0000 (4)	−0.0088 (5)
Al31A	0.0090 (5)	0.0215 (9)	0.0155 (6)	0.000	−0.0021 (4)	0.000
Al32A	0.0167 (5)	0.0181 (5)	0.0105 (4)	0.0000 (3)	0.0052 (3)	0.0019 (3)

### Heptacosaaluminium decaruthenium tridecasilicon (III) Geometric parameters (Å, º)

**Table:**

Ru1—Si17^i^	2.4453 (4)	Si9B—Al25^ii^	2.5876 (10)
Ru1—Si17^ii^	2.4453 (4)	Si9B—Al25^i^	2.5876 (10)
Ru1—Al12^iii^	2.5063 (7)	Si10A—Al31A^vi^	2.576 (5)
Ru1—Al20^ii^	2.5113 (5)	Si10A—Al22^vii^	2.6631 (16)
Ru1—Al20^i^	2.5113 (5)	Si10A—Al22	2.6631 (16)
Ru1—Al30B^i^	2.534 (3)	Si10A—Al23^vii^	2.7183 (11)
Ru1—Al30B^ii^	2.534 (3)	Si10A—Al23	2.7183 (11)
Ru1—Al30A^i^	2.5661 (12)	Si10A—Al11	2.803 (5)
Ru1—Al30A^ii^	2.5661 (12)	Al10B—Al31A^vi^	2.23 (2)
Ru1—Al29A^ii^	2.585 (4)	Al10B—Al31B^v^	2.65 (2)
Ru1—Al29A^i^	2.585 (4)	Al10B—Al31B^vi^	2.65 (2)
Ru1—Al14	2.7932 (7)	Al10B—Al23^vii^	2.716 (7)
Ru2—Si16^i^	2.5220 (4)	Al10B—Al23	2.716 (7)
Ru2—Si16^ii^	2.5220 (4)	Al10B—Al22^vii^	2.766 (10)
Ru2—Al12	2.5516 (6)	Al10B—Al22	2.766 (10)
Ru2—Si18^i^	2.5536 (5)	Al11—Al26^vii^	2.7287 (7)
Ru2—Si18^ii^	2.5536 (4)	Al11—Al26	2.7288 (7)
Ru2—Al28^ii^	2.5649 (5)	Al11—Al22^vii^	2.7310 (7)
Ru2—Al28^i^	2.5649 (5)	Al11—Al22	2.7310 (7)
Ru2—Al20^i^	2.6150 (5)	Al11—Al14	2.7447 (9)
Ru2—Al20^ii^	2.6150 (5)	Al12—Si16^ii^	2.6025 (5)
Ru2—Al14^iv^	2.6640 (7)	Al12—Si16^i^	2.6025 (5)
Ru3—Al15	2.3213 (8)	Al12—Si17^xii^	2.6263 (6)
Ru3—Si19^v^	2.4975 (4)	Al12—Si17^xiii^	2.6263 (6)
Ru3—Si19^vi^	2.4975 (4)	Al12—Al14^iv^	2.9652 (10)
Ru3—Al21^vii^	2.5582 (5)	Al13—Si19^vii^	2.5792 (7)
Ru3—Al21	2.5582 (5)	Al13—Si19	2.5793 (7)
Ru3—Al26^v^	2.6041 (5)	Al13—Al27	2.6650 (6)
Ru3—Al26^vi^	2.6041 (5)	Al13—Al27^vii^	2.6650 (6)
Ru3—Al23^vii^	2.6124 (5)	Al13—Al31A	2.6914 (12)
Ru3—Al23	2.6124 (5)	Al13—Al31B	2.896 (6)
Ru4A—Al31B	2.445 (6)	Al13—Al31B^vii^	2.896 (6)
Ru4A—Al31B^vii^	2.445 (6)	Al14—Al30B^ii^	2.663 (3)
Ru4A—Al15	2.479 (3)	Al14—Al30B^i^	2.663 (3)
Ru4A—Al31C^vii^	2.498 (12)	Al14—Al28^xi^	2.7563 (7)
Ru4A—Al31C	2.498 (12)	Al14—Al28^xiv^	2.7563 (7)
Ru4A—Al29B^viii^	2.505 (6)	Al14—Al30A^ii^	2.7665 (17)
Ru4A—Al29B^ix^	2.505 (6)	Al14—Al30A^i^	2.7665 (16)
Ru4A—Al13	2.5211 (8)	Al15—Si18^viii^	2.5976 (8)
Ru4A—Si18^ix^	2.541 (2)	Al15—Si18^ix^	2.5976 (8)
Ru4A—Si18^viii^	2.541 (2)	Al15—Al27^vii^	2.8406 (7)
Ru4A—Al27	2.5588 (9)	Al15—Al27	2.8406 (7)
Ru4A—Al27^vii^	2.5588 (9)	Al15—Al21	2.8784 (6)
Ru4B—Al31B	2.20 (2)	Al15—Al21^vii^	2.8784 (6)
Ru4B—Al31B^vii^	2.20 (2)	Si16—Si17^xv^	2.4623 (6)
Ru4B—Al31C^vii^	2.28 (2)	Si16—Al20	2.5307 (6)
Ru4B—Al31C	2.28 (2)	Si16—Al26^vii^	2.6175 (6)
Ru4B—Al29B^viii^	2.413 (11)	Si16—Al21^xvi^	2.6250 (6)
Ru4B—Al29B^ix^	2.413 (11)	Si16—Al28	2.6816 (6)
Ru4B—Al31A	2.44 (2)	Si16—Al25	2.7456 (6)
Ru4B—Al13	2.520 (7)	Si17—Al29A	2.5512 (15)
Ru4B—Al29A^viii^	2.529 (7)	Si17—Al23^vii^	2.6024 (6)
Ru4B—Al29A^ix^	2.529 (7)	Si17—Al21^vii^	2.6456 (6)
Ru4B—Al27	2.632 (8)	Si17—Al30A	2.659 (3)
Ru4B—Al27^vii^	2.632 (8)	Si17—Al29B	2.660 (8)
Ru5A—Al10B	2.352 (9)	Si17—Al25^vi^	2.7306 (6)
Ru5A—Al32B^vi^	2.377 (5)	Si18—Al24^vii^	2.5092 (6)
Ru5A—Al31C^vi^	2.385 (12)	Si18—Al20	2.5727 (6)
Ru5A—Al32B^x^	2.419 (5)	Si18—Al29A	2.589 (3)
Ru5A—Al31B^vi^	2.466 (6)	Si18—Al28	2.7022 (7)
Ru5A—Si19^vi^	2.4958 (19)	Si18—Al29B	2.748 (7)
Ru5A—Si10A	2.499 (4)	Si18—Al27^xvii^	2.7785 (6)
Ru5A—Si17^vii^	2.5395 (19)	Si19—Si19^vii^	2.4048 (9)
Ru5A—Al29B^vii^	2.543 (7)	Si19—Al31A	2.6552 (12)
Ru5A—Al25^v^	2.6190 (16)	Si19—Al25^vii^	2.6803 (6)
Ru5A—Al24	2.6311 (14)	Si19—Al23^xv^	2.8453 (6)
Ru5A—Al29A^vii^	2.656 (4)	Si19—Al31B	2.874 (6)
Ru5B—Al32B^vi^	2.368 (6)	Al20—Al30A	2.6202 (10)
Ru5B—Al32B^x^	2.372 (6)	Al20—Al20^xviii^	2.6215 (10)
Ru5B—Al29B^vii^	2.400 (9)	Al20—Al30B	2.650 (3)
Ru5B—Si17^vii^	2.456 (5)	Al20—Al29A	2.653 (6)
Ru5B—Al31C^vi^	2.460 (13)	Al20—Al24^vii^	2.6549 (7)
Ru5B—Al10B	2.479 (9)	Al20—Al29B	2.996 (14)
Ru5B—Al29A^vii^	2.513 (7)	Al21—Al23	2.6080 (6)
Ru5B—Si19^vi^	2.575 (5)	Al21—Al26^vi^	2.6350 (6)
Ru5B—Al25^v^	2.576 (4)	Al21—Al30A^vii^	2.764 (3)
Ru5B—Al31B^vi^	2.578 (8)	Al21—Al28^v^	2.7938 (7)
Ru5B—Al24	2.594 (4)	Al21—Al28^viii^	2.9369 (7)
Ru5B—Si10A	2.615 (6)	Al22—Al32A^x^	2.6670 (10)
Ru6—Si16^vii^	2.4926 (4)	Al22—Al24	2.6898 (6)
Ru6—Si19	2.5300 (4)	Al22—Al27^x^	2.6996 (7)
Ru6—Si8	2.5426 (3)	Al22—Al32B^x^	2.737 (5)
Ru6—Al24	2.5715 (5)	Al23—Al24	2.7846 (6)
Ru6—Al25^vii^	2.5898 (5)	Al24—Al29A^vii^	2.716 (3)
Ru6—Al20^vii^	2.6083 (5)	Al24—Al26	2.7772 (6)
Ru6—Al23	2.6367 (5)	Al25—Al32A^vii^	2.6586 (8)
Ru6—Al13	2.6429 (4)	Al25—Al27^vii^	2.7200 (7)
Ru6—Al26	2.7455 (5)	Al25—Al32B^vii^	2.738 (4)
Ru6—Al27	2.7596 (5)	Al26—Al26^vii^	2.8388 (10)
Ru6—Al30A^vii^	2.987 (2)	Al27—Al30A^vii^	2.5594 (16)
Ru7—Al21^x^	2.4760 (5)	Al27—Al32A	2.6497 (10)
Ru7—Al11	2.4892 (3)	Al27—Al30B^vii^	2.744 (4)
Ru7—Si18^vii^	2.5655 (5)	Al28—Al28^xiv^	2.6519 (10)
Ru7—Al30A^i^	2.6492 (8)	Al29A—Al32A^xvii^	2.551 (5)
Ru7—Al27^x^	2.7068 (5)	Al29A—Al29A^xviii^	2.567 (2)
Ru7—Al30B^i^	2.711 (3)	Al29A—Al29B^xviii^	2.639 (6)
Ru7—Al26	2.7156 (5)	Al29B—Al32A^xvii^	2.229 (14)
Ru7—Al28^xi^	2.7385 (5)	Al29B—Al31C^xvii^	2.507 (19)
Ru7—Al24	2.7416 (5)	Al29B—Al29B^xviii^	2.666 (12)
Ru7—Al28^vii^	2.8479 (5)	Al29B—Al32B^xvii^	2.684 (15)
Ru7—Al22	2.8669 (5)	Al30A—Al30B	0.507 (4)
Si8—Si10A	2.489 (5)	Al30B—Al30B^xviii^	2.659 (12)
Si8—Al13	2.5334 (8)	Al31A—Al31B^vii^	0.802 (8)
Si8—Al26^vii^	2.6404 (7)	Al31A—Al31B	0.802 (8)
Si8—Al26	2.6404 (7)	Al31A—Al31C	1.596 (16)
Si8—Al23^vii^	2.6744 (7)	Al31A—Al31C^vii^	1.596 (16)
Si8—Al23	2.6744 (7)	Al31A—Al32A^vii^	2.5064 (11)
Si8—Al11	2.7624 (9)	Al31A—Al32A	2.5064 (11)
Si8—Al10B	2.79 (2)	Al31B—Al31C	0.808 (15)
Si8—Al24^vii^	2.8005 (5)	Al31B—Al31B^vii^	1.269 (15)
Si9A—Si9B	0.979 (3)	Al31B—Al32A	1.718 (8)
Si9A—Al22^vii^	2.5205 (8)	Al31B—Al31C^vii^	2.028 (19)
Si9A—Al22	2.5205 (8)	Al31B—Al32B	2.229 (9)
Si9A—Al12	2.5484 (10)	Al31C—Al32A	0.912 (16)
Si9A—Al25^ii^	2.5683 (6)	Al31C—Al32B	1.503 (16)
Si9A—Al25^i^	2.5683 (6)	Al31C—Al32B^xix^	2.223 (15)
Si9A—Al30B^i^	2.659 (7)	Al31C—Al31C^vii^	2.73 (3)
Si9A—Al30B^ii^	2.659 (7)	Al32A—Al32B	0.892 (4)
Si9B—Al12	2.540 (2)	Al32A—Al32B^xix^	1.680 (5)
Si9B—Al22^vii^	2.5812 (19)	Al32A—Al32A^xix^	2.5713 (17)
Si9B—Al22	2.5812 (19)	Al32B—Al32B^xix^	0.790 (9)
			
Si17^i^—Ru1—Si17^ii^	127.96 (2)	Ru2^ix^—Si18—Al15^ii^	88.28 (2)
Si17^i^—Ru1—Al12^iii^	64.049 (11)	Ru7^vii^—Si18—Al15^ii^	93.755 (17)
Si17^ii^—Ru1—Al12^iii^	64.049 (11)	Al20—Si18—Al15^ii^	142.75 (2)
Si17^i^—Ru1—Al20^ii^	144.754 (15)	Al29A—Si18—Al15^ii^	118.30 (8)
Si17^ii^—Ru1—Al20^ii^	83.029 (15)	Al24^vii^—Si18—Al28	103.52 (2)
Al12^iii^—Ru1—Al20^ii^	144.381 (13)	Ru4A^ii^—Si18—Al28	136.96 (5)
Si17^i^—Ru1—Al20^i^	83.029 (15)	Ru2^ix^—Si18—Al28	58.339 (14)
Si17^ii^—Ru1—Al20^i^	144.754 (15)	Ru7^vii^—Si18—Al28	65.395 (15)
Al12^iii^—Ru1—Al20^i^	144.381 (14)	Al20—Si18—Al28	99.38 (2)
Al20^ii^—Ru1—Al20^i^	62.92 (2)	Al29A—Si18—Al28	160.49 (10)
Si17^i^—Ru1—Al30B^i^	75.28 (17)	Al15^ii^—Si18—Al28	79.54 (2)
Si17^ii^—Ru1—Al30B^i^	133.55 (13)	Al24^vii^—Si18—Ru4B^ii^	112.7 (3)
Al12^iii^—Ru1—Al30B^i^	116.43 (11)	Ru4A^ii^—Si18—Ru4B^ii^	4.2 (3)
Al20^ii^—Ru1—Al30B^i^	95.71 (13)	Ru2^ix^—Si18—Ru4B^ii^	113.67 (18)
Al20^i^—Ru1—Al30B^i^	63.37 (8)	Ru7^vii^—Si18—Ru4B^ii^	117.73 (11)
Si17^i^—Ru1—Al30B^ii^	133.55 (13)	Al20—Si18—Ru4B^ii^	109.0 (2)
Si17^ii^—Ru1—Al30B^ii^	75.28 (17)	Al29A—Si18—Ru4B^ii^	56.7 (3)
Al12^iii^—Ru1—Al30B^ii^	116.43 (11)	Al15^ii^—Si18—Ru4B^ii^	61.8 (3)
Al20^ii^—Ru1—Al30B^ii^	63.37 (8)	Al28—Si18—Ru4B^ii^	141.1 (3)
Al20^i^—Ru1—Al30B^ii^	95.71 (13)	Al24^vii^—Si18—Al29B	65.62 (12)
Al30B^i^—Ru1—Al30B^ii^	63.3 (3)	Ru4A^ii^—Si18—Al29B	56.4 (2)
Si17^i^—Ru1—Al30A^i^	64.04 (9)	Ru2^ix^—Si18—Al29B	117.2 (3)
Si17^ii^—Ru1—Al30A^i^	141.35 (6)	Ru7^vii^—Si18—Al29B	114.16 (19)
Al12^iii^—Ru1—Al30A^i^	110.24 (6)	Al20—Si18—Al29B	68.5 (3)
Al20^ii^—Ru1—Al30A^i^	103.90 (7)	Al29A—Si18—Al29B	6.68 (19)
Al20^i^—Ru1—Al30A^i^	62.12 (2)	Al15^ii^—Si18—Al29B	114.0 (2)
Al30B^i^—Ru1—Al30A^i^	11.38 (9)	Al28—Si18—Al29B	166.2 (2)
Al30B^ii^—Ru1—Al30A^i^	74.4 (3)	Ru4B^ii^—Si18—Al29B	52.3 (4)
Si17^i^—Ru1—Al30A^ii^	141.35 (6)	Al24^vii^—Si18—Al27^xvii^	91.53 (2)
Si17^ii^—Ru1—Al30A^ii^	64.04 (9)	Ru4A^ii^—Si18—Al27^xvii^	57.299 (16)
Al12^iii^—Ru1—Al30A^ii^	110.24 (6)	Ru2^ix^—Si18—Al27^xvii^	151.80 (2)
Al20^ii^—Ru1—Al30A^ii^	62.12 (2)	Ru7^vii^—Si18—Al27^xvii^	60.707 (14)
Al20^i^—Ru1—Al30A^ii^	103.90 (7)	Al20—Si18—Al27^xvii^	145.13 (2)
Al30B^i^—Ru1—Al30A^ii^	74.4 (3)	Al29A—Si18—Al27^xvii^	86.36 (11)
Al30B^ii^—Ru1—Al30A^ii^	11.38 (9)	Al15^ii^—Si18—Al27^xvii^	63.69 (2)
Al30A^i^—Ru1—Al30A^ii^	85.39 (18)	Al28—Si18—Al27^xvii^	110.06 (2)
Si17^i^—Ru1—Al29A^ii^	113.93 (8)	Ru4B^ii^—Si18—Al27^xvii^	57.10 (11)
Si17^ii^—Ru1—Al29A^ii^	60.88 (3)	Al29B—Si18—Al27^xvii^	79.7 (3)
Al12^iii^—Ru1—Al29A^ii^	88.24 (10)	Si19^vii^—Si19—Ru5A^xv^	114.07 (6)
Al20^ii^—Ru1—Al29A^ii^	62.72 (10)	Si19^vii^—Si19—Ru3^xv^	61.220 (10)
Al20^i^—Ru1—Al29A^ii^	93.44 (7)	Ru5A^xv^—Si19—Ru3^xv^	117.82 (4)
Al30B^i^—Ru1—Al29A^ii^	154.64 (12)	Si19^vii^—Si19—Ru6	114.396 (10)
Al30B^ii^—Ru1—Al29A^ii^	112.49 (16)	Ru5A^xv^—Si19—Ru6	120.02 (5)
Al30A^i^—Ru1—Al29A^ii^	155.48 (6)	Ru3^xv^—Si19—Ru6	115.378 (16)
Al30A^ii^—Ru1—Al29A^ii^	103.68 (10)	Si19^vii^—Si19—Ru5B^xv^	116.81 (12)
Si17^i^—Ru1—Al29A^i^	60.88 (3)	Ru5A^xv^—Si19—Ru5B^xv^	2.78 (10)
Si17^ii^—Ru1—Al29A^i^	113.93 (8)	Ru3^xv^—Si19—Ru5B^xv^	118.33 (8)
Al12^iii^—Ru1—Al29A^i^	88.24 (10)	Ru6—Si19—Ru5B^xv^	118.15 (10)
Al20^ii^—Ru1—Al29A^i^	93.44 (7)	Si19^vii^—Si19—Al13	62.212 (12)
Al20^i^—Ru1—Al29A^i^	62.72 (10)	Ru5A^xv^—Si19—Al13	116.28 (4)
Al30B^i^—Ru1—Al29A^i^	112.49 (15)	Ru3^xv^—Si19—Al13	112.756 (19)
Al30B^ii^—Ru1—Al29A^i^	154.64 (12)	Ru6—Si19—Al13	62.290 (14)
Al30A^i^—Ru1—Al29A^i^	103.68 (10)	Ru5B^xv^—Si19—Al13	117.66 (10)
Al30A^ii^—Ru1—Al29A^i^	155.48 (6)	Si19^vii^—Si19—Al31A	63.073 (15)
Al29A^ii^—Ru1—Al29A^i^	59.54 (7)	Ru5A^xv^—Si19—Al31A	62.54 (5)
Si17^i^—Ru1—Al14	82.158 (12)	Ru3^xv^—Si19—Al31A	116.05 (2)
Si17^ii^—Ru1—Al14	82.158 (12)	Ru6—Si19—Al31A	114.28 (3)
Al12^iii^—Ru1—Al14	67.80 (2)	Ru5B^xv^—Si19—Al31A	65.01 (12)
Al20^ii^—Ru1—Al14	123.122 (17)	Al13—Si19—Al31A	61.86 (3)
Al20^i^—Ru1—Al14	123.122 (17)	Si19^vii^—Si19—Al25^vii^	149.448 (13)
Al30B^i^—Ru1—Al14	59.76 (8)	Ru5A^xv^—Si19—Al25^vii^	60.67 (5)
Al30B^ii^—Ru1—Al14	59.76 (8)	Ru3^xv^—Si19—Al25^vii^	149.29 (2)
Al30A^i^—Ru1—Al14	61.99 (3)	Ru6—Si19—Al25^vii^	59.526 (14)
Al30A^ii^—Ru1—Al14	61.99 (3)	Ru5B^xv^—Si19—Al25^vii^	58.67 (10)
Al29A^ii^—Ru1—Al14	142.33 (5)	Al13—Si19—Al25^vii^	92.054 (18)
Al29A^i^—Ru1—Al14	142.33 (5)	Al31A—Si19—Al25^vii^	91.10 (2)
Si16^i^—Ru2—Si16^ii^	123.36 (2)	Si19^vii^—Si19—Al23^xv^	94.905 (13)
Si16^i^—Ru2—Al12	61.718 (10)	Ru5A^xv^—Si19—Al23^xv^	61.08 (3)
Si16^ii^—Ru2—Al12	61.718 (10)	Ru3^xv^—Si19—Al23^xv^	58.108 (13)
Si16^i^—Ru2—Si18^i^	81.434 (14)	Ru6—Si19—Al23^xv^	142.54 (2)
Si16^ii^—Ru2—Si18^i^	147.022 (15)	Ru5B^xv^—Si19—Al23^xv^	61.03 (8)
Al12—Ru2—Si18^i^	138.220 (13)	Al13—Si19—Al23^xv^	154.71 (2)
Si16^i^—Ru2—Si18^ii^	147.022 (15)	Al31A—Si19—Al23^xv^	99.30 (3)
Si16^ii^—Ru2—Si18^ii^	81.434 (14)	Al25^vii^—Si19—Al23^xv^	105.80 (2)
Al12—Ru2—Si18^ii^	138.220 (13)	Si19^vii^—Si19—Al31B	78.60 (16)
Si18^i^—Ru2—Si18^ii^	68.26 (2)	Ru5A^xv^—Si19—Al31B	54.13 (13)
Si16^i^—Ru2—Al28^ii^	143.769 (17)	Ru3^xv^—Si19—Al31B	131.36 (16)
Si16^ii^—Ru2—Al28^ii^	63.620 (15)	Ru6—Si19—Al31B	105.19 (13)
Al12—Ru2—Al28^ii^	110.636 (16)	Ru5B^xv^—Si19—Al31B	56.15 (17)
Si18^i^—Ru2—Al28^ii^	110.775 (16)	Al13—Si19—Al31B	63.90 (11)
Si18^ii^—Ru2—Al28^ii^	63.730 (15)	Al31A—Si19—Al31B	16.07 (16)
Si16^i^—Ru2—Al28^i^	63.620 (15)	Al25^vii^—Si19—Al31B	75.04 (16)
Si16^ii^—Ru2—Al28^i^	143.769 (17)	Al23^xv^—Si19—Al31B	103.02 (11)
Al12—Ru2—Al28^i^	110.636 (16)	Ru1^ix^—Al20—Si16	162.93 (2)
Si18^i^—Ru2—Al28^i^	63.730 (15)	Ru1^ix^—Al20—Si18	111.67 (2)
Si18^ii^—Ru2—Al28^i^	110.775 (16)	Si16—Al20—Si18	80.894 (18)
Al28^ii^—Ru2—Al28^i^	90.55 (2)	Ru1^ix^—Al20—Ru6^vii^	125.197 (19)
Si16^i^—Ru2—Al20^i^	58.997 (14)	Si16—Al20—Ru6^vii^	58.004 (14)
Si16^ii^—Ru2—Al20^i^	111.555 (15)	Si18—Al20—Ru6^vii^	105.61 (2)
Al12—Ru2—Al20^i^	83.470 (17)	Ru1^ix^—Al20—Ru2^ix^	116.882 (18)
Si18^i^—Ru2—Al20^i^	59.691 (15)	Si16—Al20—Ru2^ix^	58.669 (14)
Si18^ii^—Ru2—Al20^i^	93.313 (15)	Si18—Al20—Ru2^ix^	58.968 (14)
Al28^ii^—Ru2—Al20^i^	156.733 (17)	Ru6^vii^—Al20—Ru2^ix^	116.420 (18)
Al28^i^—Ru2—Al20^i^	101.912 (16)	Ru1^ix^—Al20—Al30A	59.97 (3)
Si16^i^—Ru2—Al20^ii^	111.555 (15)	Si16—Al20—Al30A	112.80 (3)
Si16^ii^—Ru2—Al20^ii^	58.997 (14)	Si18—Al20—Al30A	156.41 (9)
Al12—Ru2—Al20^ii^	83.470 (17)	Ru6^vii^—Al20—Al30A	69.68 (6)
Si18^i^—Ru2—Al20^ii^	93.313 (15)	Ru2^ix^—Al20—Al30A	144.39 (9)
Si18^ii^—Ru2—Al20^ii^	59.691 (15)	Ru1^ix^—Al20—Al20^xviii^	58.538 (11)
Al28^ii^—Ru2—Al20^ii^	101.912 (16)	Si16—Al20—Al20^xviii^	111.059 (14)
Al28^i^—Ru2—Al20^ii^	156.733 (17)	Si18—Al20—Al20^xviii^	92.718 (15)
Al20^i^—Ru2—Al20^ii^	60.16 (2)	Ru6^vii^—Al20—Al20^xviii^	155.712 (11)
Si16^i^—Ru2—Al14^iv^	81.602 (12)	Ru2^ix^—Al20—Al20^xviii^	59.918 (10)
Si16^ii^—Ru2—Al14^iv^	81.602 (12)	Al30A—Al20—Al20^xviii^	99.43 (8)
Al12—Ru2—Al14^iv^	69.26 (2)	Ru1^ix^—Al20—Al30B	58.73 (7)
Si18^i^—Ru2—Al14^iv^	126.881 (15)	Si16—Al20—Al30B	110.92 (8)
Si18^ii^—Ru2—Al14^iv^	126.881 (15)	Si18—Al20—Al30B	165.71 (13)
Al28^ii^—Ru2—Al14^iv^	63.592 (15)	Ru6^vii^—Al20—Al30B	75.79 (10)
Al28^i^—Ru2—Al14^iv^	63.592 (15)	Ru2^ix^—Al20—Al30B	133.67 (16)
Al20^i^—Ru2—Al14^iv^	139.634 (15)	Al30A—Al20—Al30B	11.01 (8)
Al20^ii^—Ru2—Al14^iv^	139.634 (15)	Al20^xviii^—Al20—Al30B	90.41 (13)
Al15—Ru3—Si19^v^	146.524 (15)	Ru1^ix^—Al20—Al29A	60.00 (3)
Al15—Ru3—Si19^vi^	146.524 (15)	Si16—Al20—Al29A	136.44 (3)
Si19^v^—Ru3—Si19^vi^	57.56 (2)	Si18—Al20—Al29A	59.37 (3)
Al15—Ru3—Al21^vii^	72.113 (11)	Ru6^vii^—Al20—Al29A	113.50 (3)
Si19^v^—Ru3—Al21^vii^	79.846 (14)	Ru2^ix^—Al20—Al29A	107.50 (3)
Si19^vi^—Ru3—Al21^vii^	137.403 (15)	Al30A—Al20—Al29A	100.36 (6)
Al15—Ru3—Al21	72.112 (11)	Al20^xviii^—Al20—Al29A	89.41 (3)
Si19^v^—Ru3—Al21	137.404 (15)	Al30B—Al20—Al29A	106.75 (11)
Si19^vi^—Ru3—Al21	79.847 (14)	Ru1^ix^—Al20—Al24^vii^	113.26 (2)
Al21^vii^—Ru3—Al21	142.73 (2)	Si16—Al20—Al24^vii^	83.043 (19)
Al15—Ru3—Al26^v^	109.98 (2)	Si18—Al20—Al24^vii^	57.344 (17)
Si19^v^—Ru3—Al26^v^	70.039 (14)	Ru6^vii^—Al20—Al24^vii^	58.487 (14)
Si19^vi^—Ru3—Al26^v^	100.573 (15)	Ru2^ix^—Al20—Al24^vii^	108.797 (19)
Al21^vii^—Ru3—Al26^v^	61.378 (15)	Al30A—Al20—Al24^vii^	103.75 (9)
Al21—Ru3—Al26^v^	123.640 (16)	Al20^xviii^—Al20—Al24^vii^	145.427 (14)
Al15—Ru3—Al26^vi^	109.98 (2)	Al30B—Al20—Al24^vii^	114.60 (16)
Si19^v^—Ru3—Al26^vi^	100.573 (15)	Al29A—Al20—Al24^vii^	61.56 (3)
Si19^vi^—Ru3—Al26^vi^	70.039 (14)	Ru1^ix^—Al20—Al29B	61.22 (13)
Al21^vii^—Ru3—Al26^vi^	123.640 (16)	Si16—Al20—Al29B	135.26 (13)
Al21—Ru3—Al26^vi^	61.378 (15)	Si18—Al20—Al29B	58.55 (11)
Al26^v^—Ru3—Al26^vi^	66.06 (2)	Ru6^vii^—Al20—Al29B	112.61 (11)
Al15—Ru3—Al23^vii^	82.61 (2)	Ru2^ix^—Al20—Al29B	107.37 (10)
Si19^v^—Ru3—Al23^vii^	67.631 (14)	Al30A—Al20—Al29B	100.96 (12)
Si19^vi^—Ru3—Al23^vii^	98.759 (15)	Al20^xviii^—Al20—Al29B	90.42 (11)
Al21^vii^—Ru3—Al23^vii^	60.570 (14)	Al30B—Al20—Al29B	107.52 (16)
Al21—Ru3—Al23^vii^	123.873 (15)	Al29A—Al20—Al29B	1.27 (13)
Al26^v^—Ru3—Al23^vii^	111.863 (15)	Al24^vii^—Al20—Al29B	60.35 (12)
Al26^vi^—Ru3—Al23^vii^	167.327 (15)	Ru7^xx^—Al21—Ru3	137.97 (2)
Al15—Ru3—Al23	82.61 (2)	Ru7^xx^—Al21—Al23	123.35 (2)
Si19^v^—Ru3—Al23	98.760 (15)	Ru3—Al21—Al23	60.742 (15)
Si19^vi^—Ru3—Al23	67.632 (14)	Ru7^xx^—Al21—Si16^v^	113.707 (19)
Al21^vii^—Ru3—Al23	123.873 (16)	Ru3—Al21—Si16^v^	103.294 (18)
Al21—Ru3—Al23	60.570 (14)	Al23—Al21—Si16^v^	104.90 (2)
Al26^v^—Ru3—Al23	167.328 (15)	Ru7^xx^—Al21—Al26^vi^	123.77 (2)
Al26^vi^—Ru3—Al23	111.862 (15)	Ru3—Al21—Al26^vi^	60.167 (15)
Al23^vii^—Ru3—Al23	67.20 (2)	Al23—Al21—Al26^vi^	111.01 (2)
Al31B—Ru4A—Al31B^vii^	30.1 (4)	Si16^v^—Al21—Al26^vi^	59.684 (17)
Al31B—Ru4A—Al15	141.68 (13)	Ru7^xx^—Al21—Si17^vii^	113.06 (2)
Al31B^vii^—Ru4A—Al15	141.68 (13)	Ru3—Al21—Si17^vii^	103.791 (18)
Al31B—Ru4A—Al31C^vii^	48.4 (5)	Al23—Al21—Si17^vii^	59.380 (16)
Al31B^vii^—Ru4A—Al31C^vii^	18.8 (3)	Si16^v^—Al21—Si17^vii^	55.701 (16)
Al15—Ru4A—Al31C^vii^	136.5 (3)	Al26^vi^—Al21—Si17^vii^	105.59 (2)
Al31B—Ru4A—Al31C	18.8 (3)	Ru7^xx^—Al21—Al30A^vii^	60.44 (5)
Al31B^vii^—Ru4A—Al31C	48.4 (5)	Ru3—Al21—Al30A^vii^	132.37 (5)
Al15—Ru4A—Al31C	136.5 (3)	Al23—Al21—Al30A^vii^	73.48 (6)
Al31C^vii^—Ru4A—Al31C	66.2 (7)	Si16^v^—Al21—Al30A^vii^	100.28 (3)
Al31B—Ru4A—Al29B^viii^	72.6 (4)	Al26^vi^—Al21—Al30A^vii^	159.92 (3)
Al31B^vii^—Ru4A—Al29B^viii^	88.7 (3)	Si17^vii^—Al21—Al30A^vii^	58.84 (3)
Al15—Ru4A—Al29B^viii^	128.2 (2)	Ru7^xx^—Al21—Al28^v^	62.275 (15)
Al31C^vii^—Ru4A—Al29B^viii^	94.8 (3)	Ru3—Al21—Al28^v^	129.35 (2)
Al31C—Ru4A—Al29B^viii^	60.1 (4)	Al23—Al21—Al28^v^	161.21 (2)
Al31B—Ru4A—Al29B^ix^	88.7 (3)	Si16^v^—Al21—Al28^v^	59.224 (16)
Al31B^vii^—Ru4A—Al29B^ix^	72.6 (4)	Al26^vi^—Al21—Al28^v^	71.198 (18)
Al15—Ru4A—Al29B^ix^	128.2 (2)	Si17^vii^—Al21—Al28^v^	101.86 (2)
Al31C^vii^—Ru4A—Al29B^ix^	60.1 (4)	Al30A^vii^—Al21—Al28^v^	98.28 (5)
Al31C—Ru4A—Al29B^ix^	94.8 (3)	Ru7^xx^—Al21—Al15	89.143 (19)
Al29B^viii^—Ru4A—Al29B^ix^	64.3 (3)	Ru3—Al21—Al15	50.129 (15)
Al31B—Ru4A—Al13	71.34 (15)	Al23—Al21—Al15	72.82 (2)
Al31B^vii^—Ru4A—Al13	71.34 (15)	Si16^v^—Al21—Al15	151.61 (2)
Al15—Ru4A—Al13	73.69 (5)	Al26^vi^—Al21—Al15	94.05 (2)
Al31C^vii^—Ru4A—Al13	78.6 (3)	Si17^vii^—Al21—Al15	132.05 (3)
Al31C—Ru4A—Al13	78.6 (3)	Al30A^vii^—Al21—Al15	105.85 (3)
Al29B^viii^—Ru4A—Al13	136.8 (3)	Al28^v^—Al21—Al15	125.96 (3)
Al29B^ix^—Ru4A—Al13	136.8 (3)	Ru7^xx^—Al21—Al28^viii^	62.811 (15)
Al31B—Ru4A—Si18^ix^	153.56 (16)	Ru3—Al21—Al28^viii^	90.535 (18)
Al31B^vii^—Ru4A—Si18^ix^	127.1 (2)	Al23—Al21—Al28^viii^	143.52 (2)
Al15—Ru4A—Si18^ix^	62.31 (7)	Si16^v^—Al21—Al28^viii^	103.27 (2)
Al31C^vii^—Ru4A—Si18^ix^	108.4 (4)	Al26^vi^—Al21—Al28^viii^	65.378 (17)
Al31C—Ru4A—Si18^ix^	158.6 (3)	Si17^vii^—Al21—Al28^viii^	156.51 (2)
Al29B^viii^—Ru4A—Si18^ix^	101.1 (3)	Al30A^vii^—Al21—Al28^viii^	123.23 (5)
Al29B^ix^—Ru4A—Si18^ix^	66.0 (2)	Al28^v^—Al21—Al28^viii^	55.06 (2)
Al13—Ru4A—Si18^ix^	121.64 (8)	Al15—Al21—Al28^viii^	71.34 (2)
Al31B—Ru4A—Si18^viii^	127.1 (2)	Si9A—Al22—Si9B	22.09 (6)
Al31B^vii^—Ru4A—Si18^viii^	153.56 (16)	Si9A—Al22—Ru5A	130.73 (6)
Al15—Ru4A—Si18^viii^	62.31 (7)	Si9B—Al22—Ru5A	108.84 (7)
Al31C^vii^—Ru4A—Si18^viii^	158.6 (3)	Si9A—Al22—Si10A	103.32 (3)
Al31C—Ru4A—Si18^viii^	108.4 (4)	Si9B—Al22—Si10A	93.38 (8)
Al29B^viii^—Ru4A—Si18^viii^	66.0 (2)	Ru5A—Al22—Si10A	56.05 (10)
Al29B^ix^—Ru4A—Si18^viii^	101.1 (3)	Si9A—Al22—Al32A^x^	111.57 (3)
Al13—Ru4A—Si18^viii^	121.64 (8)	Si9B—Al22—Al32A^x^	100.13 (4)
Si18^ix^—Ru4A—Si18^viii^	68.65 (6)	Ru5A—Al22—Al32A^x^	61.66 (6)
Al31B—Ru4A—Al27	81.93 (16)	Si10A—Al22—Al32A^x^	117.32 (9)
Al31B^vii^—Ru4A—Al27	107.8 (2)	Si9A—Al22—Ru5B	132.98 (12)
Al15—Ru4A—Al27	68.62 (6)	Si9B—Al22—Ru5B	110.98 (13)
Al31C^vii^—Ru4A—Al27	125.6 (4)	Ru5A—Al22—Ru5B	3.10 (10)
Al31C—Ru4A—Al27	69.2 (3)	Si10A—Al22—Ru5B	58.60 (13)
Al29B^viii^—Ru4A—Al27	88.75 (18)	Al32A^x^—Al22—Ru5B	59.40 (10)
Al29B^ix^—Ru4A—Al27	153.02 (17)	Si9A—Al22—Al24	158.31 (3)
Al13—Ru4A—Al27	63.279 (16)	Si9B—Al22—Al24	157.71 (5)
Si18^ix^—Ru4A—Al27	124.15 (11)	Ru5A—Al22—Al24	58.96 (3)
Si18^viii^—Ru4A—Al27	66.03 (4)	Si10A—Al22—Al24	64.35 (6)
Al31B—Ru4A—Al27^vii^	107.8 (2)	Al32A^x^—Al22—Al24	90.11 (2)
Al31B^vii^—Ru4A—Al27^vii^	81.93 (16)	Ru5B—Al22—Al24	57.77 (10)
Al15—Ru4A—Al27^vii^	68.62 (6)	Si9A—Al22—Al27^x^	101.59 (2)
Al31C^vii^—Ru4A—Al27^vii^	69.2 (3)	Si9B—Al22—Al27^x^	112.79 (4)
Al31C—Ru4A—Al27^vii^	125.6 (4)	Ru5A—Al22—Al27^x^	111.30 (7)
Al29B^viii^—Ru4A—Al27^vii^	153.02 (17)	Si10A—Al22—Al27^x^	153.82 (6)
Al29B^ix^—Ru4A—Al27^vii^	88.75 (18)	Al32A^x^—Al22—Al27^x^	59.17 (2)
Al13—Ru4A—Al27^vii^	63.280 (16)	Ru5B—Al22—Al27^x^	108.24 (13)
Si18^ix^—Ru4A—Al27^vii^	66.03 (4)	Al24—Al22—Al27^x^	89.47 (2)
Si18^viii^—Ru4A—Al27^vii^	124.15 (11)	Si9A—Al22—Al11	90.74 (3)
Al27—Ru4A—Al27^vii^	118.19 (6)	Si9B—Al22—Al11	103.79 (4)
Al31B—Ru4B—Al31B^vii^	33.6 (5)	Ru5A—Al22—Al11	110.59 (4)
Al31B—Ru4B—Al31C^vii^	53.9 (7)	Si10A—Al22—Al11	62.61 (12)
Al31B^vii^—Ru4B—Al31C^vii^	20.7 (4)	Al32A^x^—Al22—Al11	156.05 (3)
Al31B—Ru4B—Al31C	20.7 (4)	Ru5B—Al22—Al11	111.75 (8)
Al31B^vii^—Ru4B—Al31C	53.9 (7)	Al24—Al22—Al11	67.886 (18)
Al31C^vii^—Ru4B—Al31C	73.5 (10)	Al27^x^—Al22—Al11	109.29 (2)
Al31B—Ru4B—Al29B^viii^	78.9 (6)	Si9A—Al22—Al32B^x^	103.33 (10)
Al31B^vii^—Ru4B—Al29B^viii^	97.3 (8)	Si9B—Al22—Al32B^x^	87.15 (11)
Al31C^vii^—Ru4B—Al29B^viii^	103.3 (8)	Ru5A—Al22—Al32B^x^	53.28 (10)
Al31C—Ru4B—Al29B^viii^	64.5 (6)	Si10A—Al22—Al32B^x^	104.81 (14)
Al31B—Ru4B—Al29B^ix^	97.3 (8)	Al32A^x^—Al22—Al32B^x^	18.94 (9)
Al31B^vii^—Ru4B—Al29B^ix^	78.9 (6)	Ru5B—Al22—Al32B^x^	51.91 (12)
Al31C^vii^—Ru4B—Al29B^ix^	64.5 (6)	Al24—Al22—Al32B^x^	97.29 (10)
Al31C—Ru4B—Al29B^ix^	103.3 (8)	Al27^x^—Al22—Al32B^x^	76.92 (10)
Al29B^viii^—Ru4B—Al29B^ix^	67.1 (5)	Al11—Al22—Al32B^x^	163.34 (9)
Al31B—Ru4B—Al31A	19.0 (3)	Si9A—Al22—Al10B	101.92 (15)
Al31B^vii^—Ru4B—Al31A	19.0 (3)	Si9B—Al22—Al10B	89.6 (3)
Al31C^vii^—Ru4B—Al31A	39.4 (5)	Ru5A—Al22—Al10B	51.4 (3)
Al31C—Ru4B—Al31A	39.4 (5)	Si10A—Al22—Al10B	7.7 (4)
Al29B^viii^—Ru4B—Al31A	95.5 (7)	Al32A^x^—Al22—Al10B	111.6 (4)
Al29B^ix^—Ru4B—Al31A	95.5 (7)	Ru5B—Al22—Al10B	54.1 (3)
Al31B—Ru4B—Al13	75.4 (4)	Al24—Al22—Al10B	68.2 (3)
Al31B^vii^—Ru4B—Al13	75.4 (4)	Al27^x^—Al22—Al10B	156.49 (16)
Al31C^vii^—Ru4B—Al13	82.8 (5)	Al11—Al22—Al10B	70.0 (5)
Al31C—Ru4B—Al13	82.8 (5)	Al32B^x^—Al22—Al10B	98.0 (4)
Al29B^viii^—Ru4B—Al13	142.6 (5)	Si17^vii^—Al23—Al21	61.029 (17)
Al29B^ix^—Ru4B—Al13	142.6 (5)	Si17^vii^—Al23—Ru3	103.499 (18)
Al31A—Ru4B—Al13	65.7 (3)	Al21—Al23—Ru3	58.687 (14)
Al31B—Ru4B—Al29A^viii^	86.4 (6)	Si17^vii^—Al23—Ru6	100.099 (19)
Al31B^vii^—Ru4B—Al29A^viii^	103.4 (8)	Al21—Al23—Ru6	109.94 (2)
Al31C^vii^—Ru4B—Al29A^viii^	107.4 (8)	Ru3—Al23—Ru6	141.414 (19)
Al31C—Ru4B—Al29A^viii^	72.1 (5)	Si17^vii^—Al23—Si8	141.88 (2)
Al29B^viii^—Ru4B—Al29A^viii^	7.6 (2)	Al21—Al23—Si8	150.82 (2)
Al29B^ix^—Ru4B—Al29A^viii^	64.5 (3)	Ru3—Al23—Si8	112.858 (19)
Al31A—Ru4B—Al29A^viii^	102.8 (7)	Ru6—Al23—Si8	57.201 (13)
Al13—Ru4B—Al29A^viii^	148.2 (3)	Si17^vii^—Al23—Al10B	107.7 (3)
Al31B—Ru4B—Al29A^ix^	103.4 (8)	Al21—Al23—Al10B	139.1 (5)
Al31B^vii^—Ru4B—Al29A^ix^	86.4 (6)	Ru3—Al23—Al10B	90.5 (4)
Al31C^vii^—Ru4B—Al29A^ix^	72.1 (5)	Ru6—Al23—Al10B	110.8 (5)
Al31C—Ru4B—Al29A^ix^	107.4 (8)	Si8—Al23—Al10B	62.3 (5)
Al29B^viii^—Ru4B—Al29A^ix^	64.5 (3)	Si17^vii^—Al23—Si10A	111.40 (6)
Al29B^ix^—Ru4B—Al29A^ix^	7.6 (2)	Al21—Al23—Si10A	146.97 (12)
Al31A—Ru4B—Al29A^ix^	102.8 (7)	Ru3—Al23—Si10A	96.25 (9)
Al13—Ru4B—Al29A^ix^	148.2 (3)	Ru6—Al23—Si10A	103.01 (12)
Al29A^viii^—Ru4B—Al29A^ix^	61.0 (2)	Si8—Al23—Si10A	54.96 (11)
Al31B—Ru4B—Al27	85.1 (3)	Al10B—Al23—Si10A	7.9 (4)
Al31B^vii^—Ru4B—Al27	113.4 (5)	Si17^vii^—Al23—Ru5A	56.81 (5)
Al31C^vii^—Ru4B—Al27	132.4 (7)	Al21—Al23—Ru5A	108.53 (4)
Al31C—Ru4B—Al27	71.1 (4)	Ru3—Al23—Ru5A	106.29 (5)
Al29B^viii^—Ru4B—Al27	89.08 (17)	Ru6—Al23—Ru5A	112.15 (4)
Al29B^ix^—Ru4B—Al27	154.9 (4)	Si8—Al23—Ru5A	100.65 (4)
Al31A—Ru4B—Al27	94.5 (3)	Al10B—Al23—Ru5A	51.2 (2)
Al13—Ru4B—Al27	62.25 (17)	Si10A—Al23—Ru5A	54.60 (8)
Al29A^viii^—Ru4B—Al27	90.80 (10)	Si17^vii^—Al23—Ru5B	54.40 (11)
Al29A^ix^—Ru4B—Al27	149.4 (3)	Al21—Al23—Ru5B	107.46 (8)
Al31B—Ru4B—Al27^vii^	113.4 (5)	Ru3—Al23—Ru5B	108.24 (11)
Al31B^vii^—Ru4B—Al27^vii^	85.1 (3)	Ru6—Al23—Ru5B	110.30 (10)
Al31C^vii^—Ru4B—Al27^vii^	71.1 (4)	Si8—Al23—Ru5B	101.71 (8)
Al31C—Ru4B—Al27^vii^	132.4 (7)	Al10B—Al23—Ru5B	53.8 (2)
Al29B^viii^—Ru4B—Al27^vii^	154.9 (4)	Si10A—Al23—Ru5B	57.00 (12)
Al29B^ix^—Ru4B—Al27^vii^	89.08 (17)	Ru5A—Al23—Ru5B	2.98 (10)
Al31A—Ru4B—Al27^vii^	94.5 (3)	Si17^vii^—Al23—Al24	80.338 (18)
Al13—Ru4B—Al27^vii^	62.25 (17)	Al21—Al23—Al24	137.05 (2)
Al29A^viii^—Ru4B—Al27^vii^	149.4 (3)	Ru3—Al23—Al24	157.64 (2)
Al29A^ix^—Ru4B—Al27^vii^	90.80 (10)	Ru6—Al23—Al24	56.553 (13)
Al27—Ru4B—Al27^vii^	113.0 (5)	Si8—Al23—Al24	61.690 (16)
Al10B—Ru5A—Al32B^vi^	113.0 (3)	Al10B—Al23—Al24	67.6 (4)
Al10B—Ru5A—Al31C^vi^	85.0 (6)	Si10A—Al23—Al24	62.38 (8)
Al32B^vi^—Ru5A—Al31C^vi^	36.8 (4)	Ru5A—Al23—Al24	56.98 (4)
Al10B—Ru5A—Al32B^x^	121.0 (2)	Ru5B—Al23—Al24	55.77 (9)
Al32B^vi^—Ru5A—Al32B^x^	18.9 (2)	Si17^vii^—Al23—Si19^vi^	66.662 (17)
Al31C^vi^—Ru5A—Al32B^x^	55.1 (4)	Al21—Al23—Si19^vi^	72.882 (17)
Al10B—Ru5A—Al31B^vi^	66.8 (5)	Ru3—Al23—Si19^vi^	54.261 (13)
Al32B^vi^—Ru5A—Al31B^vi^	54.8 (2)	Ru6—Al23—Si19^vi^	163.70 (2)
Al31C^vi^—Ru5A—Al31B^vi^	19.1 (4)	Si8—Al23—Si19^vi^	127.53 (2)
Al32B^x^—Ru5A—Al31B^vi^	72.1 (2)	Al10B—Al23—Si19^vi^	67.0 (5)
Al10B—Ru5A—Si19^vi^	78.5 (5)	Si10A—Al23—Si19^vi^	74.84 (12)
Al32B^vi^—Ru5A—Si19^vi^	107.37 (12)	Ru5A—Al23—Si19^vi^	53.13 (4)
Al31C^vi^—Ru5A—Si19^vi^	78.9 (3)	Ru5B—Al23—Si19^vi^	54.65 (10)
Al32B^x^—Ru5A—Si19^vi^	124.43 (12)	Al24—Al23—Si19^vi^	109.956 (19)
Al31B^vi^—Ru5A—Si19^vi^	70.77 (16)	Si18^vii^—Al24—Ru6	108.644 (19)
Al10B—Ru5A—Si10A	8.2 (5)	Si18^vii^—Al24—Ru5B	110.23 (12)
Al32B^vi^—Ru5A—Si10A	115.65 (14)	Ru6—Al24—Ru5B	118.14 (8)
Al31C^vi^—Ru5A—Si10A	90.9 (4)	Si18^vii^—Al24—Ru5A	112.93 (6)
Al32B^x^—Ru5A—Si10A	120.89 (13)	Ru6—Al24—Ru5A	117.76 (3)
Al31B^vi^—Ru5A—Si10A	73.3 (2)	Ru5B—Al24—Ru5A	3.11 (10)
Si19^vi^—Ru5A—Si10A	85.24 (13)	Si18^vii^—Al24—Al20^vii^	59.685 (17)
Al10B—Ru5A—Si17^vii^	122.7 (2)	Ru6—Al24—Al20^vii^	59.851 (14)
Al32B^vi^—Ru5A—Si17^vii^	122.58 (15)	Ru5B—Al24—Al20^vii^	104.30 (12)
Al31C^vi^—Ru5A—Si17^vii^	134.1 (3)	Ru5A—Al24—Al20^vii^	106.90 (6)
Al32B^x^—Ru5A—Si17^vii^	116.12 (16)	Si18^vii^—Al24—Al22	92.44 (2)
Al31B^vi^—Ru5A—Si17^vii^	139.43 (14)	Ru6—Al24—Al22	156.41 (2)
Si19^vi^—Ru5A—Si17^vii^	73.14 (3)	Ru5B—Al24—Al22	60.94 (9)
Si10A—Ru5A—Si17^vii^	121.47 (7)	Ru5A—Al24—Al22	59.89 (3)
Al10B—Ru5A—Al29B^vii^	141.5 (6)	Al20^vii^—Al24—Al22	143.42 (2)
Al32B^vi^—Ru5A—Al29B^vii^	82.6 (4)	Si18^vii^—Al24—Al29A^vii^	59.24 (4)
Al31C^vi^—Ru5A—Al29B^vii^	119.1 (5)	Ru6—Al24—Al29A^vii^	112.61 (10)
Al32B^x^—Ru5A—Al29B^vii^	65.4 (4)	Ru5B—Al24—Al29A^vii^	56.43 (16)
Al31B^vi^—Ru5A—Al29B^vii^	137.3 (4)	Ru5A—Al24—Al29A^vii^	59.53 (11)
Si19^vi^—Ru5A—Al29B^vii^	132.33 (18)	Al20^vii^—Al24—Al29A^vii^	59.19 (11)
Si10A—Ru5A—Al29B^vii^	133.3 (2)	Al22—Al24—Al29A^vii^	86.96 (11)
Si17^vii^—Ru5A—Al29B^vii^	63.1 (3)	Si18^vii^—Al24—Ru7	58.295 (14)
Al10B—Ru5A—Al25^v^	137.7 (6)	Ru6—Al24—Ru7	118.408 (17)
Al32B^vi^—Ru5A—Al25^v^	66.26 (12)	Ru5B—Al24—Ru7	122.67 (8)
Al31C^vi^—Ru5A—Al25^v^	71.2 (3)	Ru5A—Al24—Ru7	122.40 (3)
Al32B^x^—Ru5A—Al25^v^	72.90 (12)	Al20^vii^—Al24—Ru7	111.042 (19)
Al31B^vi^—Ru5A—Al25^v^	83.44 (16)	Al22—Al24—Ru7	63.710 (14)
Si19^vi^—Ru5A—Al25^v^	63.15 (3)	Al29A^vii^—Al24—Ru7	107.81 (4)
Si10A—Ru5A—Al25^v^	145.72 (16)	Si18^vii^—Al24—Al26	90.35 (2)
Si17^vii^—Ru5A—Al25^v^	63.90 (5)	Ru6—Al24—Al26	61.627 (14)
Al29B^vii^—Ru5A—Al25^v^	80.65 (16)	Ru5B—Al24—Al26	156.80 (13)
Al10B—Ru5A—Al24	75.5 (6)	Ru5A—Al24—Al26	153.69 (7)
Al32B^vi^—Ru5A—Al24	123.97 (13)	Al20^vii^—Al24—Al26	95.19 (2)
Al31C^vi^—Ru5A—Al24	140.8 (3)	Al22—Al24—Al26	109.18 (2)
Al32B^x^—Ru5A—Al24	107.45 (13)	Al29A^vii^—Al24—Al26	146.73 (8)
Al31B^vi^—Ru5A—Al24	133.11 (16)	Ru7—Al24—Al26	58.949 (13)
Si19^vi^—Ru5A—Al24	128.12 (6)	Si18^vii^—Al24—Al23	145.37 (2)
Si10A—Ru5A—Al24	67.45 (12)	Ru6—Al24—Al23	58.819 (13)
Si17^vii^—Ru5A—Al24	84.51 (5)	Ru5B—Al24—Al23	61.68 (8)
Al29B^vii^—Ru5A—Al24	67.0 (2)	Ru5A—Al24—Al23	60.48 (4)
Al25^v^—Ru5A—Al24	142.69 (11)	Al20^vii^—Al24—Al23	88.534 (19)
Al10B—Ru5A—Al29A^vii^	137.2 (6)	Al22—Al24—Al23	108.79 (2)
Al32B^vi^—Ru5A—Al29A^vii^	89.86 (18)	Al29A^vii^—Al24—Al23	93.98 (3)
Al31C^vi^—Ru5A—Al29A^vii^	126.4 (4)	Ru7—Al24—Al23	155.98 (2)
Al32B^x^—Ru5A—Al29A^vii^	72.55 (17)	Al26—Al24—Al23	107.13 (2)
Al31B^vi^—Ru5A—Al29A^vii^	144.5 (2)	Si18^vii^—Al24—Si8	146.78 (2)
Si19^vi^—Ru5A—Al29A^vii^	130.35 (8)	Ru6—Al24—Si8	56.304 (14)
Si10A—Ru5A—Al29A^vii^	129.07 (15)	Ru5B—Al24—Si8	102.75 (12)
Si17^vii^—Ru5A—Al29A^vii^	58.77 (8)	Ru5A—Al24—Si8	99.93 (6)
Al29B^vii^—Ru5A—Al29A^vii^	7.3 (2)	Al20^vii^—Al24—Si8	116.11 (2)
Al25^v^—Ru5A—Al29A^vii^	84.03 (10)	Al22—Al24—Si8	100.20 (2)
Al24—Ru5A—Al29A^vii^	61.83 (10)	Al29A^vii^—Al24—Si8	151.16 (3)
Al32B^vi^—Ru5B—Al32B^x^	19.2 (2)	Ru7—Al24—Si8	100.331 (19)
Al32B^vi^—Ru5B—Al29B^vii^	85.9 (4)	Al26—Al24—Si8	56.507 (18)
Al32B^x^—Ru5B—Al29B^vii^	68.5 (4)	Al23—Al24—Si8	57.219 (18)
Al32B^vi^—Ru5B—Si17^vii^	126.7 (2)	Si9A^ix^—Al25—Ru5B^xvi^	128.55 (13)
Al32B^x^—Ru5B—Si17^vii^	121.3 (3)	Si9A^ix^—Al25—Si9B^ix^	21.90 (6)
Al29B^vii^—Ru5B—Si17^vii^	66.4 (3)	Ru5B^xvi^—Al25—Si9B^ix^	106.70 (14)
Al32B^vi^—Ru5B—Al31C^vi^	36.2 (4)	Si9A^ix^—Al25—Ru6^vii^	114.15 (3)
Al32B^x^—Ru5B—Al31C^vi^	54.7 (4)	Ru5B^xvi^—Al25—Ru6^vii^	115.92 (12)
Al29B^vii^—Ru5B—Al31C^vi^	121.9 (5)	Si9B^ix^—Al25—Ru6^vii^	135.61 (6)
Si17^vii^—Ru5B—Al31C^vi^	134.6 (3)	Si9A^ix^—Al25—Ru5A^xvi^	131.44 (7)
Al32B^vi^—Ru5B—Al10B	108.8 (4)	Ru5B^xvi^—Al25—Ru5A^xvi^	3.09 (11)
Al32B^x^—Ru5B—Al10B	117.8 (3)	Si9B^ix^—Al25—Ru5A^xvi^	109.56 (8)
Al29B^vii^—Ru5B—Al10B	142.6 (6)	Ru6^vii^—Al25—Ru5A^xvi^	113.37 (6)
Si17^vii^—Ru5B—Al10B	120.9 (2)	Si9A^ix^—Al25—Al32A^vii^	110.33 (3)
Al31C^vi^—Ru5B—Al10B	80.8 (6)	Ru5B^xvi^—Al25—Al32A^vii^	61.68 (10)
Al32B^vi^—Ru5B—Al29A^vii^	93.6 (2)	Si9B^ix^—Al25—Al32A^vii^	100.19 (5)
Al32B^x^—Ru5B—Al29A^vii^	76.0 (2)	Ru6^vii^—Al25—Al32A^vii^	110.16 (3)
Al29B^vii^—Ru5B—Al29A^vii^	7.7 (2)	Ru5A^xvi^—Al25—Al32A^vii^	60.43 (4)
Si17^vii^—Ru5B—Al29A^vii^	61.77 (15)	Si9A^ix^—Al25—Si19^vii^	164.51 (3)
Al31C^vi^—Ru5B—Al29A^vii^	129.6 (4)	Ru5B^xvi^—Al25—Si19^vii^	58.62 (11)
Al10B—Ru5B—Al29A^vii^	138.2 (6)	Si9B^ix^—Al25—Si19^vii^	159.82 (6)
Al32B^vi^—Ru5B—Si19^vi^	105.1 (2)	Ru6^vii^—Al25—Si19^vii^	57.349 (14)
Al32B^x^—Ru5B—Si19^vi^	123.0 (2)	Ru5A^xvi^—Al25—Si19^vii^	56.18 (5)
Al29B^vii^—Ru5B—Si19^vi^	135.8 (3)	Al32A^vii^—Al25—Si19^vii^	85.14 (2)
Si17^vii^—Ru5B—Si19^vi^	73.17 (10)	Si9A^ix^—Al25—Al27^vii^	99.80 (3)
Al31C^vi^—Ru5B—Si19^vi^	76.0 (3)	Ru5B^xvi^—Al25—Al27^vii^	112.82 (13)
Al10B—Ru5B—Si19^vi^	74.8 (5)	Si9B^ix^—Al25—Al27^vii^	111.92 (5)
Al29A^vii^—Ru5B—Si19^vi^	133.56 (19)	Ru6^vii^—Al25—Al27^vii^	62.571 (15)
Al32B^vi^—Ru5B—Al25^v^	67.10 (14)	Ru5A^xvi^—Al25—Al27^vii^	110.28 (5)
Al32B^x^—Ru5B—Al25^v^	74.45 (15)	Al32A^vii^—Al25—Al27^vii^	59.01 (2)
Al29B^vii^—Ru5B—Al25^v^	84.3 (2)	Si19^vii^—Al25—Al27^vii^	87.59 (2)
Si17^vii^—Ru5B—Al25^v^	65.67 (11)	Si9A^ix^—Al25—Si17^xv^	104.45 (3)
Al31C^vi^—Ru5B—Al25^v^	70.8 (3)	Ru5B^xvi^—Al25—Si17^xv^	55.05 (10)
Al10B—Ru5B—Al25^v^	133.0 (6)	Si9B^ix^—Al25—Si17^xv^	93.09 (5)
Al29A^vii^—Ru5B—Al25^v^	87.84 (18)	Ru6^vii^—Al25—Si17^xv^	100.998 (18)
Si19^vi^—Ru5B—Al25^v^	62.70 (9)	Ru5A^xvi^—Al25—Si17^xv^	56.63 (4)
Al32B^vi^—Ru5B—Al31B^vi^	53.4 (2)	Al32A^vii^—Al25—Si17^xv^	116.53 (3)
Al32B^x^—Ru5B—Al31B^vi^	70.9 (2)	Si19^vii^—Al25—Si17^xv^	67.345 (17)
Al29B^vii^—Ru5B—Al31B^vi^	139.2 (4)	Al27^vii^—Al25—Si17^xv^	154.93 (2)
Si17^vii^—Ru5B—Al31B^vi^	137.7 (2)	Si9A^ix^—Al25—Al32B^vii^	102.03 (10)
Al31C^vi^—Ru5B—Al31B^vi^	18.3 (3)	Ru5B^xvi^—Al25—Al32B^vii^	52.82 (12)
Al10B—Ru5B—Al31B^vi^	63.3 (5)	Si9B^ix^—Al25—Al32B^vii^	87.00 (11)
Al29A^vii^—Ru5B—Al31B^vi^	146.8 (3)	Ru6^vii^—Al25—Al32B^vii^	128.48 (10)
Si19^vi^—Ru5B—Al31B^vi^	67.8 (2)	Ru5A^xvi^—Al25—Al32B^vii^	52.63 (10)
Al25^v^—Ru5B—Al31B^vi^	82.13 (19)	Al32A^vii^—Al25—Al32B^vii^	18.95 (9)
Al32B^vi^—Ru5B—Al24	126.01 (19)	Si19^vii^—Al25—Al32B^vii^	92.89 (10)
Al32B^x^—Ru5B—Al24	110.14 (19)	Al27^vii^—Al25—Al32B^vii^	76.56 (10)
Al29B^vii^—Ru5B—Al24	69.6 (3)	Si17^xv^—Al25—Al32B^vii^	104.12 (10)
Si17^vii^—Ru5B—Al24	86.99 (15)	Si9A^ix^—Al25—Si16	97.04 (3)
Al31C^vi^—Ru5B—Al24	138.4 (3)	Ru5B^xvi^—Al25—Si16	101.30 (8)
Al10B—Ru5B—Al24	74.1 (6)	Si9B^ix^—Al25—Si16	105.52 (5)
Al29A^vii^—Ru5B—Al24	64.23 (16)	Ru6^vii^—Al25—Si16	55.609 (13)
Si19^vi^—Ru5B—Al24	126.23 (15)	Ru5A^xvi^—Al25—Si16	101.37 (3)
Al25^v^—Ru5B—Al24	148.3 (3)	Al32A^vii^—Al25—Si16	152.62 (3)
Al31B^vi^—Ru5B—Al24	129.4 (2)	Si19^vii^—Al25—Si16	67.479 (16)
Al32B^vi^—Ru5B—Si10A	111.8 (2)	Al27^vii^—Al25—Si16	117.52 (2)
Al32B^x^—Ru5B—Si10A	118.1 (2)	Si17^xv^—Al25—Si16	53.440 (15)
Al29B^vii^—Ru5B—Si10A	134.7 (3)	Al32B^vii^—Al25—Si16	153.98 (10)
Si17^vii^—Ru5B—Si10A	120.13 (14)	Si9A^ix^—Al25—Al22^xxi^	55.52 (2)
Al31C^vi^—Ru5B—Si10A	86.6 (4)	Ru5B^xvi^—Al25—Al22^xxi^	110.56 (8)
Al10B—Ru5B—Si10A	7.9 (4)	Si9B^ix^—Al25—Al22^xxi^	56.81 (5)
Al29A^vii^—Ru5B—Si10A	130.4 (2)	Ru6^vii^—Al25—Al22^xxi^	114.61 (2)
Si19^vi^—Ru5B—Si10A	81.33 (19)	Ru5A^xvi^—Al25—Al22^xxi^	110.96 (3)
Al25^v^—Ru5B—Si10A	140.8 (3)	Al32A^vii^—Al25—Al22^xxi^	58.16 (2)
Al31B^vi^—Ru5B—Si10A	69.6 (2)	Si19^vii^—Al25—Al22^xxi^	138.51 (2)
Al24—Ru5B—Si10A	66.34 (15)	Al27^vii^—Al25—Al22^xxi^	58.274 (17)
Si16^vii^—Ru6—Si19	73.752 (14)	Si17^xv^—Al25—Al22^xxi^	143.73 (2)
Si16^vii^—Ru6—Si8	119.473 (16)	Al32B^vii^—Al25—Al22^xxi^	58.96 (10)
Si19—Ru6—Si8	84.882 (16)	Si16—Al25—Al22^xxi^	146.74 (2)
Si16^vii^—Ru6—Al24	85.523 (14)	Ru3^xv^—Al26—Si16^vii^	102.244 (18)
Si19—Ru6—Al24	129.919 (14)	Ru3^xv^—Al26—Al21^xv^	58.455 (14)
Si8—Ru6—Al24	66.403 (16)	Si16^vii^—Al26—Al21^xv^	59.966 (17)
Si16^vii^—Ru6—Al25^vii^	65.368 (14)	Ru3^xv^—Al26—Si8	95.784 (19)
Si19—Ru6—Al25^vii^	63.124 (14)	Si16^vii^—Al26—Si8	111.61 (2)
Si8—Ru6—Al25^vii^	145.654 (17)	Al21^xv^—Al26—Si8	146.06 (2)
Al24—Ru6—Al25^vii^	144.281 (16)	Ru3^xv^—Al26—Ru7	141.451 (19)
Si16^vii^—Ru6—Al20^vii^	59.439 (14)	Si16^vii^—Al26—Ru7	99.520 (18)
Si19—Ru6—Al20^vii^	131.436 (15)	Al21^xv^—Al26—Ru7	108.583 (19)
Si8—Ru6—Al20^vii^	128.011 (16)	Si8—Al26—Ru7	105.23 (2)
Al24—Ru6—Al20^vii^	61.662 (15)	Ru3^xv^—Al26—Al11	113.311 (19)
Al25^vii^—Ru6—Al20^vii^	84.991 (15)	Si16^vii^—Al26—Al11	144.17 (2)
Si16^vii^—Ru6—Al23	147.194 (14)	Al21^xv^—Al26—Al11	145.26 (2)
Si19—Ru6—Al23	135.764 (15)	Si8—Al26—Al11	61.90 (2)
Si8—Ru6—Al23	62.146 (17)	Ru7—Al26—Al11	54.412 (12)
Al24—Ru6—Al23	64.628 (14)	Ru3^xv^—Al26—Ru6	105.140 (16)
Al25^vii^—Ru6—Al23	134.258 (15)	Si16^vii^—Al26—Ru6	55.329 (13)
Al20^vii^—Ru6—Al23	92.788 (15)	Al21^xv^—Al26—Ru6	106.047 (19)
Si16^vii^—Ru6—Al13	133.510 (18)	Si8—Al26—Ru6	56.299 (13)
Si19—Ru6—Al13	59.769 (18)	Ru7—Al26—Ru6	113.409 (17)
Si8—Ru6—Al13	58.452 (17)	Al11—Al26—Ru6	108.61 (2)
Al24—Ru6—Al13	122.930 (17)	Ru3^xv^—Al26—Al24	155.88 (2)
Al25^vii^—Ru6—Al13	92.680 (17)	Si16^vii^—Al26—Al24	79.141 (18)
Al20^vii^—Ru6—Al13	163.833 (17)	Al21^xv^—Al26—Al24	135.83 (2)
Al23—Ru6—Al13	77.385 (18)	Si8—Al26—Al24	62.192 (16)
Si16^vii^—Ru6—Al26	59.727 (14)	Ru7—Al26—Al24	59.871 (13)
Si19—Ru6—Al26	67.301 (13)	Al11—Al26—Al24	66.696 (18)
Si8—Ru6—Al26	59.762 (16)	Ru6—Al26—Al24	55.497 (13)
Al24—Ru6—Al26	62.875 (14)	Ru3^xv^—Al26—Al26^vii^	56.970 (11)
Al25^vii^—Ru6—Al26	113.153 (15)	Si16^vii^—Al26—Al26^vii^	151.308 (13)
Al20^vii^—Ru6—Al26	97.033 (15)	Al21^xv^—Al26—Al26^vii^	112.414 (15)
Al23—Ru6—Al26	112.461 (14)	Si8—Al26—Al26^vii^	57.481 (12)
Al13—Ru6—Al26	98.582 (17)	Ru7—Al26—Al26^vii^	108.889 (11)
Si16^vii^—Ru6—Al27	125.610 (15)	Al11—Al26—Al26^vii^	58.656 (12)
Si19—Ru6—Al27	89.805 (15)	Ru6—Al26—Al26^vii^	107.552 (10)
Si8—Ru6—Al27	109.764 (16)	Al24—Al26—Al26^vii^	111.536 (14)
Al24—Ru6—Al27	137.383 (16)	Ru4A—Al27—Al30A^vii^	166.95 (10)
Al25^vii^—Ru6—Al27	61.026 (15)	Ru4A—Al27—Ru4B	5.8 (4)
Al20^vii^—Ru6—Al27	106.387 (15)	Al30A^vii^—Al27—Ru4B	172.7 (5)
Al23—Ru6—Al27	76.151 (15)	Ru4A—Al27—Al32A	65.45 (6)
Al13—Ru6—Al27	59.067 (16)	Al30A^vii^—Al27—Al32A	126.77 (9)
Al26—Ru6—Al27	154.792 (15)	Ru4B—Al27—Al32A	60.3 (4)
Si16^vii^—Ru6—Al30A^vii^	102.70 (7)	Ru4A—Al27—Al13	57.67 (2)
Si19—Ru6—Al30A^vii^	132.20 (5)	Al30A^vii^—Al27—Al13	122.47 (7)
Si8—Ru6—Al30A^vii^	131.46 (8)	Ru4B—Al27—Al13	56.81 (15)
Al24—Ru6—Al30A^vii^	96.30 (4)	Al32A—Al27—Al13	88.32 (3)
Al25^vii^—Ru6—Al30A^vii^	72.10 (7)	Ru4A—Al27—Al22^xx^	113.48 (4)
Al20^vii^—Ru6—Al30A^vii^	55.35 (3)	Al30A^vii^—Al27—Al22^xx^	74.19 (9)
Al23—Ru6—Al30A^vii^	69.43 (8)	Ru4B—Al27—Al22^xx^	110.7 (3)
Al13—Ru6—Al30A^vii^	108.73 (4)	Al32A—Al27—Al22^xx^	59.81 (2)
Al26—Ru6—Al30A^vii^	152.08 (3)	Al13—Al27—Al22^xx^	144.86 (3)
Al27—Ru6—Al30A^vii^	52.71 (2)	Ru4A—Al27—Ru7^xx^	112.40 (5)
Al21^x^—Ru7—Al11	141.649 (18)	Al30A^vii^—Al27—Ru7^xx^	60.33 (2)
Al21^x^—Ru7—Si18^vii^	91.431 (15)	Ru4B—Al27—Ru7^xx^	116.2 (3)
Al11—Ru7—Si18^vii^	126.820 (18)	Al32A—Al27—Ru7^xx^	113.89 (2)
Al21^x^—Ru7—Al30A^i^	65.17 (9)	Al13—Al27—Ru7^xx^	150.38 (3)
Al11—Ru7—Al30A^i^	89.79 (9)	Al22^xx^—Al27—Ru7^xx^	64.048 (15)
Si18^vii^—Ru7—Al30A^i^	119.80 (5)	Ru4A—Al27—Al25^vii^	115.47 (7)
Al21^x^—Ru7—Al27^x^	74.643 (15)	Al30A^vii^—Al27—Al25^vii^	77.24 (6)
Al11—Ru7—Al27^x^	116.895 (19)	Ru4B—Al27—Al25^vii^	109.8 (5)
Si18^vii^—Ru7—Al27^x^	63.540 (14)	Al32A—Al27—Al25^vii^	59.34 (2)
Al30A^i^—Ru7—Al27^x^	57.08 (4)	Al13—Al27—Al25^vii^	89.33 (2)
Al21^x^—Ru7—Al30B^i^	75.60 (15)	Al22^xx^—Al27—Al25^vii^	62.742 (18)
Al11—Ru7—Al30B^i^	79.40 (15)	Ru7^xx^—Al27—Al25^vii^	118.42 (2)
Si18^vii^—Ru7—Al30B^i^	124.40 (8)	Ru4A—Al27—Al30B^vii^	172.03 (8)
Al30A^i^—Ru7—Al30B^i^	10.77 (8)	Al30A^vii^—Al27—Al30B^vii^	10.21 (7)
Al27^x^—Ru7—Al30B^i^	60.87 (9)	Ru4B—Al27—Al30B^vii^	174.26 (18)
Al21^x^—Ru7—Al26	126.897 (15)	Al32A—Al27—Al30B^vii^	116.91 (14)
Al11—Ru7—Al26	63.063 (18)	Al13—Al27—Al30B^vii^	128.85 (10)
Si18^vii^—Ru7—Al26	90.562 (14)	Al22^xx^—Al27—Al30B^vii^	64.32 (14)
Al30A^i^—Ru7—Al26	148.47 (7)	Ru7^xx^—Al27—Al30B^vii^	59.63 (7)
Al27^x^—Ru7—Al26	148.316 (15)	Al25^vii^—Al27—Al30B^vii^	70.90 (10)
Al30B^i^—Ru7—Al26	140.15 (13)	Ru4A—Al27—Ru6	115.40 (3)
Al21^x^—Ru7—Al28^xi^	64.563 (16)	Al30A^vii^—Al27—Ru6	68.21 (4)
Al11—Ru7—Al28^xi^	95.882 (19)	Ru4B—Al27—Ru6	113.4 (2)
Si18^vii^—Ru7—Al28^xi^	116.253 (15)	Al32A—Al27—Ru6	105.41 (2)
Al30A^i^—Ru7—Al28^xi^	102.54 (4)	Al13—Al27—Ru6	58.283 (15)
Al27^x^—Ru7—Al28^xi^	139.202 (16)	Al22^xx^—Al27—Ru6	113.12 (2)
Al30B^i^—Ru7—Al28^xi^	106.22 (9)	Ru7^xx^—Al27—Ru6	127.19 (2)
Al26—Ru7—Al28^xi^	67.242 (15)	Al25^vii^—Al27—Ru6	56.403 (14)
Al21^x^—Ru7—Al24	147.730 (15)	Al30B^vii^—Al27—Ru6	71.86 (8)
Al11—Ru7—Al24	70.529 (17)	Ru4A—Al27—Si18^viii^	56.67 (4)
Si18^vii^—Ru7—Al24	56.314 (14)	Al30A^vii^—Al27—Si18^viii^	115.33 (3)
Al30A^i^—Ru7—Al24	127.09 (6)	Ru4B—Al27—Si18^viii^	60.5 (3)
Al27^x^—Ru7—Al24	88.245 (15)	Al32A—Al27—Si18^viii^	88.48 (2)
Al30B^i^—Ru7—Al24	119.75 (13)	Al13—Al27—Si18^viii^	108.54 (2)
Al26—Ru7—Al24	61.180 (14)	Al22^xx^—Al27—Si18^viii^	86.554 (19)
Al28^xi^—Ru7—Al24	127.289 (15)	Ru7^xx^—Al27—Si18^viii^	55.752 (13)
Al21^x^—Ru7—Al28^vii^	66.535 (15)	Al25^vii^—Al27—Si18^viii^	143.16 (2)
Al11—Ru7—Al28^vii^	131.540 (19)	Al30B^vii^—Al27—Si18^viii^	115.37 (7)
Si18^vii^—Ru7—Al28^vii^	59.617 (14)	Ru6—Al27—Si18^viii^	159.70 (2)
Al30A^i^—Ru7—Al28^vii^	131.67 (9)	Ru4A—Al27—Al15	54.37 (6)
Al27^x^—Ru7—Al28^vii^	107.887 (16)	Al30A^vii^—Al27—Al15	112.87 (9)
Al30B^i^—Ru7—Al28^vii^	142.13 (15)	Ru4B—Al27—Al15	59.8 (4)
Al26—Ru7—Al28^vii^	69.242 (15)	Al32A—Al27—Al15	119.51 (3)
Al28^xi^—Ru7—Al28^vii^	56.639 (19)	Al13—Al27—Al15	65.91 (2)
Al24—Ru7—Al28^vii^	94.190 (14)	Al22^xx^—Al27—Al15	140.86 (3)
Al21^x^—Ru7—Al22	127.209 (14)	Ru7^xx^—Al27—Al15	85.55 (2)
Al11—Ru7—Al22	60.831 (18)	Al25^vii^—Al27—Al15	155.08 (3)
Si18^vii^—Ru7—Al22	87.294 (15)	Al30B^vii^—Al27—Al15	121.91 (13)
Al30A^i^—Ru7—Al22	70.12 (7)	Ru6—Al27—Al15	104.73 (2)
Al27^x^—Ru7—Al22	57.854 (15)	Si18^viii^—Al27—Al15	55.05 (2)
Al30B^i^—Ru7—Al22	62.53 (12)	Ru2^ix^—Al28—Al28^xiv^	165.46 (3)
Al26—Ru7—Al22	105.892 (14)	Ru2^ix^—Al28—Si16	57.410 (14)
Al28^xi^—Ru7—Al22	154.796 (16)	Al28^xiv^—Al28—Si16	109.86 (3)
Al24—Ru7—Al22	57.266 (13)	Ru2^ix^—Al28—Si18	57.930 (14)
Al28^vii^—Ru7—Al22	145.875 (15)	Al28^xiv^—Al28—Si18	114.58 (3)
Si10A—Si8—Al13	134.29 (9)	Si16—Al28—Si18	75.902 (18)
Si10A—Si8—Ru6	112.83 (3)	Ru2^ix^—Al28—Ru7^xxii^	123.54 (2)
Al13—Si8—Ru6	62.754 (12)	Al28^xiv^—Al28—Ru7^xxii^	63.76 (2)
Si10A—Si8—Ru6^vii^	112.83 (3)	Si16—Al28—Ru7^xxii^	104.017 (19)
Al13—Si8—Ru6^vii^	62.754 (12)	Si18—Al28—Ru7^xxii^	178.26 (2)
Ru6—Si8—Ru6^vii^	124.23 (2)	Ru2^ix^—Al28—Al14^xiv^	59.956 (18)
Si10A—Si8—Al26^vii^	113.89 (8)	Al28^xiv^—Al28—Al14^xiv^	127.43 (3)
Al13—Si8—Al26^vii^	104.28 (2)	Si16—Al28—Al14^xiv^	77.13 (2)
Ru6—Si8—Al26^vii^	120.73 (2)	Si18—Al28—Al14^xiv^	117.52 (2)
Ru6^vii^—Si8—Al26^vii^	63.940 (13)	Ru7^xxii^—Al28—Al14^xiv^	64.053 (17)
Si10A—Si8—Al26	113.90 (8)	Ru2^ix^—Al28—Al21^xvi^	107.85 (2)
Al13—Si8—Al26	104.28 (2)	Al28^xiv^—Al28—Al21^xvi^	65.21 (2)
Ru6—Si8—Al26	63.940 (13)	Si16—Al28—Al21^xvi^	57.251 (16)
Ru6^vii^—Si8—Al26	120.73 (2)	Si18—Al28—Al21^xvi^	125.93 (2)
Al26^vii^—Si8—Al26	65.04 (2)	Ru7^xxii^—Al28—Al21^xvi^	53.162 (13)
Si10A—Si8—Al23^vii^	63.42 (7)	Al14^xiv^—Al28—Al21^xvi^	79.051 (19)
Al13—Si8—Al23^vii^	78.61 (2)	Ru2^ix^—Al28—Ru7^vii^	111.722 (19)
Ru6—Si8—Al23^vii^	117.74 (2)	Al28^xiv^—Al28—Ru7^vii^	59.60 (2)
Ru6^vii^—Si8—Al23^vii^	60.654 (13)	Si16—Al28—Ru7^vii^	94.790 (19)
Al26^vii^—Si8—Al23^vii^	114.682 (15)	Si18—Al28—Ru7^vii^	54.988 (14)
Al26—Si8—Al23^vii^	177.11 (3)	Ru7^xxii^—Al28—Ru7^vii^	123.361 (19)
Si10A—Si8—Al23	63.42 (7)	Al14^xiv^—Al28—Ru7^vii^	170.62 (2)
Al13—Si8—Al23	78.61 (2)	Al21^xvi^—Al28—Ru7^vii^	100.73 (2)
Ru6—Si8—Al23	60.653 (13)	Ru2^ix^—Al28—Al21^xvii^	125.63 (2)
Ru6^vii^—Si8—Al23	117.74 (2)	Al28^xiv^—Al28—Al21^xvii^	59.73 (2)
Al26^vii^—Si8—Al23	177.11 (3)	Si16—Al28—Al21^xvii^	145.22 (2)
Al26—Si8—Al23	114.681 (15)	Si18—Al28—Al21^xvii^	79.480 (18)
Al23^vii^—Si8—Al23	65.44 (2)	Ru7^xxii^—Al28—Al21^xvii^	99.854 (18)
Si10A—Si8—Al11	64.29 (9)	Al14^xiv^—Al28—Al21^xvii^	136.84 (3)
Al13—Si8—Al11	161.43 (3)	Al21^xvi^—Al28—Al21^xvii^	124.94 (2)
Ru6—Si8—Al11	113.799 (13)	Ru7^vii^—Al28—Al21^xvii^	50.654 (12)
Ru6^vii^—Si8—Al11	113.799 (13)	Ru5B^vii^—Al29A—Ru4B^ii^	124.8 (3)
Al26^vii^—Si8—Al11	60.621 (19)	Ru5B^vii^—Al29A—Si17	58.02 (9)
Al26—Si8—Al11	60.621 (19)	Ru4B^ii^—Al29A—Si17	164.2 (5)
Al23^vii^—Si8—Al11	116.60 (2)	Ru5B^vii^—Al29A—Al32A^xvii^	63.08 (13)
Al23—Si8—Al11	116.60 (2)	Ru4B^ii^—Al29A—Al32A^xvii^	62.9 (3)
Si10A—Si8—Al10B	4.9 (2)	Si17—Al29A—Al32A^xvii^	113.93 (14)
Al13—Si8—Al10B	129.4 (3)	Ru5B^vii^—Al29A—Al29A^xviii^	155.59 (12)
Ru6—Si8—Al10B	111.37 (10)	Ru4B^ii^—Al29A—Al29A^xviii^	59.50 (11)
Ru6^vii^—Si8—Al10B	111.37 (10)	Si17—Al29A—Al29A^xviii^	110.99 (3)
Al26^vii^—Si8—Al10B	117.8 (2)	Al32A^xvii^—Al29A—Al29A^xviii^	110.11 (4)
Al26—Si8—Al10B	117.8 (2)	Ru5B^vii^—Al29A—Ru1^ix^	114.81 (10)
Al23^vii^—Si8—Al10B	59.6 (2)	Ru4B^ii^—Al29A—Ru1^ix^	118.55 (12)
Al23—Si8—Al10B	59.6 (2)	Si17—Al29A—Ru1^ix^	56.86 (6)
Al11—Si8—Al10B	69.2 (3)	Al32A^xvii^—Al29A—Ru1^ix^	154.35 (16)
Si10A—Si8—Al24^vii^	64.91 (3)	Al29A^xviii^—Al29A—Ru1^ix^	60.23 (4)
Al13—Si8—Al24^vii^	118.294 (14)	Ru5B^vii^—Al29A—Si18	110.29 (9)
Ru6—Si8—Al24^vii^	177.69 (3)	Ru4B^ii^—Al29A—Si18	64.4 (5)
Ru6^vii^—Si8—Al24^vii^	57.293 (10)	Si17—Al29A—Si18	130.88 (19)
Al26^vii^—Si8—Al24^vii^	61.302 (15)	Al32A^xvii^—Al29A—Si18	94.94 (7)
Al26—Si8—Al24^vii^	117.14 (2)	Al29A^xviii^—Al29A—Si18	93.31 (3)
Al23^vii^—Si8—Al24^vii^	61.091 (15)	Ru1^ix^—Al29A—Si18	108.8 (2)
Al23—Si8—Al24^vii^	117.26 (2)	Ru5B^vii^—Al29A—Ru4A^ii^	126.87 (19)
Al11—Si8—Al24^vii^	65.928 (15)	Ru4B^ii^—Al29A—Ru4A^ii^	5.9 (4)
Al10B—Si8—Al24^vii^	66.34 (10)	Si17—Al29A—Ru4A^ii^	169.12 (12)
Si9B—Si9A—Al22^vii^	82.44 (11)	Al32A^xvii^—Al29A—Ru4A^ii^	66.40 (11)
Si9B—Si9A—Al22	82.44 (11)	Al29A^xviii^—Al29A—Ru4A^ii^	60.32 (4)
Al22^vii^—Si9A—Al22	76.04 (3)	Ru1^ix^—Al29A—Ru4A^ii^	117.76 (8)
Si9B—Si9A—Al12	78.41 (14)	Si18—Al29A—Ru4A^ii^	58.74 (7)
Al22^vii^—Si9A—Al12	137.27 (3)	Ru5B^vii^—Al29A—Al29B^xviii^	150.30 (15)
Al22—Si9A—Al12	137.26 (3)	Ru4B^ii^—Al29A—Al29B^xviii^	55.6 (2)
Si9B—Si9A—Al25^ii^	80.16 (5)	Si17—Al29A—Al29B^xviii^	113.27 (16)
Al22^vii^—Si9A—Al25^ii^	67.335 (17)	Al32A^xvii^—Al29A—Al29B^xviii^	102.94 (18)
Al22—Si9A—Al25^ii^	141.05 (4)	Al29A^xviii^—Al29A—Al29B^xviii^	7.53 (19)
Al12—Si9A—Al25^ii^	71.93 (2)	Ru1^ix^—Al29A—Al29B^xviii^	65.7 (2)
Si9B—Si9A—Al25^i^	80.16 (5)	Si18—Al29A—Al29B^xviii^	96.40 (12)
Al22^vii^—Si9A—Al25^i^	141.05 (4)	Ru4A^ii^—Al29A—Al29B^xviii^	57.23 (17)
Al22—Si9A—Al25^i^	67.334 (17)	Ru5B^vii^—Al29A—Al20	106.68 (14)
Al12—Si9A—Al25^i^	71.93 (2)	Ru4B^ii^—Al29A—Al20	112.8 (5)
Al25^ii^—Si9A—Al25^i^	141.54 (4)	Si17—Al29A—Al20	78.27 (13)
Si9B—Si9A—Al30B^i^	146.67 (10)	Al32A^xvii^—Al29A—Al20	148.12 (6)
Al22^vii^—Si9A—Al30B^i^	103.95 (10)	Al29A^xviii^—Al29A—Al20	90.59 (3)
Al22—Si9A—Al30B^i^	67.98 (7)	Ru1^ix^—Al29A—Al20	57.28 (11)
Al12—Si9A—Al30B^i^	113.01 (9)	Si18—Al29A—Al20	58.77 (11)
Al25^ii^—Si9A—Al30B^i^	132.77 (9)	Ru4A^ii^—Al29A—Al20	107.26 (14)
Al25^i^—Si9A—Al30B^i^	74.62 (10)	Al29B^xviii^—Al29A—Al20	98.07 (17)
Si9B—Si9A—Al30B^ii^	146.67 (10)	Ru5B^vii^—Al29A—Ru5A^vii^	0.72 (11)
Al22^vii^—Si9A—Al30B^ii^	67.98 (7)	Ru4B^ii^—Al29A—Ru5A^vii^	124.7 (3)
Al22—Si9A—Al30B^ii^	103.95 (10)	Si17—Al29A—Ru5A^vii^	58.34 (4)
Al12—Si9A—Al30B^ii^	113.01 (9)	Al32A^xvii^—Al29A—Ru5A^vii^	63.15 (10)
Al25^ii^—Si9A—Al30B^ii^	74.62 (10)	Al29A^xviii^—Al29A—Ru5A^vii^	156.31 (8)
Al25^i^—Si9A—Al30B^ii^	132.77 (9)	Ru1^ix^—Al29A—Ru5A^vii^	115.09 (6)
Al30B^i^—Si9A—Al30B^ii^	60.00 (18)	Si18—Al29A—Ru5A^vii^	109.57 (5)
Si9A—Si9B—Al12	79.39 (14)	Ru4A^ii^—Al29A—Ru5A^vii^	126.69 (16)
Si9A—Si9B—Al22^vii^	75.46 (12)	Al29B^xviii^—Al29A—Ru5A^vii^	150.99 (13)
Al12—Si9B—Al22^vii^	134.37 (7)	Al20—Al29A—Ru5A^vii^	106.24 (11)
Si9A—Si9B—Al22	75.46 (12)	Al32A^xvii^—Al29B—Ru5B^vii^	69.7 (3)
Al12—Si9B—Al22	134.37 (7)	Al32A^xvii^—Al29B—Ru4B^ii^	69.5 (4)
Al22^vii^—Si9B—Al22	73.94 (6)	Ru5B^vii^—Al29B—Ru4B^ii^	136.4 (6)
Si9A—Si9B—Al25^ii^	77.95 (7)	Al32A^xvii^—Al29B—Ru4A^ii^	72.7 (3)
Al12—Si9B—Al25^ii^	71.75 (5)	Ru5B^vii^—Al29B—Ru4A^ii^	137.2 (5)
Al22^vii^—Si9B—Al25^ii^	66.16 (3)	Ru4B^ii^—Al29B—Ru4A^ii^	5.9 (4)
Al22—Si9B—Al25^ii^	136.32 (9)	Al32A^xvii^—Al29B—Al31C^xvii^	21.2 (4)
Si9A—Si9B—Al25^i^	77.95 (7)	Ru5B^vii^—Al29B—Al31C^xvii^	88.6 (6)
Al12—Si9B—Al25^i^	71.75 (5)	Ru4B^ii^—Al29B—Al31C^xvii^	55.2 (6)
Al22^vii^—Si9B—Al25^i^	136.32 (9)	Ru4A^ii^—Al29B—Al31C^xvii^	59.8 (4)
Al22—Si9B—Al25^i^	66.16 (3)	Al32A^xvii^—Al29B—Ru5A^vii^	69.4 (3)
Al25^ii^—Si9B—Al25^i^	139.17 (10)	Ru5B^vii^—Al29B—Ru5A^vii^	0.67 (12)
Si8—Si10A—Ru5A^vii^	113.04 (8)	Ru4B^ii^—Al29B—Ru5A^vii^	135.9 (5)
Si8—Si10A—Ru5A	113.04 (8)	Ru4A^ii^—Al29B—Ru5A^vii^	136.6 (5)
Ru5A^vii^—Si10A—Ru5A	125.3 (3)	Al31C^xvii^—Al29B—Ru5A^vii^	88.4 (5)
Si8—Si10A—Al31A^vi^	163.35 (15)	Al32A^xvii^—Al29B—Al29A^xviii^	118.8 (3)
Ru5A^vii^—Si10A—Al31A^vi^	63.66 (12)	Ru5B^vii^—Al29B—Al29A^xviii^	160.4 (3)
Ru5A—Si10A—Al31A^vi^	63.66 (12)	Ru4B^ii^—Al29B—Al29A^xviii^	59.88 (17)
Si8—Si10A—Ru5B	111.39 (12)	Ru4A^ii^—Al29B—Al29A^xviii^	60.44 (13)
Ru5A^vii^—Si10A—Ru5B	127.3 (3)	Al31C^xvii^—Al29B—Al29A^xviii^	97.8 (4)
Ru5A—Si10A—Ru5B	2.00 (11)	Ru5A^vii^—Al29B—Al29A^xviii^	161.1 (3)
Al31A^vi^—Si10A—Ru5B	65.58 (16)	Al32A^xvii^—Al29B—Si17	121.8 (3)
Si8—Si10A—Ru5B^vii^	111.39 (12)	Ru5B^vii^—Al29B—Si17	57.81 (15)
Ru5A^vii^—Si10A—Ru5B^vii^	2.00 (11)	Ru4B^ii^—Al29B—Si17	165.4 (4)
Ru5A—Si10A—Ru5B^vii^	127.3 (3)	Ru4A^ii^—Al29B—Si17	164.7 (5)
Al31A^vi^—Si10A—Ru5B^vii^	65.58 (16)	Al31C^xvii^—Al29B—Si17	132.2 (5)
Ru5B—Si10A—Ru5B^vii^	129.3 (3)	Ru5A^vii^—Al29B—Si17	58.37 (12)
Si8—Si10A—Al22^vii^	109.66 (18)	Al29A^xviii^—Al29B—Si17	105.5 (3)
Ru5A^vii^—Si10A—Al22^vii^	61.84 (4)	Al32A^xvii^—Al29B—Al29B^xviii^	111.79 (14)
Ru5A—Si10A—Al22^vii^	124.32 (7)	Ru5B^vii^—Al29B—Al29B^xviii^	158.92 (15)
Al31A^vi^—Si10A—Al22^vii^	83.66 (8)	Ru4B^ii^—Al29B—Al29B^xviii^	56.5 (2)
Ru5B—Si10A—Al22^vii^	124.72 (10)	Ru4A^ii^—Al29B—Al29B^xviii^	57.86 (17)
Ru5B^vii^—Si10A—Al22^vii^	61.04 (8)	Al31C^xvii^—Al29B—Al29B^xviii^	90.7 (4)
Si8—Si10A—Al22	109.66 (18)	Ru5A^vii^—Al29B—Al29B^xviii^	159.53 (12)
Ru5A^vii^—Si10A—Al22	124.32 (7)	Al29A^xviii^—Al29B—Al29B^xviii^	7.53 (19)
Ru5A—Si10A—Al22	61.84 (4)	Si17—Al29B—Al29B^xviii^	108.96 (16)
Al31A^vi^—Si10A—Al22	83.66 (8)	Al32A^xvii^—Al29B—Al32B^xvii^	18.05 (14)
Ru5B—Si10A—Al22	61.04 (8)	Ru5B^vii^—Al29B—Al32B^xvii^	55.3 (3)
Ru5B^vii^—Si10A—Al22	124.72 (10)	Ru4B^ii^—Al29B—Al32B^xvii^	86.7 (5)
Al22^vii^—Si10A—Al22	71.31 (5)	Ru4A^ii^—Al29B—Al32B^xvii^	90.3 (4)
Si8—Si10A—Al23^vii^	61.62 (5)	Al31C^xvii^—Al29B—Al32B^xvii^	33.4 (4)
Ru5A^vii^—Si10A—Al23^vii^	62.95 (5)	Ru5A^vii^—Al29B—Al32B^xvii^	55.1 (2)
Ru5A—Si10A—Al23^vii^	119.35 (13)	Al29A^xviii^—Al29B—Al32B^xvii^	128.2 (3)
Al31A^vi^—Si10A—Al23^vii^	104.74 (17)	Si17—Al29B—Al32B^xvii^	103.9 (3)
Ru5B—Si10A—Al23^vii^	119.82 (14)	Al29B^xviii^—Al29B—Al32B^xvii^	120.67 (17)
Ru5B^vii^—Si10A—Al23^vii^	62.35 (9)	Al32A^xvii^—Al29B—Si18	98.6 (3)
Al22^vii^—Si10A—Al23^vii^	111.61 (3)	Ru5B^vii^—Al29B—Si18	108.7 (2)
Al22—Si10A—Al23^vii^	171.2 (2)	Ru4B^ii^—Al29B—Si18	63.5 (5)
Si8—Si10A—Al23	61.62 (5)	Ru4A^ii^—Al29B—Si18	57.63 (13)
Ru5A^vii^—Si10A—Al23	119.35 (13)	Al31C^xvii^—Al29B—Si18	102.0 (4)
Ru5A—Si10A—Al23	62.95 (5)	Ru5A^vii^—Al29B—Si18	108.11 (18)
Al31A^vi^—Si10A—Al23	104.74 (17)	Al29A^xviii^—Al29B—Si18	88.2 (2)
Ru5B—Si10A—Al23	62.35 (9)	Si17—Al29B—Si18	119.6 (5)
Ru5B^vii^—Si10A—Al23	119.82 (14)	Al29B^xviii^—Al29B—Si18	92.09 (12)
Al22^vii^—Si10A—Al23	171.2 (2)	Al32B^xvii^—Al29B—Si18	112.4 (3)
Al22—Si10A—Al23	111.61 (3)	Al32A^xvii^—Al29B—Ru1^ix^	162.7 (3)
Al23^vii^—Si10A—Al23	64.26 (3)	Ru5B^vii^—Al29B—Ru1^ix^	110.0 (3)
Si8—Si10A—Al11	62.60 (13)	Ru4B^ii^—Al29B—Ru1^ix^	113.5 (4)
Ru5A^vii^—Si10A—Al11	113.16 (11)	Ru4A^ii^—Al29B—Ru1^ix^	112.0 (3)
Ru5A—Si10A—Al11	113.16 (11)	Al31C^xvii^—Al29B—Ru1^ix^	146.8 (4)
Al31A^vi^—Si10A—Al11	134.05 (5)	Ru5A^vii^—Al29B—Ru1^ix^	110.5 (3)
Ru5B—Si10A—Al11	111.49 (14)	Al29A^xviii^—Al29B—Ru1^ix^	56.21 (15)
Ru5B^vii^—Si10A—Al11	111.49 (14)	Si17—Al29B—Ru1^ix^	52.7 (2)
Al22^vii^—Si10A—Al11	59.88 (9)	Al29B^xviii^—Al29B—Ru1^ix^	61.96 (12)
Al22—Si10A—Al11	59.88 (9)	Al32B^xvii^—Al29B—Ru1^ix^	149.2 (2)
Al23^vii^—Si10A—Al11	113.80 (16)	Si18—Al29B—Ru1^ix^	97.8 (4)
Al23—Si10A—Al11	113.80 (16)	Al30B—Al30A—Al27^vii^	106.2 (4)
Al31A^vi^—Al10B—Ru5A^vii^	71.4 (5)	Al30B—Al30A—Ru1^ix^	80.6 (4)
Al31A^vi^—Al10B—Ru5A	71.4 (5)	Al27^vii^—Al30A—Ru1^ix^	168.32 (11)
Ru5A^vii^—Al10B—Ru5A	141.5 (11)	Al30B—Al30A—Al20	87.8 (4)
Al31A^vi^—Al10B—Ru5B	73.0 (5)	Al27^vii^—Al30A—Al20	112.19 (5)
Ru5A^vii^—Al10B—Ru5B	143.2 (11)	Ru1^ix^—Al30A—Al20	57.91 (2)
Ru5A—Al10B—Ru5B	1.72 (12)	Al30B—Al30A—Ru7^xxi^	91.6 (4)
Al31A^vi^—Al10B—Ru5B^vii^	73.0 (5)	Al27^vii^—Al30A—Ru7^xxi^	62.59 (3)
Ru5A^vii^—Al10B—Ru5B^vii^	1.72 (12)	Ru1^ix^—Al30A—Ru7^xxi^	127.54 (4)
Ru5A—Al10B—Ru5B^vii^	143.2 (11)	Al20—Al30A—Ru7^xxi^	174.32 (4)
Ru5B—Al10B—Ru5B^vii^	144.9 (11)	Al30B—Al30A—Si17	135.5 (5)
Al31A^vi^—Al10B—Al31B^v^	16.12 (19)	Al27^vii^—Al30A—Si17	118.23 (16)
Ru5A^vii^—Al10B—Al31B^v^	58.7 (4)	Ru1^ix^—Al30A—Si17	55.77 (3)
Ru5A—Al10B—Al31B^v^	86.1 (7)	Al20—Al30A—Si17	76.97 (5)
Ru5B—Al10B—Al31B^v^	87.6 (6)	Ru7^xxi^—Al30A—Si17	107.23 (10)
Ru5B^vii^—Al10B—Al31B^v^	60.2 (4)	Al30B—Al30A—Al21^vii^	143.3 (4)
Al31A^vi^—Al10B—Al31B^vi^	16.12 (19)	Al27^vii^—Al30A—Al21^vii^	72.38 (8)
Ru5A^vii^—Al10B—Al31B^vi^	86.1 (7)	Ru1^ix^—Al30A—Al21^vii^	107.95 (8)
Ru5A—Al10B—Al31B^vi^	58.7 (4)	Al20—Al30A—Al21^vii^	127.46 (14)
Ru5B—Al10B—Al31B^vi^	60.2 (4)	Ru7^xxi^—Al30A—Al21^vii^	54.39 (5)
Ru5B^vii^—Al10B—Al31B^vi^	87.6 (6)	Si17—Al30A—Al21^vii^	58.36 (8)
Al31B^v^—Al10B—Al31B^vi^	27.7 (4)	Al30B—Al30A—Al14^ix^	73.0 (4)
Al31A^vi^—Al10B—Al23^vii^	115.5 (7)	Al27^vii^—Al30A—Al14^ix^	127.62 (4)
Ru5A^vii^—Al10B—Al23^vii^	64.74 (13)	Ru1^ix^—Al30A—Al14^ix^	63.04 (4)
Ru5A—Al10B—Al23^vii^	125.3 (4)	Al20—Al30A—Al14^ix^	120.03 (7)
Ru5B—Al10B—Al23^vii^	125.3 (4)	Ru7^xxi^—Al30A—Al14^ix^	65.06 (3)
Ru5B^vii^—Al10B—Al23^vii^	64.05 (14)	Si17—Al30A—Al14^ix^	79.00 (4)
Al31B^v^—Al10B—Al23^vii^	112.9 (6)	Al21^vii^—Al30A—Al14^ix^	79.39 (4)
Al31B^vi^—Al10B—Al23^vii^	130.1 (8)	Al30B—Al30A—Ru6^vii^	114.5 (4)
Al31A^vi^—Al10B—Al23	115.5 (7)	Al27^vii^—Al30A—Ru6^vii^	59.08 (4)
Ru5A^vii^—Al10B—Al23	125.3 (4)	Ru1^ix^—Al30A—Ru6^vii^	109.64 (4)
Ru5A—Al10B—Al23	64.74 (13)	Al20—Al30A—Ru6^vii^	54.97 (3)
Ru5B—Al10B—Al23	64.05 (14)	Ru7^xxi^—Al30A—Ru6^vii^	120.50 (8)
Ru5B^vii^—Al10B—Al23	125.3 (4)	Si17—Al30A—Ru6^vii^	90.49 (10)
Al31B^v^—Al10B—Al23	130.1 (8)	Al21^vii^—Al30A—Ru6^vii^	96.54 (12)
Al31B^vi^—Al10B—Al23	112.9 (6)	Al14^ix^—Al30A—Ru6^vii^	169.37 (11)
Al23^vii^—Al10B—Al23	64.31 (19)	Al30A—Al30B—Ru1^ix^	88.0 (5)
Al31A^vi^—Al10B—Al22^vii^	88.0 (3)	Al30A—Al30B—Al20	81.1 (4)
Ru5A^vii^—Al10B—Al22^vii^	61.92 (12)	Ru1^ix^—Al30B—Al20	57.90 (7)
Ru5A—Al10B—Al22^vii^	126.1 (3)	Al30A—Al30B—Al30B^xviii^	144.1 (4)
Ru5B—Al10B—Al22^vii^	126.1 (3)	Ru1^ix^—Al30B—Al30B^xviii^	58.35 (17)
Ru5B^vii^—Al10B—Al22^vii^	61.19 (13)	Al20—Al30B—Al30B^xviii^	89.59 (13)
Al31B^v^—Al10B—Al22^vii^	73.5 (3)	Al30A—Al30B—Si9A^ix^	151.0 (5)
Al31B^vi^—Al10B—Al22^vii^	89.1 (3)	Ru1^ix^—Al30B—Si9A^ix^	105.2 (2)
Al23^vii^—Al10B—Al22^vii^	108.6 (3)	Al20—Al30B—Si9A^ix^	84.30 (13)
Al23—Al10B—Al22^vii^	156.4 (10)	Al30B^xviii^—Al30B—Si9A^ix^	60.00 (9)
Al31A^vi^—Al10B—Al22	88.0 (3)	Al30A—Al30B—Al14^ix^	96.5 (5)
Ru5A^vii^—Al10B—Al22	126.1 (3)	Ru1^ix^—Al30B—Al14^ix^	64.97 (8)
Ru5A—Al10B—Al22	61.92 (12)	Al20—Al30B—Al14^ix^	122.86 (13)
Ru5B—Al10B—Al22	61.19 (13)	Al30B^xviii^—Al30B—Al14^ix^	60.05 (14)
Ru5B^vii^—Al10B—Al22	126.1 (3)	Si9A^ix^—Al30B—Al14^ix^	112.4 (2)
Al31B^v^—Al10B—Al22	89.1 (3)	Al30A—Al30B—Ru7^xxi^	77.7 (4)
Al31B^vi^—Al10B—Al22	73.5 (3)	Ru1^ix^—Al30B—Ru7^xxi^	126.25 (15)
Al23^vii^—Al10B—Al22	156.4 (10)	Al20—Al30B—Ru7^xxi^	158.1 (3)
Al23—Al10B—Al22	108.6 (3)	Al30B^xviii^—Al30B—Ru7^xxi^	110.95 (13)
Al22^vii^—Al10B—Al22	68.3 (3)	Si9A^ix^—Al30B—Ru7^xxi^	112.11 (19)
Ru7—Al11—Ru7^vii^	134.87 (3)	Al14^ix^—Al30B—Ru7^xxi^	65.67 (7)
Ru7—Al11—Al26^vii^	119.96 (3)	Al30A—Al30B—Al27^vii^	63.6 (4)
Ru7^vii^—Al11—Al26^vii^	62.525 (13)	Ru1^ix^—Al30B—Al27^vii^	150.0 (3)
Ru7—Al11—Al26	62.525 (14)	Al20—Al30B—Al27^vii^	105.66 (16)
Ru7^vii^—Al11—Al26	119.96 (3)	Al30B^xviii^—Al30B—Al27^vii^	151.45 (14)
Al26^vii^—Al11—Al26	62.69 (2)	Si9A^ix^—Al30B—Al27^vii^	96.97 (13)
Ru7—Al11—Al22^vii^	130.54 (3)	Al14^ix^—Al30B—Al27^vii^	124.23 (15)
Ru7^vii^—Al11—Al22^vii^	66.435 (13)	Ru7^xxi^—Al30B—Al27^vii^	59.49 (8)
Al26^vii^—Al11—Al22^vii^	109.406 (16)	Al30A—Al30B—Al22^xxi^	118.9 (5)
Al26—Al11—Al22^vii^	157.46 (3)	Ru1^ix^—Al30B—Al22^xxi^	152.7 (3)
Ru7—Al11—Al22	66.435 (13)	Al20—Al30B—Al22^xxi^	126.71 (16)
Ru7^vii^—Al11—Al22	130.54 (3)	Al30B^xviii^—Al30B—Al22^xxi^	94.41 (13)
Al26^vii^—Al11—Al22	157.46 (3)	Si9A^ix^—Al30B—Al22^xxi^	53.74 (12)
Al26—Al11—Al22	109.406 (16)	Al14^ix^—Al30B—Al22^xxi^	104.43 (15)
Al22^vii^—Al11—Al22	69.28 (3)	Ru7^xxi^—Al30B—Al22^xxi^	61.37 (7)
Ru7—Al11—Al14	67.460 (14)	Al27^vii^—Al30B—Al22^xxi^	57.09 (7)
Ru7^vii^—Al11—Al14	67.460 (14)	Al31B^vii^—Al31A—Al31B	104.6 (8)
Al26^vii^—Al11—Al14	95.15 (2)	Al31B^vii^—Al31A—Al31C	111.1 (6)
Al26—Al11—Al14	95.15 (2)	Al31B—Al31A—Al31C	7.5 (6)
Al22^vii^—Al11—Al14	106.85 (2)	Al31B^vii^—Al31A—Al31C^vii^	7.5 (6)
Al22—Al11—Al14	106.85 (2)	Al31B—Al31A—Al31C^vii^	111.1 (6)
Ru7—Al11—Si8	108.159 (15)	Al31C—Al31A—Al31C^vii^	117.4 (8)
Ru7^vii^—Al11—Si8	108.160 (15)	Al31B^vii^—Al31A—Al10B^xv^	113.3 (4)
Al26^vii^—Al11—Si8	57.477 (18)	Al31B—Al31A—Al10B^xv^	113.3 (4)
Al26—Al11—Si8	57.478 (18)	Al31C—Al31A—Al10B^xv^	112.4 (4)
Al22^vii^—Al11—Si8	100.13 (2)	Al31C^vii^—Al31A—Al10B^xv^	112.4 (4)
Al22—Al11—Si8	100.13 (2)	Al31B^vii^—Al31A—Ru4B	63.1 (4)
Al14—Al11—Si8	147.02 (3)	Al31B—Al31A—Ru4B	63.1 (4)
Ru7—Al11—Si10A	111.479 (16)	Al31C—Al31A—Ru4B	65.0 (4)
Ru7^vii^—Al11—Si10A	111.479 (16)	Al31C^vii^—Al31A—Ru4B	65.0 (4)
Al26^vii^—Al11—Si10A	102.01 (5)	Al10B^xv^—Al31A—Ru4B	172.6 (4)
Al26—Al11—Si10A	102.01 (5)	Al31B^vii^—Al31A—Al32A^vii^	8.9 (4)
Al22^vii^—Al11—Si10A	57.51 (4)	Al31B—Al31A—Al32A^vii^	112.1 (4)
Al22—Al11—Si10A	57.51 (4)	Al31C—Al31A—Al32A^vii^	118.2 (4)
Al14—Al11—Si10A	159.87 (6)	Al31C^vii^—Al31A—Al32A^vii^	1.6 (4)
Si8—Al11—Si10A	53.11 (5)	Al10B^xv^—Al31A—Al32A^vii^	112.72 (12)
Ru1^iv^—Al12—Si9B	105.42 (6)	Ru4B—Al31A—Al32A^vii^	64.82 (9)
Ru1^iv^—Al12—Si9A	127.62 (3)	Al31B^vii^—Al31A—Al32A	112.1 (4)
Si9B—Al12—Si9A	22.19 (6)	Al31B—Al31A—Al32A	8.9 (4)
Ru1^iv^—Al12—Ru2	117.87 (3)	Al31C—Al31A—Al32A	1.6 (4)
Si9B—Al12—Ru2	136.71 (7)	Al31C^vii^—Al31A—Al32A	118.2 (4)
Si9A—Al12—Ru2	114.52 (3)	Al10B^xv^—Al31A—Al32A	112.72 (12)
Ru1^iv^—Al12—Si16^ii^	105.283 (19)	Ru4B—Al31A—Al32A	64.82 (9)
Si9B—Al12—Si16^ii^	111.35 (3)	Al32A^vii^—Al31A—Al32A	119.08 (6)
Si9A—Al12—Si16^ii^	101.29 (2)	Al31B^vii^—Al31A—Si10A^xv^	111.3 (4)
Ru2—Al12—Si16^ii^	58.581 (15)	Al31B—Al31A—Si10A^xv^	111.3 (4)
Ru1^iv^—Al12—Si16^i^	105.283 (19)	Al31C—Al31A—Si10A^xv^	110.9 (4)
Si9B—Al12—Si16^i^	111.35 (3)	Al31C^vii^—Al31A—Si10A^xv^	110.9 (4)
Si9A—Al12—Si16^i^	101.29 (2)	Al10B^xv^—Al31A—Si10A^xv^	3.7 (3)
Ru2—Al12—Si16^i^	58.581 (15)	Ru4B—Al31A—Si10A^xv^	168.9 (3)
Si16^ii^—Al12—Si16^i^	117.10 (3)	Al32A^vii^—Al31A—Si10A^xv^	111.36 (4)
Ru1^iv^—Al12—Si17^xii^	56.847 (15)	Al32A—Al31A—Si10A^xv^	111.36 (4)
Si9B—Al12—Si17^xii^	96.74 (4)	Al31B^vii^—Al31A—Si19^vii^	97.6 (4)
Si9A—Al12—Si17^xii^	108.10 (2)	Al31B—Al31A—Si19^vii^	148.1 (4)
Ru2—Al12—Si17^xii^	106.339 (18)	Al31C—Al31A—Si19^vii^	140.9 (4)
Si16^ii^—Al12—Si17^xii^	56.184 (13)	Al31C^vii^—Al31A—Si19^vii^	90.2 (4)
Si16^i^—Al12—Si17^xii^	150.58 (3)	Al10B^xv^—Al31A—Si19^vii^	77.3 (3)
Ru1^iv^—Al12—Si17^xiii^	56.847 (15)	Ru4B—Al31A—Si19^vii^	109.3 (2)
Si9B—Al12—Si17^xiii^	96.74 (4)	Al32A^vii^—Al31A—Si19^vii^	88.77 (2)
Si9A—Al12—Si17^xiii^	108.10 (2)	Al32A—Al31A—Si19^vii^	139.39 (4)
Ru2—Al12—Si17^xiii^	106.339 (18)	Si10A^xv^—Al31A—Si19^vii^	80.56 (6)
Si16^ii^—Al12—Si17^xiii^	150.58 (3)	Al31B^vii^—Al31A—Si19	148.1 (4)
Si16^i^—Al12—Si17^xiii^	56.184 (13)	Al31B—Al31A—Si19	97.6 (4)
Si17^xii^—Al12—Si17^xiii^	113.59 (3)	Al31C—Al31A—Si19	90.2 (4)
Ru1^iv^—Al12—Al14^iv^	60.71 (2)	Al31C^vii^—Al31A—Si19	140.9 (4)
Si9B—Al12—Al14^iv^	166.13 (7)	Al10B^xv^—Al31A—Si19	77.3 (3)
Si9A—Al12—Al14^iv^	171.68 (4)	Ru4B—Al31A—Si19	109.3 (2)
Ru2—Al12—Al14^iv^	57.157 (19)	Al32A^vii^—Al31A—Si19	139.39 (4)
Si16^ii^—Al12—Al14^iv^	74.706 (18)	Al32A—Al31A—Si19	88.77 (2)
Si16^i^—Al12—Al14^iv^	74.706 (18)	Si10A^xv^—Al31A—Si19	80.55 (6)
Si17^xii^—Al12—Al14^iv^	75.984 (18)	Si19^vii^—Al31A—Si19	53.85 (3)
Si17^xiii^—Al12—Al14^iv^	75.984 (18)	Al31B^vii^—Al31A—Ru5A^xv^	155.4 (4)
Ru4B—Al13—Ru4A	6.2 (4)	Al31B—Al31A—Ru5A^xv^	66.3 (4)
Ru4B—Al13—Si8	164.9 (5)	Al31C—Al31A—Ru5A^xv^	61.9 (4)
Ru4A—Al13—Si8	158.75 (8)	Al31C^vii^—Al31A—Ru5A^xv^	161.0 (4)
Ru4B—Al13—Si19^vii^	109.2 (4)	Al10B^xv^—Al31A—Ru5A^xv^	56.37 (5)
Ru4A—Al13—Si19^vii^	114.51 (6)	Ru4B—Al31A—Ru5A^xv^	123.97 (4)
Si8—Al13—Si19^vii^	84.06 (2)	Al32A^vii^—Al31A—Ru5A^xv^	162.40 (5)
Ru4B—Al13—Si19	109.2 (4)	Al32A—Al31A—Ru5A^xv^	61.54 (4)
Ru4A—Al13—Si19	114.51 (6)	Si10A^xv^—Al31A—Ru5A^xv^	56.78 (4)
Si8—Al13—Si19	84.06 (2)	Si19^vii^—Al31A—Ru5A^xv^	100.90 (5)
Si19^vii^—Al13—Si19	55.57 (2)	Si19—Al31A—Ru5A^xv^	55.81 (3)
Ru4B—Al13—Ru6^vii^	121.61 (3)	Al31A—Al31B—Al31C	165.1 (12)
Ru4A—Al13—Ru6^vii^	121.078 (15)	Al31A—Al31B—Al31B^vii^	37.7 (4)
Si8—Al13—Ru6^vii^	58.794 (12)	Al31C—Al31B—Al31B^vii^	154.5 (10)
Si19^vii^—Al13—Ru6^vii^	57.940 (13)	Al31A—Al31B—Al32A	166.9 (6)
Si19—Al13—Ru6^vii^	105.19 (2)	Al31C—Al31B—Al32A	2.0 (9)
Ru4B—Al13—Ru6	121.61 (3)	Al31B^vii^—Al31B—Al32A	152.61 (19)
Ru4A—Al13—Ru6	121.078 (15)	Al31A—Al31B—Al31C^vii^	47.2 (5)
Si8—Al13—Ru6	58.794 (12)	Al31C—Al31B—Al31C^vii^	144.7 (13)
Si19^vii^—Al13—Ru6	105.19 (2)	Al31B^vii^—Al31B—Al31C^vii^	9.9 (4)
Si19—Al13—Ru6	57.941 (13)	Al32A—Al31B—Al31C^vii^	142.7 (5)
Ru6^vii^—Al13—Ru6	116.50 (2)	Al31A—Al31B—Ru4B	97.9 (5)
Ru4B—Al13—Al27	60.94 (17)	Al31C—Al31B—Ru4B	85.6 (10)
Ru4A—Al13—Al27	59.05 (2)	Al31B^vii^—Al31B—Ru4B	73.2 (3)
Si8—Al13—Al27	113.14 (2)	Al32A—Al31B—Ru4B	84.3 (3)
Si19^vii^—Al13—Al27	141.60 (3)	Al31C^vii^—Al31B—Ru4B	65.2 (4)
Si19—Al13—Al27	90.892 (17)	Al31A—Al31B—Al32B	156.6 (6)
Ru6^vii^—Al13—Al27	160.34 (3)	Al31C—Al31B—Al32B	20.9 (9)
Ru6—Al13—Al27	62.650 (11)	Al31B^vii^—Al31B—Al32B	158.02 (18)
Ru4B—Al13—Al27^vii^	60.94 (17)	Al32A—Al31B—Al32B	21.53 (15)
Ru4A—Al13—Al27^vii^	59.05 (2)	Al31C^vii^—Al31B—Al32B	151.1 (5)
Si8—Al13—Al27^vii^	113.14 (2)	Ru4B—Al31B—Al32B	104.7 (4)
Si19^vii^—Al13—Al27^vii^	90.892 (16)	Al31A—Al31B—Ru4A	98.4 (5)
Si19—Al13—Al27^vii^	141.60 (3)	Al31C—Al31B—Ru4A	84.4 (10)
Ru6^vii^—Al13—Al27^vii^	62.650 (11)	Al31B^vii^—Al31B—Ru4A	74.96 (19)
Ru6—Al13—Al27^vii^	160.34 (3)	Al32A—Al31B—Ru4A	83.2 (3)
Al27—Al13—Al27^vii^	110.94 (3)	Al31C^vii^—Al31B—Ru4A	67.2 (4)
Ru4B—Al13—Al31A	55.7 (5)	Ru4B—Al31B—Ru4A	2.6 (2)
Ru4A—Al13—Al31A	61.84 (7)	Al32B—Al31B—Ru4A	103.9 (3)
Si8—Al13—Al31A	139.41 (4)	Al31A—Al31B—Ru5A^xv^	96.4 (4)
Si19^vii^—Al13—Al31A	60.45 (3)	Al31C—Al31B—Ru5A^xv^	74.8 (10)
Si19—Al13—Al31A	60.45 (3)	Al31B^vii^—Al31B—Ru5A^xv^	130.0 (2)
Ru6^vii^—Al13—Al31A	109.49 (2)	Al32A—Al31B—Ru5A^xv^	76.5 (2)
Ru6—Al13—Al31A	109.49 (2)	Al31C^vii^—Al31B—Ru5A^xv^	139.5 (5)
Al27—Al13—Al31A	88.14 (2)	Ru4B—Al31B—Ru5A^xv^	151.4 (4)
Al27^vii^—Al13—Al31A	88.14 (2)	Al32B—Al31B—Ru5A^xv^	60.6 (2)
Ru4B—Al13—Al31B	47.2 (5)	Ru4A—Al31B—Ru5A^xv^	148.9 (3)
Ru4A—Al13—Al31B	53.11 (14)	Al31A—Al31B—Ru5B^xv^	98.5 (5)
Si8—Al13—Al31B	146.96 (12)	Al31C—Al31B—Ru5B^xv^	72.6 (10)
Si19^vii^—Al13—Al31B	75.54 (16)	Al31B^vii^—Al31B—Ru5B^xv^	132.1 (2)
Si19—Al13—Al31B	62.99 (11)	Al32A—Al31B—Ru5B^xv^	74.3 (2)
Ru6^vii^—Al13—Al31B	125.08 (16)	Al31C^vii^—Al31B—Ru5B^xv^	141.5 (5)
Ru6—Al13—Al31B	101.66 (13)	Ru4B—Al31B—Ru5B^xv^	150.0 (4)
Al27—Al13—Al31B	72.19 (16)	Al32B—Al31B—Ru5B^xv^	58.5 (2)
Al27^vii^—Al13—Al31B	93.16 (12)	Ru4A—Al31B—Ru5B^xv^	147.5 (3)
Al31A—Al13—Al31B	15.97 (15)	Ru5A^xv^—Al31B—Ru5B^xv^	2.15 (10)
Ru4B—Al13—Al31B^vii^	47.2 (5)	Al31A—Al31B—Al10B^xv^	50.6 (4)
Ru4A—Al13—Al31B^vii^	53.11 (14)	Al31C—Al31B—Al10B^xv^	126.5 (10)
Si8—Al13—Al31B^vii^	146.96 (12)	Al31B^vii^—Al31B—Al10B^xv^	76.16 (19)
Si19^vii^—Al13—Al31B^vii^	62.99 (11)	Al32A—Al31B—Al10B^xv^	127.8 (3)
Si19—Al13—Al31B^vii^	75.53 (16)	Al31C^vii^—Al31B—Al10B^xv^	85.1 (4)
Ru6^vii^—Al13—Al31B^vii^	101.66 (13)	Ru4B—Al31B—Al10B^xv^	147.8 (4)
Ru6—Al13—Al31B^vii^	125.08 (16)	Al32B—Al31B—Al10B^xv^	107.4 (3)
Al27—Al13—Al31B^vii^	93.16 (12)	Ru4A—Al31B—Al10B^xv^	148.8 (4)
Al27^vii^—Al13—Al31B^vii^	72.19 (16)	Ru5A^xv^—Al31B—Al10B^xv^	54.54 (15)
Al31A—Al13—Al31B^vii^	15.97 (15)	Ru5B^xv^—Al31B—Al10B^xv^	56.55 (17)
Al31B—Al13—Al31B^vii^	25.3 (3)	Al31A—Al31B—Si19	66.3 (4)
Al30B^ii^—Al14—Al30B^i^	59.9 (3)	Al31C—Al31B—Si19	98.8 (9)
Al30B^ii^—Al14—Ru2^iii^	141.14 (16)	Al31B^vii^—Al31B—Si19	101.39 (16)
Al30B^i^—Al14—Ru2^iii^	141.14 (16)	Al32A—Al31B—Si19	100.7 (2)
Al30B^ii^—Al14—Al11	75.89 (12)	Al31C^vii^—Al31B—Si19	109.1 (4)
Al30B^i^—Al14—Al11	75.89 (12)	Ru4B—Al31B—Si19	109.4 (3)
Ru2^iii^—Al14—Al11	132.37 (3)	Al32B—Al31B—Si19	99.9 (2)
Al30B^ii^—Al14—Al28^xi^	162.41 (16)	Ru4A—Al31B—Si19	107.26 (19)
Al30B^i^—Al14—Al28^xi^	107.05 (13)	Ru5A^xv^—Al31B—Si19	55.09 (12)
Ru2^iii^—Al14—Al28^xi^	56.454 (17)	Ru5B^xv^—Al31B—Si19	56.06 (14)
Al11—Al14—Al28^xi^	89.86 (2)	Al10B^xv^—Al31B—Si19	67.3 (3)
Al30B^ii^—Al14—Al28^xiv^	107.05 (13)	Al31B—Al31C—Al32A	176.2 (17)
Al30B^i^—Al14—Al28^xiv^	162.41 (16)	Al31B—Al31C—Al32B	148.0 (14)
Ru2^iii^—Al14—Al28^xiv^	56.454 (17)	Al32A—Al31C—Al32B	33.2 (5)
Al11—Al14—Al28^xiv^	89.86 (2)	Al31B—Al31C—Al31A	7.4 (6)
Al28^xi^—Al14—Al28^xiv^	82.78 (3)	Al32A—Al31C—Al31A	175.7 (11)
Al30B^ii^—Al14—Al30A^ii^	10.48 (9)	Al32B—Al31C—Al31A	148.5 (9)
Al30B^i^—Al14—Al30A^ii^	69.2 (2)	Al31B—Al31C—Al31B^vii^	15.6 (6)
Ru2^iii^—Al14—Al30A^ii^	130.97 (8)	Al32A—Al31C—Al31B^vii^	160.7 (11)
Al11—Al14—Al30A^ii^	82.35 (6)	Al32B—Al31C—Al31B^vii^	153.5 (8)
Al28^xi^—Al14—Al30A^ii^	171.95 (7)	Al31A—Al31C—Al31B^vii^	21.6 (2)
Al28^xiv^—Al14—Al30A^ii^	99.12 (7)	Al31B—Al31C—Al32B^xix^	138.0 (12)
Al30B^ii^—Al14—Al30A^i^	69.2 (2)	Al32A—Al31C—Al32B^xix^	43.3 (6)
Al30B^i^—Al14—Al30A^i^	10.48 (9)	Al32B—Al31C—Al32B^xix^	10.2 (3)
Ru2^iii^—Al14—Al30A^i^	130.97 (8)	Al31A—Al31C—Al32B^xix^	138.3 (7)
Al11—Al14—Al30A^i^	82.35 (6)	Al31B^vii^—Al31C—Al32B^xix^	145.3 (7)
Al28^xi^—Al14—Al30A^i^	99.12 (7)	Al31B—Al31C—Ru4B	73.8 (10)
Al28^xiv^—Al14—Al30A^i^	171.95 (7)	Al32A—Al31C—Ru4B	103.9 (9)
Al30A^ii^—Al14—Al30A^i^	77.95 (15)	Al32B—Al31C—Ru4B	134.7 (9)
Al30B^ii^—Al14—Ru1	55.27 (7)	Al31A—Al31C—Ru4B	75.7 (6)
Al30B^i^—Al14—Ru1	55.27 (7)	Al31B^vii^—Al31C—Ru4B	61.0 (5)
Ru2^iii^—Al14—Ru1	105.08 (2)	Al32B^xix^—Al31C—Ru4B	144.0 (7)
Al11—Al14—Ru1	122.55 (3)	Al31B—Al31C—Ru5A^xv^	86.2 (10)
Al28^xi^—Al14—Ru1	129.33 (2)	Al32A—Al31C—Ru5A^xv^	97.1 (9)
Al28^xiv^—Al14—Ru1	129.33 (2)	Al32B—Al31C—Ru5A^xv^	71.3 (5)
Al30A^ii^—Al14—Ru1	54.97 (2)	Al31A—Al31C—Ru5A^xv^	81.9 (5)
Al30A^i^—Al14—Ru1	54.97 (2)	Al31B^vii^—Al31C—Ru5A^xv^	101.4 (6)
Al30B^ii^—Al14—Ru7^vii^	57.95 (8)	Al32B^xix^—Al31C—Ru5A^xv^	63.2 (3)
Al30B^i^—Al14—Ru7^vii^	104.88 (16)	Ru4B—Al31C—Ru5A^xv^	151.1 (7)
Ru2^iii^—Al14—Ru7^vii^	113.742 (17)	Al31B—Al31C—Ru5B^xv^	89.1 (10)
Al11—Al14—Ru7^vii^	52.088 (11)	Al32A—Al31C—Ru5B^xv^	94.1 (9)
Al28^xi^—Al14—Ru7^vii^	120.55 (3)	Al32B—Al31C—Ru5B^xv^	68.6 (5)
Al28^xiv^—Al14—Ru7^vii^	57.677 (13)	Al31A—Al31C—Ru5B^xv^	84.9 (5)
Al30A^ii^—Al14—Ru7^vii^	55.52 (2)	Al31B^vii^—Al31C—Ru5B^xv^	104.4 (6)
Al30A^i^—Al14—Ru7^vii^	115.22 (9)	Al32B^xix^—Al31C—Ru5B^xv^	60.6 (3)
Ru1—Al14—Ru7^vii^	110.100 (17)	Ru4B—Al31C—Ru5B^xv^	153.2 (6)
Al30B^ii^—Al14—Ru7	104.88 (16)	Ru5A^xv^—Al31C—Ru5B^xv^	2.97 (11)
Al30B^i^—Al14—Ru7	57.95 (8)	Al31B—Al31C—Ru4A	76.9 (10)
Ru2^iii^—Al14—Ru7	113.743 (17)	Al32A—Al31C—Ru4A	100.9 (9)
Al11—Al14—Ru7	52.087 (11)	Al32B—Al31C—Ru4A	132.4 (8)
Al28^xi^—Al14—Ru7	57.677 (13)	Al31A—Al31C—Ru4A	78.5 (5)
Al28^xiv^—Al14—Ru7	120.55 (3)	Al31B^vii^—Al31C—Ru4A	64.4 (4)
Al30A^ii^—Al14—Ru7	115.22 (9)	Al32B^xix^—Al31C—Ru4A	142.0 (7)
Al30A^i^—Al14—Ru7	55.52 (2)	Ru4B—Al31C—Ru4A	3.9 (3)
Ru1—Al14—Ru7	110.100 (17)	Ru5A^xv^—Al31C—Ru4A	151.3 (6)
Ru7^vii^—Al14—Ru7	104.15 (2)	Ru5B^xv^—Al31C—Ru4A	153.1 (6)
Ru3—Al15—Ru4A	161.17 (5)	Al31B—Al31C—Al29B^viii^	114.2 (11)
Ru3—Al15—Si18^viii^	133.53 (3)	Al32A—Al31C—Al29B^viii^	62.0 (7)
Ru4A—Al15—Si18^viii^	60.00 (3)	Al32B—Al31C—Al29B^viii^	79.7 (6)
Ru3—Al15—Si18^ix^	133.53 (3)	Al31A—Al31C—Al29B^viii^	120.6 (7)
Ru4A—Al15—Si18^ix^	60.00 (3)	Al31B^vii^—Al31C—Al29B^viii^	99.0 (6)
Si18^viii^—Al15—Si18^ix^	66.95 (3)	Al32B^xix^—Al31C—Al29B^viii^	86.6 (5)
Ru3—Al15—Ru4B	159.3 (2)	Ru4B—Al31C—Al29B^viii^	60.3 (4)
Ru4A—Al15—Ru4B	1.90 (19)	Ru5A^xv^—Al31C—Al29B^viii^	148.5 (6)
Si18^viii^—Al15—Ru4B	61.48 (16)	Ru5B^xv^—Al31C—Al29B^viii^	146.4 (6)
Si18^ix^—Al15—Ru4B	61.48 (16)	Ru4A—Al31C—Al29B^viii^	60.1 (3)
Ru3—Al15—Al27^vii^	114.21 (2)	Al31B—Al31C—Al31C^vii^	25.5 (10)
Ru4A—Al15—Al27^vii^	57.01 (2)	Al32A—Al31C—Al31C^vii^	150.9 (7)
Si18^viii^—Al15—Al27^vii^	111.82 (3)	Al32B—Al31C—Al31C^vii^	152.9 (5)
Si18^ix^—Al15—Al27^vii^	61.257 (17)	Al31A—Al31C—Al31C^vii^	31.3 (4)
Ru4B—Al15—Al27^vii^	56.29 (8)	Al31B^vii^—Al31C—Al31C^vii^	9.9 (4)
Ru3—Al15—Al27	114.21 (2)	Al32B^xix^—Al31C—Al31C^vii^	147.3 (3)
Ru4A—Al15—Al27	57.01 (2)	Ru4B—Al31C—Al31C^vii^	53.3 (5)
Si18^viii^—Al15—Al27	61.258 (17)	Ru5A^xv^—Al31C—Al31C^vii^	111.0 (4)
Si18^ix^—Al15—Al27	111.82 (3)	Ru5B^xv^—Al31C—Al31C^vii^	114.0 (4)
Ru4B—Al15—Al27	56.29 (8)	Ru4A—Al31C—Al31C^vii^	56.9 (4)
Al27^vii^—Al15—Al27	101.23 (3)	Al29B^viii^—Al31C—Al31C^vii^	89.3 (4)
Ru3—Al15—Al21	57.760 (15)	Al32B—Al32A—Al31C	112.8 (8)
Ru4A—Al15—Al21	122.019 (17)	Al32B—Al32A—Al32B^xix^	2.4 (5)
Si18^viii^—Al15—Al21	82.302 (16)	Al31C—Al32A—Al32B^xix^	114.9 (8)
Si18^ix^—Al15—Al21	142.66 (3)	Al32B—Al32A—Al31B	113.5 (4)
Ru4B—Al15—Al21	121.78 (3)	Al31C—Al32A—Al31B	1.8 (8)
Al27^vii^—Al15—Al21	155.18 (4)	Al32B^xix^—Al32A—Al31B	115.5 (2)
Al27—Al15—Al21	66.782 (15)	Al32B—Al32A—Al29B^viii^	111.2 (3)
Ru3—Al15—Al21^vii^	57.759 (15)	Al31C—Al32A—Al29B^viii^	96.8 (7)
Ru4A—Al15—Al21^vii^	122.019 (17)	Al32B^xix^—Al32A—Al29B^viii^	111.7 (2)
Si18^viii^—Al15—Al21^vii^	142.66 (3)	Al31B—Al32A—Al29B^viii^	95.1 (2)
Si18^ix^—Al15—Al21^vii^	82.302 (16)	Al32B—Al32A—Al31A	114.0 (3)
Ru4B—Al15—Al21^vii^	121.78 (3)	Al31C—Al32A—Al31A	2.7 (7)
Al27^vii^—Al15—Al21^vii^	66.782 (15)	Al32B^xix^—Al32A—Al31A	115.96 (16)
Al27—Al15—Al21^vii^	155.18 (4)	Al31B—Al32A—Al31A	4.16 (18)
Al21—Al15—Al21^vii^	114.74 (3)	Al29B^viii^—Al32A—Al31A	98.51 (15)
Ru3—Al15—Al13	107.37 (3)	Al32B—Al32A—Al29A^viii^	112.6 (3)
Ru4A—Al15—Al13	53.79 (3)	Al31C—Al32A—Al29A^viii^	98.7 (7)
Si18^viii^—Al15—Al13	104.09 (3)	Al32B^xix^—Al32A—Al29A^viii^	112.93 (15)
Si18^ix^—Al15—Al13	104.09 (3)	Al31B—Al32A—Al29A^viii^	96.98 (19)
Ru4B—Al15—Al13	51.9 (2)	Al29B^viii^—Al32A—Al29A^viii^	3.24 (15)
Al27^vii^—Al15—Al13	54.228 (18)	Al31A—Al32A—Al29A^viii^	100.31 (5)
Al27—Al15—Al13	54.228 (18)	Al32B—Al32A—Al32A^xix^	1.5 (3)
Al21—Al15—Al13	103.53 (2)	Al31C—Al32A—Al32A^xix^	114.2 (7)
Al21^vii^—Al15—Al13	103.53 (2)	Al32B^xix^—Al32A—Al32A^xix^	0.82 (16)
Si17^xv^—Si16—Ru6^vii^	111.935 (18)	Al31B—Al32A—Al32A^xix^	114.81 (19)
Si17^xv^—Si16—Ru2^ix^	112.514 (19)	Al29B^viii^—Al32A—Al32A^xix^	111.50 (14)
Ru6^vii^—Si16—Ru2^ix^	124.595 (17)	Al31A—Al32A—Al32A^xix^	115.28 (5)
Si17^xv^—Si16—Al20	138.74 (2)	Al29A^viii^—Al32A—Al32A^xix^	112.81 (5)
Ru6^vii^—Si16—Al20	62.558 (15)	Al32B—Al32A—Ru5B^xx^	62.4 (3)
Ru2^ix^—Si16—Al20	62.335 (15)	Al31C—Al32A—Ru5B^xx^	143.9 (7)
Si17^xv^—Si16—Al12^ix^	62.396 (19)	Al32B^xix^—Al32A—Ru5B^xx^	61.60 (19)
Ru6^vii^—Si16—Al12^ix^	119.15 (2)	Al31B—Al32A—Ru5B^xx^	142.5 (2)
Ru2^ix^—Si16—Al12^ix^	59.702 (15)	Al29B^viii^—Al32A—Ru5B^xx^	58.16 (18)
Al20—Si16—Al12^ix^	84.14 (2)	Al31A—Al32A—Ru5B^xx^	146.61 (11)
Si17^xv^—Si16—Al26^vii^	111.74 (2)	Al29A^viii^—Al32A—Ru5B^xx^	57.75 (13)
Ru6^vii^—Si16—Al26^vii^	64.944 (14)	Al32A^xix^—Al32A—Ru5B^xx^	61.87 (11)
Ru2^ix^—Si16—Al26^vii^	123.088 (19)	Al32B—Al32A—Al27	140.0 (3)
Al20—Si16—Al26^vii^	102.37 (2)	Al31C—Al32A—Al27	94.7 (7)
Al12^ix^—Si16—Al26^vii^	173.49 (2)	Al32B^xix^—Al32A—Al27	137.76 (16)
Si17^xv^—Si16—Al21^xvi^	62.572 (17)	Al31B—Al32A—Al27	95.01 (18)
Ru6^vii^—Si16—Al21^xvi^	114.263 (19)	Al29B^viii^—Al32A—Al27	92.69 (15)
Ru2^ix^—Si16—Al21^xvi^	114.665 (18)	Al31A—Al32A—Al27	92.49 (4)
Al20—Si16—Al21^xvi^	158.69 (2)	Al29A^viii^—Al32A—Al27	89.91 (6)
Al12^ix^—Si16—Al21^xvi^	113.27 (2)	Al32A^xix^—Al32A—Al27	138.53 (5)
Al26^vii^—Si16—Al21^xvi^	60.348 (17)	Ru5B^xx^—Al32A—Al27	110.71 (8)
Si17^xv^—Si16—Al28	110.33 (2)	Al32B—Al32A—Ru4B	160.5 (5)
Ru6^vii^—Si16—Al28	128.66 (2)	Al31C—Al32A—Ru4B	56.6 (8)
Ru2^ix^—Si16—Al28	58.969 (15)	Al32B^xix^—Al32A—Ru4B	162.6 (4)
Al20—Si16—Al28	101.00 (2)	Al31B—Al32A—Ru4B	55.5 (4)
Al12^ix^—Si16—Al28	105.54 (2)	Al29B^viii^—Al32A—Ru4B	58.5 (2)
Al26^vii^—Si16—Al28	73.278 (18)	Al31A—Al32A—Ru4B	56.4 (3)
Al21^xvi^—Si16—Al28	63.524 (17)	Al29A^viii^—Al32A—Ru4B	58.12 (17)
Si17^xv^—Si16—Al25	62.967 (17)	Al32A^xix^—Al32A—Ru4B	161.9 (3)
Ru6^vii^—Si16—Al25	59.025 (13)	Ru5B^xx^—Al32A—Ru4B	114.9 (2)
Ru2^ix^—Si16—Al25	118.817 (19)	Al27—Al32A—Ru4B	59.5 (3)
Al20—Si16—Al25	83.338 (19)	Al32B—Al32A—Ru5A^xv^	62.3 (3)
Al12^ix^—Si16—Al25	68.317 (19)	Al31C—Al32A—Ru5A^xv^	63.0 (7)
Al26^vii^—Si16—Al25	112.27 (2)	Al32B^xix^—Al32A—Ru5A^xv^	63.20 (16)
Al21^xvi^—Si16—Al25	113.86 (2)	Al31B—Al32A—Ru5A^xv^	64.5 (2)
Al28—Si16—Al25	172.26 (2)	Al29B^viii^—Al32A—Ru5A^xv^	148.96 (17)
Ru1^ix^—Si17—Ru5B^vii^	122.40 (13)	Al31A—Al32A—Ru5A^xv^	62.40 (7)
Ru1^ix^—Si17—Si16^vi^	111.692 (19)	Al29A^viii^—Al32A—Ru5A^xv^	152.17 (8)
Ru5B^vii^—Si17—Si16^vi^	113.65 (11)	Al32A^xix^—Al32A—Ru5A^xv^	62.89 (6)
Ru1^ix^—Si17—Ru5A^vii^	125.02 (6)	Ru5B^xx^—Al32A—Ru5A^xv^	124.72 (10)
Ru5B^vii^—Si17—Ru5A^vii^	2.76 (11)	Al27—Al32A—Ru5A^xv^	111.33 (4)
Si16^vi^—Si17—Ru5A^vii^	112.18 (4)	Ru4B—Al32A—Ru5A^xv^	116.7 (3)
Ru1^ix^—Si17—Al29A	62.27 (8)	Al32B^xix^—Al32B—Al32A	175.0 (10)
Ru5B^vii^—Si17—Al29A	60.21 (15)	Al32B^xix^—Al32B—Al31C	150.2 (10)
Si16^vi^—Si17—Al29A	140.22 (10)	Al32A—Al32B—Al31C	34.0 (5)
Ru5A^vii^—Si17—Al29A	62.89 (9)	Al32B^xix^—Al32B—Al32A^xix^	2.7 (5)
Ru1^ix^—Si17—Al23^vii^	125.87 (2)	Al32A—Al32B—Al32A^xix^	177.6 (5)
Ru5B^vii^—Si17—Al23^vii^	66.12 (10)	Al31C—Al32B—Al32A^xix^	148.0 (6)
Si16^vi^—Si17—Al23^vii^	109.97 (2)	Al32B^xix^—Al32B—Al31C^xix^	19.6 (7)
Ru5A^vii^—Si17—Al23^vii^	64.14 (5)	Al32A—Al32B—Al31C^xix^	156.1 (5)
Al29A—Si17—Al23^vii^	102.62 (5)	Al31C—Al32B—Al31C^xix^	169.8 (3)
Ru1^ix^—Si17—Al12^xii^	59.105 (16)	Al32A^xix^—Al32B—Al31C^xix^	21.8 (4)
Ru5B^vii^—Si17—Al12^xii^	118.91 (10)	Al32B^xix^—Al32B—Al31B	139.3 (8)
Si16^vi^—Si17—Al12^xii^	61.419 (18)	Al32A—Al32B—Al31B	45.0 (3)
Ru5A^vii^—Si17—Al12^xii^	120.58 (5)	Al31C—Al32B—Al31B	11.1 (5)
Al29A—Si17—Al12^xii^	86.41 (5)	Al32A^xix^—Al32B—Al31B	137.0 (3)
Al23^vii^—Si17—Al12^xii^	170.93 (2)	Al31C^xix^—Al32B—Al31B	158.9 (4)
Ru1^ix^—Si17—Al21^vii^	115.734 (19)	Al32B^xix^—Al32B—Ru5B^xv^	80.7 (6)
Ru5B^vii^—Si17—Al21^vii^	116.11 (12)	Al32A—Al32B—Ru5B^xv^	101.0 (3)
Si16^vi^—Si17—Al21^vii^	61.723 (16)	Al31C—Al32B—Ru5B^xv^	75.2 (5)
Ru5A^vii^—Si17—Al21^vii^	113.40 (6)	Al32A^xix^—Al32B—Ru5B^xv^	79.8 (2)
Al29A—Si17—Al21^vii^	158.05 (10)	Al31C^xix^—Al32B—Ru5B^xv^	96.6 (4)
Al23^vii^—Si17—Al21^vii^	59.590 (16)	Al31B—Al32B—Ru5B^xv^	68.1 (2)
Al12^xii^—Si17—Al21^vii^	111.82 (2)	Al32B^xix^—Al32B—Ru5B^xx^	80.2 (6)
Ru1^ix^—Si17—Al30A	60.19 (7)	Al32A—Al32B—Ru5B^xx^	98.1 (4)
Ru5B^vii^—Si17—Al30A	130.58 (10)	Al31C—Al32B—Ru5B^xx^	122.9 (5)
Si16^vi^—Si17—Al30A	107.71 (3)	Al32A^xix^—Al32B—Ru5B^xx^	81.0 (2)
Ru5A^vii^—Si17—Al30A	130.32 (5)	Al31C^xix^—Al32B—Ru5B^xx^	64.7 (4)
Al29A—Si17—Al30A	102.02 (13)	Al31B—Al32B—Ru5B^xx^	128.6 (3)
Al23^vii^—Si17—Al30A	75.34 (7)	Ru5B^xv^—Al32B—Ru5B^xx^	160.8 (2)
Al12^xii^—Si17—Al30A	103.87 (6)	Al32B^xix^—Al32B—Ru5A^xv^	83.6 (6)
Al21^vii^—Si17—Al30A	62.80 (5)	Al32A—Al32B—Ru5A^xv^	98.3 (3)
Ru1^ix^—Si17—Al29B	67.3 (2)	Al31C—Al32B—Ru5A^xv^	71.9 (5)
Ru5B^vii^—Si17—Al29B	55.8 (2)	Al32A^xix^—Al32B—Ru5A^xv^	82.59 (19)
Si16^vi^—Si17—Al29B	134.5 (2)	Al31C^xix^—Al32B—Ru5A^xv^	99.8 (4)
Ru5A^vii^—Si17—Al29B	58.5 (2)	Al31B—Al32B—Ru5A^xv^	64.6 (2)
Al29A—Si17—Al29B	7.30 (18)	Ru5B^xv^—Al32B—Ru5A^xv^	3.53 (12)
Al23^vii^—Si17—Al29B	104.21 (11)	Ru5B^xx^—Al32B—Ru5A^xv^	163.6 (3)
Al12^xii^—Si17—Al29B	84.64 (11)	Al32B^xix^—Al32B—Ru5A^xx^	77.5 (6)
Al21^vii^—Si17—Al29B	162.70 (15)	Al32A—Al32B—Ru5A^xx^	100.6 (3)
Al30A—Si17—Al29B	109.3 (3)	Al31C—Al32B—Ru5A^xx^	126.1 (5)
Ru1^ix^—Si17—Al25^vi^	117.214 (19)	Al32A^xix^—Al32B—Ru5A^xx^	78.50 (18)
Ru5B^vii^—Si17—Al25^vi^	59.29 (9)	Al31C^xix^—Al32B—Ru5A^xx^	61.7 (3)
Si16^vi^—Si17—Al25^vi^	63.594 (17)	Al31B—Al32B—Ru5A^xx^	131.9 (3)
Ru5A^vii^—Si17—Al25^vi^	59.47 (3)	Ru5B^xv^—Al32B—Ru5A^xx^	158.1 (2)
Al29A—Si17—Al25^vi^	83.82 (13)	Ru5B^xx^—Al32B—Ru5A^xx^	3.31 (12)
Al23^vii^—Si17—Al25^vi^	111.48 (2)	Ru5A^xv^—Al32B—Ru5A^xx^	161.1 (2)
Al12^xii^—Si17—Al25^vi^	68.218 (19)	Al32B^xix^—Al32B—Al29B^viii^	130.1 (7)
Al21^vii^—Si17—Al25^vi^	113.69 (2)	Al32A—Al32B—Al29B^viii^	50.8 (3)
Al30A—Si17—Al25^vi^	170.09 (4)	Al31C—Al32B—Al29B^viii^	66.8 (5)
Al29B—Si17—Al25^vi^	76.6 (3)	Al32A^xix^—Al32B—Al29B^viii^	129.7 (3)
Al24^vii^—Si18—Ru4A^ii^	116.89 (5)	Al31C^xix^—Al32B—Al29B^viii^	119.7 (4)
Al24^vii^—Si18—Ru2^ix^	115.631 (19)	Al31B—Al32B—Al29B^viii^	72.6 (2)
Ru4A^ii^—Si18—Ru2^ix^	111.39 (3)	Ru5B^xv^—Al32B—Al29B^viii^	140.5 (3)
Al24^vii^—Si18—Ru7^vii^	65.391 (15)	Ru5B^xx^—Al32B—Al29B^viii^	56.3 (2)
Ru4A^ii^—Si18—Ru7^vii^	117.981 (18)	Ru5A^xv^—Al32B—Al29B^viii^	136.9 (3)
Ru2^ix^—Si18—Ru7^vii^	122.264 (17)	Ru5A^xx^—Al32B—Al29B^viii^	59.51 (19)
Al24^vii^—Si18—Al20	62.973 (18)	Al32B^xix^—Al32B—Al22^xx^	99.0 (7)
Ru4A^ii^—Si18—Al20	111.35 (3)	Al32A—Al32B—Al22^xx^	76.1 (3)
Ru2^ix^—Si18—Al20	61.341 (15)	Al31C—Al32B—Al22^xx^	108.2 (5)
Ru7^vii^—Si18—Al20	119.95 (2)	Al32A^xix^—Al32B—Al22^xx^	101.6 (2)
Al24^vii^—Si18—Al29A	64.36 (3)	Al31C^xix^—Al32B—Al22^xx^	81.1 (4)
Ru4A^ii^—Si18—Al29A	60.69 (9)	Al31B—Al32B—Al22^xx^	118.9 (2)
Ru2^ix^—Si18—Al29A	111.40 (10)	Ru5B^xv^—Al32B—Al22^xx^	120.6 (2)
Ru7^vii^—Si18—Al29A	117.65 (6)	Ru5B^xx^—Al32B—Al22^xx^	62.83 (14)
Al20—Si18—Al29A	61.85 (11)	Ru5A^xv^—Al32B—Al22^xx^	122.35 (18)
Al24^vii^—Si18—Al15^ii^	153.95 (2)	Ru5A^xx^—Al32B—Al22^xx^	61.65 (11)
Ru4A^ii^—Si18—Al15^ii^	57.69 (5)	Al29B^viii^—Al32B—Al22^xx^	82.81 (18)

Symmetry codes: (i) −*x*+1/2, *y*−1/2, *z*+1/2; (ii) −*x*+1/2, −*y*+1, *z*+1/2; (iii) *x*−1/2, *y*, −*z*+3/2; (iv) *x*+1/2, *y*, −*z*+3/2; (v) *x*+1/2, −*y*+1/2, −*z*+1/2; (vi) *x*+1/2, *y*, −*z*+1/2; (vii) *x*, −*y*+1/2, *z*; (viii) −*x*+1/2, *y*−1/2, *z*−1/2; (ix) −*x*+1/2, −*y*+1, *z*−1/2; (x) −*x*+1/2, −*y*, *z*+1/2; (xi) −*x*, *y*−1/2, −*z*+1; (xii) −*x*+1, −*y*+1, −*z*+1; (xiii) −*x*+1, *y*−1/2, −*z*+1; (xiv) −*x*, −*y*+1, −*z*+1; (xv) *x*−1/2, *y*, −*z*+1/2; (xvi) *x*−1/2, −*y*+1/2, −*z*+1/2; (xvii) −*x*+1/2, *y*+1/2, *z*+1/2; (xviii) *x*, −*y*+3/2, *z*; (xix) −*x*, −*y*, −*z*; (xx) −*x*+1/2, −*y*, *z*−1/2; (xxi) −*x*+1/2, *y*+1/2, *z*−1/2; (xxii) −*x*, *y*+1/2, −*z*+1.

### Trialuminium ruthenium disilicon (IV) Crystal data

**Table:**

~Ru(Al_0.57_Si_0.43_)_5_	*D*_x_ = 4.248 Mg m^−^^3^
*M**_r_* = 238.38	Mo *K*α radiation, λ = 0.71073 Å
Tetragonal, *I*4/*m**c**m*	Cell parameters from 5393 reflections
*a* = 6.20079 (8) Å	θ = 4.2–38.2°
*c* = 9.6937 (2) Å	µ = 5.32 mm^−^^1^
*V* = 372.72 (1) Å^3^	*T* = 297 K
*Z* = 4	Irregular, metallic light silver
*F*(000) = 440.99	0.05 × 0.03 × 0.02 mm

### Trialuminium ruthenium disilicon (IV) Data collection

**Table:**

XtaLAB Synergy R, HyPix diffractometer	295 independent reflections
Radiation source: micro-focus sealed X-ray tube, Mova (Mo) X-ray Source	279 reflections with *I* > 2σ(*I*)
Mirror monochromator	*R*_int_ = 0.029
Detector resolution: 10.0000 pixels mm^-1^	θ_max_ = 37.9°, θ_min_ = 4.2°
ω scans	*h* = −10→10
Absorption correction: gaussian (CrysAlisPro; Matsumoto *et al.*, 2021)	*k* = −10→10
*T*_min_ = 0.833, *T*_max_ = 0.906	*l* = −16→16
8063 measured reflections	

### Trialuminium ruthenium disilicon (IV) Refinement

**Table:**

Refinement on *F*^2^	Primary atom site location: dual
Least-squares matrix: full	*w* = 1/[σ^2^(*F*_o_^2^) + (0.0084*P*)^2^ + 0.2167*P*] where *P* = (*F*_o_^2^ + 2*F*_c_^2^)/3
*R*[*F*^2^ > 2σ(*F*^2^)] = 0.009	(Δ/σ)_max_ < 0.001
*wR*(*F*^2^) = 0.021	Δρ_max_ = 0.44 e Å^−^^3^
*S* = 1.21	Δρ_min_ = −0.42 e Å^−^^3^
295 reflections	Extinction correction: SHELXL-2019/3 (Sheldrick, 2015b), Fc^*^=kFc[1+0.001xFc^2^λ^3^/sin(2θ)]^-1/4^
13 parameters	Extinction coefficient: 0.0037 (3)
2 restraints	

### Trialuminium ruthenium disilicon (IV) Special details

**Table:**

Geometry. All esds (except the esd in the dihedral angle between two l.s. planes) are estimated using the full covariance matrix. The cell esds are taken into account individually in the estimation of esds in distances, angles and torsion angles; correlations between esds in cell parameters are only used when they are defined by crystal symmetry. An approximate (isotropic) treatment of cell esds is used for estimating esds involving l.s. planes.

### Trialuminium ruthenium disilicon (IV) Fractional atomic coordinates and isotropic or equivalent isotropic displacement parameters (Å^2^)

**Table:**

	*x*	*y*	*z*	*U*_iso_*/*U*_eq_	Occ. (<1)
Ru1	0.000000	0.000000	0.250000	0.00545 (5)	
Al2	0.000000	0.000000	0.000000	0.01321 (14)	
Si3	0.14854 (4)	0.64854 (4)	0.14385 (4)	0.01122 (8)	0.542 (6)
Al3	0.14854 (4)	0.64854 (4)	0.14385 (4)	0.01122 (8)	0.458 (6)

### Trialuminium ruthenium disilicon (IV) Atomic displacement parameters (Å^2^)

**Table:**

	*U*^11^	*U*^22^	*U*^33^	*U*^12^	*U*^13^	*U*^23^
Ru1	0.00611 (6)	0.00611 (6)	0.00414 (7)	0.000	0.000	0.000
Al2	0.0174 (2)	0.0174 (2)	0.0048 (3)	0.000	0.000	0.000
Si3	0.01030 (10)	0.01030 (10)	0.01304 (15)	0.00364 (11)	0.00111 (8)	0.00111 (8)
Al3	0.01030 (10)	0.01030 (10)	0.01304 (15)	0.00364 (11)	0.00111 (8)	0.00111 (8)

### Trialuminium ruthenium disilicon (IV) Geometric parameters (Å, º)

**Table:**

Ru1—Al2^i^	2.4234 (1)	Ru1—Si3^ix^	2.5800 (2)
Ru1—Al2	2.4234 (1)	Al2—Si3^x^	2.7463 (2)
Ru1—Si3^ii^	2.5800 (2)	Al2—Si3^vii^	2.7463 (2)
Ru1—Si3^iii^	2.5800 (2)	Al2—Si3^xi^	2.7463 (2)
Ru1—Si3^iv^	2.5800 (2)	Al2—Si3^ix^	2.7463 (2)
Ru1—Si3^v^	2.5800 (2)	Al2—Si3^xii^	2.7463 (2)
Ru1—Si3^vi^	2.5800 (2)	Al2—Si3^ii^	2.7463 (2)
Ru1—Si3^vii^	2.5800 (2)	Al2—Si3^xiii^	2.7463 (2)
Ru1—Si3^viii^	2.5800 (2)	Al2—Si3^iv^	2.7463 (2)
			
Al2^i^—Ru1—Al2	180.0	Si3^vii^—Al2—Si3^xii^	61.029 (13)
Al2^i^—Ru1—Si3^ii^	113.504 (7)	Si3^xi^—Al2—Si3^xii^	75.060 (6)
Al2—Ru1—Si3^ii^	66.496 (7)	Si3^ix^—Al2—Si3^xii^	104.940 (6)
Al2^i^—Ru1—Si3^iii^	66.496 (7)	Ru1^xiv^—Al2—Si3^ii^	120.514 (7)
Al2—Ru1—Si3^iii^	113.504 (7)	Ru1—Al2—Si3^ii^	59.486 (7)
Si3^ii^—Ru1—Si3^iii^	139.656 (16)	Si3^x^—Al2—Si3^ii^	61.029 (13)
Al2^i^—Ru1—Si3^iv^	113.504 (7)	Si3^vii^—Al2—Si3^ii^	118.971 (13)
Al2—Ru1—Si3^iv^	66.496 (7)	Si3^xi^—Al2—Si3^ii^	104.940 (6)
Si3^ii^—Ru1—Si3^iv^	80.848 (5)	Si3^ix^—Al2—Si3^ii^	75.060 (6)
Si3^iii^—Ru1—Si3^iv^	138.167 (15)	Si3^xii^—Al2—Si3^ii^	180.000 (17)
Al2^i^—Ru1—Si3^v^	66.496 (7)	Ru1^xiv^—Al2—Si3^xiii^	59.486 (7)
Al2—Ru1—Si3^v^	113.504 (7)	Ru1—Al2—Si3^xiii^	120.514 (7)
Si3^ii^—Ru1—Si3^v^	138.167 (15)	Si3^x^—Al2—Si3^xiii^	75.060 (6)
Si3^iii^—Ru1—Si3^v^	80.848 (5)	Si3^vii^—Al2—Si3^xiii^	104.940 (6)
Si3^iv^—Ru1—Si3^v^	63.637 (14)	Si3^xi^—Al2—Si3^xiii^	118.971 (13)
Al2^i^—Ru1—Si3^vi^	66.496 (7)	Si3^ix^—Al2—Si3^xiii^	61.029 (13)
Al2—Ru1—Si3^vi^	113.504 (7)	Si3^xii^—Al2—Si3^xiii^	75.060 (6)
Si3^ii^—Ru1—Si3^vi^	63.637 (14)	Si3^ii^—Al2—Si3^xiii^	104.940 (6)
Si3^iii^—Ru1—Si3^vi^	132.991 (15)	Ru1^xiv^—Al2—Si3^iv^	120.514 (7)
Si3^iv^—Ru1—Si3^vi^	64.724 (16)	Ru1—Al2—Si3^iv^	59.486 (7)
Si3^v^—Ru1—Si3^vi^	80.848 (5)	Si3^x^—Al2—Si3^iv^	104.940 (6)
Al2^i^—Ru1—Si3^vii^	113.504 (7)	Si3^vii^—Al2—Si3^iv^	75.060 (6)
Al2—Ru1—Si3^vii^	66.496 (7)	Si3^xi^—Al2—Si3^iv^	61.029 (13)
Si3^ii^—Ru1—Si3^vii^	132.991 (15)	Si3^ix^—Al2—Si3^iv^	118.971 (13)
Si3^iii^—Ru1—Si3^vii^	63.637 (14)	Si3^xii^—Al2—Si3^iv^	104.940 (6)
Si3^iv^—Ru1—Si3^vii^	80.848 (5)	Si3^ii^—Al2—Si3^iv^	75.060 (6)
Si3^v^—Ru1—Si3^vii^	64.724 (16)	Si3^xiii^—Al2—Si3^iv^	180.000 (13)
Si3^vi^—Ru1—Si3^vii^	139.656 (16)	Ru1^xv^—Al3—Ru1^v^	116.363 (14)
Al2^i^—Ru1—Si3^viii^	66.496 (7)	Ru1^xv^—Al3—Si3^ix^	110.173 (8)
Al2—Ru1—Si3^viii^	113.504 (7)	Ru1^v^—Al3—Si3^ix^	110.173 (8)
Si3^ii^—Ru1—Si3^viii^	64.724 (16)	Ru1^xv^—Al3—Si3^xvi^	58.182 (7)
Si3^iii^—Ru1—Si3^viii^	80.848 (5)	Ru1^v^—Al3—Si3^xvi^	58.182 (7)
Si3^iv^—Ru1—Si3^viii^	139.656 (16)	Si3^ix^—Al3—Si3^xvi^	130.850 (15)
Si3^v^—Ru1—Si3^viii^	132.991 (15)	Ru1^xv^—Al3—Al2^xv^	54.019 (3)
Si3^vi^—Ru1—Si3^viii^	80.848 (5)	Ru1^v^—Al3—Al2^xv^	140.272 (15)
Si3^vii^—Ru1—Si3^viii^	138.167 (15)	Si3^ix^—Al3—Al2^xv^	108.902 (7)
Al2^i^—Ru1—Si3^ix^	113.504 (7)	Si3^xvi^—Al3—Al2^xv^	99.916 (13)
Al2—Ru1—Si3^ix^	66.496 (7)	Ru1^xv^—Al3—Al2^xvii^	140.272 (15)
Si3^ii^—Ru1—Si3^ix^	80.848 (5)	Ru1^v^—Al3—Al2^xvii^	54.019 (3)
Si3^iii^—Ru1—Si3^ix^	64.724 (16)	Si3^ix^—Al3—Al2^xvii^	108.902 (7)
Si3^iv^—Ru1—Si3^ix^	132.991 (15)	Si3^xvi^—Al3—Al2^xvii^	99.916 (13)
Si3^v^—Ru1—Si3^ix^	139.656 (16)	Al2^xv^—Al3—Al2^xvii^	105.930 (12)
Si3^vi^—Ru1—Si3^ix^	138.167 (15)	Ru1^xv^—Al3—Si3^xviii^	57.638 (8)
Si3^vii^—Ru1—Si3^ix^	80.848 (5)	Ru1^v^—Al3—Si3^xviii^	105.441 (17)
Si3^viii^—Ru1—Si3^ix^	63.637 (14)	Si3^ix^—Al3—Si3^xviii^	61.861 (8)
Ru1^xiv^—Al2—Ru1	180.0	Si3^xvi^—Al3—Si3^xviii^	75.218 (17)
Ru1^xiv^—Al2—Si3^x^	59.486 (7)	Al2^xv^—Al3—Si3^xviii^	98.895 (8)
Ru1—Al2—Si3^x^	120.514 (7)	Al2^xvii^—Al3—Si3^xviii^	155.175 (11)
Ru1^xiv^—Al2—Si3^vii^	120.514 (7)	Ru1^xv^—Al3—Si3^iii^	105.441 (17)
Ru1—Al2—Si3^vii^	59.486 (7)	Ru1^v^—Al3—Si3^iii^	57.638 (8)
Si3^x^—Al2—Si3^vii^	180.000 (13)	Si3^ix^—Al3—Si3^iii^	61.861 (8)
Ru1^xiv^—Al2—Si3^xi^	59.486 (7)	Si3^xvi^—Al3—Si3^iii^	75.218 (17)
Ru1—Al2—Si3^xi^	120.514 (7)	Al2^xv^—Al3—Si3^iii^	155.175 (11)
Si3^x^—Al2—Si3^xi^	75.060 (6)	Al2^xvii^—Al3—Si3^iii^	98.895 (8)
Si3^vii^—Al2—Si3^xi^	104.940 (6)	Si3^xviii^—Al3—Si3^iii^	56.280 (15)
Ru1^xiv^—Al2—Si3^ix^	120.514 (7)	Ru1^xv^—Al3—Si3^xix^	113.504 (7)
Ru1—Al2—Si3^ix^	59.486 (7)	Ru1^v^—Al3—Si3^xix^	113.504 (7)
Si3^x^—Al2—Si3^ix^	104.940 (6)	Si3^ix^—Al3—Si3^xix^	90.0
Si3^vii^—Al2—Si3^ix^	75.060 (6)	Si3^xvi^—Al3—Si3^xix^	139.151 (15)
Si3^xi^—Al2—Si3^ix^	180.00 (2)	Al2^xv^—Al3—Si3^xix^	59.485 (7)
Ru1^xiv^—Al2—Si3^xii^	59.486 (7)	Al2^xvii^—Al3—Si3^xix^	59.485 (7)
Ru1—Al2—Si3^xii^	120.514 (7)	Si3^xviii^—Al3—Si3^xix^	138.167 (13)
Si3^x^—Al2—Si3^xii^	118.971 (13)	Si3^iii^—Al3—Si3^xix^	138.167 (13)

Symmetry codes: (i) −*x*, *y*, −*z*+1/2; (ii) *y*−1, −*x*, *z*; (iii) *y*−1/2, −*x*+1/2, −*z*+1/2; (iv) *x*, *y*−1, *z*; (v) −*x*+1/2, −*y*+1/2, −*z*+1/2; (vi) −*y*+1/2, *x*−1/2, −*z*+1/2; (vii) −*y*+1, *x*, *z*; (viii) *x*−1/2, *y*−1/2, −*z*+1/2; (ix) −*x*, −*y*+1, *z*; (x) *y*−1, −*x*, −*z*; (xi) *x*, *y*−1, −*z*; (xii) −*y*+1, *x*, −*z*; (xiii) −*x*, −*y*+1, −*z*; (xiv) −*x*, −*y*, −*z*; (xv) *x*, *y*+1, *z*; (xvi) −*x*+1/2, −*y*+3/2, −*z*+1/2; (xvii) −*x*+1/2, *y*+1/2, −*z*; (xviii) −*y*+1/2, *x*+1/2, −*z*+1/2; (xix) *x*, *y*, −*z*.

### Tetraaluminium diruthenium pentasilicon (V) Crystal data

**Table:**

~Ru_2_(Al_0.46_Si_0.54_)_9_	*D*_x_ = 4.512 Mg m^−^^3^
*M**_r_* = 450.41	Mo *K*α radiation, λ = 0.71073 Å
Orthorhombic, *C**m**c**m*	Cell parameters from 11544 reflections
*a* = 8.63058 (14) Å	θ = 2.3–30.7°
*b* = 8.79888 (15) Å	µ = 5.88 mm^−^^1^
*c* = 17.4620 (3) Å	*T* = 301 K
*V* = 1326.05 (4) Å^3^	Irregular, metallic light silver
*Z* = 8	0.05 × 0.04 × 0.04 mm
*F*(000) = 1664.17	

### Tetraaluminium diruthenium pentasilicon (V) Data collection

**Table:**

XtaLAB Synergy R, HyPix diffractometer	1029 independent reflections
Radiation source: micro-focus sealed X-ray tube, Mova (Mo) X-ray Source	1001 reflections with *I* > 2σ(*I*)
Mirror monochromator	*R*_int_ = 0.016
Detector resolution: 10.0000 pixels mm^-1^	θ_max_ = 30.8°, θ_min_ = 2.3°
ω scans	*h* = −12→11
Absorption correction: gaussian (CrysAlisPro; Matsumoto *et al.*, 2021)	*k* = −11→11
*T*_min_ = 0.826, *T*_max_ = 0.868	*l* = −22→24
17672 measured reflections	

### Tetraaluminium diruthenium pentasilicon (V) Refinement

**Table:**

Refinement on *F*^2^	Primary atom site location: dual
Least-squares matrix: full	*w* = 1/[σ^2^(*F*_o_^2^) + (0.0114*P*)^2^ + 0.7123*P*] where *P* = (*F*_o_^2^ + 2*F*_c_^2^)/3
*R*[*F*^2^ > 2σ(*F*^2^)] = 0.011	(Δ/σ)_max_ = 0.001
*wR*(*F*^2^) = 0.027	Δρ_max_ = 0.37 e Å^−^^3^
*S* = 1.31	Δρ_min_ = −0.42 e Å^−^^3^
1029 reflections	Extinction correction: SHELXL-2019/3 (Sheldrick, 2015b), Fc^*^=kFc[1+0.001xFc^2^λ^3^/sin(2θ)]^-1/4^
61 parameters	Extinction coefficient: 0.00053 (3)
2 restraints	

### Tetraaluminium diruthenium pentasilicon (V) Special details

**Table:**

Geometry. All esds (except the esd in the dihedral angle between two l.s. planes) are estimated using the full covariance matrix. The cell esds are taken into account individually in the estimation of esds in distances, angles and torsion angles; correlations between esds in cell parameters are only used when they are defined by crystal symmetry. An approximate (isotropic) treatment of cell esds is used for estimating esds involving l.s. planes.

### Tetraaluminium diruthenium pentasilicon (V) Fractional atomic coordinates and isotropic or equivalent isotropic displacement parameters (Å^2^)

**Table:**

	*x*	*y*	*z*	*U*_iso_*/*U*_eq_	Occ. (<1)
Ru1	0.25176 (2)	0.12442 (2)	0.11292 (2)	0.00469 (5)	
Si2	0.000000	0.50890 (5)	0.16887 (2)	0.00820 (9)	
Si3	0.000000	0.28515 (5)	0.56422 (2)	0.00938 (9)	
Si4	0.000000	0.10304 (5)	0.04648 (3)	0.01097 (9)	
Si5	0.000000	0.23256 (5)	0.17514 (2)	0.0069 (2)	0.65 (4)
Al5	0.000000	0.23256 (5)	0.17514 (2)	0.0069 (2)	0.35 (4)
Al6	0.26445 (6)	0.13651 (5)	0.250000	0.01175 (12)	
Si7	0.35637 (4)	0.38251 (3)	0.17041 (2)	0.00739 (13)	0.63 (2)
Al7	0.35637 (4)	0.38251 (3)	0.17041 (2)	0.00739 (13)	0.37 (2)
Al8	0.18469 (5)	0.37313 (3)	0.04292 (2)	0.01109 (9)	

### Tetraaluminium diruthenium pentasilicon (V) Atomic displacement parameters (Å^2^)

**Table:**

	*U*^11^	*U*^22^	*U*^33^	*U*^12^	*U*^13^	*U*^23^
Ru1	0.00473 (7)	0.00517 (7)	0.00417 (7)	0.00013 (3)	−0.00034 (2)	0.00002 (2)
Si2	0.00592 (18)	0.00705 (19)	0.0116 (2)	0.000	0.000	0.00072 (14)
Si3	0.00535 (18)	0.0126 (2)	0.01020 (19)	0.000	0.000	−0.00283 (15)
Si4	0.00543 (19)	0.0197 (2)	0.00778 (19)	0.000	0.000	−0.00166 (15)
Si5	0.0065 (3)	0.0070 (3)	0.0073 (3)	0.000	0.000	−0.00015 (14)
Al5	0.0065 (3)	0.0070 (3)	0.0073 (3)	0.000	0.000	−0.00015 (14)
Al6	0.0146 (3)	0.0162 (3)	0.0045 (2)	0.00041 (17)	0.000	0.000
Si7	0.0068 (2)	0.00576 (18)	0.00963 (19)	0.00060 (10)	−0.00059 (11)	0.00062 (9)
Al7	0.0068 (2)	0.00576 (18)	0.00963 (19)	0.00060 (10)	−0.00059 (11)	0.00062 (9)
Al8	0.0175 (2)	0.00624 (17)	0.00951 (17)	0.00015 (11)	−0.00498 (14)	0.00012 (10)

### Tetraaluminium diruthenium pentasilicon (V) Geometric parameters (Å, º)

**Table:**

Ru1—Al6	2.3986 (1)	Si3—Si7^x^	2.6742 (5)
Ru1—Si3^i^	2.4385 (2)	Si3—Si7^xi^	2.6742 (5)
Ru1—Si4	2.4703 (2)	Si4—Si4^xii^	2.4337 (9)
Ru1—Si7^ii^	2.5317 (3)	Si4—Si5	2.5192 (6)
Ru1—Si2^iii^	2.5647 (2)	Si4—Al8^xiii^	2.8622 (5)
Ru1—Al8	2.5727 (3)	Si4—Al8	2.8622 (5)
Ru1—Al8^ii^	2.5853 (3)	Si5—Si5^viii^	2.6144 (8)
Ru1—Si5	2.6091 (2)	Si5—Al6^ix^	2.7626 (5)
Ru1—Si7	2.6420 (3)	Si5—Al6	2.7626 (5)
Ru1—Al8^iv^	2.7759 (4)	Al6—Si7^viii^	2.6918 (5)
Si2—Si5	2.4339 (6)	Al6—Si7	2.6918 (5)
Si2—Si3^v^	2.5736 (6)	Al6—Si7^ii^	2.8309 (5)
Si2—Al6^vi^	2.7204 (5)	Al6—Si7^xiv^	2.8309 (5)
Si2—Al6^vii^	2.7204 (5)	Si7—Si7^xv^	2.4792 (7)
Si2—Si2^viii^	2.8334 (8)	Si7—Al8	2.6755 (5)
Si3—Si4^viii^	2.5108 (6)	Si7—Si7^viii^	2.7796 (6)
Si3—Al8^ix^	2.5768 (5)	Al8—Al8^xvi^	2.6890 (6)
Si3—Al8^viii^	2.5768 (5)	Al8—Al8^iv^	2.8658 (7)
			
Al6—Ru1—Si3^i^	107.063 (16)	Si4—Si5—Ru1^xiii^	57.561 (8)
Al6—Ru1—Si4	120.811 (16)	Ru1—Si5—Ru1^xiii^	112.774 (15)
Si3^i^—Ru1—Si4	129.335 (13)	Si2—Si5—Si5^viii^	92.579 (13)
Al6—Ru1—Si7^ii^	70.025 (14)	Si4—Si5—Si5^viii^	153.104 (14)
Si3^i^—Ru1—Si7^ii^	137.435 (12)	Ru1—Si5—Si5^viii^	114.611 (8)
Si4—Ru1—Si7^ii^	78.346 (13)	Ru1^xiii^—Si5—Si5^viii^	114.612 (8)
Al6—Ru1—Si2^iii^	66.376 (15)	Si2—Si5—Al6^ix^	109.080 (15)
Si3^i^—Ru1—Si2^iii^	61.852 (12)	Si4—Si5—Al6^ix^	106.475 (13)
Si4—Ru1—Si2^iii^	152.066 (14)	Ru1—Si5—Al6^ix^	140.67 (2)
Si7^ii^—Ru1—Si2^iii^	79.843 (11)	Ru1^xiii^—Si5—Al6^ix^	52.949 (6)
Al6—Ru1—Al8	116.532 (14)	Si5^viii^—Si5—Al6^ix^	61.760 (9)
Si3^i^—Ru1—Al8	75.784 (12)	Si2—Si5—Al6	109.080 (15)
Si4—Ru1—Al8	69.127 (14)	Si4—Si5—Al6	106.475 (13)
Si7^ii^—Ru1—Al8	145.151 (12)	Ru1—Si5—Al6	52.948 (6)
Si2^iii^—Ru1—Al8	134.919 (12)	Ru1^xiii^—Si5—Al6	140.67 (2)
Al6—Ru1—Al8^ii^	120.008 (14)	Si5^viii^—Si5—Al6	61.760 (9)
Si3^i^—Ru1—Al8^ii^	85.859 (13)	Al6^ix^—Si5—Al6	111.41 (2)
Si4—Ru1—Al8^ii^	84.229 (14)	Ru1—Al6—Ru1^viii^	172.70 (3)
Si7^ii^—Ru1—Al8^ii^	63.042 (11)	Ru1—Al6—Si7^viii^	124.360 (19)
Si2^iii^—Ru1—Al8^ii^	70.363 (13)	Ru1^viii^—Al6—Si7^viii^	62.218 (9)
Al8—Ru1—Al8^ii^	123.409 (15)	Ru1—Al6—Si7	62.216 (9)
Al6—Ru1—Si5	66.810 (15)	Ru1^viii^—Al6—Si7	124.362 (19)
Si3^i^—Ru1—Si5	139.280 (14)	Si7^viii^—Al6—Si7	62.170 (17)
Si4—Ru1—Si5	59.392 (12)	Ru1—Al6—Si2^xix^	122.379 (19)
Si7^ii^—Ru1—Si5	80.473 (12)	Ru1^viii^—Al6—Si2^xix^	59.741 (10)
Si2^iii^—Ru1—Si5	132.966 (13)	Si7^viii^—Al6—Si2^xix^	80.955 (15)
Al8—Ru1—Si5	72.557 (13)	Si7—Al6—Si2^xix^	112.37 (2)
Al8^ii^—Ru1—Si5	133.275 (12)	Ru1—Al6—Si2^iii^	59.741 (10)
Al6—Ru1—Si7	64.344 (14)	Ru1^viii^—Al6—Si2^iii^	122.378 (19)
Si3^i^—Ru1—Si7	63.371 (12)	Si7^viii^—Al6—Si2^iii^	112.37 (2)
Si4—Ru1—Si7	122.988 (13)	Si7—Al6—Si2^iii^	80.955 (15)
Si7^ii^—Ru1—Si7	134.263 (14)	Si2^xix^—Al6—Si2^iii^	62.768 (19)
Si2^iii^—Ru1—Si7	84.862 (11)	Ru1—Al6—Si5	60.241 (11)
Al8—Ru1—Si7	61.722 (10)	Ru1^viii^—Al6—Si5	116.619 (19)
Al8^ii^—Ru1—Si7	147.382 (11)	Si7^viii^—Al6—Si5	103.993 (19)
Si5—Ru1—Si7	79.214 (11)	Si7—Al6—Si5	75.712 (14)
Al6—Ru1—Al8^iv^	165.673 (16)	Si2^xix^—Al6—Si5	171.91 (2)
Si3^i^—Ru1—Al8^iv^	58.803 (12)	Si2^iii^—Al6—Si5	119.817 (12)
Si4—Ru1—Al8^iv^	73.383 (12)	Ru1—Al6—Si5^viii^	116.620 (19)
Si7^ii^—Ru1—Al8^iv^	117.911 (10)	Ru1^viii^—Al6—Si5^viii^	60.241 (11)
Si2^iii^—Ru1—Al8^iv^	102.208 (12)	Si7^viii^—Al6—Si5^viii^	75.712 (14)
Al8—Ru1—Al8^iv^	64.665 (12)	Si7—Al6—Si5^viii^	103.993 (19)
Al8^ii^—Ru1—Al8^iv^	60.083 (11)	Si2^xix^—Al6—Si5^viii^	119.817 (12)
Si5—Ru1—Al8^iv^	124.747 (12)	Si2^iii^—Al6—Si5^viii^	171.91 (2)
Si7—Ru1—Al8^iv^	107.354 (10)	Si5—Al6—Si5^viii^	56.480 (18)
Si5—Si2—Ru1^xvii^	114.396 (11)	Ru1—Al6—Si7^ii^	57.194 (10)
Si5—Si2—Ru1^vii^	114.396 (11)	Ru1^viii^—Al6—Si7^ii^	115.982 (18)
Ru1^xvii^—Si2—Ru1^vii^	113.304 (16)	Si7^viii^—Al6—Si7^ii^	175.46 (2)
Si5—Si2—Si3^v^	137.34 (2)	Si7—Al6—Si7^ii^	119.334 (12)
Ru1^xvii^—Si2—Si3^v^	56.663 (8)	Si2^xix^—Al6—Si7^ii^	101.833 (18)
Ru1^vii^—Si2—Si3^v^	56.663 (8)	Si2^iii^—Al6—Si7^ii^	72.165 (13)
Si5—Si2—Al6^vi^	112.887 (16)	Si5—Al6—Si7^ii^	72.836 (15)
Ru1^xvii^—Si2—Al6^vi^	53.884 (7)	Si5^viii^—Al6—Si7^ii^	99.76 (2)
Ru1^vii^—Si2—Al6^vi^	131.23 (2)	Ru1—Al6—Si7^xiv^	115.982 (18)
Si3^v^—Si2—Al6^vi^	94.539 (15)	Ru1^viii^—Al6—Si7^xiv^	57.194 (10)
Si5—Si2—Al6^vii^	112.887 (16)	Si7^viii^—Al6—Si7^xiv^	119.335 (12)
Ru1^xvii^—Si2—Al6^vii^	131.23 (2)	Si7—Al6—Si7^xiv^	175.46 (2)
Ru1^vii^—Si2—Al6^vii^	53.884 (7)	Si2^xix^—Al6—Si7^xiv^	72.165 (13)
Si3^v^—Si2—Al6^vii^	94.539 (15)	Si2^iii^—Al6—Si7^xiv^	101.833 (18)
Al6^vi^—Si2—Al6^vii^	96.71 (2)	Si5—Al6—Si7^xiv^	99.76 (2)
Si5—Si2—Si2^viii^	87.421 (13)	Si5^viii^—Al6—Si7^xiv^	72.836 (15)
Ru1^xvii^—Si2—Si2^viii^	112.394 (9)	Si7^ii^—Al6—Si7^xiv^	58.805 (16)
Ru1^vii^—Si2—Si2^viii^	112.394 (9)	Si7^xv^—Si7—Ru1^xvii^	111.632 (8)
Si3^v^—Si2—Si2^viii^	135.240 (14)	Si7^xv^—Si7—Ru1	109.982 (7)
Al6^vi^—Si2—Si2^viii^	58.617 (10)	Ru1^xvii^—Si7—Ru1	116.489 (12)
Al6^vii^—Si2—Si2^viii^	58.617 (10)	Si7^xv^—Si7—Si3^i^	62.385 (8)
Ru1^x^—Si3—Ru1^xi^	122.944 (17)	Ru1^xvii^—Si7—Si3^i^	111.082 (14)
Ru1^x^—Si3—Si4^viii^	118.472 (9)	Ru1—Si7—Si3^i^	54.602 (9)
Ru1^xi^—Si3—Si4^viii^	118.472 (9)	Si7^xv^—Si7—Al8	123.630 (11)
Ru1^x^—Si3—Si2^xviii^	61.485 (9)	Ru1^xvii^—Si7—Al8	59.457 (9)
Ru1^xi^—Si3—Si2^xviii^	61.485 (9)	Ru1—Si7—Al8	57.864 (9)
Si4^viii^—Si3—Si2^xviii^	174.90 (2)	Si3^i^—Si7—Al8	70.290 (12)
Ru1^x^—Si3—Al8^ix^	134.314 (19)	Si7^xv^—Si7—Al6	107.139 (13)
Ru1^xi^—Si3—Al8^ix^	67.148 (10)	Ru1^xvii^—Si7—Al6	140.547 (17)
Si4^viii^—Si3—Al8^ix^	68.456 (15)	Ru1—Si7—Al6	53.439 (8)
Si2^xviii^—Si3—Al8^ix^	107.696 (16)	Si3^i^—Si7—Al6	92.927 (14)
Ru1^x^—Si3—Al8^viii^	67.148 (10)	Al8—Si7—Al6	103.982 (14)
Ru1^xi^—Si3—Al8^viii^	134.314 (19)	Si7^xv^—Si7—Si7^viii^	89.999 (1)
Si4^viii^—Si3—Al8^viii^	68.456 (15)	Ru1^xvii^—Si7—Si7^viii^	113.363 (7)
Si2^xviii^—Si3—Al8^viii^	107.696 (17)	Ru1—Si7—Si7^viii^	112.333 (7)
Al8^ix^—Si3—Al8^viii^	76.42 (2)	Si3^i^—Si7—Si7^viii^	133.900 (10)
Ru1^x^—Si3—Si7^x^	62.028 (9)	Al8—Si7—Si7^viii^	146.312 (11)
Ru1^xi^—Si3—Si7^x^	110.212 (16)	Al6—Si7—Si7^viii^	58.914 (9)
Si4^viii^—Si3—Si7^x^	100.475 (18)	Si7^xv^—Si7—Al6^vi^	111.613 (13)
Si2^xviii^—Si3—Si7^x^	84.035 (16)	Ru1^xvii^—Si7—Al6^vi^	52.782 (8)
Al8^ix^—Si3—Si7^x^	162.921 (18)	Ru1—Si7—Al6^vi^	137.671 (17)
Al8^viii^—Si3—Si7^x^	112.484 (11)	Si3^i^—Si7—Al6^vi^	161.207 (16)
Ru1^x^—Si3—Si7^xi^	110.212 (16)	Al8—Si7—Al6^vi^	103.230 (14)
Ru1^xi^—Si3—Si7^xi^	62.028 (9)	Al6—Si7—Al6^vi^	105.829 (11)
Si4^viii^—Si3—Si7^xi^	100.475 (18)	Si7^viii^—Si7—Al6^vi^	60.597 (8)
Si2^xviii^—Si3—Si7^xi^	84.035 (16)	Ru1—Al8—Si3^viii^	103.213 (14)
Al8^ix^—Si3—Si7^xi^	112.484 (11)	Ru1—Al8—Ru1^xvii^	117.073 (14)
Al8^viii^—Si3—Si7^xi^	162.921 (18)	Si3^viii^—Al8—Ru1^xvii^	137.009 (16)
Si7^x^—Si3—Si7^xi^	55.233 (16)	Ru1—Al8—Si7	60.413 (10)
Si4^xii^—Si4—Ru1^xiii^	111.712 (13)	Si3^viii^—Al8—Si7	162.927 (17)
Si4^xii^—Si4—Ru1	111.713 (13)	Ru1^xvii^—Al8—Si7	57.501 (9)
Ru1^xiii^—Si4—Ru1	123.183 (18)	Ru1—Al8—Al8^xvi^	166.190 (12)
Si4^xii^—Si4—Si3^viii^	87.82 (2)	Si3^viii^—Al8—Al8^xvi^	81.071 (16)
Ru1^xiii^—Si4—Si3^viii^	108.238 (13)	Ru1^xvii^—Al8—Al8^xvi^	63.478 (11)
Ru1—Si4—Si3^viii^	108.238 (13)	Si7—Al8—Al8^xvi^	115.988 (15)
Si4^xii^—Si4—Si5	158.73 (3)	Ru1—Al8—Ru1^iv^	115.335 (12)
Ru1^xiii^—Si4—Si5	63.047 (10)	Si3^viii^—Al8—Ru1^iv^	54.048 (10)
Ru1—Si4—Si5	63.046 (10)	Ru1^xvii^—Al8—Ru1^iv^	114.514 (12)
Si3^viii^—Si4—Si5	113.45 (2)	Si7—Al8—Ru1^iv^	134.909 (17)
Si4^xii^—Si4—Al8^xiii^	127.17 (2)	Al8^xvi^—Al8—Ru1^iv^	56.439 (12)
Ru1^xiii^—Si4—Al8^xiii^	57.123 (9)	Ru1—Al8—Si4	53.750 (9)
Ru1—Si4—Al8^xiii^	115.901 (18)	Si3^viii^—Al8—Si4	54.678 (14)
Si3^viii^—Si4—Al8^xiii^	56.864 (14)	Ru1^xvii^—Al8—Si4	144.883 (17)
Si5—Si4—Al8^xiii^	69.132 (14)	Si7—Al8—Si4	108.417 (15)
Si4^xii^—Si4—Al8	127.17 (2)	Al8^xvi^—Al8—Si4	134.546 (16)
Ru1^xiii^—Si4—Al8	115.902 (18)	Ru1^iv^—Al8—Si4	97.920 (13)
Ru1—Si4—Al8	57.123 (9)	Ru1—Al8—Al8^iv^	61.104 (11)
Si3^viii^—Si4—Al8	56.864 (14)	Si3^viii^—Al8—Al8^iv^	68.684 (15)
Si5—Si4—Al8	69.132 (14)	Ru1^xvii^—Al8—Al8^iv^	144.14 (2)
Al8^xiii^—Si4—Al8	67.682 (18)	Si7—Al8—Al8^iv^	103.923 (19)
Si2—Si5—Si4	114.32 (2)	Al8^xvi^—Al8—Al8^iv^	109.65 (2)
Si2—Si5—Ru1	110.218 (11)	Ru1^iv^—Al8—Al8^iv^	54.231 (12)
Si4—Si5—Ru1	57.562 (8)	Si4—Al8—Al8^iv^	66.587 (13)
Si2—Si5—Ru1^xiii^	110.218 (11)		

Symmetry codes: (i) −*x*+1/2, −*y*+1/2, *z*−1/2; (ii) −*x*+1/2, *y*−1/2, *z*; (iii) *x*+1/2, *y*−1/2, *z*; (iv) −*x*+1/2, −*y*+1/2, −*z*; (v) −*x*, −*y*+1, *z*−1/2; (vi) −*x*+1/2, *y*+1/2, −*z*+1/2; (vii) *x*−1/2, *y*+1/2, *z*; (viii) *x*, *y*, −*z*+1/2; (ix) −*x*, *y*, −*z*+1/2; (x) −*x*+1/2, −*y*+1/2, *z*+1/2; (xi) *x*−1/2, −*y*+1/2, *z*+1/2; (xii) −*x*, −*y*, −*z*; (xiii) −*x*, *y*, *z*; (xiv) −*x*+1/2, *y*−1/2, −*z*+1/2; (xv) −*x*+1, *y*, *z*; (xvi) *x*, −*y*+1, −*z*; (xvii) −*x*+1/2, *y*+1/2, *z*; (xviii) −*x*, −*y*+1, *z*+1/2; (xix) *x*+1/2, *y*−1/2, −*z*+1/2.
